# Supplementary material for: Estimates of the global burden of Japanese encephalitis and the impact of vaccination from 2000-2015
Source: eLife. 2020 May 26;9:e51027. doi: 10.7554/eLife.51027 (PMC7282807; doi:10.7554/eLife.51027)
Supplement: Figure 2—source data 1. — Abbreviation: AUS: Australia, BGD: Bangladesh, BRN: Brunei, BTN: Bhutan, CHN: China, IDN: Indonesia, IND: India, JPN: Japan, KHM: Cambodia, KOR: South Korea, LAO: Laos, LKA: Sri Lanka, MMR: Myanmar, MYS: Malaysia, NPL: Nepal, PAK: Pakistan, PHL: Philippines, PNG: Papua New Guinea, PRK: North Korea, RUS: Russia, SGP: Singapore, THA: Thailand, TLS: Timor-Leste, TWN: Taiwan, VNM: Vietnam. [file elife-51027-fig2-data1.docx]

Fig2- Supp1. Studies that contain age-stratified case data from the systematic review. Abbreviation: AUS: Australia, BGD: Bangladesh, BRN: Brunei, BTN: Bhutan, CHN: China, IDN: Indonesia, IND: India, JPN: Japan, KHM: Cambodia, KOR: South Korea, LAO: Laos, LKA: Sri Lanka, MMR: Myanmar, MYS: Malaysia, NPL: Nepal, PAK: Pakistan, PHL: Philippines, PNG: Papua New Guinea, PRK: North Korea, RUS: Russia, SGP: Singapore, THA: Thailand, TLS: Timor-Leste, TWN: Taiwan, VNM: Vietnam

| Endemic areas | Author/  Reference | Time | Age groups | Vaccination program | Surveillance system | Catchment areas | Patient recruitment | Cases confirmation |
| --- | --- | --- | --- | --- | --- | --- | --- | --- |
| BGD | M. Jahangir Hossain et al [1] | 06/2003-07/2005 | 00-05;06-10;11-15;16-20;21-40;41-99 | NA | Hospital-based | 4 tertiary care hospitals at Dhaka, Mymensingh, Rajshahi, Sylhet | Daily review of admission logbooks for AES cases with CSF, pleocytosis available, and no local bacterial test positive. | 1) IgM against JEV in CSF, or 2) IgM against JEV in serum with a JEV PRNT 90 titer ≥ 20 and a JEV PRNT 90 to DENV PRNT 90 titer ratio ≥ 4. |
| Low or high CHN | Zundong Yin et al [2] | 07/2006-09/2008 for Jinan and Yichang; 04/2007-09/2008 for Shijiazhuang and Guigang | 00-01;02-04;05-14;15-29;30-44;45-99 | 1981 to 2007: vaccination for people willing to pay. Hubei, Hebei, and Guangxi have mass vaccination campaign before JE seasons. Shandong routine immunization since 1986 for 1-10 years old children. From 2008, included in EPI program for disease endemic areas (including in all study sites). | Sentinel hospitals | 4 cities Jinan, Yichang, Shijiazhuang, Guigang | Six hospitals were selected as sentinel hospitals in each prefecture, including the largest general hospital, infectious disease hospital, children’s hospital, and one local county hospital. Collecting diagnostic specimens (serum and cerebrospinal fluid (CSF)) for laboratory testing from all clinical cases of meningitis and encephalitis syndromes (AMES). | IgM ELISA of serum or CSF. Second blood specimens were asked if the first obtained < 7 days after onset. Confirmed if at least one positivity in serum or CSF. |
| High CHN | Yihong Xie et al [3] | 05/2007_06/2012 | continuous | Routine vaccination since 2008 | Sentinel hospitals | Guigang city, Guangxi province | Admitted AME suspected cases in 12 hospitals. (One county was excluded from the study area since 2010 due to the budget limitations.) | IgM ELISA of CSF or serum shown positive and no immunizing against JE virus vaccine within the past 3 months. |
| High CHN | Hu Wei Jun et al [4] | 07/2013-12/2016 | 00-06; 07-14; 15-99 | NA | Sentinel hospitals | Baoji-Shaanxi province | 9 hospitals as monitoring sentinel hospital. All cases that met the surveillance definition of AMES were laboratory tested. | IgM ELISA on CSF and/or serum. |
| High CHN | Si Zhang et al [5] | every years from 2005 to 2012 | 00-09;10-19;20-29;30-39;40-49;50-59;60-69;70-79 | NA | Sentinel hospitals | Longnan City | data reporting system was based on the Control Disease Center of Longnan City, which collected cases from the local health departments and did a cross-comparison with data from the hospitals in rural areas | IgM ELISA in serum (no JE vaccination past month) |
| High CHN | Ye Xufang et al [6] | 04/2006_11/2006 | 0 to 14;15-19;20-39;40-70 | EPI program since 2007 | Case-based surveillance nationwide | Guizhou province | collect specimen from hospitalized reported JE cases in 9 prefectures | clinical confirmed cases tested positive by IgM ELISA or PCR or virus isolation in CSF or serum sample. |
| JPN | Satoru Arai et al [7] | 01/1982-01/2005 | 00-04; 05-09; 10-14; 15-19; 20-24; 25-29; 30-34; 35-39; 40-44; 45-49; 50-54; 55-59; 60-69; 70-79; 80+ | NA | Case-based surveillance nationwide | National | Japanese encephalitis surveillance since 1965. confirmation of notice JE cases; prevalence of JE antibodies among general populations | HI, neutralization test, complement fixation test, PCR, ELISA |
| JPN | Matsunaga Y et al [8] | 01/1982-01/1997 | age group of 10 | NA | Case-based surveillance nationwide | National | NA | NA |
| JPN | Infectious Agent Surveillance Report [9] | 12/2002-12/2008 | age group of 5 | A special intensive immunization program targeting at all age groups, particularly the elderly and children in 1967-1976 | Case-based surveillance nationwide | National | NA | NA |
| JPN | Infectious Agent Surveillance Report [10] | 12/2006-12/2016 | age group of 5 | NA | Case-based surveillance nationwide | National | NA | NA |
| Low and high IDN | Komang Kari et al [11] | 07/2001-12/2003 | 00-04;05-09;10-11 | NA | Sentinel hospitals | Bali | <12 years old admitted tor referred to pediatric department of 10 surveillance centers | anti-JEV IgM in CSF or serum in 1:10 dilution |
| Low and high IDN | Sahat Ompusunggu et al [12] | 01/2005-12/2006 | 00-00; 01-04;05-09;10-15 | NA | Sentinel hospitals | The six sites selected were West Sumatera, West Kalimantan, East Java, West Nusa Tenggara (Lombok Island), East Nusa Tenggara (West Timor), and Papua provinces | children <= 15 years old; definition of AES in WHO. | IgM ELISA from CSF, serum |
| Medium IND | Lalitha Kabilan et al [13] | 07/2002-02/2003 | from 1 to 15 years old | NA | Sentinel hospitals | Cuddalore district | Patients from 2 hospitals Rajah Muthiah Medical College and Hospital (RMMCH) Chidambaram, and Jawaharlal Institute of Post graduate Medical Education and Research (JIPMER), Pondicherry. | IgM-ELISA, PCR from CSF and serum |
| Medium IND | Raghava Potula et al [14] | NA | 00-05;06-10;11-15;15-99 | NA | Sentinel hospitals | Pondicherry | patients admitted to Jawaharlal Institute of Postgraduate Medical Education and Research (JIPMER) Hospital, Pondicherry, India with clinical diagnosis of viral encephalitis. | MAC ELISA (Serum, CSF), microneutralization test (CSF) |
| Medium IND | P. Gunasekaran et al [15] | 01/2007-12/2009 | 00-00;01-04;05-11;12-17;18-100 | NA | Sentinel hospitals | Tamil Nadu | AES cases from government hospitals and private institutions | IgM ELISA from CSF or serum |
| Medium IND | Lalitha Kabilan et al [16] | 11/1998-04/1999 | 0 to 12 | NA | Sentinel hospitals | Tamil Nadu | paediatric AES cases reported to Government Rajaji Hospital, Madurai, which is one of the major referral hospitals in Tamil Nadu, India. | CSF-cell immunofluorescence assay, virus isolation, HI |
| Medium IND | Jitendra Sharma et al [17] | 01/2013-01/2014 | 00-05;06-15;16-30;31-60;61-99 | The campaign for JE vaccination in Dhemaji District was done in April, 2008. However, it was evident from the present study that the vaccination programme could not cover the target population adequately. | NA | Dhemaji district of Assam | acute encephalitis syndrome cases | IgM ELISA test on blood samples |
| Medium IND | Neeru Gupta et al [18] | 06/2004-09/2004 | 00-00; 01-04; 05-11;12-99 | Vaccination was not introduced thus far (2004). | NA | Bellary district | The investigation consisted of discussions with key health personnel in the Bellary District, clinical discussions with clinicians of the district medical college hospital, reviewing of available records in the district surveillance unit, district hospital (Vijayanagar Institute of Medical Sciences), Bellary and Primary health centres. District surveillance unit data consisted of detailed longitudinal surveillance data on epidemiological, clinical (medical records) and laboratory records of encephalitis patients. | IgM antibody in CSF or four-fold antibody in paired sera |
| Medium IND | Avabratha et al [19] | 01/1995-01/1998 | 00-00;01-02;03-04;05-11 | NA | Hospital-based | Bellary district and neighbouring Raichur , Chitradurga , Kurnool, and Anantapur districts | All cases of encephalitis under 12 years of age admitted in Paediatric wards of Vijayanagara Institute of Medical Sciences, Bellary, Karnataka during two epidemics(years 1995-96 and 1996-97) were included in this study | IgM ELISA of CSF, serum |
| Medium IND | A.C. Phukan et al [20] | 06/2000-08/2002 | 01-06; 07-12; 13-20; 21-40; 41-60; 60-99 | NA | Sentinel hospitals | Different hospitals in Assam | clinically suspected viral encephalitis patients admitted to the hospitals | IgM-ELISA from CSF and serum |
| Medium IND | Yogesh K. Gurav et al [21] | 06/2014_08/2014 | 00-09; 10-19; 20-39; 40-59; 60-99 | Campaign vaccination among children old 1–15 years followed by routine vaccination in five districts (Jalpaiguri, Darjeeling, Dakshin Dinajpur, Uttar Dinajpur, and Malda) in 2013. Details are in the paper. | Hospital-based | Northen districts of West Bengal: Jalpaiguri, Darjeeling, Dakshin Dinajpur, Uttar Dinajpur, and Cooch Behar | all AES cases admitted in NBMCH, Silliguri, Darjeeling district | IgM ELISA on CSF and/or serum. |
| High IND | P. Jain et al [22] | 01/2011-12/2013 | 00-04; 05-14; 15-99 | Campaign vaccine in from 2006 to 2009 in selected districts. Details are in the paper. | Hospital-based | Uttar Pradesh | AES patients admitted to King George’s Medical University (KGMU), Lucknow -> lab test | IgM ELISA on CSF and/or serum. |
| High IND | Manish Kakkar et al [23] | 07/2012-02/2013 | continuous 1-15 | NA | Population-based | 12 villages in Kushinagar, Uttar Pradesh | Multistage sampling -> 5% village house hold -> then collect whole blood and serum from healthy children 1-15 years old. Children vaccinated against JE in last three months were excluded | IgM-ELISA, PCR from CSF and serum |
| High IND | Nikky Nyari et al [24] | 09/2010-03/2013 | 00-00, 01-04, 05-09, 10-14, 15-19, 20-44, 45-59 | JEV vaccination was started in Uttar Pradesh on 27 May 2007. The vaccination programme covered 97% of targeted 10 million children between 1 and 15 yr-old, in the endemic Districts of Uttar Pradesh. | Sentinel hospitals | Eastern Uttar Pradesh: Gorakhpur district | Patients with clinical symptoms of high grade fever (≥39 °C) for <10 days with any two of the following symptoms: headache, vomiting, unconsciousness, convulsions, abnormal movements, stupor, delirium, altered sensorium, neck rigidity, presence of Kernig’s sign; admitted to district hospital of Gorakhpur, were enrolled for the study | IgM-ELISA from CSF and serum |
| High IND | Parul Jain et al [25] | 01/2011-12/2012 | 00-00;01-04;05-14; 15-49; 50-99 | Campaign vaccine in from 2006 to 2009 in selected districts. Details are in the paper. | Sentinel hospitals | 34/75 districts of UP, north India | consecutive patints referred to a viral diagnostic lab with a clinical diagnosis of AES | IgM-ELISA from CSF and serum |
| High IND | Roop Kumari et al [26] | 2009, 2005, 1988, 1985 | varied by studies in different year | Table of vaccinated children | Sentinel hospitals | Gorakhpur division | AES cases admitted to district hospitals | IgM-ELISA from CSF and serum |
| High IND | Prashant Ranjan et al [27] | 01/2008-01/2013 | 00-04;05-09;10-14;15-99 | In Gorakhpur, mass JE vaccination was conducted during 2010 and subsequently the vaccine was introduced in the routine immunization programme in 2011. The administrative coverage was high, ranging between 99 and 100% in different districts. | Sentinel hospitals | Gorakhpur division | Most AES cases are admitted in the BRD Medical College => test for JE in the laboratory of the National Institute of Virology (NIV) at the Baba Raghav Das Medical College, Gorakhpur | IgM ELISA (spec: 85% (96% with CSF and 77% with serum) and sensitivity of 71% (75% with CSF and 71% with serum) |
| KHM | Paul F Horwood et al [28] | 07/2010-12/2013 | 00-00;01-05;06-10;11-15 | NA | Sentinel hospitals | National | average of 5 children (0-14 years old) with AME randomly selected each week in the largest children hospital (90% cases will go there) | PCR, MAC-ELISA, Cell culture isolation of CSF |
| KOR | Eun Ju Lee et al [29] | Every years from 2001 to 2014 | 00-10;10-19;20-29;30-39;40-49;50-59;60-100 | 1984->1995 vaccination annually for 3-15 years old. | Case-based surveillance nationwide | National | NA | NA |
| KOR | Jun-Sang Sunwoo et al [30] | 01/2010-01/2016 | 00-09; 10-19; 20-29; 30-39; 40-49; 50-59; 60-69; 70-99 | There is a table of distributed vaccine doses | Case-based surveillance nationwide | National | electrical record from Korea Centers for Disease Control and Prevention | IgM-ELISA from CSF and serum |
| LAO | Catrin E. Moore et al [31] | 01/2001-04/2008 | 00-04->77-80 | NA | Hospital-based | Mahosot Hospital, Vientiane | The responsible physician felt the patients have a CNS infection (no formal definition) | IgM MAC-ELISA of CSF |
| LKA | Epidemiology Unit Ministry of Health [32] | every year from 2006 to 2010 | age group of 5 until 60 years old | Routine vaccination since 1988 for 1-10 age group | Case-based surveillance nationwide | National | All notified cases of encephalitis during the week of concern is entered in in the Weekly Return of communicable Diseases (WRCD) | IgM ELISA of serum or CSF, detection of the JE virus, antigen or genome in brain, spinal cord by immunochemistry or immunofluorescence or PCR. |
| Low NPL | Jeffrey Partridge et al [33] | 01/2006-01/2007 | 00-00; 01-14; 15-99 |  | Case-based surveillance nationwide | Kathmandu Valley (3 district) | AES cases | IgM-ELISA from CSF and serum |
| Low NPL | Anuj Bhattachan et al [34] | 01/2007-01/2008 | 00-00;01-14;15-80 | NA | Case-based surveillance nationwide | hill or mountain districts (excluding 3 hill districts of Kathmandu Valley) | surveillance medical officers recruit AES patients | IgM ELISA from CSF or serum |
| Low and high NPL | Thomas F. Wierzba et al [35] | 05/2004-04/2006 | 00-00; 01-04; 05-14; 15-19; 20-34; 35-79 | Campaign vaccine in from 1999 to 2001 in selected districts. Details are in the paper. | Sentinel hospitals | Terai and non-Terai region | AES cases admitted to 64 referral hospitals | IgM-ELISA from CSF and serum |
| High NPL | T. Akiba et al [36] | 01/1997-01/1998 | 00-05;06-15;16-99 | NA | Hospital-based | 6 health facilities at Nepalgunj; 3 hospitals in Bake and Bardia districts => western Terai | retrospective study - information of JE cases collected in 3 epidemic districts (Banke, Bardia and Kailali) in 1997 were reviewed. | (1) clinical symptoms including high fever, altered sensory, neck rigidity and unconsciousness, (2) exclusion of malaria, bacterial meningitis and typhoid fever through the microscopic observation of thick blood, CSF test and Wider test and (3) follow up for several days. |
| MYS | Nur Izati Mustapa et al [37] | 01/2006-01/2014 | | Sarawak had included JE vaccination in their Extended Programme of Immunization (EPI) since 2001. Currently, JE vaccine has not been included in the EPI in Peninsular Malaysia | NA | National | clinical specimens received from various hospitals | MAC ELISA of Serum or CSF |
| PHL | Anna Lena Lopez et al [38] | 01/2011-03/2014 | 00-01;02-04;05-09;10-14;15-18;19-99 | No vaccination | Case-based surveillance nationwide | National | case-based sentinel surveillance with JE laboratory confirmation from 5 hospitals that represent the major regions of the country | based on WHO surveillance standards |
| THA | Sonja J. Olsen et al [39] | 07/2003-08/2005 | Age group of 5 | In Thailand, nationwide JE vaccination began in 2000 but start at high risk areas since 1990 | Hospital-based | 5 hospitals in Bangkok and 2 hospitals Hat Yai | enrollment criteria (1: fever or hypothemia, 2: acute brain dysfunction 14 days prior to admission, 3: indication for lumbar punture) + subclinical symptoms (neuroimaging, EEG, CSF plecytosis) + positive test to JE | IgM ELISA, PRNT from CSF, serum |
| TWN | Li-Ching Hsu et al [40] | 01/2002-01/2013 | 00-09;10-19;20-29;30-39;40-49;50-59;60-100 | Comprehensive vaccination campaign since 1968, children < 3 years old. After 1980s, children > 15 months old are vaccinated. | Case-based surveillance nationwide | National | Taiwanese Survey on the Prevalence of Hyperglycemia, Hyperlipidemia, and Hypertension from 2002; sampled randomly from the national population | IgM, PCR, HI |
| TWN | Yu-Kang Chang et al [41] | every year from 2000 to 2014 | 00-09; 10-19; 20-29; 30-39; 40-49; 50-59; 60+ | Comprehensive vaccination campaign since 1968, children < 3 years old. After 1980s, children > 15 months old are vaccinated. | Case-based surveillance nationwide | National | JE cases reported to the Taiwan Centers for Disease Control (Taiwan CDC). clinical case was defined with an acute onset of fever and a change in mental status and/or a new onset of seizures | IgM ELISA, IgG ELISA, HI, immunohistochemistry, RT-PCR |
| TWN | Ying-Chang Wu et al [42] | 1967-1997 | 00-09;10-19;20-29;30-39;40-99 | Comprehensive vaccination campaign since 1968, children < 3 years old. After 1980s, children > 15 months old are vaccinated. | Case-based surveillance nationwide | National | National active surveillance system | The HI titer of the convalescent serum is 1:160 and has at least a 4-fold rise from titer in the acute phase serum, or the HI titer of either single serum is 1:320 |
| TWN | TWN-CDC [43] | every years from 1998 to 2017 | age group of 5 until 70 years old | NA | Case-based surveillance nationwide | National and subnational | National active surveillance system | NA |
| VNM | Nghia Ho Dang Trung et al [44] | 08/2007-04/2010 | 00-00;01-04;05-14;15-29;30-44;45-59;60-100 | NA | Sentinel hospitals | 13 hospitals in central and southern | patients meet the criteria (>1m old, fever, 1 of {headache, neck stiffness, altered consciousness, and focal neurological signs}, CSF taken. | Culture (CSF and blood), IgM ELISA (CSF) |
| VNM | Nguyen Thu Yen et al [45] | 01/2004-01/2006 | 00-00;01-05;06-10;11-15;16-100 | NA | Sentinel hospitals | 5 provinces: Thai Binh, Hai Phong, Thanh Hoa, Hai Duong, Bac Giang. | AES cases from surveillance program | IgM ELISA |
| NA* | Hui Zhang et al [46] | 2015 | 01-23;24-45;46-99 | NA | Population | Nyingchi District (3 counties Nyingchi, Mainling Country, Gongbo’gyamda County) - Tibet | 364 healthy human volunteers from the 3 counties | IgM ELISA |
| NA* | Wang Huanyu et al [47] | every year from 1996 to 2005 | 00-05;06-15;16-25;25-99 | Since 1980s. Prior to 2006, 16 provinces with free vaccination program. Since 2006, EPI of JE | Case-based surveillance nationwide | All provinces except Xinjiang Uygur Autonomous, Tibet, and Qinghai | case report system since 1951, electronically record from China CDC since 2004 | ? |
| NA* | Kaliaperumal Kanagasabai et al [48] | 04/2014-04/2017 | 00-00;01-04;05-14;15-44;45-99 | In India, vaccination with SA-14-14-2 vaccine started in 2006. | Case-based surveillance nationwide | assumed to be nation | AES cases from surveillance program | IgM antibodies in serum and/or cerebro-spinal fluid (CSF) |
| NA* | Dhan Kumar Pant et al [49] | 01/2007-01/2016 | 00-00;01-04;05-09;10-14;15-19;20-24;25-100 | NA | Case-based surveillance nationwide | National | AES patients collected from 127 sentinel sites, supported from WHO-IPD | IgM |
| NA* | Anupama Bhattarai et al [50] | 04/2015-10/2015 | 00-15;16-30;31-45;46-60;61-75;75-100 | NA | Case-based surveillance nationwide | National | Samples with AES admitted to hospital in Nepal | IgM ELISA from CSF, serum |
| NA* | J D McCallum et al [51] | 08/1986-10/1986 | age group of 3 | NA | Sentinel hospitals | Koshi Zone | patients with clinically suspected acute encephalitis admitted to study hospitals | IgM ELISA (CSF, serum), IgG ELISA (CSF, serum), virus isolation and IFA (CSF) |
| NA* | Shushil Dev Pant et al [52] | each from 2004 to 2007 | 00-00; 01-04;5-14;15-99 | Following mass immunization campaign in endemic districts of Terai in 2006 | ? | National | NA | NA |
| NA* | Shyam Raj Upreti et al [53] | 01/2004-01/2015 | 00-00; 01-04; 05-14; 15-99 | From 2006 through 2011, mass immunization campaigns were conducted in 31 (41%) of Nepal’s 75 administrative districts, starting with Terai districts with the highest JE disease burden. Details are in the paper | Case-based surveillance nationwide | National | Routine national surveillance for AES cases since 1978: 45 medical facilities (34 sites in 20 Teraj districts + 11 sites in 5 hill districts) + 2 additional sites in hill districts. WHO-recommended case definitions => lab test for JE | MAC ELISA (Serum, CSF) |

*Studies remained unused in the FOI inference because they did not agree with our FOI inference rules. Specifically, there were six studies that contained national data in China, India, and Nepal. These studies were then used for a sensitivity analysis to compare the burdens estimated from using subnational and national data.

# Reference

1. Hossain MJ, Gurley ES, Montgomery S, Petersen L, Sejvar J, Fischer M, et al. Hospital-based surveillance for Japanese encephalitis at four sites in Bangladesh, 2003-2005. Am J Trop Med Hyg. 2010;82(2):344–9.

2. Yin Z, Wang H, Yang J, Luo H, Li Y, Hadler SC, et al. Japanese encephalitis disease burden and clinical features of Japanese encephalitis in four cities in the People’s Republic of China. Am J Trop Med Hyg [Internet]. 2010;83(4):766–73. Available from: http://www.ncbi.nlm.nih.gov/pubmed/20889863

3. Xie Y, Tan Y, Chongsuvivatwong V, Wu X, Bi F, Hadler SC, et al. A population-based acute meningitis and encephalitis syndromes surveillance in Guangxi, China, May 2007- June 2012. PLoS One. 2015;10(12):1–12.

4. Hu Wei Jun, Zhonghua Yu Fang, Yi Xue Za Zhi. [ Quality of case report of Japanese Encephalitis after the establishment of surveillance on acute meningitis and encephalitis syndrome in Baoji ]. 2017;

5. Zhang S, Lu Z, Liu H, Xiao X, Zhao Z, Bao G, et al. Incidence of Japanese encephalitis, visceral leishmaniasis and malaria before and after the Wenchuan earthquake, in China. Acta Trop [Internet]. 2013;128(1):85–9. Available from: http://dx.doi.org/10.1016/j.actatropica.2013.06.015

6. Ye X, Wang H, Fu S, Gao X, Zhao S, Liu C, et al. Etiological spectrum of clinically diagnosed japanese encephalitis cases reported in guizhou province, China, in 2006. J Clin Microbiol. 2010;48(4):1343–9.

7. Arai S, Matsunaga Y, Takasaki T, Tanaka-Taya K, Taniguchi K, Okabe N, et al. Japanese encephalitis: Surveillance and elimination effort in Japan from 1982 to 2004. Jpn J Infect Dis. 2008;61(5):333–8.

8. Matsunaga Y, Yabe S, Taniguchi K, Nakayama M, Kurane I. [Current status of Japanese encephalitis in Japan]. Kansenshogaku Zasshi [Internet]. 1999 Feb [cited 2018 Dec 6];73(2):97–103. Available from: http://www.ncbi.nlm.nih.gov/pubmed/10213985

9. Infectious Agent Surveillance Report. Japanese encephalitis, Japan, 2003-2008. 2009;30(6):147–8. Available from: http://idsc.nih.go.jp/iasr/30/352/tpc352.html

10. Infectious Agent Surveillance Report. Japanese encephalitis, Japan, 2007-2016. 2017;38(8):151–2.

11. Kari K, Liu W, Gautama K, Jr MPM, Clemens JD, Nisalak A, et al. A hospital-based surveillance for Japanese encephalitis in Bali , Indonesia. 2006;7:2–8.

12. Ompusunggu S, Hills SL, Maha MS, Moniaga VA, Susilarini NK, Widjaya A, et al. Confirmation of Japanese encephalitis as an endemic human disease through sentinel surveillance in Indonesia. Am J Trop Med Hyg. 2008;79(6):963–70.

13. Kabilan L, Rajendran R, Arunachalam N, Ramesh S, Srinivasan S, Samuel PP, et al. Japanese encephalitis in India: an overview. Indian J Pediatr. 2004;71(7):609–15.

14. Potula R, Badrinath S, Srinivasan S. Japanese encephalitis in and around Pondicherry, South India: A clinical appraisal and prognostic indicators for the outcome. J Trop Pediatr. 2003;49(1):48–53.

15. Gunasekaran P, Kaveri K, Arunagiri K, Mohana S, Kiruba R, Kumar VS, et al. Japanese encephalitis in Tamil Nadu (2007-2009). Indian J Med Res [Internet]. 2012 May [cited 2018 Dec 6];135(5):680–2. Available from: http://www.ncbi.nlm.nih.gov/pubmed/22771601

16. Kabilan L, Edwin N, Balashankar S, Meikandan D, Thenmozhi V, Gajanana A. Japanese encephalitis among paediatric patients with acute encephalitis syndrome in Tamil Nadu, India. Trans R Soc Trop Med Hyg. 2000;94(2):157–8.

17. Jitendra S, Baruah MK, Anjumoni P, Khan SA, Prafulla D. Epidemiology of Japanese encephalitis cases in Dhemaji district of Assam, India. Ann Biol Res. 2014;5(August 1989):50–4.

18. Gupta N, Chatterjee K, Karmakar S, Jain SK, Venkatesh S, Lal S. Bellary, India achieves negligible case fatality due to Japanese encephalitis despite no vaccination: An outbreak investigation in 2004. Indian J Pediatr. 2008;75(1):31–7.

19. Avabratha KS, Sulochana P, Nirmala G, Vishwanath B, Veerashankar M, Bhagyalakshmi K. Japanese Encephalitis in Children in Bellary Karnataka : Clinical Profile and Sequelae. 2012;100–5.

20. Phukan AC, Borah PK, Mahanta J. Japanese encephalitis in Assam, northeast India. Southeast Asian J Trop Med Public Health. 2004;35(3):618–22.

21. Gurav YK, Bondre VP, Tandale B V, Damle RG, Mallick S, Ghosh US, et al. A large outbreak of Japanese encephalitis predominantly among adults in northern region of West Bengal, India. J Med Virol [Internet]. 2016;88(11):2004–11. Available from: http://www.ncbi.nlm.nih.gov/pubmed/27096294

22. Jain P, Singh AK, Khan DN, Pandey M, Kumar R, Garg R, et al. Trend of Japanese encephalitis in Uttar Pradesh, India from 2011 to 2013. Epidemiol Infect. 2016;144(2):363–70.

23. Kakkar M, Dhole TN, Rogawski ET, Chaturvedi S. Public health laboratory surveillance and diagnosis of Japanese encephalitis: Time to revisit. Indian Pediatr. 2016;53(1):33–5.

24. Nyari N, Singh D, Kakkar K, Sharma S, Pandey SN, Dhole TN. Entomological and serological investigation of Japanese encephalitis in endemic area of eastern Uttar Pradesh, India. J Vector Borne Dis. 2015;52(4):321–8.

25. Jain P, Jain A, Kumar A, Prakash S, Khan DN, Singh KP, et al. Epidemiology and etiology of acute encephalitis syndrome in North India. Jpn J Infect Dis. 2014;67(3):197–203.

26. Kumari R, Joshi PL. A review of Japanese encephalitis in Uttar Pradesh, India. WHO South East Asia J Public Heal [Internet]. 2012;1(4):374–95. Available from: http://www.ncbi.nlm.nih.gov/pubmed/28615603

27. Ranjan P, Gore M, Selvaraju S, Kushwaha KP, Srivastava DK, Murhekar M. Changes in acute encephalitis syndrome incidence after introduction of Japanese encephalitis vaccine in a region of India. J Infect. 2014;69(2):200–2.

28. Horwood PF, Duong V, Laurent D, Mey C, Sothy H, Santy K, et al. Aetiology of acute meningoencephalitis in Cambodian children, 2010-2013. Emerg Microbes Infect [Internet]. 2017;6(5):e35-8. Available from: http://dx.doi.org/10.1038/emi.2017.15

29. Lee EJ, Cha GW, Ju YR, Han MG, Lee WJ, Jeong YE. Prevalence of neutralizing antibodies to Japanese encephalitis virus among high-risk age groups in South Korea, 2010. PLoS One. 2016;11(1):1–13.

30. Sunwoo JS, Jung KH, Lee ST, Lee SK, Chu K. Reemergence of Japanese encephalitis in South Korea, 2010–2015. Emerg Infect Dis. 2016;22(10):1841–3.

31. Moore CE, Blacksell SD, Taojaikong T, Jarman RG, Gibbons R V., Lee SJ, et al. A prospective assessment of the accuracy of commercial IgM ELISAs in diagnosis of Japanese encephalitis virus infections in patients with suspected central nervous system infections in Laos. Am J Trop Med Hyg. 2012;87(1):171–8.

32. Epidemiology Unit Ministry of Health. Japanese Encephalitis A manual for Medical Officers of Health. 2012;(January).

33. Partridge J, Ghimire P, Sedai T, Bista MB, Banerjee M. Endemic Japanese encephalitis in the Kathmandu valley, Nepal. Am J Trop Med Hyg [Internet]. 2007;77(6):1146–9. Available from: http://www.ncbi.nlm.nih.gov/pubmed/18165538

34. Bhattachan A, Amatya S, Sedai TR, Upreti SR, Partridge J. Japanese Encephalitis in Hill and Mountain Districts, Nepal. Emerg Infect Dis [Internet]. 2009 Oct [cited 2018 Dec 6];15(10):1691–2. Available from: http://www.ncbi.nlm.nih.gov/pubmed/19861079

35. Wierzba TF, Ghimire P, Malla S, Banerjee MK, Shrestha S, Khanal B, et al. Laboratory-based Japanese encephalitis surveillance in Nepal and the implications for a national immunization strategy. Am J Trop Med Hyg [Internet]. 2008;78(6):1002–6. Available from: http://www.ncbi.nlm.nih.gov/pubmed/18541784

36. AKIBA T, Osaka K, TANG S, NAKAYAMA M, Yamamoto A, Kurane I, et al. Analysis of Japanese encephalitis epidemic in Western Nepal in 1997. Epidemiol Infect [Internet]. 2001;126(1):81–8. Available from: http://www.pubmedcentral.nih.gov/articlerender.fcgi?artid=2869676&tool=pmcentrez&rendertype=abstract%5Cnhttp://journals.cambridge.org/abstract_S0950268801004769

37. Mustapa NI, Saraswathy Subramaniam TS, Mohd Ruslan NA, Kassim FM, Saat Z. Japanese encephalitis in Malaysia: Review of laboratory data from 2006 to 2013. Southeast Asian J Trop Med Public Health. 2016;47(4):759–65.

38. Lopez AL, Aldaba JG, Roque VG, Tandoc AO, Sy K, Espino FE, et al. Epidemiology of Japanese Encephalitis in the Philippines : A Systematic Review. 2015;73(January 2011):1–17.

39. Olsen SJ, Supawat K, Campbell AP, Anantapreecha S, Liamsuwan S, Tunlayadechanont S, et al. Japanese encephalitis virus remains an important cause of encephalitis in Thailand. Int J Infect Dis [Internet]. 2010;14(10):e888–92. Available from: http://dx.doi.org/10.1016/j.ijid.2010.03.022

40. Hsu LC, Chen YJ, Hsu FK, Huang JH, Chang CM, Chou P, et al. The incidence of Japanese encephalitis in Taiwan--a population-based study. PLoS Negl Trop Dis [Internet]. 2014;8(7):e3030. Available from: http://www.ncbi.nlm.nih.gov/pubmed/25058573

41. Chang YK, Chang HL, Wu HS, Chen KT. Epidemiological features of Japanese encephalitis in Taiwan from 2000 to 2014. Am J Trop Med Hyg. 2017;96(2):382–8.

42. Wu YC, Huang YS, Chien LJ, Lin TL, Yueh YY, Tseng WL, et al. The epidemiology of Japanese encephalitis on Taiwan during 1966-1997. Am J Trop Med Hyg. 1999;61(1):78–84.

43. Taiwan CDC. Taiwan National Infectious Disease Statistics System - Japanese encephalitis [Internet]. Available from: https://nidss.cdc.gov.tw/en/SingleDisease.aspx?dc=1&dt=3&disease=0620

44. Ho Dang Trung N, Le Thi Phuong T, Wolbers M, Nguyen van Minh H, Nguyen Thanh V, Van MP, et al. Aetiologies of central nervous system infection in Viet Nam: A prospective provincial hospital-based descriptive surveillance study. PLoS One. 2012;7(5).

45. Yen NT, Duffy MR, Hong NM, Hien NT, Fischer M, Hills SL. Surveillance for Japanese encephalitis in Vietnam, 1998-2007. Am J Trop Med Hyg. 2010;83(4):816–9.

46. Zhang H, Rehman MU, Li K, Luo H, Lan Y, Nabi F, et al. Epidemiologic Survey of Japanese Encephalitis Virus Infection, Tibet, China, 2015. Emerg Infect Dis [Internet]. 2017 Jun [cited 2018 Dec 6];23(6):1023–4. Available from: http://wwwnc.cdc.gov/eid/article/23/6/15-2115_article.htm

47. Wang H, Li Y, Liang X, Liang G. Japanese encephalitis in mainland china. Jpn J Infect Dis [Internet]. 2009;62(5):331–6. Available from: http://www.ncbi.nlm.nih.gov/pubmed/19762980

48. Kanagasabai K, Joshua V, Ravi M, Sabarinathan R, Kirubakaran BK, Ramachandran V, et al. Epidemiology of Japanese Encephalitis in India: Analysis of laboratory surveillance data, 2014-2017. J Infect [Internet]. 2018 Mar [cited 2018 Dec 6];76(3):317–20. Available from: https://linkinghub.elsevier.com/retrieve/pii/S0163445317303109

49. Kumar Pant D, Tenzin T, Chand R, Kumar Sharma B, Raj Bist P. Spatio-temporal epidemiology of Japanese encephalitis in Nepal, 2007-2015. Forrester N, editor. PLoS One [Internet]. 2017 Jul 26 [cited 2018 Dec 6];12(7):e0180591. Available from: https://dx.plos.org/10.1371/journal.pone.0180591

50. Bhattarai A, Pant ND, Nepal K, Adhikari S, Sharma M, Parajuli P. Anti Japanese encephalitis virus IgM positivity among patients with acute encephalitic syndrome admitted to different hospitals from all over Nepal. Ariën KK, editor. PLoS One [Internet]. 2017 Mar 6 [cited 2018 Dec 6];12(3):e0173434. Available from: http://www.ncbi.nlm.nih.gov/pubmed/28264024

51. McCallum JD. Japanese encephalitis in southeastern Nepal: clinical aspects in the 1986 epidemic. J R Army Med Corps [Internet]. 1991 Feb [cited 2018 Dec 6];137(1):8–13. Available from: http://www.ncbi.nlm.nih.gov/pubmed/1850800

52. Pant SD. Epidemiology of Japanese encephalitis in Nepal. J Nepal Paediatr Soc. 2009;29(1):35–7.

53. Upreti SR, Lindsey NP, Bohara R, Choudhary GR, Shakya S, Gautam M, et al. Updated estimation of the impact of a Japanese encephalitis immunization program with live, attenuated SA 14-14-2 vaccine in Nepal. Recuenco S, editor. PLoS Negl Trop Dis [Internet]. 2017 Sep 21 [cited 2018 Jun 26];11(9):e0005866. Available from: http://dx.plos.org/10.1371/journal.pntd.0005866
