# Supplementary material for: Estimates of the global burden of Japanese encephalitis and the impact of vaccination from 2000-2015
Source: eLife. 2020 May 26;9:e51027. doi: 10.7554/eLife.51027 (PMC7282807; doi:10.7554/eLife.51027)
Supplement: Figure 3—source data 1. — Abbreviation: AUS: Australia, BGD: Bangladesh, BRN: Brunei, BTN: Bhutan, CHN: China, IDN: Indonesia, IND: India, JPN: Japan, KHM: Cambodia, KOR: South Korea, LAO: Laos, LKA: Sri Lanka, MMR: Myanmar, MYS: Malaysia, NPL: Nepal, PAK: Pakistan, PHL: Philippines, PNG: Papua New Guinea, PRK: North Korea, RUS: Russia, SGP: Singapore, THA: Thailand, TLS: Timor-Leste, TWN: Taiwan, VNM: Vietnam. [file elife-51027-fig3-data1.docx]

| Country  **Figure 3- Source Data. Vaccine information and how it was used in our model**. Abbreviation: AUS: Australia, BGD: Bangladesh, BRN: Brunei, BTN: Bhutan, CHN: China, IDN: Indonesia, IND: India, JPN: Japan, KHM: Cambodia, KOR: South Korea, LAO: Laos, LKA: Sri Lanka, MMR: Myanmar, MYS: Malaysia, NPL: Nepal, PAK: Pakistan, PHL: Philippines, PNG: Papua New Guinea, PRK: North Korea, RUS: Russia, SGP: Singapore, THA: Thailand, TLS: Timor-Leste, TWN: Taiwan, VNM: Vietnam. | From literature | Other sources | Information included in the model |
| --- | --- | --- | --- |
| IND (Campaign) | [1] Vaccinated people for every districts from 2006 to 2009. In national scale, they are respectively: 9.3 million, 16.5 million, 16.9 million, 18.1 million.  [2] Mass JE vaccination in Gorakhpur division in 2010, assume coverage to be 99% (total estimated vaccinated people: 4,162,832)  [3] Campaign vaccination in 2 to 15 years old in 2013 with coverage difference in the 5 northern districts of West Bengal : 0.7893, 0.8038, 0.761, 0.6789, 0 (total estimated vaccinated people: 2,219,197)  [4] In 2010, vaccination campaign with 1 dose for 1–15 years of age in Kushinagar district, maybe a part of vaccination program in Gorakhpur division. (total estimated vaccinated people: 1,074,834) | From Gavi  (vaccinated people)  2006:9.3 millions  2007:18.4 millions  2008:16.9 millions  2009:18.1 millions  2010:9.23 millions | Use numbers in the literature for subnational data.  Use 2006-2009 numbers, 2013 in West Bengal from literature and 2010 number from Gavi for national scale data. |
| IND (Routine) | [2] Routine vaccination in Gorakhpur since 2011, coverage ranged 99-100%  [3] Routine vaccination in 1 year old since 2013 in 5 Northern districts in West Bengal, assumed coverage to be 99% | From Gavi  (coverage %)  2009:9  2010:37  2011:63  2012:65  2013:69  2014:36  2015:31  From WHO  (Year\|vaccinated\|coverage%)  2009\|2,000,000\|9  2010\|2,000,000\|27  2011\|4,000,000\|63  2012\|2,000,000\|43  2013\|4,000,000\|69  2014\|NA  2015\|6,000,000\|70 | Used literature information in subnational data.  Use WHO Doses Administered in national data |
| JPN (Campaign) | [5] In 1954-1967, vaccination is voluntary  In 1967-1976, there is an intensive vaccination program in adult and children. | NA | currently did not include this information to the model |
| JPN (Routine) | [6–8] Assumption: routine vaccination coverage from 1976 to 1994 are 50% , from 1995 to 2005 are 80%, from 2005 to 2009 are 0%, from 2010 till now are 50%  Estimated vaccinated from 2010 to 2015: around 550,000 | From WHO  (Year\|vaccinated\|coverage%)  2007\|969,925\|83  2008\|045,158\|4  2009\|232,264\|21  2010\|232,264\|22  2011\|656,048\|61  2012\|2,000,000\|172  2013\|2,000,000\|169  2014\|2,000,000\|145  2015\|1,000,000\|117 | literature information |
| TWN (Campaign) | [9] campaign vaccination in 1968 for 0-3 year old | NA | From the literature |
| TWN (Routine) | [8,9] routine vaccination for 15-27 months old after 1968. Routine vaccination from 1968 to 1974 with 80% coverage, from 1975 to now with 95% coverage. | NA | From the literature |
| KHM (Campaign) | [10] 2009- 2010: Small scale vaccination in 3 districts: total vaccinated children is 50,000 | From Gavi:  vaccinated people in 2013: 309,549 | From literature and Gavi |
| KHM (Routine) | NA | From Gavi(coverage%)  2015:47  From WHO (Year\|vaccinated\|coverage%)  2015\|146,385\| 125% | From WHO |
| KOR (Campaign) | [11] A graph of liters of vaccine distributed, 1963–1998.  In the 1970s, the vaccine coverage rate <5%, increased to 16.8% in 1981. In, 1983, a mass immunization program for children 3 to 15 years age, increased coverage to 60%. Since 1984, vaccine coverage reached almost 90%. | NA | From the literature => the vaccine distributed are relative comparable with coverage reported => use the information in the graph |
| KOR (Routine) | [8,11] Since 2000, routine vaccination starts at 12 months of age, assumed to be 95% => vaccinated estimated to be around 420,000 | From WHO  (Year\|vaccinated\|coverage%)  2006\|382,014\|88  2007\|378,054\|84  2008\|389,308\|84  2009\|413,246\|89  2010\|430,431\|97  2011\|379,682\|85  2012\|441,296\|93  2013\|454,140\|96  2014\|470,825\|96  2015\|423,519\|97 | From 2000 to 2005, use 95% coverage. Since 2015, use vaccinated people reported from WHO |
| NPL (Campaign) | [12] vaccination program in western Terai: 1999: 224,000 ; 2000 and 2001: 378,112  [13] number of vaccinated from 2005 to 2006, 2008 in every high risk districts. In national scale:  2005: 609,147  2006: 2,536,700  2008: 1,892,803  [14] Updated vaccination program information in national scale and target age group from 2006 to 2011 (data for 2010, 2011 is unavailable). Estimated vaccinated children:  2006: 2,972,031  2007:833,587  2008:1,694,545  2009:4,106,200 | From Gavi (vaccinated people)  2005: 609,147  2006: 2,536,700  2008: 1,892,803  2016: 3,440,018 | Year 2006 and 2008 in [14] are comparable to [13] => use [14] numbers in these year because we have better information about vaccinated age group.  Overall, we used these number:  [12]  1999:224,000  2000 and 2001:378,112  [13]  2005:609,147  [14]  2006:2,972,031  2007:833,587  2008:1,694,545  2009:4,106,200  2016:3,440,018 |
| NPL (Routine) | [15] children aged 12–23 months | From Gavi(coverage%)  2009:21  2010:47  2011:54  2012:62  2013:72  2014:78  2015:58  2016:59  2017:91  From WHO  (Year\|vaccinated\|coverage%)  2010\|154,988\|47  2011\|201,277\|54  2012\|260,040\|62  2013\|296,069\|72  2014\|267,738\|78  2015\|302,291\|77  2016\|301,466\|63  2017\|420,494\|67 | Estimated vaccinated people from Gavi:  2009: 133,608.9  2010: 292,958  2011: 329,518.8  2012: 363,743.5  2013: 409,752.7  2014: 433,117.6  2015: 381,504.6  2016: 388,393.4  2017: 475,918.7  => Larger than WHO number => use info from WHO + year 2009 from Gavi |
| THA (Routine) | [8,16] In Thailand, JE vaccination in high risk areas began in 1990, age group 18-24 months. 1-2 million doses administered annually (there are around 800,000 children in 1^st^ age group annually). National vaccination since 2000. | NA | literature information. From 1990 to 2000, increase coverage from 10% to 100% by 10%. From 2000, coverages are assumed to be 99%. |
| CHN (Campaign) | [17] From 1971 to 2003, 650 000 doses of JE vaccine were distributed each year. Low rate coverage around 10% for <10 years old from 1991 to 2003. From 2004 to 2008, number of vaccinated people age 1-10 in Guizhou province.  [18] Voluntary vaccination from 1970s => did not include this in the model. From 2000 to 2013, number of vaccine distributed in Guangxi province (no clear age group vaccinated) | NA | From the literature =>  Guizhou: coverage of < 10 years old are 10% from 1971 to 2003 + number of vaccinated people age 1-10 from 2004 to 2008  Guangxi:  From 2000 to 2007: number of vaccine/3 (assuming 3 doses program for SA-14-14-2 vaccine). From 2008: routine vaccination |
| CHN (Routine) | [8,19,20] Voluntary vaccination since 1971. EPI since 2008  [20,21] routine vaccination in Shandong prefecture since 1986 | From WHO (Year\|vaccinated\|coverage%)  2010\|20,000,000\|99  2011\|20,000,000\|99  2012\|20,000,000\|99  2013\|20,000,000\|99.5  2014\|20,000,000\|99  2015\|20,000,000\|99.6 | We did not include the voluntary vaccination period in the model.  literature information => routine vaccination since 2008 with coverage = 99%.  Routine vaccination in Shandong with coverage = 99% |
| MYS (Routine) | EPI since 2001 in Sarawak [22] | From WHO  (Year\|vaccinated\|coverage%)  2008\|41,784\|99  2010\|40,507\|96  2012\|41,870\|97  2013\|40,334\|98  2014\|38,806\|90  2015\|39,976\|93 | Number of vaccinated people from WHO in 2008, 2010, 2012 to 2015. For other years, assumed coverage to be 99% |
| VNM (Routine) | [23] number of vaccinated people in every province since 1997, 2 doses and 3 doses scheme.  National vaccinated people from 2006 to 2016 for 2\|3 doses scheme  2006 1,016,105\|781,399  2007 1,068,471\|900,923  2008 1,192,699\|1,049,339  2009 1,466,528\|1,193,935  2010 768,955\|657,413  2011 1,346,460\|1,050,803  2012 1,338,752\|1,378,649  2013 1,336,689\|1,180,933  2014 1,786,787\|1,312,534  2015 1,980,804\|1,769,525  2016 1,764,640\|1,770,448  Target group from 1 to 5 years old, around 7,000,000 annually | From Gavi (coverage%)  2006:95  2007:93  2008:88  2009:NA  2010:93  2011:NA  2012:94  2013:91  2014:94  2015:98  From WHO  (Year\|vaccinated\|coverage%)  2006\|1,000,000\|95  2007\|900,923\|93  2008\|1,000,000\|88  2009\|NA  2010\|728,432\|93  2011\|NA  2012\|1,000,000\|94  2013\|1,000,000\|91  2014\|2,000,000\|94  2015\|2,000,000\|96 | Used [23], 2 doses scheme |
| AUS (Routine) | [24,25] EPI since 1995, assuming coverage to be 99% | NA | literature information |
| LKA (Routine) | EPI since 1988, assuming coverage to be 99% => around 260,000 annually | NA | literature information |
| PRK (Campaign) | [26] vaccinated people:  2009: 500,000  2010: 500,000  2013: 500,000  2014: 500,000 | From Gavi (vaccinated people)  2009: 500,000 | From the literature |
| LAO (Campaign) | [27] in 2015, aim to vaccinate 1.5 million children | From Gavi (vaccinated people)  2013: 630,631  2015: 1,142,063 (target population: 1,429,068 ) | From Gavi |
| LAO (Routine) | NA | From Gavi (coverage%)  2015: 59  From WHO (Year\|vaccinated\|coverage%)  2015\|36,369\|20 | WHO information |
| TLS (Campaign) | NA | From Gavi (vaccinated people)  2015: 453,399 | From Gavi |

# Reference

1. New Delhi: Ministry of Health & Family Welfare, editor. Operational guide for Japanese encephalitis vaccination in India. [Internet]. 2010. Available from: http://www.nipccd-earchive.wcd.nic.in/sites/default/files/PDF/Operational Guide Japanese Encephalitis Vaccination in India - 2010-MOHFW.pdf

2. Ranjan P, Gore M, Selvaraju S, Kushwaha KP, Srivastava DK, Murhekar M. Changes in acute encephalitis syndrome incidence after introduction of Japanese encephalitis vaccine in a region of India. J Infect. 2014;69(2):200–2.

3. Gurav YK, Bondre VP, Tandale B V, Damle RG, Mallick S, Ghosh US, et al. A large outbreak of Japanese encephalitis predominantly among adults in northern region of West Bengal, India. J Med Virol [Internet]. 2016;88(11):2004–11. Available from: http://www.ncbi.nlm.nih.gov/pubmed/27096294

4. Ranjan P, Gore M, Selvaraju S, Kushwaha KP, Srivastava DK, Murhekar M. Decline in Japanese encephalitis, Kushinagar District, Uttar Pradesh, India. Emerg Infect Dis [Internet]. 2014;20(8):1406–7. Available from: http://www.embase.com/search/results?subaction=viewrecord&from=export&id=L373678544%5Cnhttp://dx.doi.org/10.3201/eid2008.131403%5Cnhttp://vu.on.worldcat.org/atoztitles/link?sid=EMBASE&issn=10806059&id=doi:10.3201%2Feid2008.131403&atitle=Decline+in+Japanes

5. ISAR. [Human Japanese encephalitis neutralizing antibody status in 2007 - 2016 and Japanese encephalitis vaccination status] [Internet]. 2017. Available from: https://www.niid.go.jp/niid/ja/allarticles/surveillance/2410-iasr/related-articles/related-articles-450/7466-450r06.html

6. Takeshita N, Lim CK, Mizuno Y, Shimbo T, Kotaki A, Ujiie M, et al. Immunogenicity of single-dose Vero cell-derived Japanese encephalitis vaccine in Japanese adults. J Infect Chemother [Internet]. 2014;20(4):238–42. Available from: http://www.ncbi.nlm.nih.gov/pubmed/24485326

7. Harter DH. Japanese Encephalitis and West Nile Viruses. In: Current Topics in Microbiology and Immunology [Internet]. 2002. p. 558–9. Available from: http://archneur.jamanetwork.com/article.aspx?articleid=567672

8. Monath TP. Japanese Encephalitis Vaccines: Current Vaccines and Future Prospects. Vol. 267, Current Topics in Microbiology and Immunology. Springer; 2002. 105-134 p.

9. Hsu LC, Chen YJ, Hsu FK, Huang JH, Chang CM, Chou P, et al. The incidence of Japanese encephalitis in Taiwan--a population-based study. PLoS Negl Trop Dis [Internet]. 2014;8(7):e3030. Available from: http://www.ncbi.nlm.nih.gov/pubmed/25058573

10. PATH. Japanese Encephalitis Vaccine Introduction in Cambodia [Internet]. 2010. p. 1–2. Available from: https://www.path.org/publications/files/CP_cambodia_je_vac_intro.pdf

11. Sohn YM. Japanese encephalitis immunization in South Korea: past, present, and future. Emerg Infect Dis [Internet]. 2000;6(1):17–24. Available from: http://www.ncbi.nlm.nih.gov/pubmed/10653564

12. Wierzba TF, Ghimire P, Malla S, Banerjee MK, Shrestha S, Khanal B, et al. Laboratory-based Japanese encephalitis surveillance in Nepal and the implications for a national immunization strategy. Am J Trop Med Hyg [Internet]. 2008;78(6):1002–6. Available from: http://www.ncbi.nlm.nih.gov/pubmed/18541784

13. World Health Organization. Program for Immunization Preventable Diseases and Child Health Division. Nepal Ministry of Health and Population. Control of Japanese Encephalitis in Nepal, 1978–2012. 2012;

14. Joshi DD. Japanese encephalitis vaccination in children population of Nepal during the year 2005, 2006 and 2008. 2009;29(2):85.

15. Upreti SR, Janusz KB, Schluter WW, Bichha RP, Shakya G, Biggerstaff BJ, et al. Estimation of the impact of a Japanese encephalitis immunization program with live, attenuated SA 14-14-2 vaccine in Nepal. Am J Trop Med Hyg. 2013;88(3):464–8.

16. Olsen SJ, Supawat K, Campbell AP, Anantapreecha S, Liamsuwan S, Tunlayadechanont S, et al. Japanese encephalitis virus remains an important cause of encephalitis in Thailand. Int J Infect Dis [Internet]. 2010;14(10):e888–92. Available from: http://dx.doi.org/10.1016/j.ijid.2010.03.022

17. Zhang L, Luan RS, Jiang F, Rui LP, Liu M, Li YX, et al. Epidemiological characteristics of Japanese encephalitis in Guizhou Province, China, 1971-2009. Biomed Environ Sci [Internet]. 2012;25(3):297–304. Available from: http://www.ncbi.nlm.nih.gov/pubmed/22840580

18. Yang Y, Liang N, Tan Y, Xie Z. Epidemiological trends and characteristics of Japanese encephalitis changed based on the vaccination program between 1960 and 2013 in Guangxi Zhuang Autonomous Region, southern China. Int J Infect Dis [Internet]. 2016;45:135–8. Available from: http://www.ncbi.nlm.nih.gov/pubmed/26972041

19. Wang H, Li Y, Liang X, Liang G. Japanese encephalitis in mainland china. Jpn J Infect Dis [Internet]. 2009;62(5):331–6. Available from: http://www.ncbi.nlm.nih.gov/pubmed/19762980

20. Gao X, Li X, Li M, Fu S, Wang H, Lu Z, et al. Vaccine strategies for the control and prevention of Japanese encephalitis in Mainland China, 1951-2011. PLoS Negl Trop Dis [Internet]. 2014;8(8):e3015. Available from: http://www.ncbi.nlm.nih.gov/pubmed/25121596

21. Yin Z, Wang H, Yang J, Luo H, Li Y, Hadler SC, et al. Japanese encephalitis disease burden and clinical features of Japanese encephalitis in four cities in the People’s Republic of China. Am J Trop Med Hyg [Internet]. 2010;83(4):766–73. Available from: http://www.ncbi.nlm.nih.gov/pubmed/20889863

22. Mustapa NI, Saraswathy Subramaniam TS, Mohd Ruslan NA, Kassim FM, Saat Z. Japanese encephalitis in Malaysia: Review of laboratory data from 2006 to 2013. Southeast Asian J Trop Med Public Health. 2016;47(4):759–65.

23. Choisy M. Vaccine coverages from the WHO EPI in Vietnam [Internet]. [cited 2018 Dec 18]. Available from: https://github.com/choisy/epiVN

24. Campbell G, Hills S, Fischer M, Jacobson J, Hoke C, Hombach J, et al. Estimated global incidence of Japanese encephalitis: Bull World Health Organ [Internet]. 2011;89(10):766–74. Available from: http://www.who.int/bulletin/volumes/89/10/10-085233.pdf

25. Mackenzie JS, Johansen CA, Ritchie SA, van den Hurk AF, Hall RA. Japanese encephalitis as an emerging virus: the emergence and spread of Japanese encephalitis virus in Australasia. Curr Top Microbiol Immunol [Internet]. 2002;267:49–73. Available from: http://www.ncbi.nlm.nih.gov/pubmed/12083000

26. Marks F, Nyambat B, Xu ZY, von Kalckreuth V, Kilgore PE, Seo HJ, et al. Vaccine introduction in the Democratic People’s Republic of Korea. Vaccine. 2015;33(20):2297–300.

27. World Health Organization. GAVI. UNICEF. Lao People’s Democratic Republic to protect more than 1.5 million children from Japanese encephalitis [Internet]. 2015. Available from: http://www.who.int/immunization/newsroom/press/lao_japanese_encephalitis_apr2015/en/
