# Supplementary material for: Estimates of the global burden of Japanese encephalitis and the impact of vaccination from 2000-2015
Source: eLife. 2020 May 26;9:e51027. doi: 10.7554/eLife.51027 (PMC7282807; doi:10.7554/eLife.51027)
Supplement: Figure 4—source data 1. [file elife-51027-fig4-data1.docx]

Figure 4- Source Data. Estimated FOI and studies used/assumptions of 30 endemic areas.

| Endemic areas | Studies used  /assumptions | Inferred FOI -mean (CI 95%) (%) |  | Endemic areas | Studies used  /assumptions | Inferred FOI -mean (CI 95%) (%) |
| --- | --- | --- | --- | --- | --- | --- |
| Australia | Group B* | 1.7 (0.1-7.0) |  | **Sri Lanka** | Sri Lanka [1] | 4.0 (3.1-4.9) |
| Bangladesh | 4 divisions [2] | 6.2 (3.6-9.1) |  | **Myanmar** | Vientiane [3] (group D)* | 7.3 (2.1-11.1) |
| Brunei | 4 divisions [2] (group G)* | 6.2 (3.6-9.1) |  | **Malaysia** | Malaysia [4] | 7.7 (5.5-9.7) |
| Bhutan | 4 divisions [2] (group G)* | 6.2 (3.6-9.1) |  | **Nepal (Low)** | Kathmandu [5], non-Kathmandu [6] | 9.0 (5.6-15.5) |
| China (Low) | Shijiazhuang [7] | 11.1 (5.9-17.5) |  | **Nepal (High)** | West Terai [8,9], non-West Terai [8] | 8.4 (5.9-10.7) |
| China (High) | Baoji [10], Guigang [7,11], Guizhou [12], Longnan [13], Yichang [7], Jinan [7]. | 17.8(3.5-53.9) |  | **Pakistan** | Group B* | 1.7 (0.1-7.0) |
| Indonesia (Low) | 6 provinces [14], Bali [15] | 26.5 (8.7-45.1) |  | **Philippines** | Philippines [16] | 16.5 (11.8-19.3) |
| Indonesia (High) | 6 provinces [14], Bali [15] | 26.5 (8.7-45.1) |  | **Papua New Guinea** | 6 provinces [14], Bali [15] (group E)* | 26.5 (8.7-45.1) |
| India (Low) | Group B* | 1.7 (0.1-7.0) |  | **North Korea** | 2 cities of Thailand [17] (group H)* | 7.7 (5.0-11.0) |
| India (Medium) | Bellary and neighbors [18], Bellary [19], Cuddalore [20], Dhemaji [21], Pondicherry [22], Tamil Nadu [23,24]. | 14.4 (0.9-31.2) |  | **Russia** | Group B* | 1.7 (0.1-7.0) |
| India (High) | 7 districts of Assam, Assam [25], Gorakhpur district [26], Gorakhpur division [27], Kushinagar [28], North Uttar Pradesh [29], Uttar Pradesh [30], North West Bengal [31] | 14.1 (0.1-41.8) |  | **Singapore** | Group B* | 1.7 (0.1-7.0) |
| Japan | Japan [32–35] | 0.1 (0.0-0.2) |  | **Thailand** | 2 cities of Thailand [17] | 7.7 (5.0-11.0) |
| Cambodia | Cambodia [36] | 8.7 (2.0-14.1) |  | **Timor-Leste** | 6 provinces [14], Bali [15] (group D)* | 26.5 (8.7-45.1) |
| South Korea | South Korea [37,38] | 4.1 (2.6-5.7) |  | **Taiwan** | Taiwan [39–42], Central [42], Eastern [42], Kaoping [42], Northern [42], Southern [42], Taipei [42] | 6.1 (1.3-9.3) |
| LAO | Vientiane [3] | 7.3 (2.1-11.1) |  | **VNM** | Middle and South [43], North [44] | 17.8 (7.7-27.8) |

**Areas which age-stratified data is not available.*

# Reference

1. Epidemiology Unit Ministry of Health. Japanese Encephalitis A manual for Medical Officers of Health. 2012;(January).

2. Hossain MJ, Gurley ES, Montgomery S, Petersen L, Sejvar J, Fischer M, et al. Hospital-based surveillance for Japanese encephalitis at four sites in Bangladesh, 2003-2005. Am J Trop Med Hyg. 2010;82(2):344–9.

3. Moore CE, Blacksell SD, Taojaikong T, Jarman RG, Gibbons R V., Lee SJ, et al. A prospective assessment of the accuracy of commercial IgM ELISAs in diagnosis of Japanese encephalitis virus infections in patients with suspected central nervous system infections in Laos. Am J Trop Med Hyg. 2012;87(1):171–8.

4. Mustapa NI, Saraswathy Subramaniam TS, Mohd Ruslan NA, Kassim FM, Saat Z. Japanese encephalitis in Malaysia: Review of laboratory data from 2006 to 2013. Southeast Asian J Trop Med Public Health. 2016;47(4):759–65.

5. Partridge J, Ghimire P, Sedai T, Bista MB, Banerjee M. Endemic Japanese encephalitis in the Kathmandu valley, Nepal. Am J Trop Med Hyg [Internet]. 2007;77(6):1146–9. Available from: http://www.ncbi.nlm.nih.gov/pubmed/18165538

6. Bhattachan A, Amatya S, Sedai TR, Upreti SR, Partridge J. Japanese Encephalitis in Hill and Mountain Districts, Nepal. Emerg Infect Dis [Internet]. 2009 Oct [cited 2018 Dec 6];15(10):1691–2. Available from: http://www.ncbi.nlm.nih.gov/pubmed/19861079

7. Yin Z, Wang H, Yang J, Luo H, Li Y, Hadler SC, et al. Japanese encephalitis disease burden and clinical features of Japanese encephalitis in four cities in the People’s Republic of China. Am J Trop Med Hyg [Internet]. 2010;83(4):766–73. Available from: http://www.ncbi.nlm.nih.gov/pubmed/20889863

8. Wierzba TF, Ghimire P, Malla S, Banerjee MK, Shrestha S, Khanal B, et al. Laboratory-based Japanese encephalitis surveillance in Nepal and the implications for a national immunization strategy. Am J Trop Med Hyg [Internet]. 2008;78(6):1002–6. Available from: http://www.ncbi.nlm.nih.gov/pubmed/18541784

9. AKIBA T, Osaka K, TANG S, NAKAYAMA M, Yamamoto A, Kurane I, et al. Analysis of Japanese encephalitis epidemic in Western Nepal in 1997. Epidemiol Infect [Internet]. 2001;126(1):81–8. Available from: http://www.pubmedcentral.nih.gov/articlerender.fcgi?artid=2869676&tool=pmcentrez&rendertype=abstract%5Cnhttp://journals.cambridge.org/abstract_S0950268801004769

10. Hu Wei Jun, Zhonghua Yu Fang, Yi Xue Za Zhi. [ Quality of case report of Japanese Encephalitis after the establishment of surveillance on acute meningitis and encephalitis syndrome in Baoji ]. 2017;

11. Xie Y, Tan Y, Chongsuvivatwong V, Wu X, Bi F, Hadler SC, et al. A population-based acute meningitis and encephalitis syndromes surveillance in Guangxi, China, May 2007- June 2012. PLoS One. 2015;10(12):1–12.

12. Ye X, Wang H, Fu S, Gao X, Zhao S, Liu C, et al. Etiological spectrum of clinically diagnosed japanese encephalitis cases reported in guizhou province, China, in 2006. J Clin Microbiol. 2010;48(4):1343–9.

13. Zhang S, Lu Z, Liu H, Xiao X, Zhao Z, Bao G, et al. Incidence of Japanese encephalitis, visceral leishmaniasis and malaria before and after the Wenchuan earthquake, in China. Acta Trop [Internet]. 2013;128(1):85–9. Available from: http://dx.doi.org/10.1016/j.actatropica.2013.06.015

14. Ompusunggu S, Hills SL, Maha MS, Moniaga VA, Susilarini NK, Widjaya A, et al. Confirmation of Japanese encephalitis as an endemic human disease through sentinel surveillance in Indonesia. Am J Trop Med Hyg. 2008;79(6):963–70.

15. Kari K, Liu W, Gautama K, Jr MPM, Clemens JD, Nisalak A, et al. A hospital-based surveillance for Japanese encephalitis in Bali , Indonesia. 2006;7:2–8.

16. Lopez AL, Aldaba JG, Roque VG, Tandoc AO, Sy K, Espino FE, et al. Epidemiology of Japanese Encephalitis in the Philippines : A Systematic Review. 2015;73(January 2011):1–17.

17. Olsen SJ, Supawat K, Campbell AP, Anantapreecha S, Liamsuwan S, Tunlayadechanont S, et al. Japanese encephalitis virus remains an important cause of encephalitis in Thailand. Int J Infect Dis [Internet]. 2010;14(10):e888–92. Available from: http://dx.doi.org/10.1016/j.ijid.2010.03.022

18. Avabratha KS, Sulochana P, Nirmala G, Vishwanath B, Veerashankar M, Bhagyalakshmi K. Japanese Encephalitis in Children in Bellary Karnataka : Clinical Profile and Sequelae. 2012;100–5.

19. Gupta N, Chatterjee K, Karmakar S, Jain SK, Venkatesh S, Lal S. Bellary, India achieves negligible case fatality due to Japanese encephalitis despite no vaccination: An outbreak investigation in 2004. Indian J Pediatr. 2008;75(1):31–7.

20. Kabilan L, Rajendran R, Arunachalam N, Ramesh S, Srinivasan S, Samuel PP, et al. Japanese encephalitis in India: an overview. Indian J Pediatr. 2004;71(7):609–15.

21. Jitendra S, Baruah MK, Anjumoni P, Khan SA, Prafulla D. Epidemiology of Japanese encephalitis cases in Dhemaji district of Assam, India. Ann Biol Res. 2014;5(August 1989):50–4.

22. Potula R, Badrinath S, Srinivasan S. Japanese encephalitis in and around Pondicherry, South India: A clinical appraisal and prognostic indicators for the outcome. J Trop Pediatr. 2003;49(1):48–53.

23. Gunasekaran P, Kaveri K, Arunagiri K, Mohana S, Kiruba R, Kumar VS, et al. Japanese encephalitis in Tamil Nadu (2007-2009). Indian J Med Res [Internet]. 2012 May [cited 2018 Dec 6];135(5):680–2. Available from: http://www.ncbi.nlm.nih.gov/pubmed/22771601

24. Kabilan L, Edwin N, Balashankar S, Meikandan D, Thenmozhi V, Gajanana A. Japanese encephalitis among paediatric patients with acute encephalitis syndrome in Tamil Nadu, India. Trans R Soc Trop Med Hyg. 2000;94(2):157–8.

25. Phukan AC, Borah PK, Mahanta J. Japanese encephalitis in Assam, northeast India. Southeast Asian J Trop Med Public Health. 2004;35(3):618–22.

26. Nyari N, Singh D, Kakkar K, Sharma S, Pandey SN, Dhole TN. Entomological and serological investigation of Japanese encephalitis in endemic area of eastern Uttar Pradesh, India. J Vector Borne Dis. 2015;52(4):321–8.

27. Kumari R, Joshi PL. A review of Japanese encephalitis in Uttar Pradesh, India. WHO South East Asia J Public Heal [Internet]. 2012;1(4):374–95. Available from: http://www.ncbi.nlm.nih.gov/pubmed/28615603

28. Kakkar M, Dhole TN, Rogawski ET, Chaturvedi S. Public health laboratory surveillance and diagnosis of Japanese encephalitis: Time to revisit. Indian Pediatr. 2016;53(1):33–5.

29. Jain P, Jain A, Kumar A, Prakash S, Khan DN, Singh KP, et al. Epidemiology and etiology of acute encephalitis syndrome in North India. Jpn J Infect Dis. 2014;67(3):197–203.

30. Jain P, Singh AK, Khan DN, Pandey M, Kumar R, Garg R, et al. Trend of Japanese encephalitis in Uttar Pradesh, India from 2011 to 2013. Epidemiol Infect. 2016;144(2):363–70.

31. Gurav YK, Bondre VP, Tandale B V, Damle RG, Mallick S, Ghosh US, et al. A large outbreak of Japanese encephalitis predominantly among adults in northern region of West Bengal, India. J Med Virol [Internet]. 2016;88(11):2004–11. Available from: http://www.ncbi.nlm.nih.gov/pubmed/27096294

32. Arai S, Matsunaga Y, Takasaki T, Tanaka-Taya K, Taniguchi K, Okabe N, et al. Japanese encephalitis: Surveillance and elimination effort in Japan from 1982 to 2004. Jpn J Infect Dis. 2008;61(5):333–8.

33. Matsunaga Y, Yabe S, Taniguchi K, Nakayama M, Kurane I. [Current status of Japanese encephalitis in Japan]. Kansenshogaku Zasshi [Internet]. 1999 Feb [cited 2018 Dec 6];73(2):97–103. Available from: http://www.ncbi.nlm.nih.gov/pubmed/10213985

34. Infectious Agent Surveillance Report. Japanese encephalitis, Japan, 2003-2008. 2009;30(6):147–8. Available from: http://idsc.nih.go.jp/iasr/30/352/tpc352.html

35. Infectious Agent Surveillance Report. Japanese encephalitis, Japan, 2007-2016. 2017;38(8):151–2.

36. Horwood PF, Duong V, Laurent D, Mey C, Sothy H, Santy K, et al. Aetiology of acute meningoencephalitis in Cambodian children, 2010-2013. Emerg Microbes Infect [Internet]. 2017;6(5):e35-8. Available from: http://dx.doi.org/10.1038/emi.2017.15

37. Lee EJ, Cha GW, Ju YR, Han MG, Lee WJ, Jeong YE. Prevalence of neutralizing antibodies to Japanese encephalitis virus among high-risk age groups in South Korea, 2010. PLoS One. 2016;11(1):1–13.

38. Sunwoo JS, Jung KH, Lee ST, Lee SK, Chu K. Reemergence of Japanese encephalitis in South Korea, 2010–2015. Emerg Infect Dis. 2016;22(10):1841–3.

39. Hsu LC, Chen YJ, Hsu FK, Huang JH, Chang CM, Chou P, et al. The incidence of Japanese encephalitis in Taiwan--a population-based study. PLoS Negl Trop Dis [Internet]. 2014;8(7):e3030. Available from: http://www.ncbi.nlm.nih.gov/pubmed/25058573

40. Chang YK, Chang HL, Wu HS, Chen KT. Epidemiological features of Japanese encephalitis in Taiwan from 2000 to 2014. Am J Trop Med Hyg. 2017;96(2):382–8.

41. Wu YC, Huang YS, Chien LJ, Lin TL, Yueh YY, Tseng WL, et al. The epidemiology of Japanese encephalitis on Taiwan during 1966-1997. Am J Trop Med Hyg. 1999;61(1):78–84.

42. Taiwan CDC. Taiwan National Infectious Disease Statistics System - Japanese encephalitis [Internet]. Available from: https://nidss.cdc.gov.tw/en/SingleDisease.aspx?dc=1&dt=3&disease=0620

43. Ho Dang Trung N, Le Thi Phuong T, Wolbers M, Nguyen van Minh H, Nguyen Thanh V, Van MP, et al. Aetiologies of central nervous system infection in Viet Nam: A prospective provincial hospital-based descriptive surveillance study. PLoS One. 2012;7(5).

44. Yen NT, Duffy MR, Hong NM, Hien NT, Fischer M, Hills SL. Surveillance for Japanese encephalitis in Vietnam, 1998-2007. Am J Trop Med Hyg. 2010;83(4):816–9.
