# Supplementary material for: Estimates of the global burden of Japanese encephalitis and the impact of vaccination from 2000-2015
Source: eLife. 2020 May 26;9:e51027. doi: 10.7554/eLife.51027 (PMC7282807; doi:10.7554/eLife.51027)

Fig4- Supp1 - 1. Model fit of all age-stratified case data in a study of 4 divisions in Bangladesh. For each study, the red dots with red vertical lines are the mean cases by age group estimated from the model with 95% credible interval. The blue bars are the cases reported by each age group.

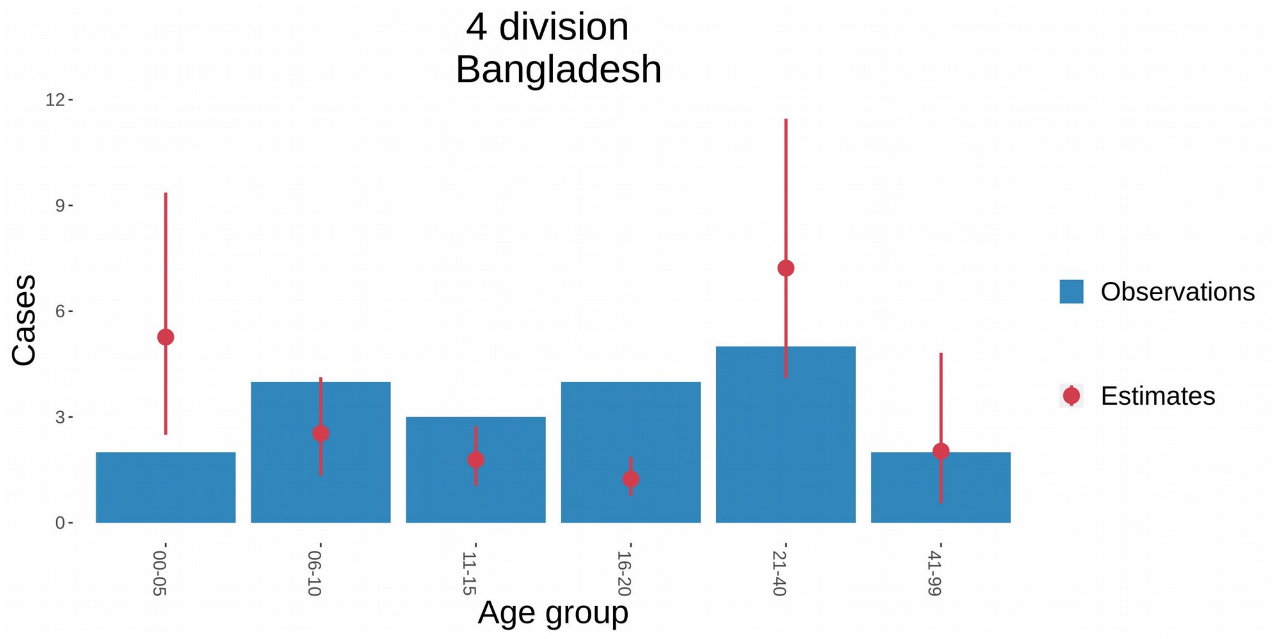

Fig4- Supp1 - 2. Model fit of all age-stratified case data in a study of 5 northern provinces in Vietnam. For each study, the red dots with red vertical lines are the mean cases by age group estimated from the model with 95% credible interval. The blue bars are the cases reported by each age group.

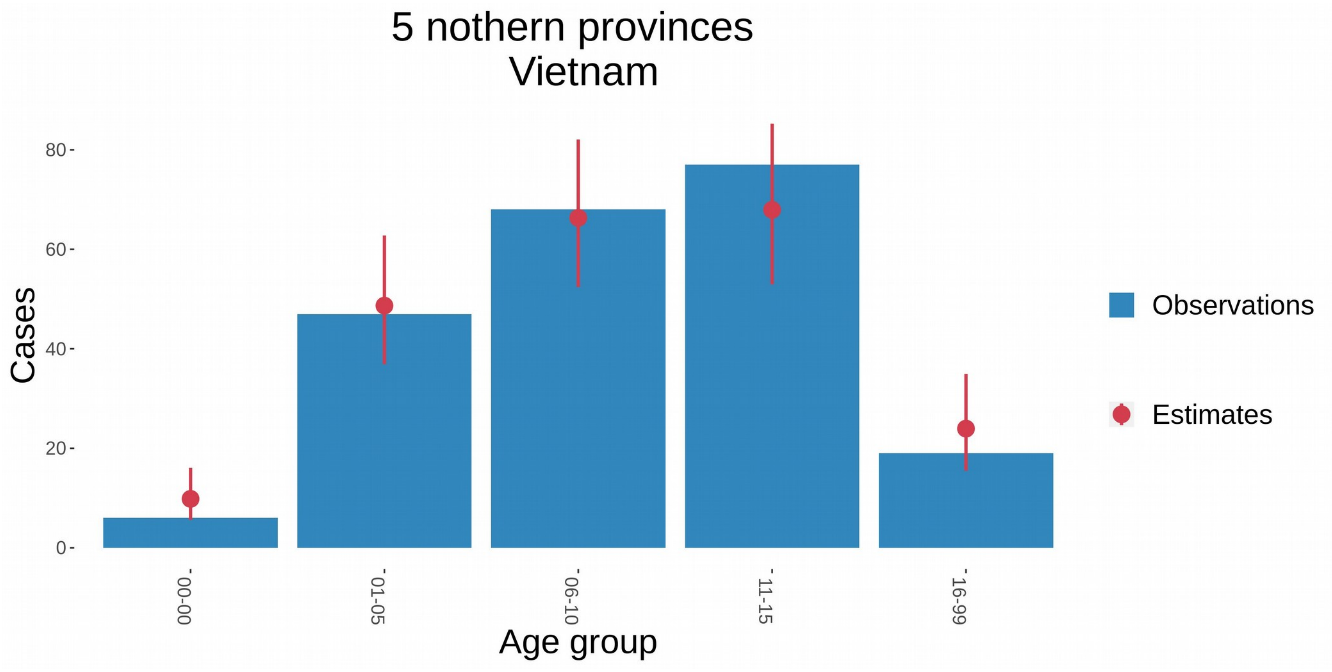

Fig4- Supp1 - 3. Model fit of all age-stratified case data in a study of 6 provinces in Indonesia. For each study, the red dots with red vertical lines are the mean cases by age group estimated from the model with 95% credible interval. The blue bars are the cases reported by each age group.

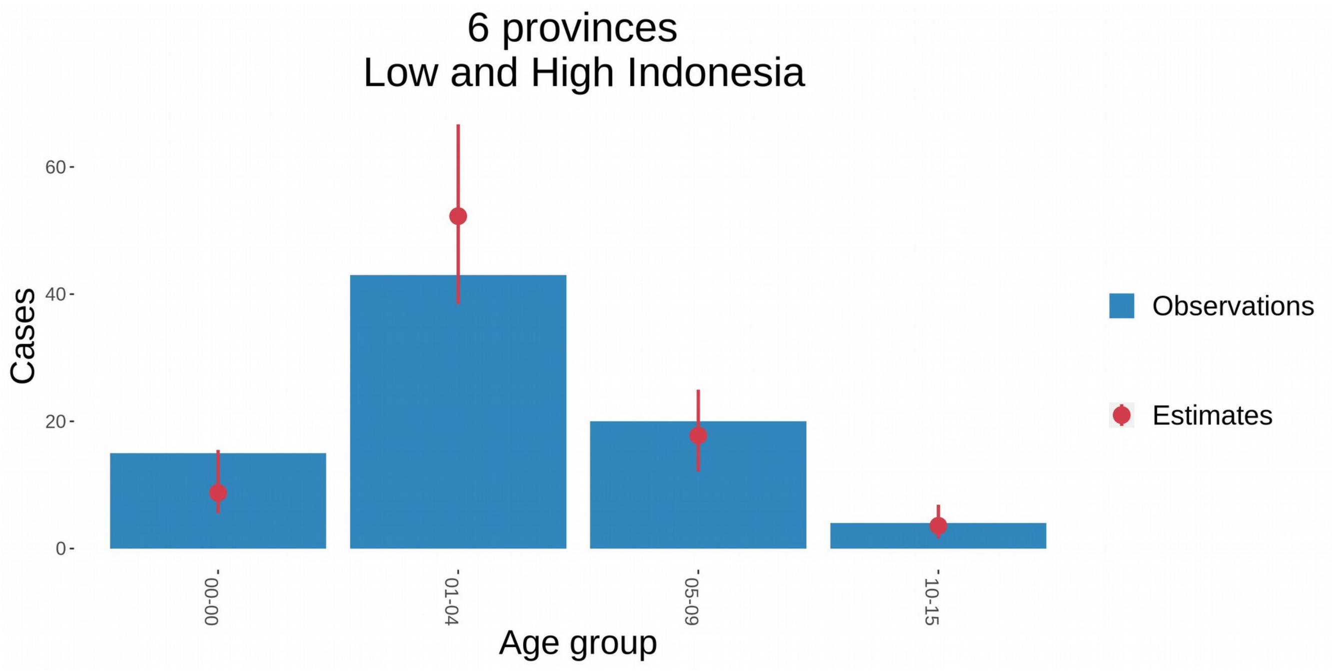

Fig4- Supp1 - 4. Model fit of all age-stratified case data in a study of 13 southern provinces in Vietnam. For each study, the red dots with red vertical lines are the mean cases by age group estimated from the model with 95% credible interval. The blue bars are the cases reported by each age group.

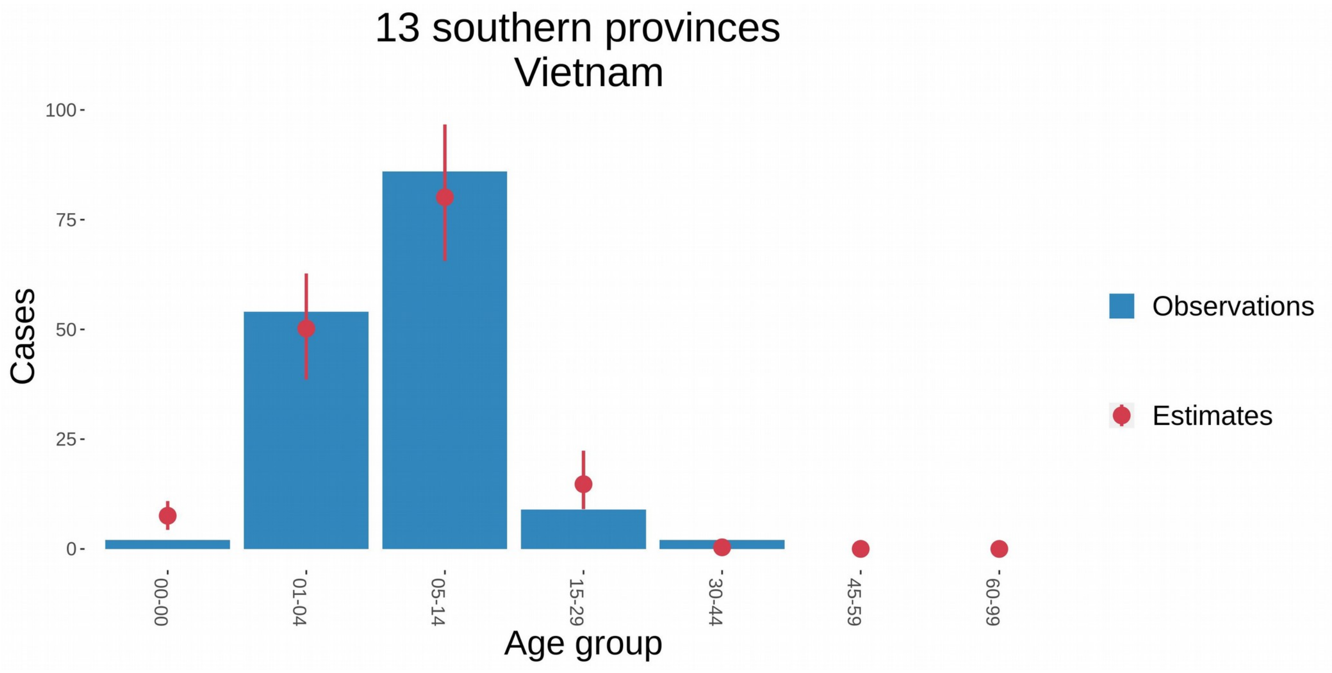

Fig4- Supp1 - 5. Model fit of all age-stratified case data in a study of Assam in medium incidence region in India. For each study, the red dots with red vertical lines are the mean cases by age group estimated from the model with 95% credible interval. The blue bars are the cases reported by each age group.

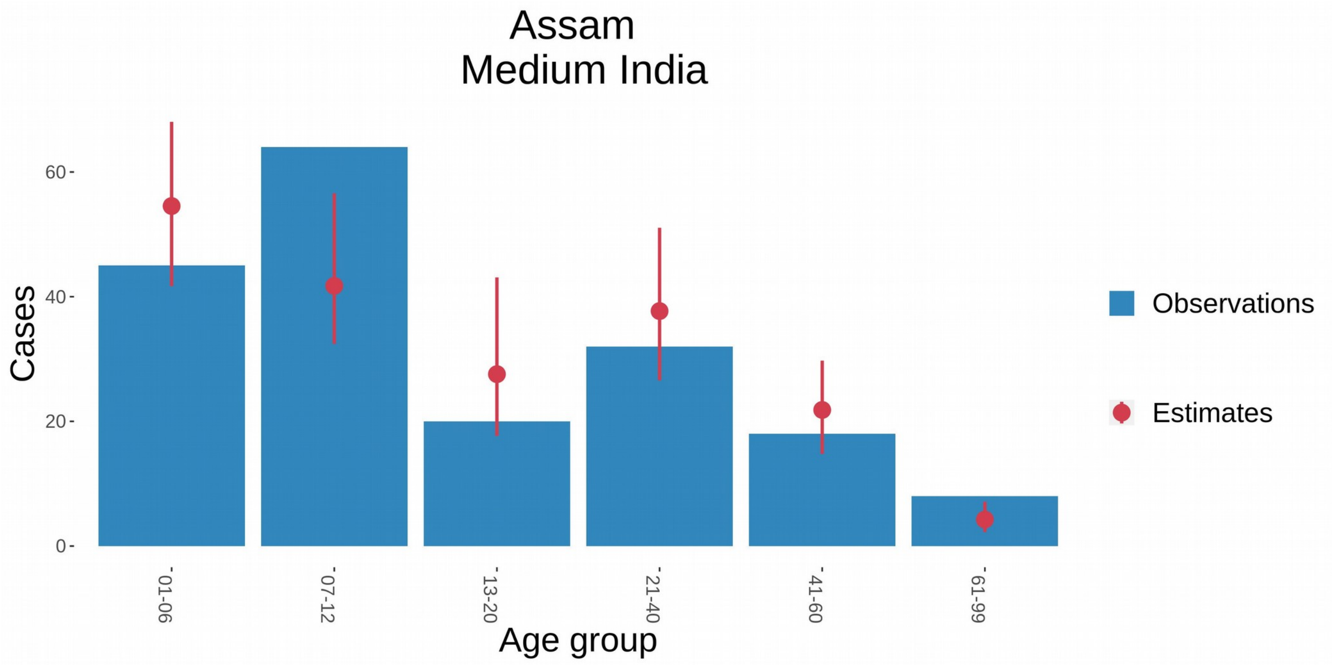

Fig4- Supp1 - 6. Model fit of all age-stratified case data in a study of Bali in Indonesia. For each study, the red dots with red vertical lines are the mean cases by age group estimated from the model with 95% credible interval. The blue bars are the cases reported by each age group.

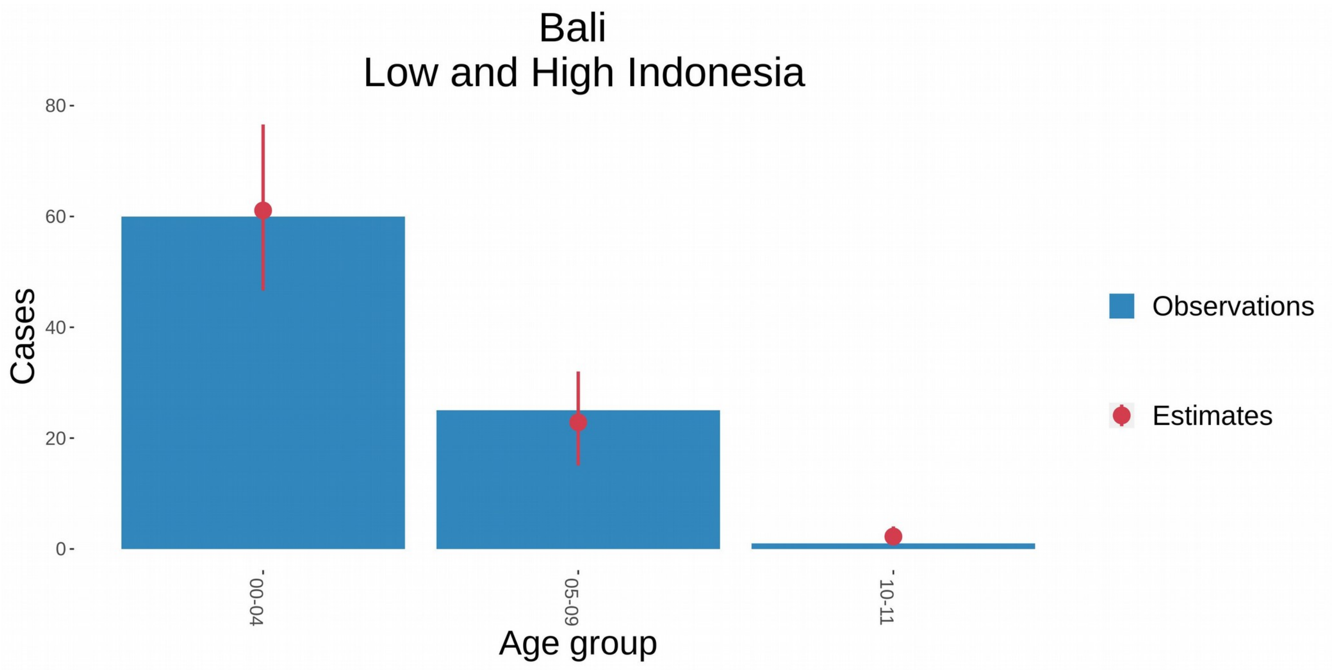

Fig4- Supp1 - 7. Model fit of all age-stratified case data in a study of Bangkok and Hat Yai in Thailand. For each study, the red dots with red vertical lines are the mean cases by age group estimated from the model with 95% credible interval. The blue bars are the cases reported by each age group.

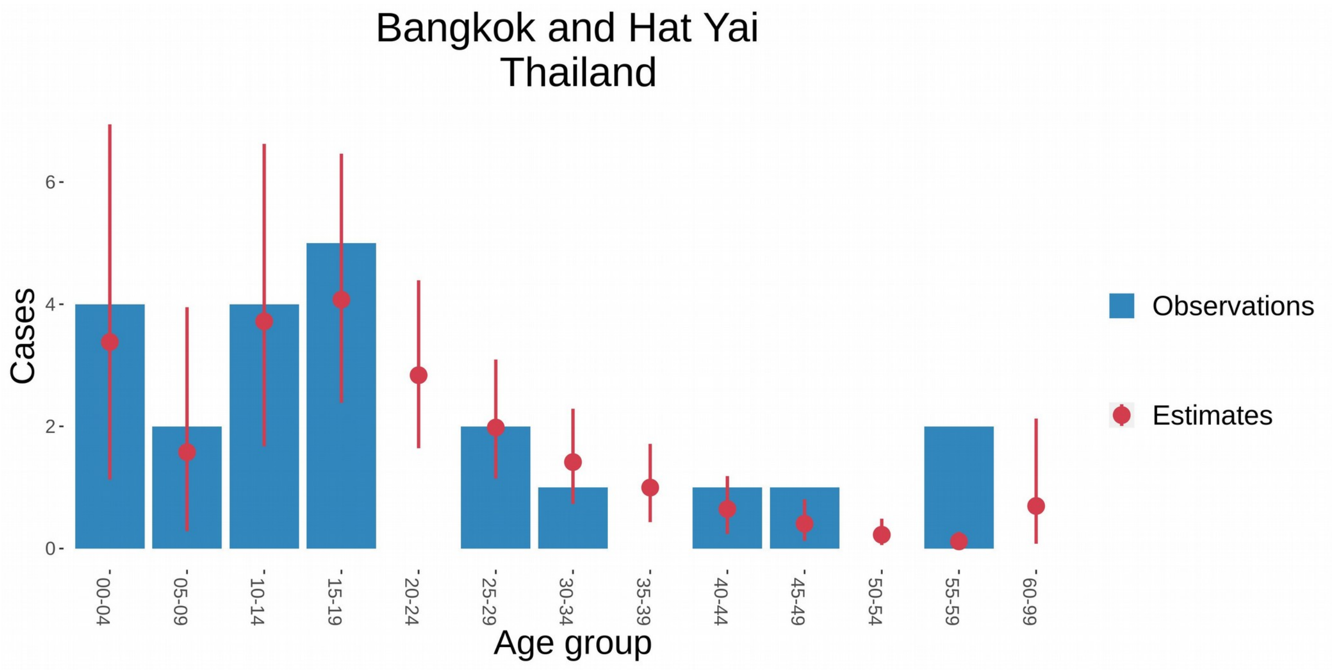

Fig4- Supp1 - 8. Model fit of all age-stratified case data in a study of Baoji in high incidence region in China. For each study, the red dots with red vertical lines are the mean cases by age group estimated from the model with 95% credible interval. The blue bars are the cases reported by each age group.

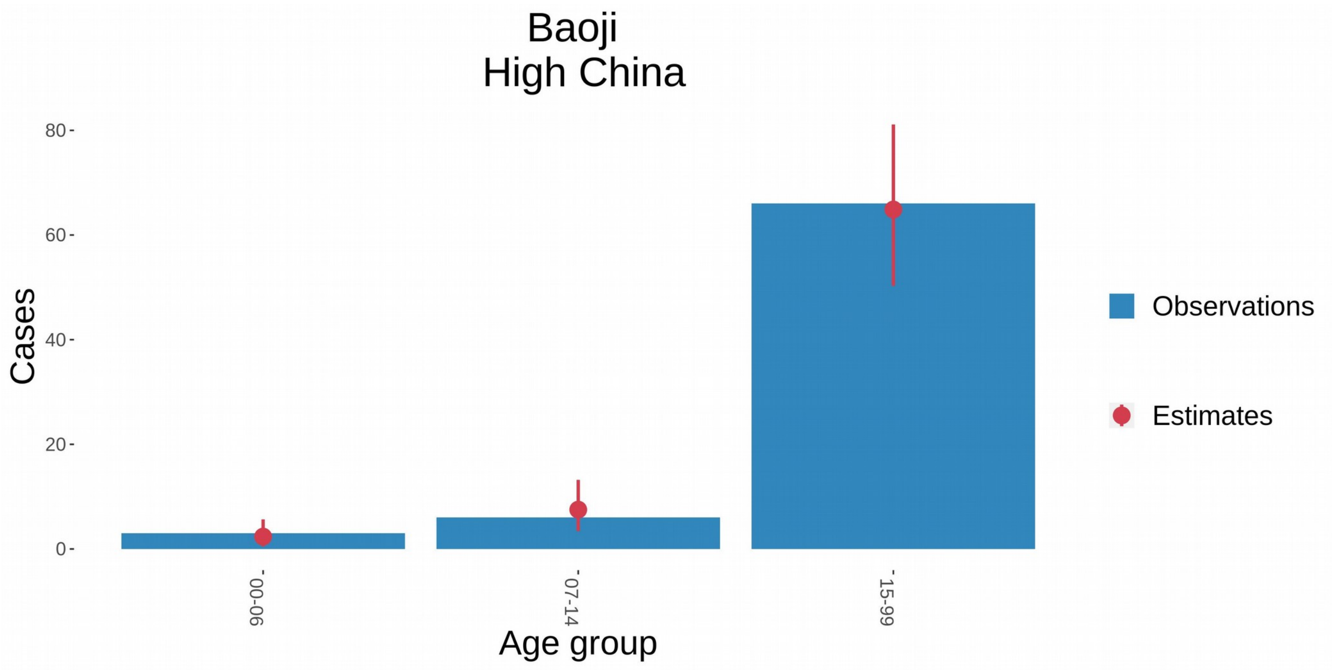

Fig4- Supp1 - 9. Model fit of all age-stratified case data in a study of Bellary and neighbors in India. For each study, the red dots with red vertical lines are the mean cases by age group estimated from the model with 95% credible interval. The blue bars are the cases reported by each age group.

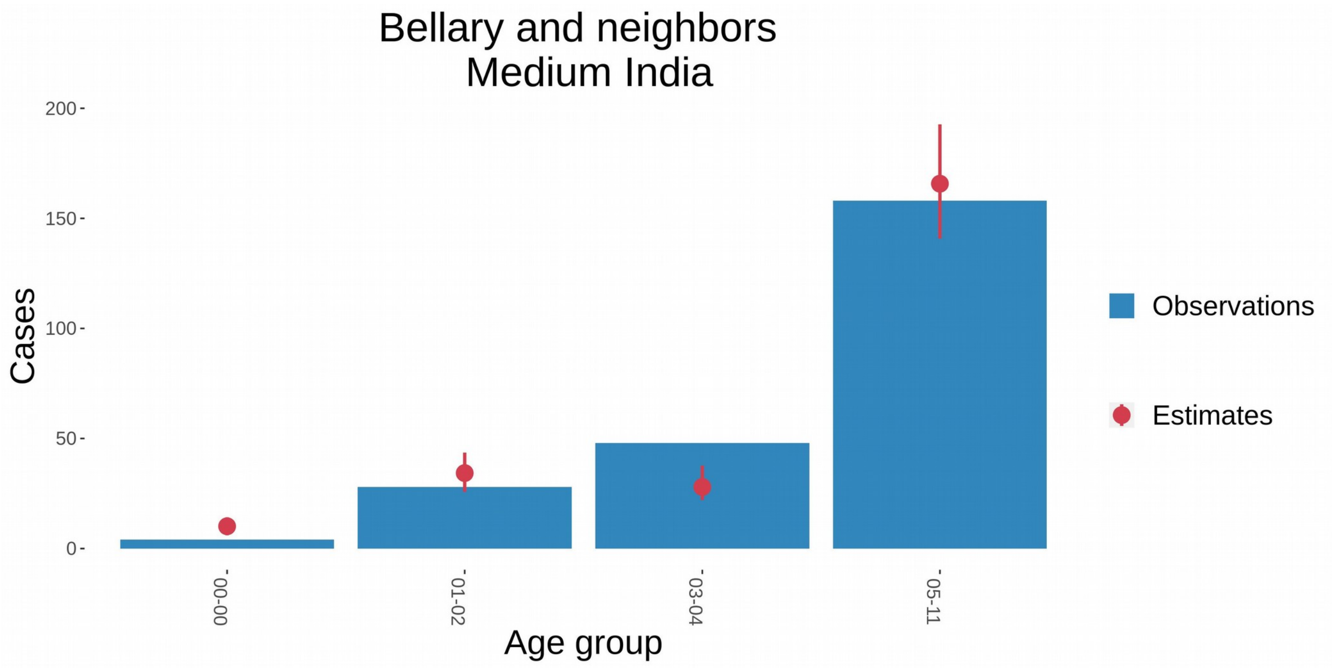

Fig4- Supp1 - 10 Model fit of all age-stratified case data in a study of Bellary in India. For each study, the red dots with red vertical lines are the mean cases by age group estimated from the model with 95% credible interval. The blue bars are the cases reported by each age group.

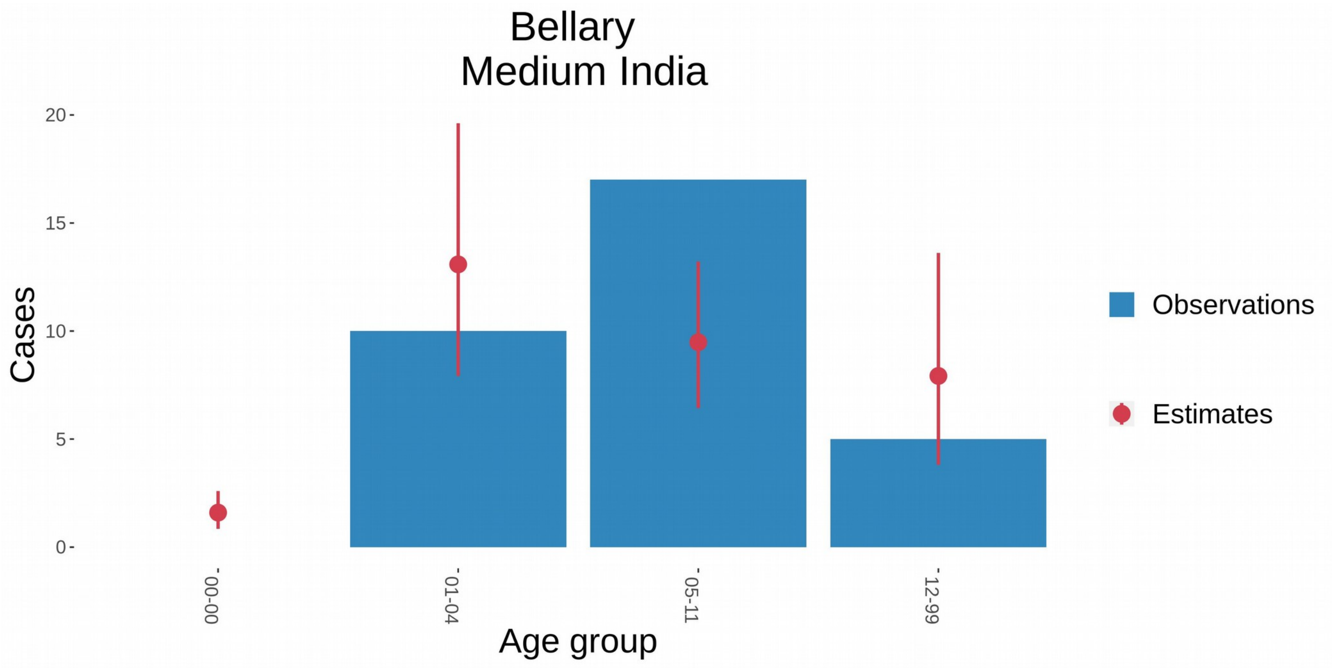

Fig4- Supp1 – 11. Model fit of all age-stratified case data in a study of central Taiwan. For each study, the red dots with red vertical lines are the mean cases by age group estimated from the model with 95% credible interval. The blue bars are the cases reported by each age group.

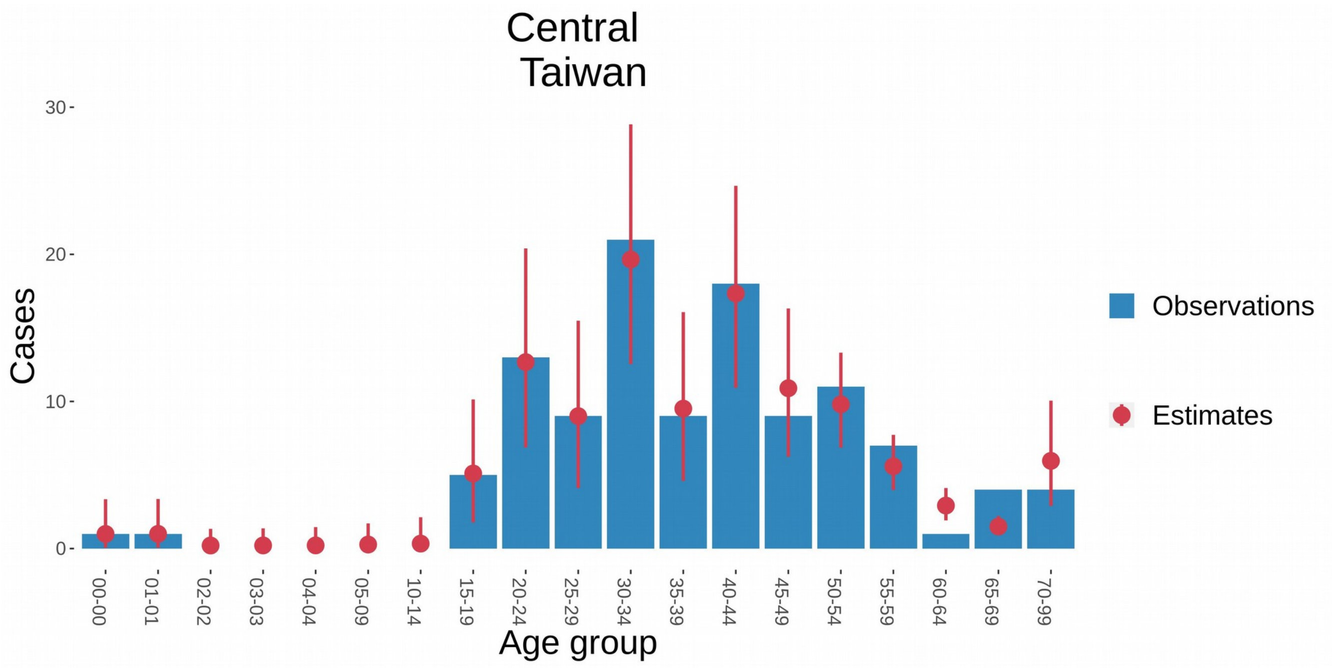

Fig4- Supp1 – 12. Model fit of all age-stratified case data in a study of Cuddalore in medium incidence region in India. For each study, the red dots with red vertical lines are the mean cases by age group estimated from the model with 95% credible interval. The blue bars are the cases reported by each age group.

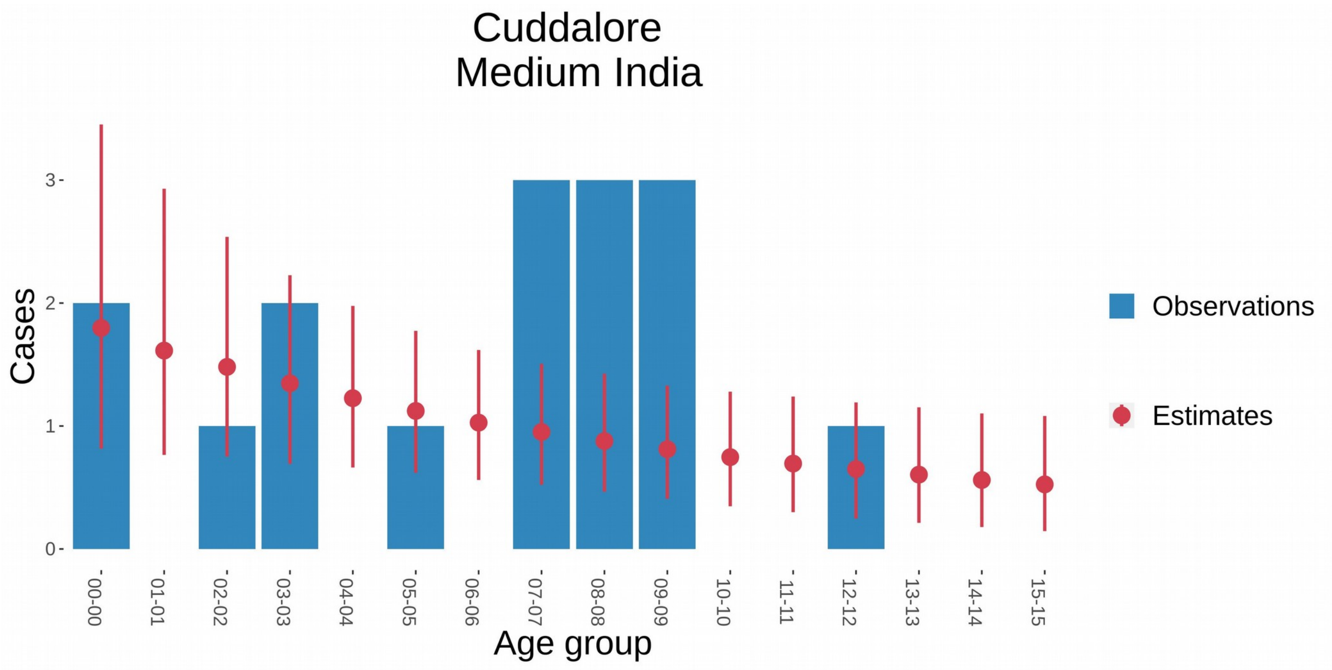

Fig4- Supp1 – 13. Model fit of all age-stratified case data in a study of Dhemaji in medium incidence region in India. For each study, the red dots with red vertical lines are the mean cases by age group estimated from the model with 95% credible interval. The blue bars are the cases reported by each age group.

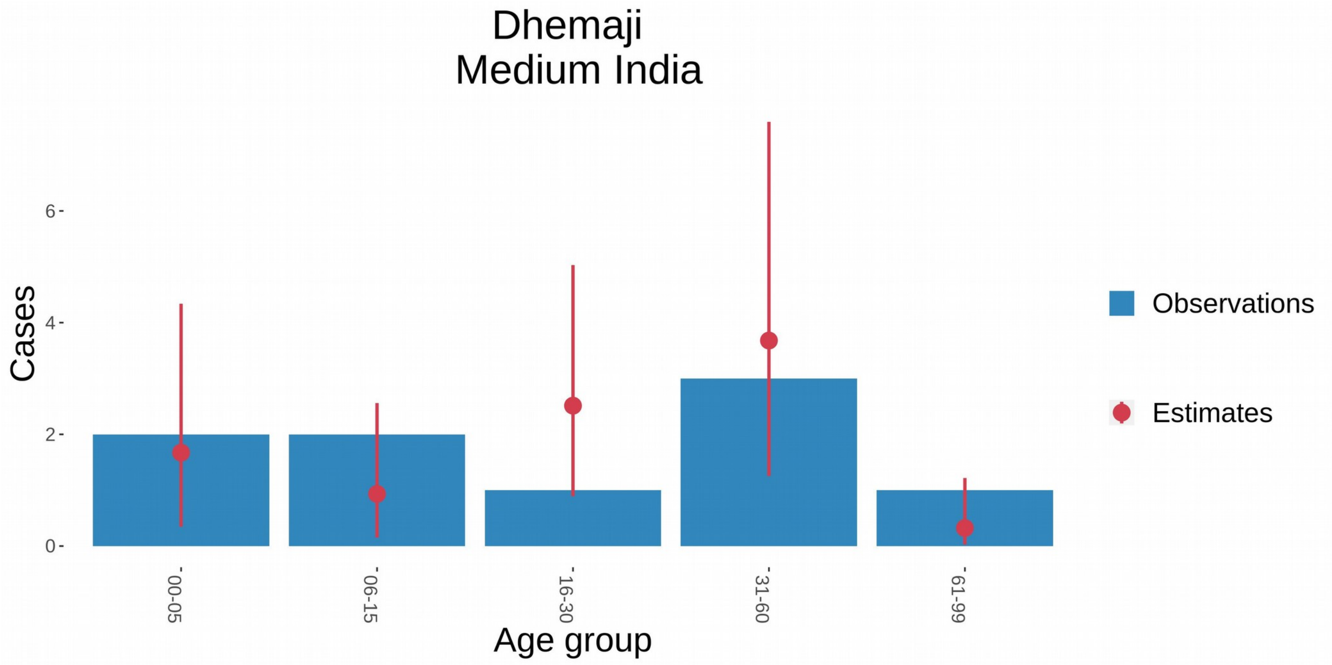

Fig4- Supp1 – 14. Model fit of all age-stratified case data in a study of eastern Taiwan. For each study, the red dots with red vertical lines are the mean cases by age group estimated from the model with 95% credible interval. The blue bars are the cases reported by each age group.

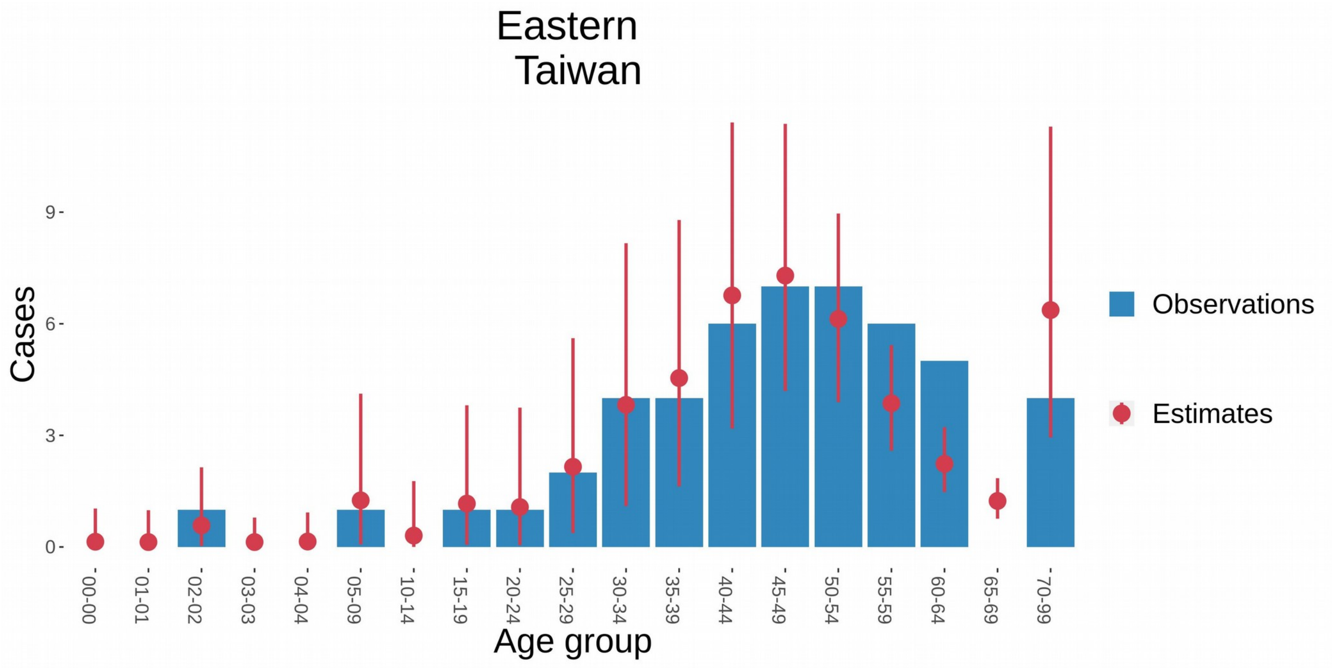

Fig4- Supp1 – 15. Model fit of all age-stratified case data in a study of endemic provinces in China. For each study, the red dots with red vertical lines are the mean cases by age group estimated from the model with 95% credible interval. The blue bars are the cases reported by each age group.

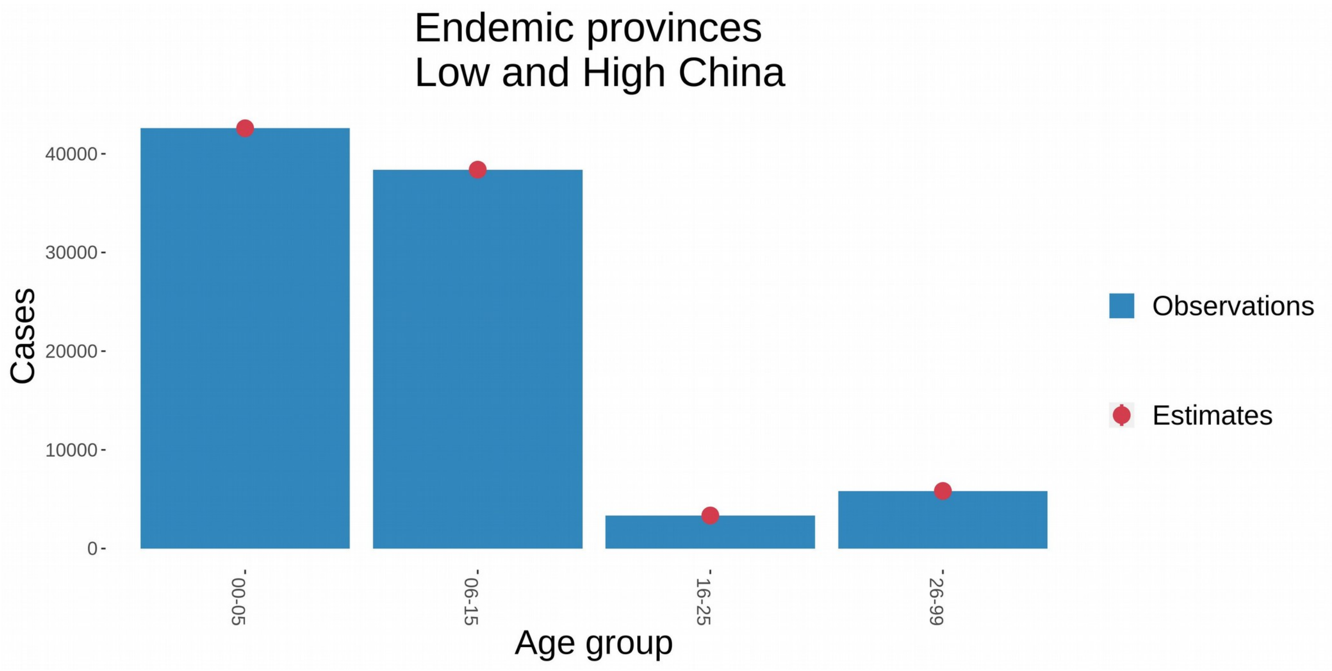

Fig4- Supp1 – 16. Model fit of all age-stratified case data in a study of Gorakhupur in high incidence region in India. For each study, the red dots with red vertical lines are the mean cases by age group estimated from the model with 95% credible interval. The blue bars are the cases reported by each age group.

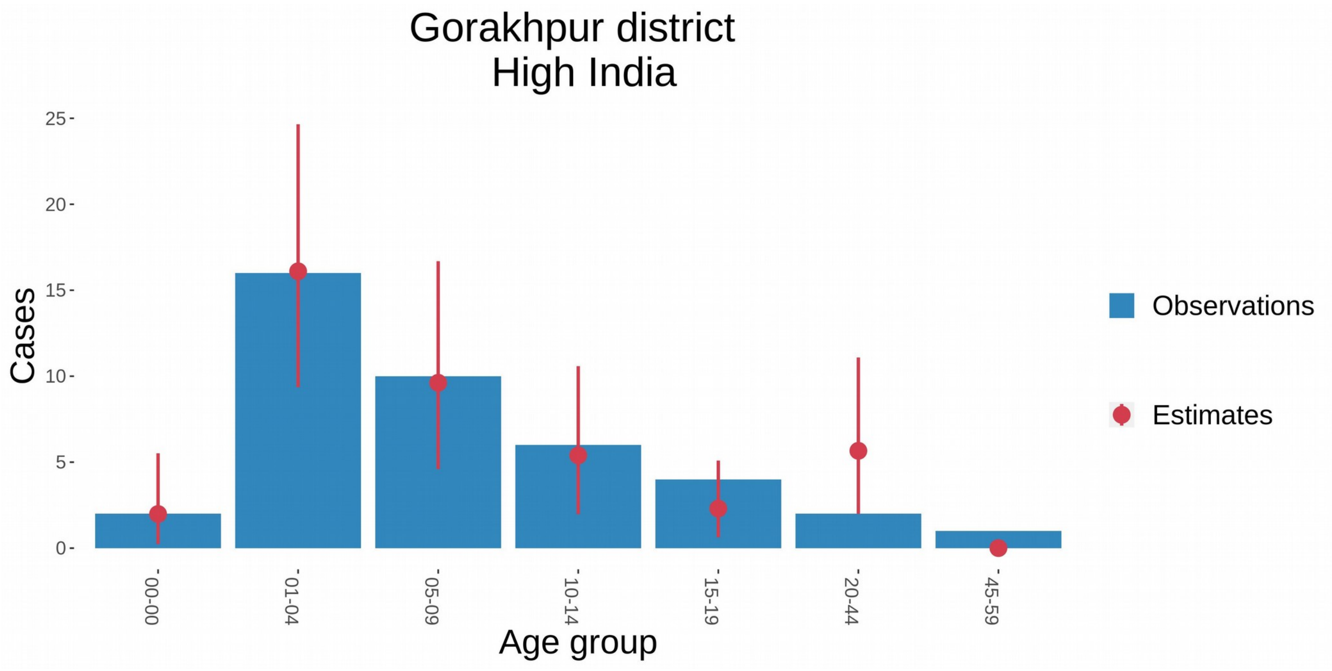

Fig4- Supp1 – 17. Model fit of all age-stratified case data in a study of Gorakhpur division in high incidence region in India. For each study, the red dots with red vertical lines are the mean cases by age group estimated from the model with 95% credible interval. The blue bars are the cases reported by each age group.

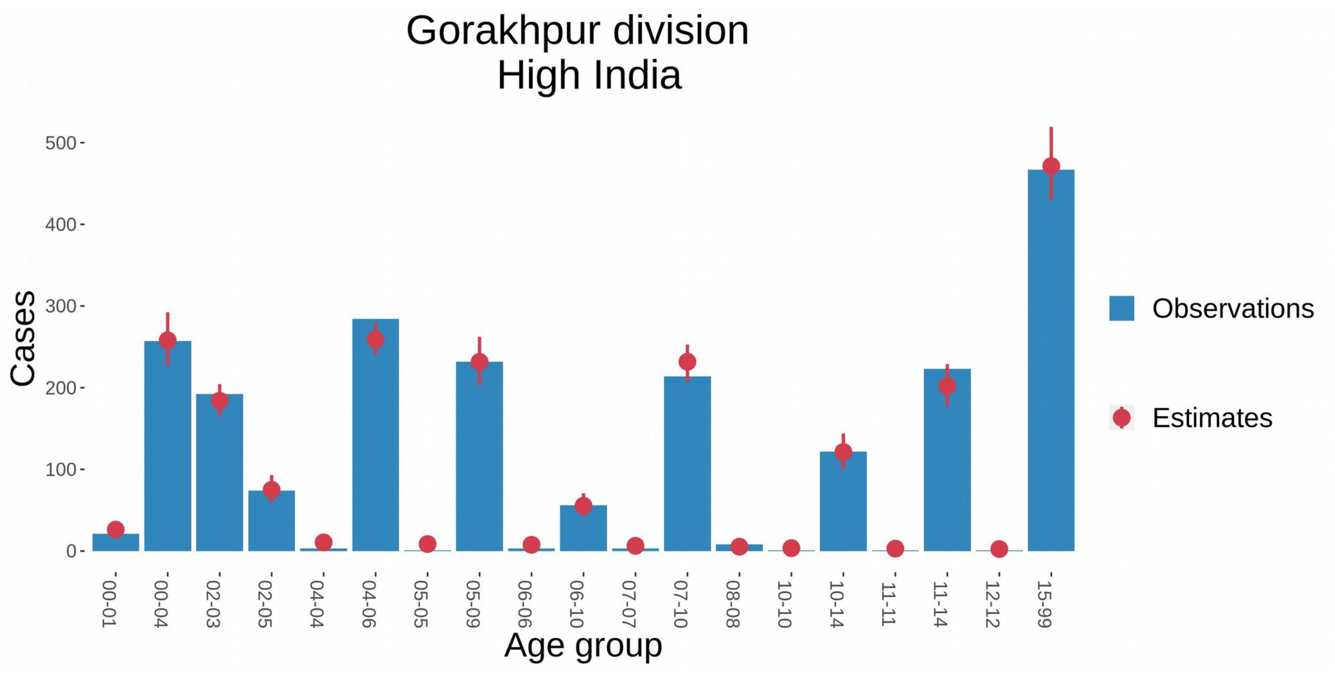

Fig4- Supp1 – 18. Model fit of all age-stratified case data in a study of Guigang in high incidence region in China. For each study, the red dots with red vertical lines are the mean cases by age group estimated from the model with 95% credible interval. The blue bars are the cases reported by each age group.

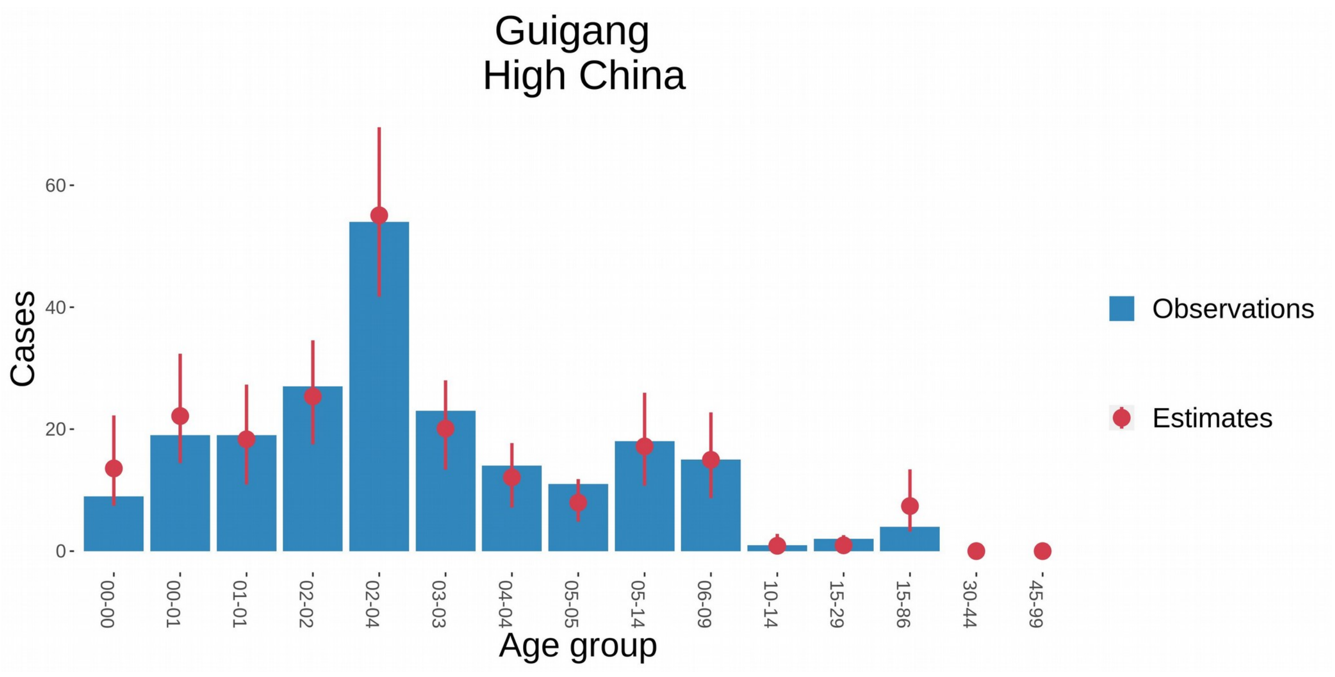

Fig4- Supp1 – 19. Model fit of all age-stratified case data in a study of Guizhou in high incidence region in China. For each study, the red dots with red vertical lines are the mean cases by age group estimated from the model with 95% credible interval. The blue bars are the cases reported by each age group.

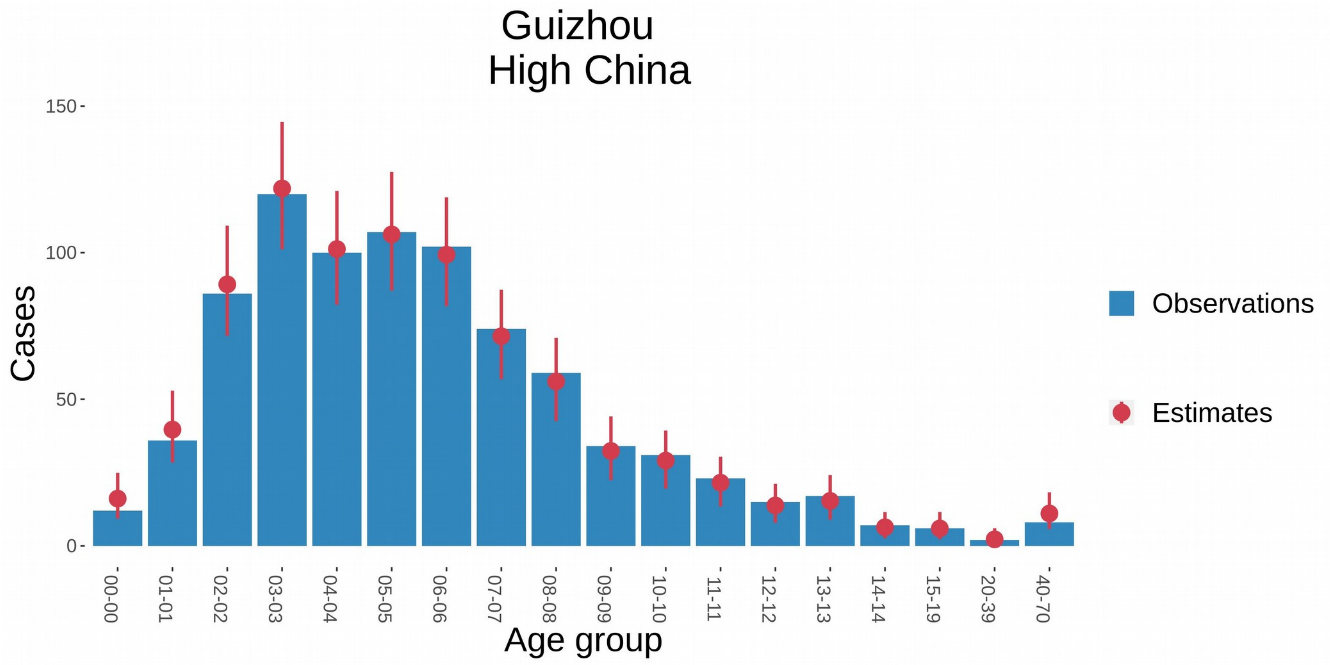

Fig4- Supp1 – 20. Model fit of all age-stratified case data in a study of hill and mountain districts in low incidence region in Nepal. For each study, the red dots with red vertical lines are the mean cases by age group estimated from the model with 95% credible interval. The blue bars are the cases reported by each age group.

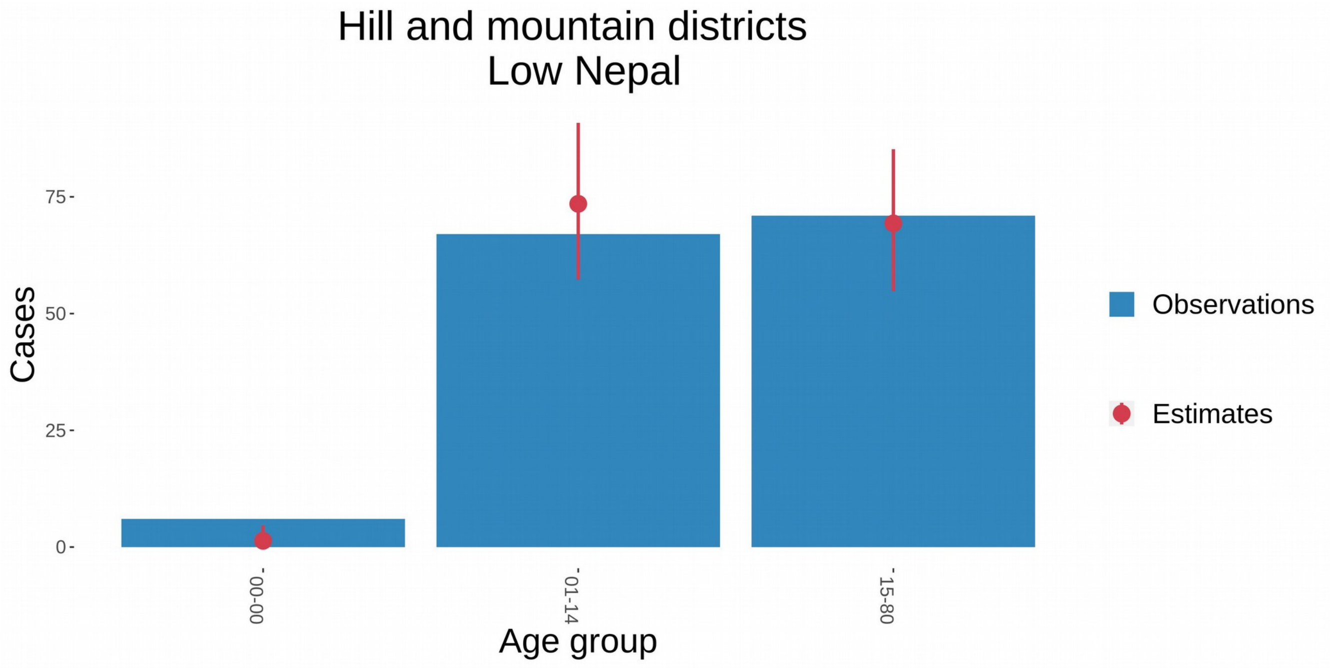

Fig4- Supp1 – 21. Model fit of all age-stratified case data in a study of Jinan in high incidence region in China. For each study, the red dots with red vertical lines are the mean cases by age group estimated from the model with 95% credible interval. The blue bars are the cases reported by each age group.

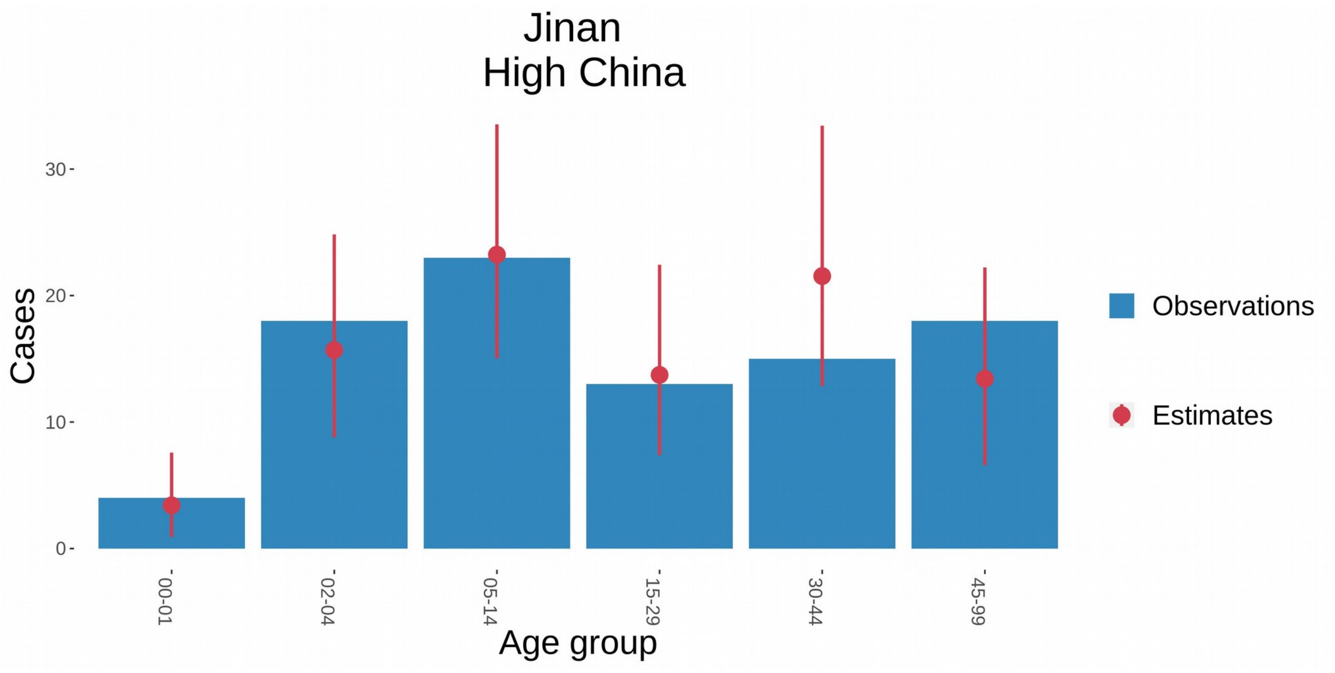

Fig4- Supp1 – 22. Model fit of all age-stratified case data in a study of Kaopping in Taiwan. For each study, the red dots with red vertical lines are the mean cases by age group estimated from the model with 95% credible interval. The blue bars are the cases reported by each age group.

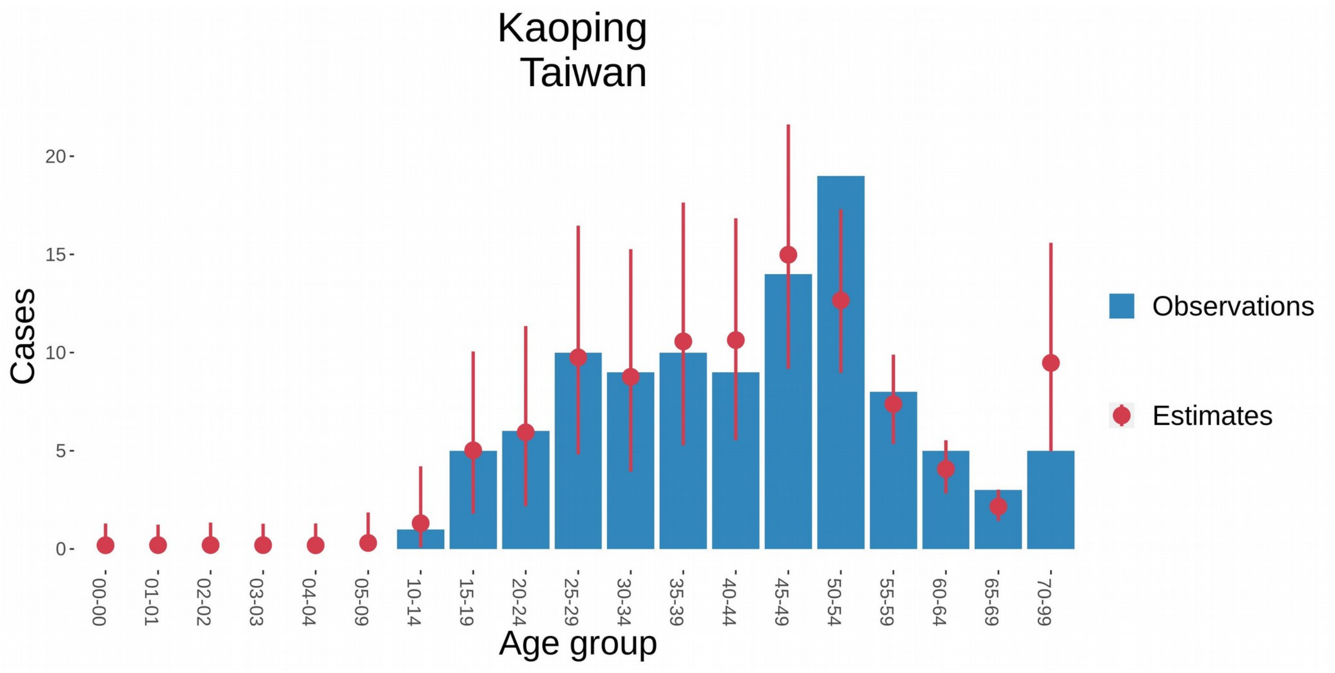

Fig4- Supp1 – 23. Model fit of all age-stratified case data in a study of Kathmandu districts in low incidence region in Nepal. For each study, the red dots with red vertical lines are the mean cases by age group estimated from the model with 95% credible interval. The blue bars are the cases reported by each age group.

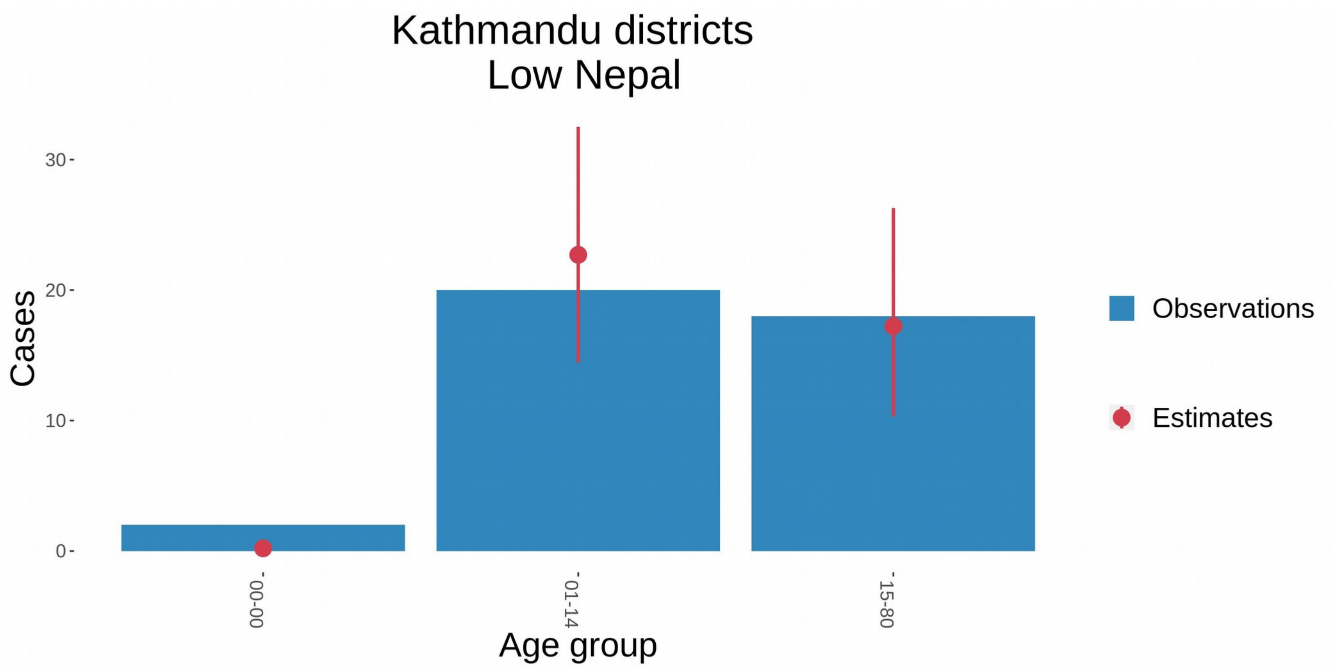

Fig4- Supp1 – 24. Model fit of all age-stratified case data in a study of Longnan in high incidence region in China. For each study, the red dots with red vertical lines are the mean cases by age group estimated from the model with 95% credible interval. The blue bars are the cases reported by each age group.

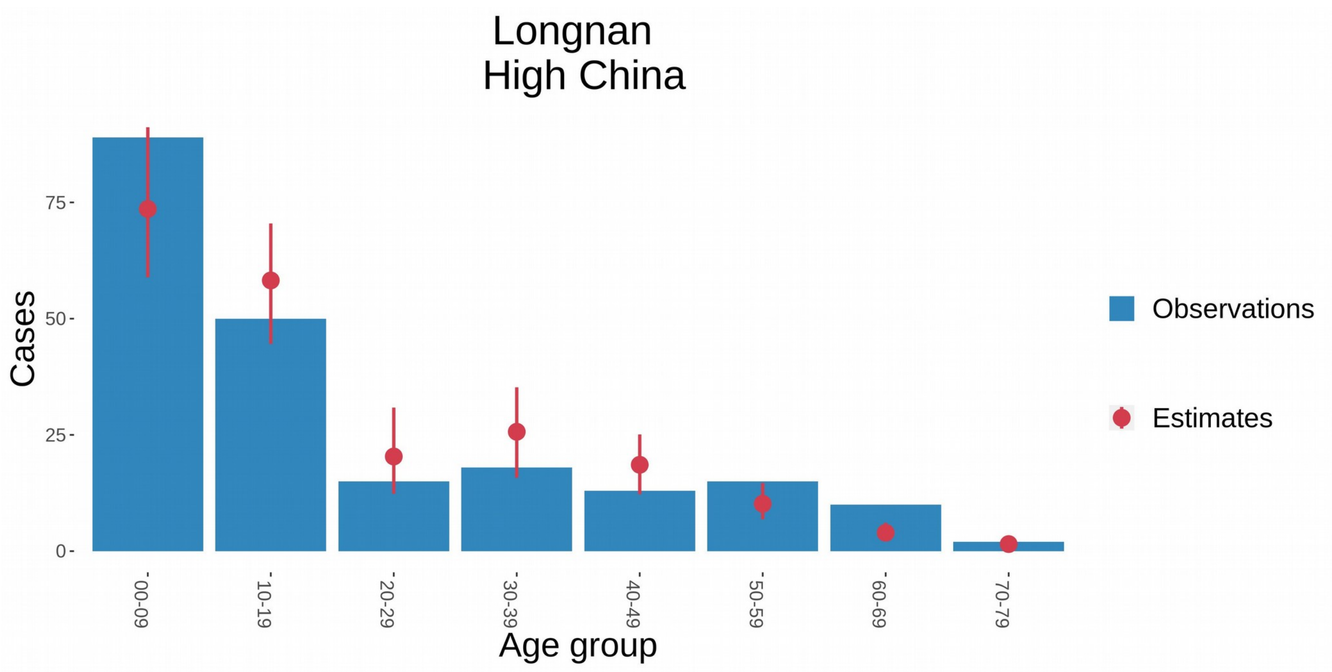

Fig4- Supp1 – 25. Model fit of all age-stratified case data in a study in Cambodia. For each study, the red dots with red vertical lines are the mean cases by age group estimated from the model with 95% credible interval. The blue bars are the cases reported by each age group.

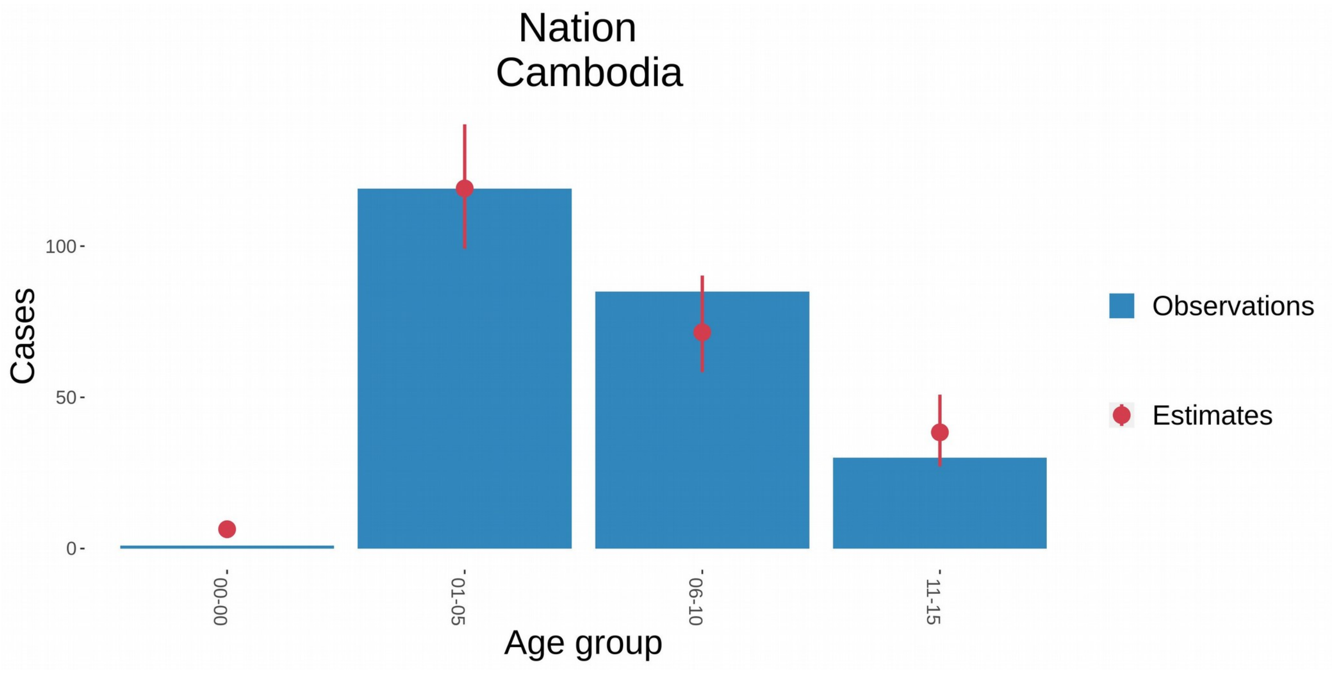

Fig4- Supp1 – 26. Model fit of all age-stratified case data in a study in India. For each study, the red dots with red vertical lines are the mean cases by age group estimated from the model with 95% credible interval. The blue bars are the cases reported by each age group.

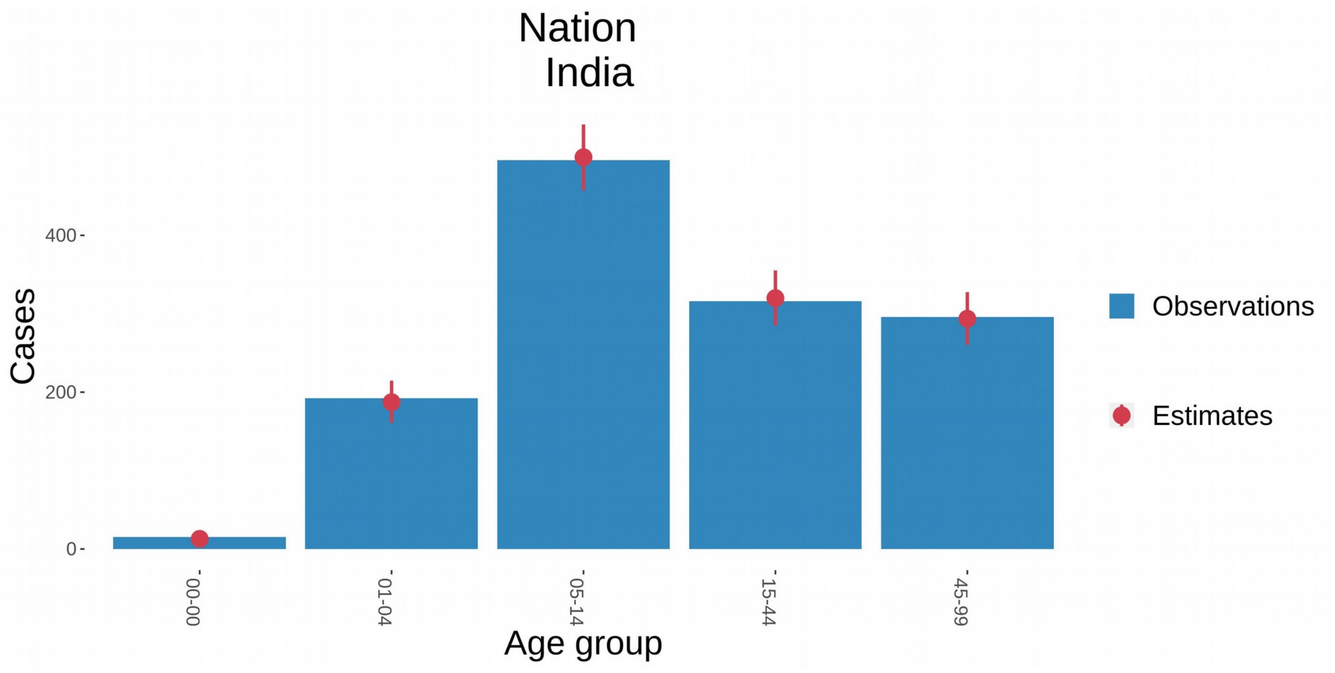

Fig4- Supp1 – 27. Model fit of all age-stratified case data in a study in Japan. For each study, the red dots with red vertical lines are the mean cases by age group estimated from the model with 95% credible interval. The blue bars are the cases reported by each age group.

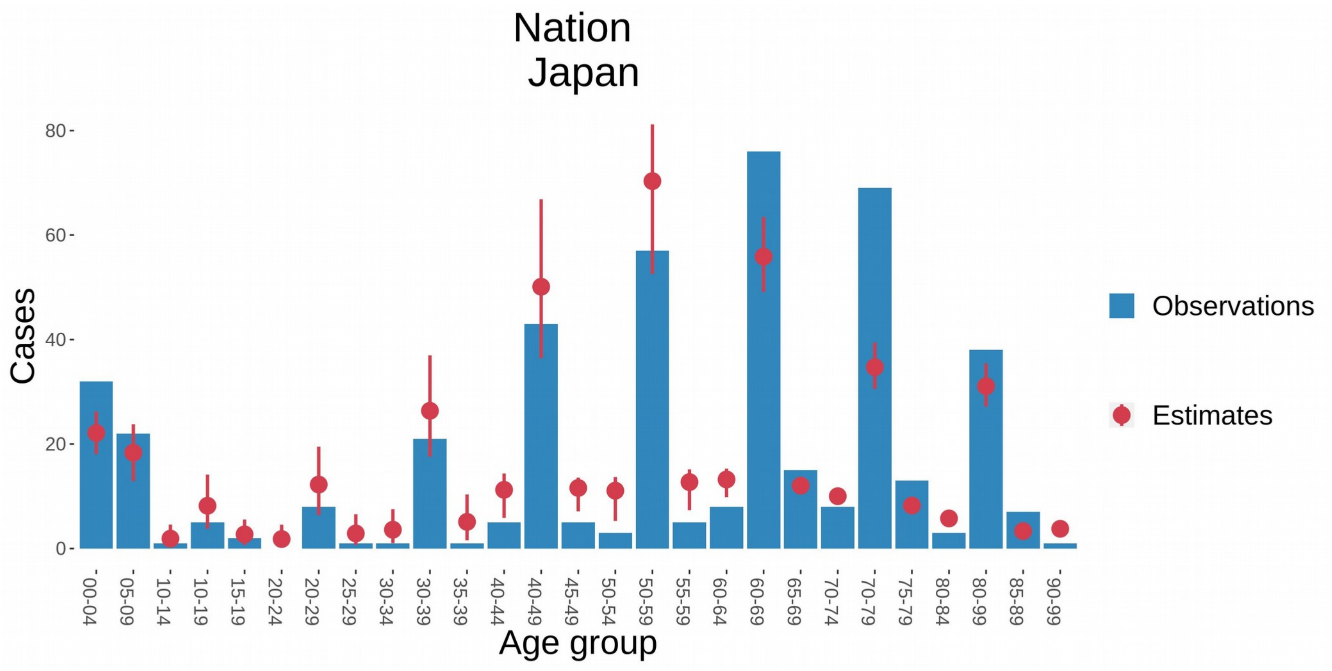

Fig4- Supp1 – 28. Model fit of all age-stratified case data in a study in Malayasia. For each study, the red dots with red vertical lines are the mean cases by age group estimated from the model with 95% credible interval. The blue bars are the cases reported by each age group.

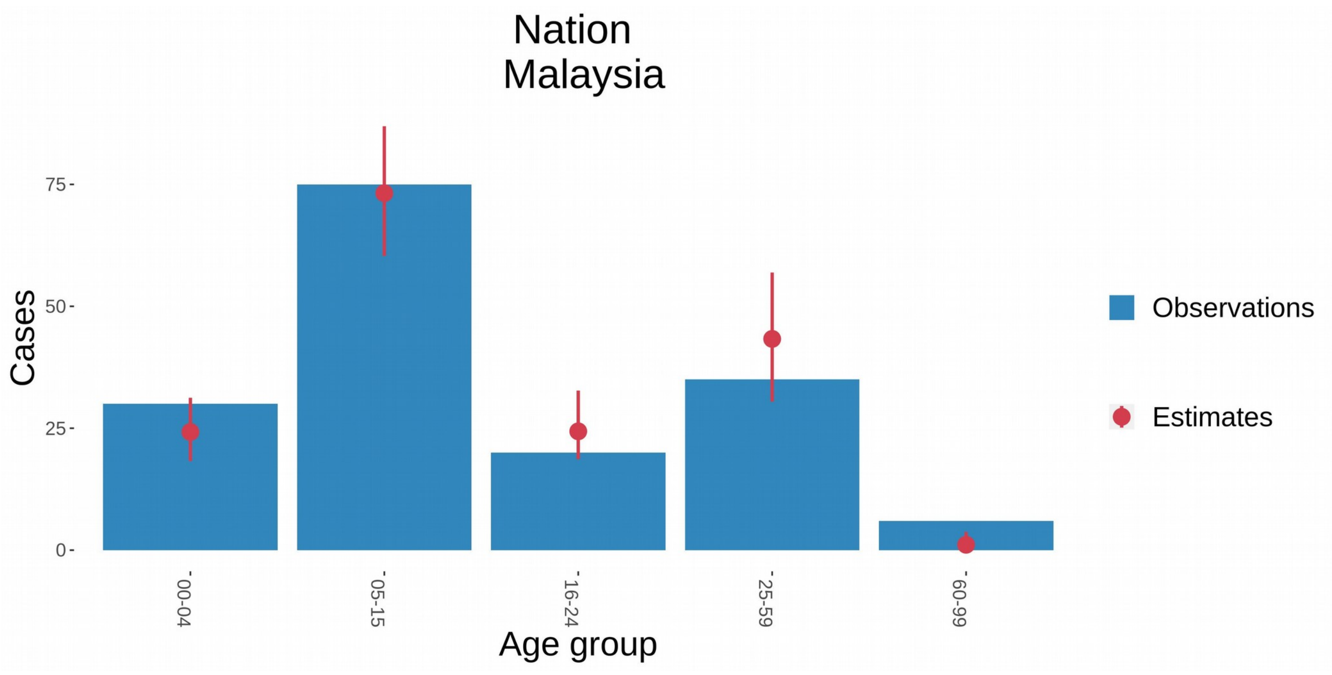

Fig4- Supp1 – 29. Model fit of all age-stratified case data in a study In Nepal. For each study, the red dots with red vertical lines are the mean cases by age group estimated from the model with 95% credible interval. The blue bars are the cases reported by each age group.

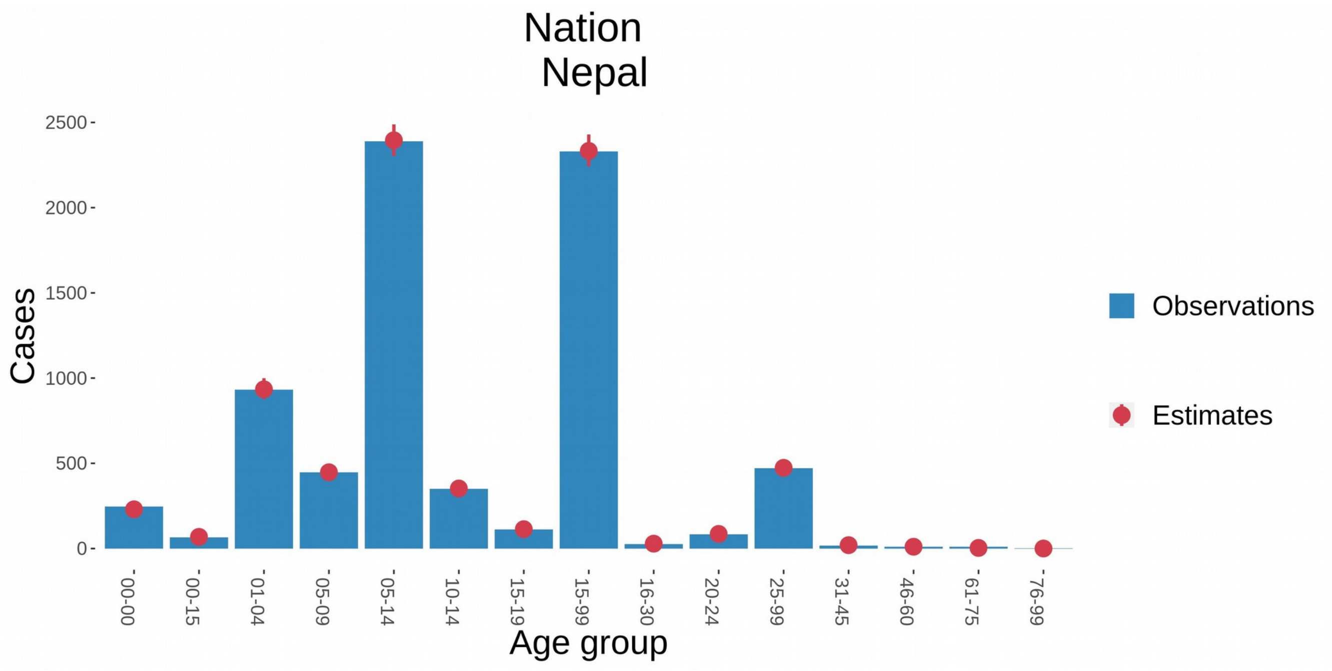

Fig4- Supp1 – 30. Model fit of all age-stratified case data in a study in Philippines. For each study, the red dots with red vertical lines are the mean cases by age group estimated from the model with 95% credible interval. The blue bars are the cases reported by each age group.

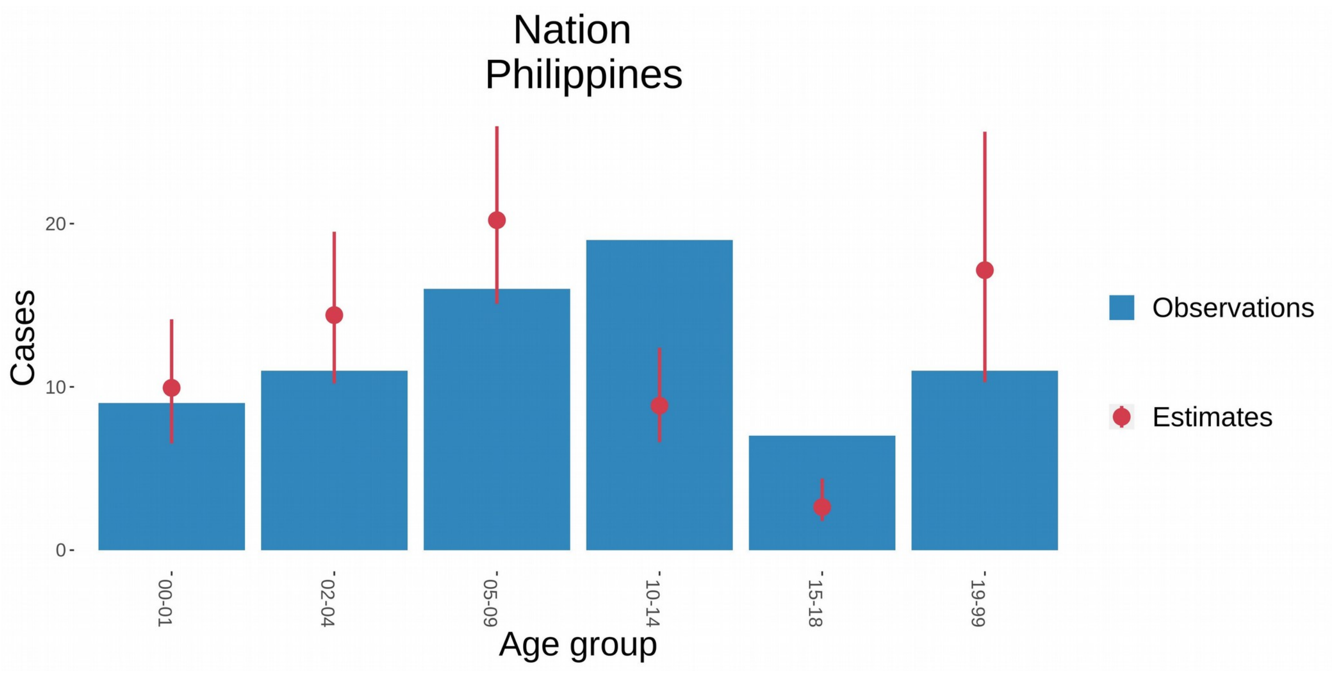

Fig4- Supp1 – 31. Model fit of all age-stratified case data in a study in South Korea. For each study, the red dots with red vertical lines are the mean cases by age group estimated from the model with 95% credible interval. The blue bars are the cases reported by each age group.

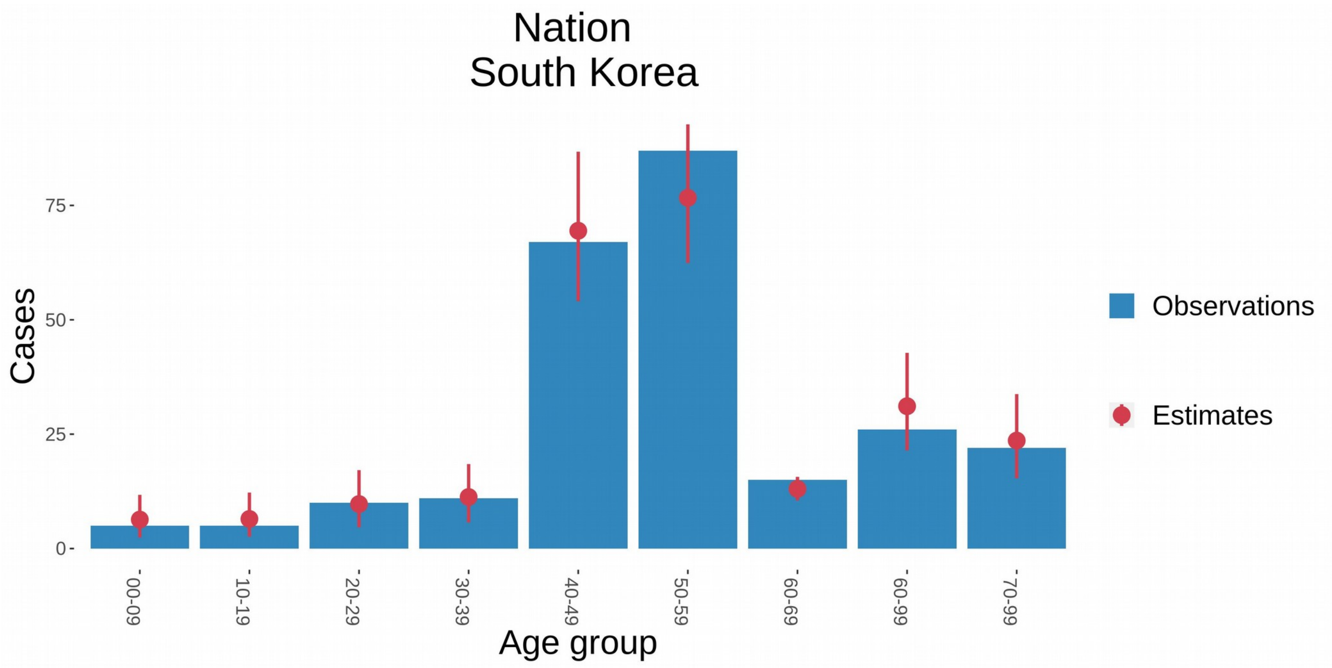

Fig4- Supp1 – 32. Model fit of all age-stratified case data in a study In Sri Lanka. For each study, the red dots with red vertical lines are the mean cases by age group estimated from the model with 95% credible interval. The blue bars are the cases reported by each age group.

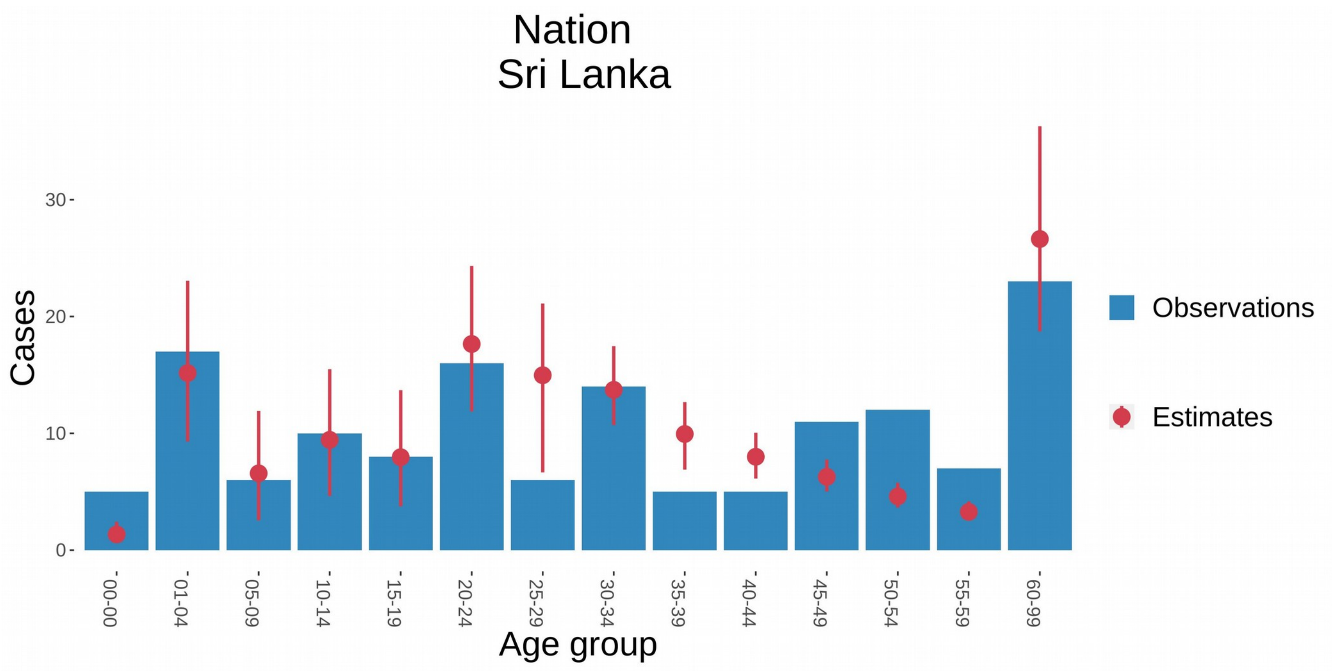

Fig4- Supp1 – 33. Model fit of all age-stratified case data in a study In Taiwan. For each study, the red dots with red vertical lines are the mean cases by age group estimated from the model with 95% credible interval. The blue bars are the cases reported by each age group.

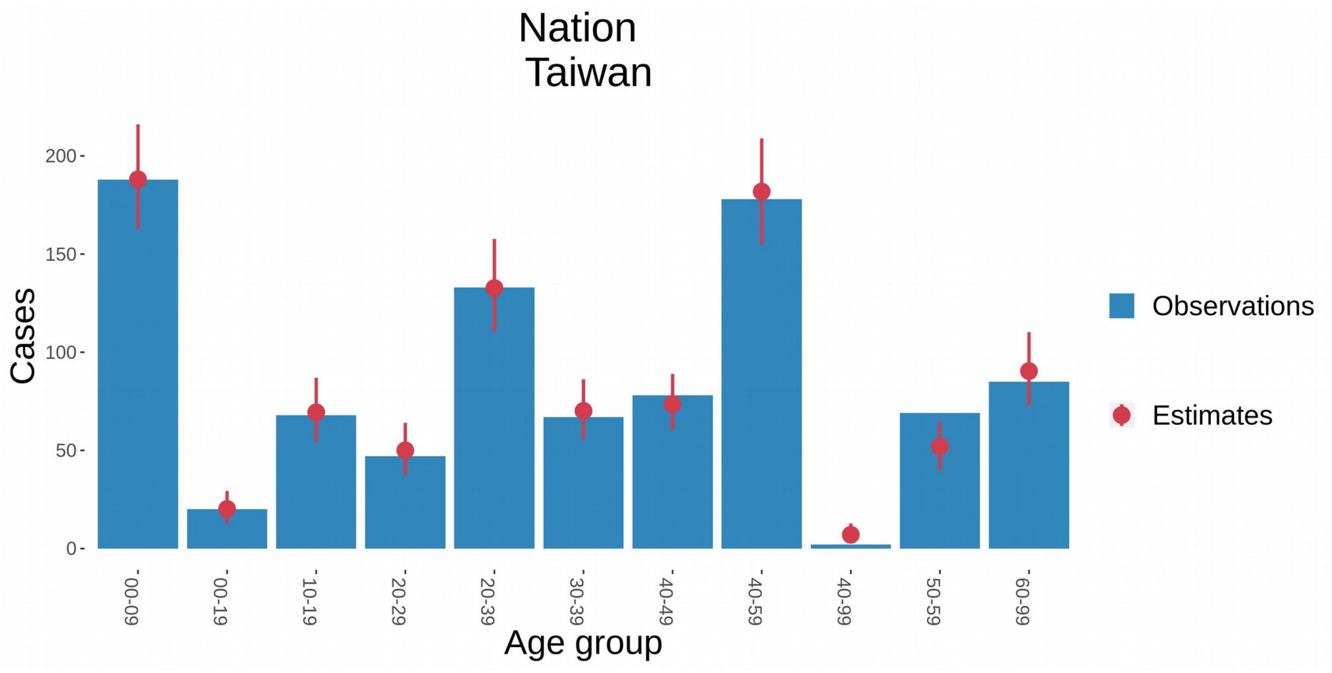

Fig4- Supp1 – 34. Model fit of all age-stratified case data in a study of Northern Taiwan. For each study, the red dots with red vertical lines are the mean cases by age group estimated from the model with 95% credible interval. The blue bars are the cases reported by each age group.

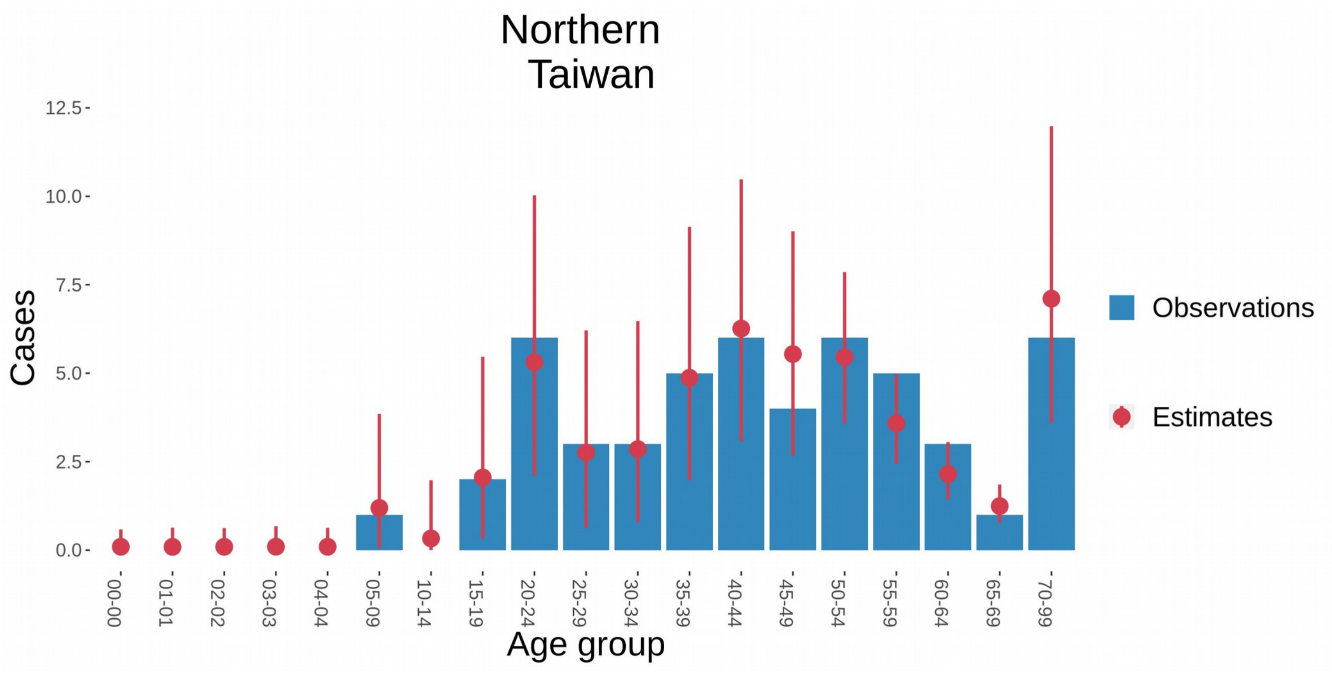

Fig4- Supp1 – 35. Model fit of all age-stratified case data in a study of Northern of Uttar Pradesh in high incidence region in India. For each study, the red dots with red vertical lines are the mean cases by age group estimated from the model with 95% credible interval. The blue bars are the cases reported by each age group.

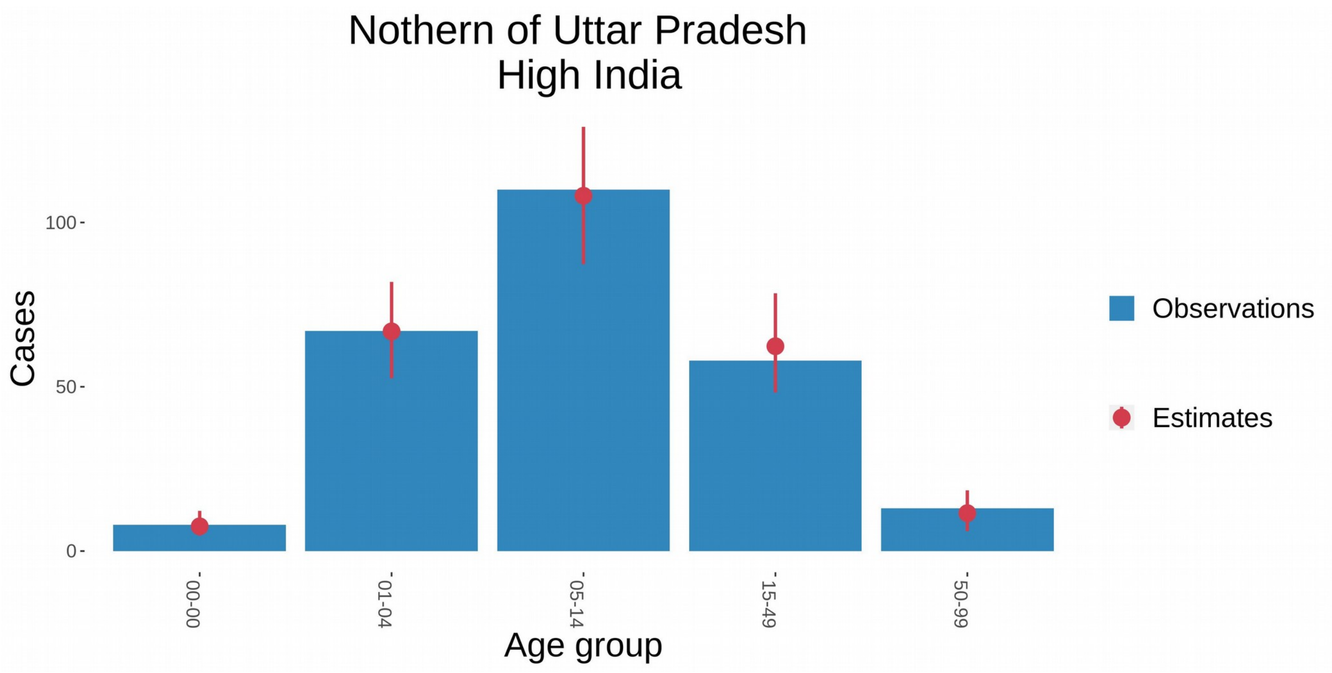

Fig4- Supp1 – 36. Model fit of all age-stratified case data in a study of not western Terai region in low incidence region in Nepal. For each study, the red dots with red vertical lines are the mean cases by age group estimated from the model with 95% credible interval. The blue bars are the cases reported by each age group.

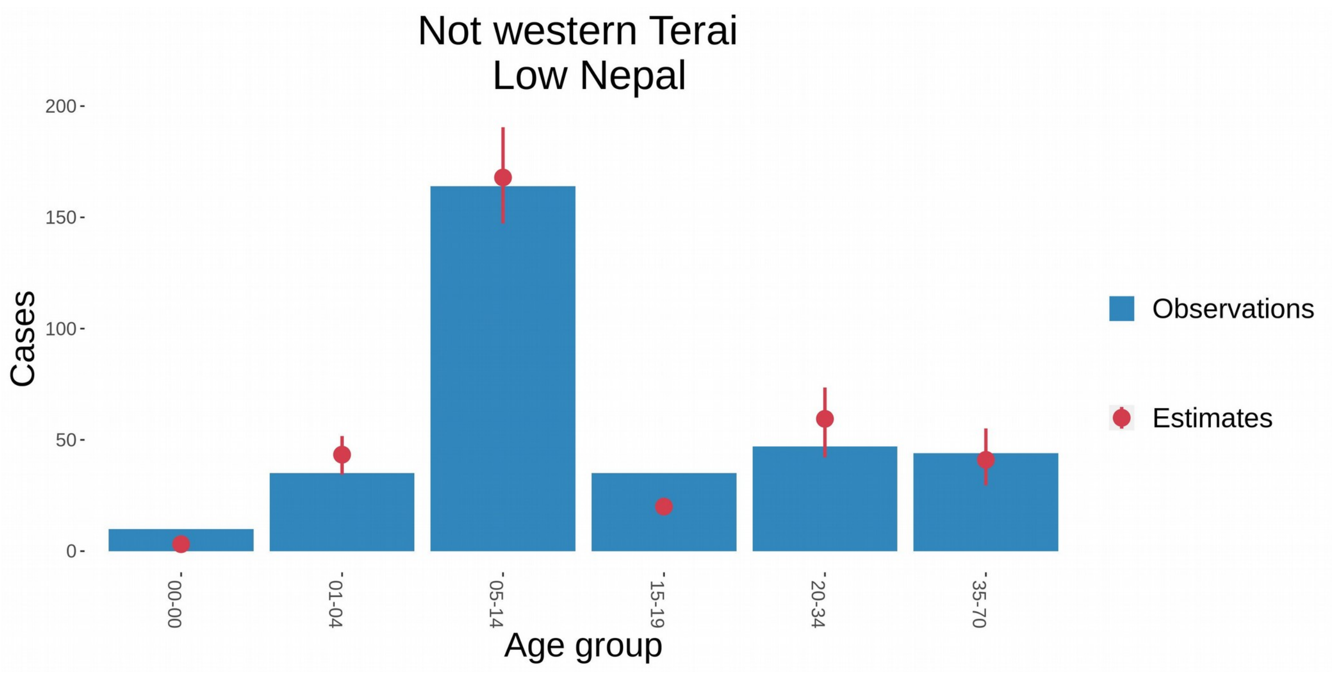

Fig4- Supp1 – 37. Model fit of all age-stratified case data in a study of Pondicherry in medium incidence region in India. For each study, the red dots with red vertical lines are the mean cases by age group estimated from the model with 95% credible interval. The blue bars are the cases reported by each age group.

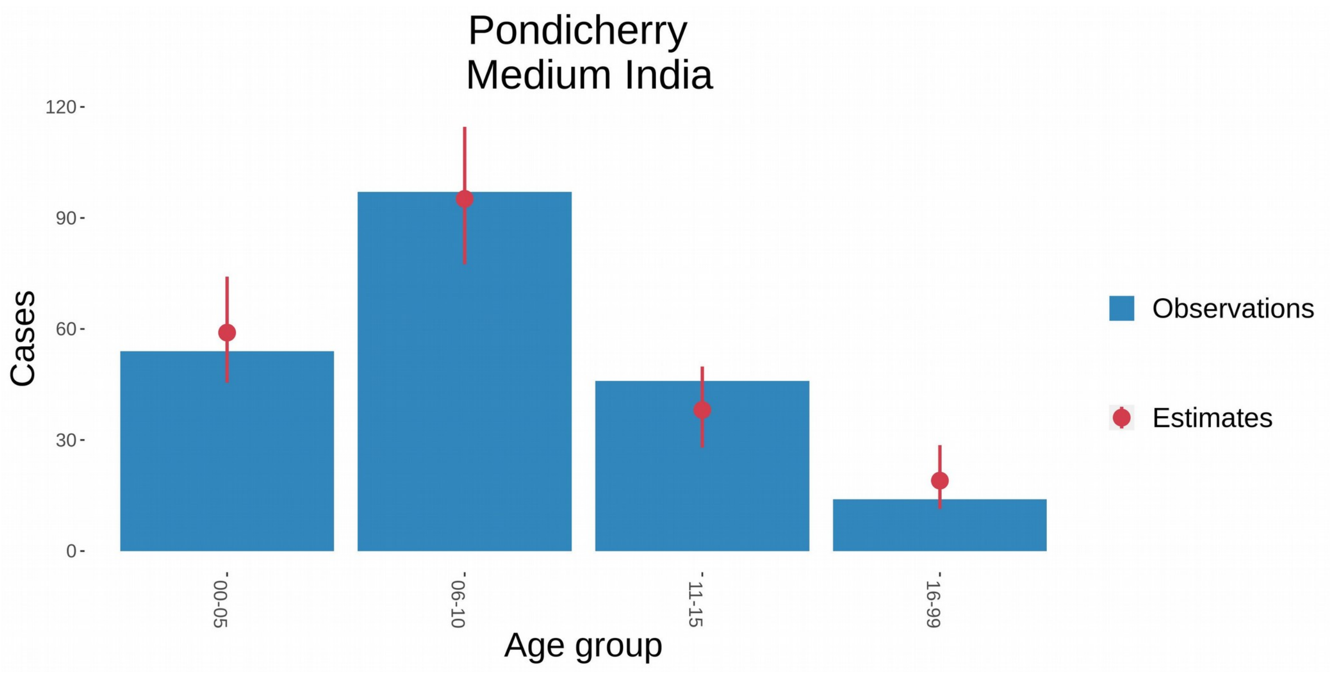

Fig4- Supp1 – 38. Model fit of all age-stratified case data in a study of Shijiazhuang in low incidence region in China. For each study, the red dots with red vertical lines are the mean cases by age group estimated from the model with 95% credible interval. The blue bars are the cases reported by each age group.

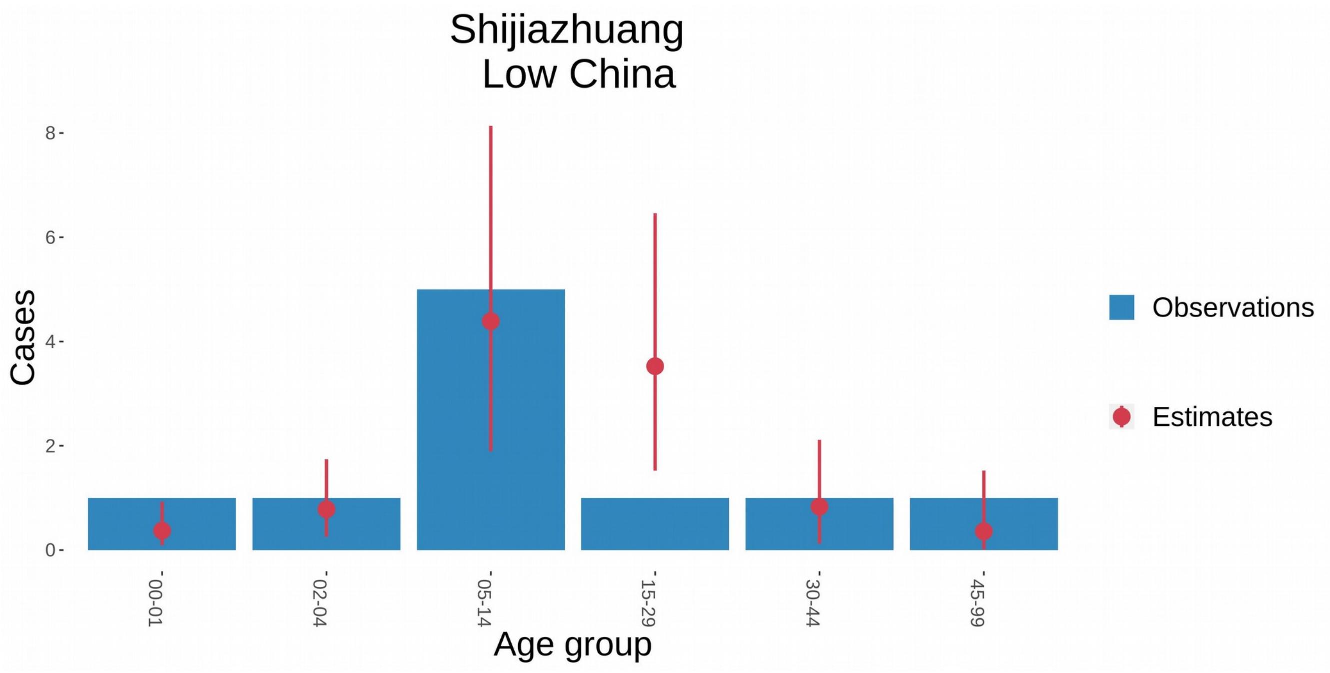

Fig4- Supp1 – 39. Model fit of all age-stratified case data in a study in Southern Taiwan. For each study, the red dots with red vertical lines are the mean cases by age group estimated from the model with 95% credible interval. The blue bars are the cases reported by each age group.

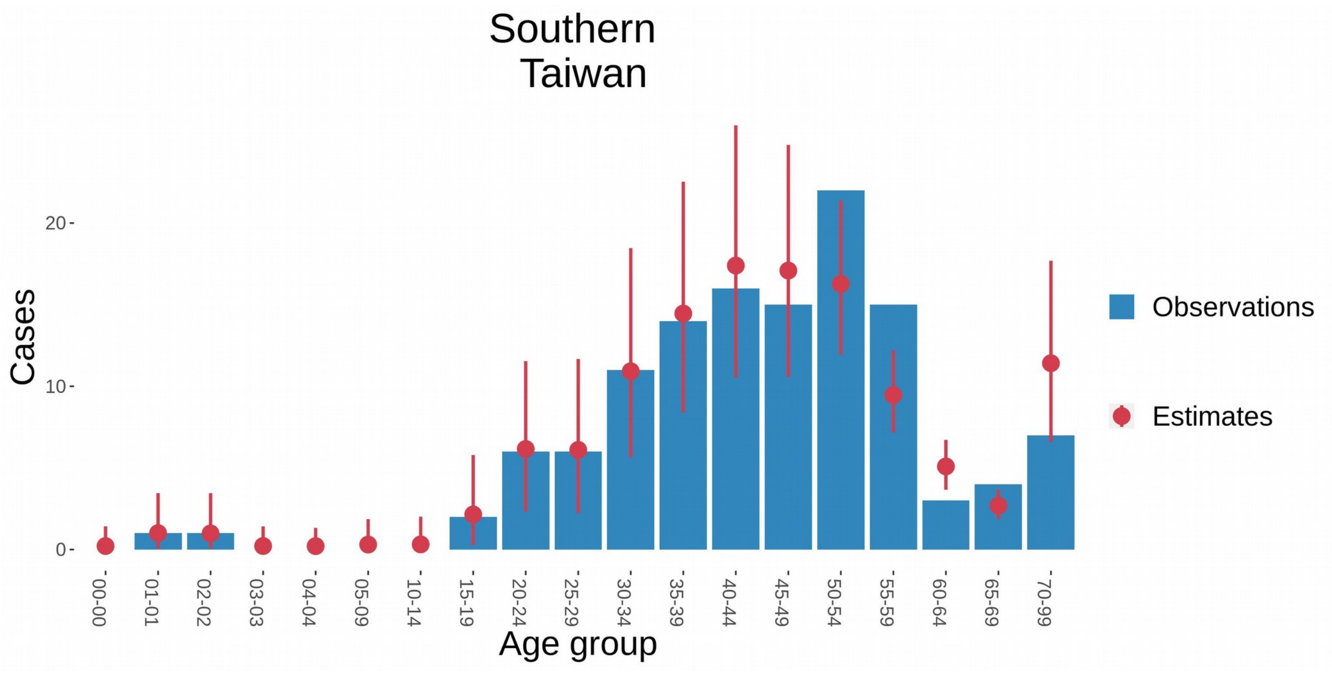

Fig4- Supp1 – 40. Model fit of all age-stratified case data in a study of Taipei in Taiwan. For each study, the red dots with red vertical lines are the mean cases by age group estimated from the model with 95% credible interval. The blue bars are the cases reported by each age group.

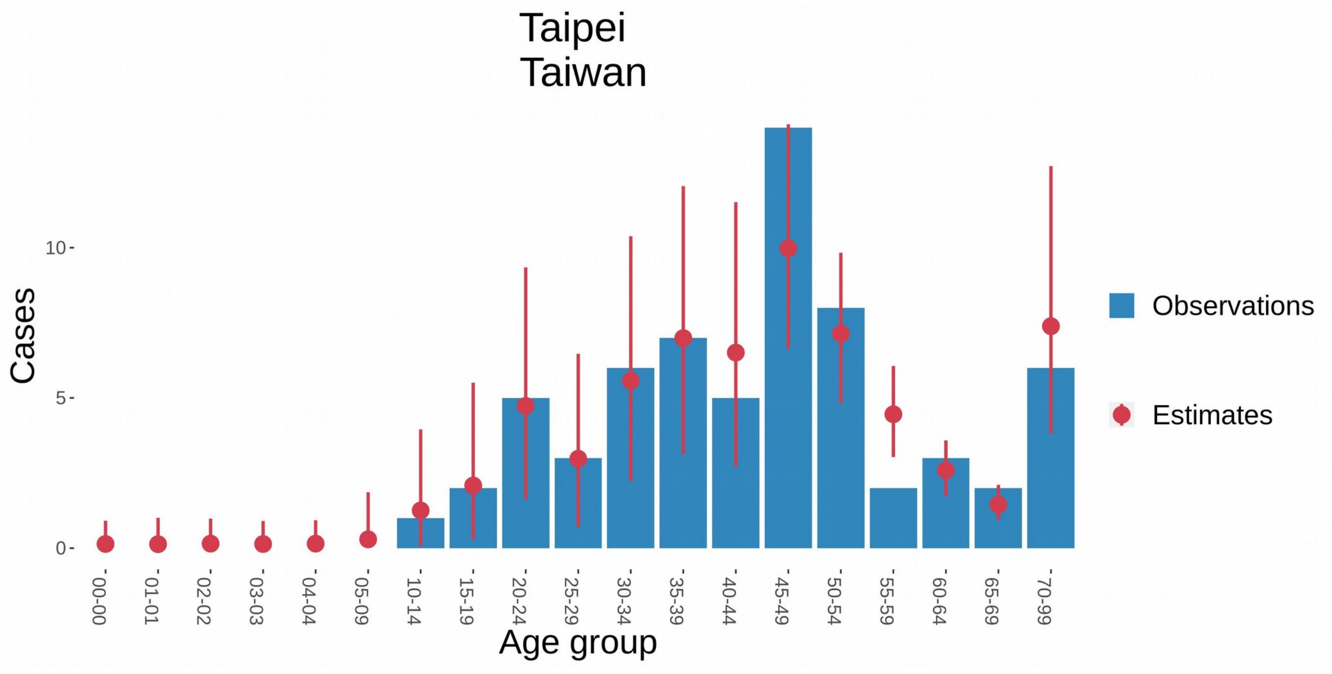

Fig4- Supp1 – 41. Model fit of all age-stratified case data in a study of Tamil Nadu in medium incidence region in India. For each study, the red dots with red vertical lines are the mean cases by age group estimated from the model with 95% credible interval. The blue bars are the cases reported by each age group.

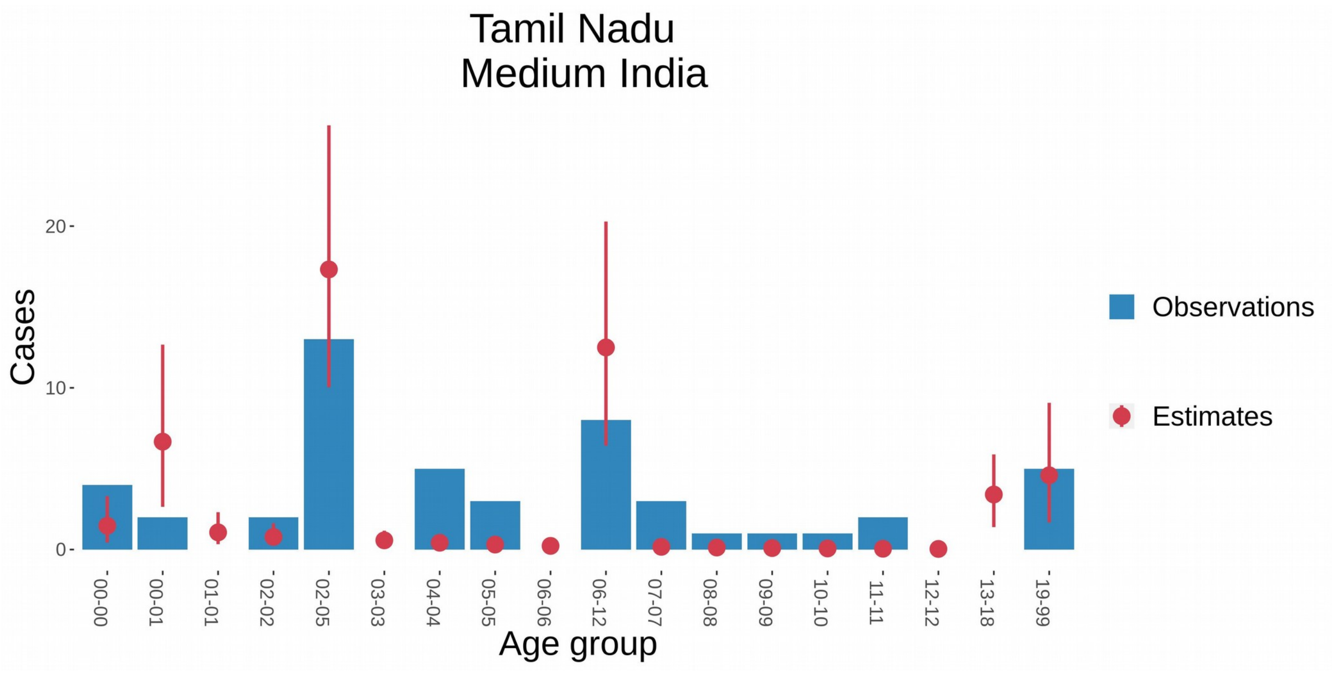

Fig4- Supp1 – 42. Model fit of all age-stratified case data in a study of Uttar Pradesh in high incidence region in India. For each study, the red dots with red vertical lines are the mean cases by age group estimated from the model with 95% credible interval. The blue bars are the cases reported by each age group.

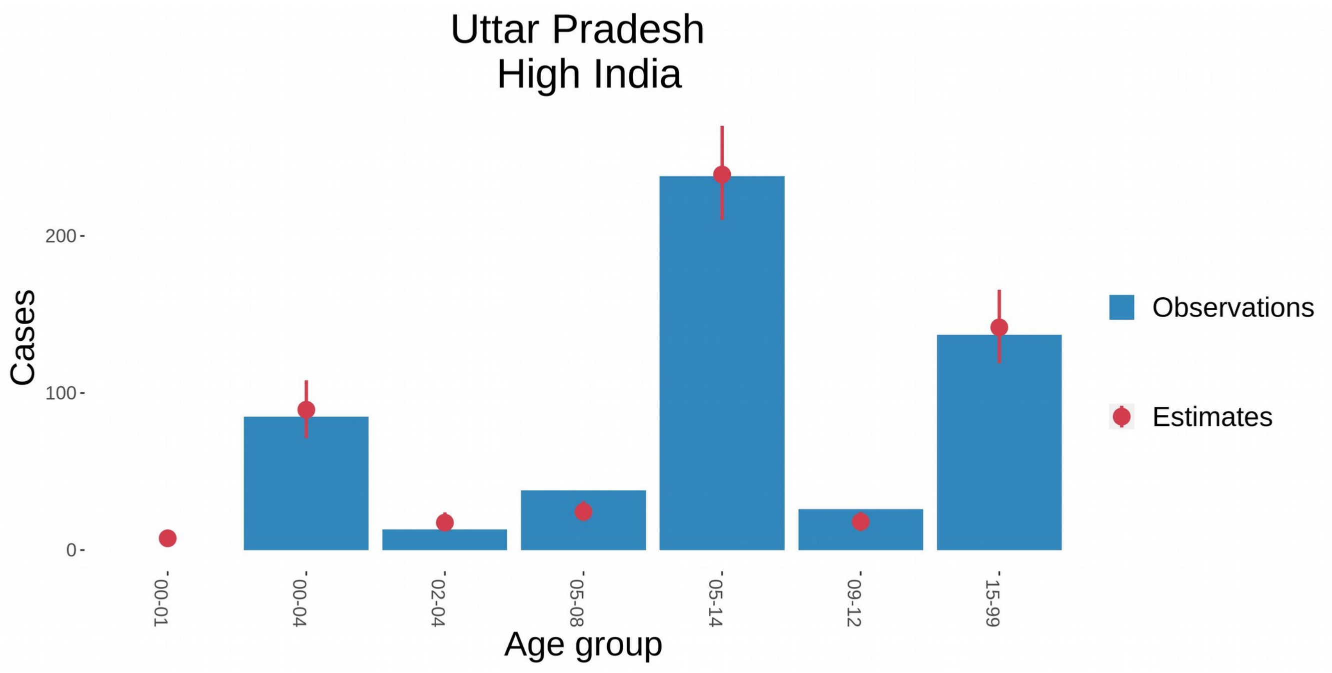

Fig4- Supp1 – 43. Model fit of all age-stratified case data in a study of Vientiane in Laos. For each study, the red dots with red vertical lines are the mean cases by age group estimated from the model with 95% credible interval. The blue bars are the cases reported by each age group.

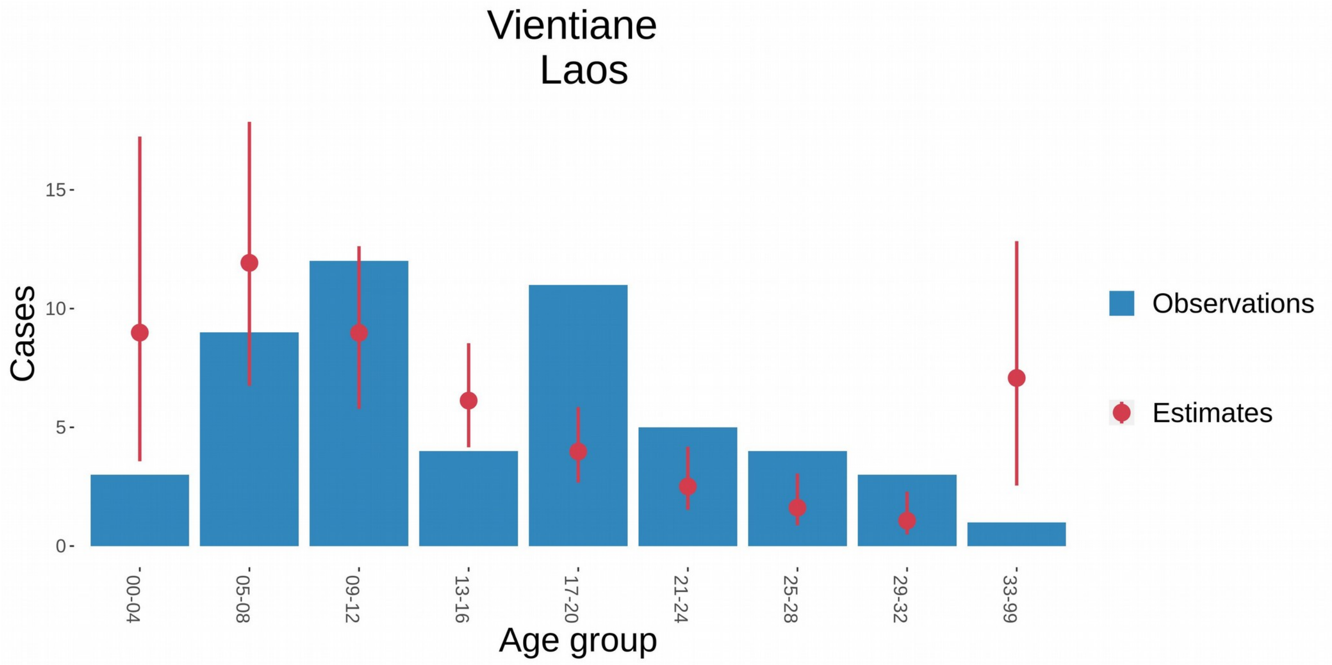

Fig4- Supp1 – 44. Model fit of all age-stratified case data in a study of West Bengal in medium incidence region in India. For each study, the red dots with red vertical lines are the mean cases by age group estimated from the model with 95% credible interval. The blue bars are the cases reported by each age group.

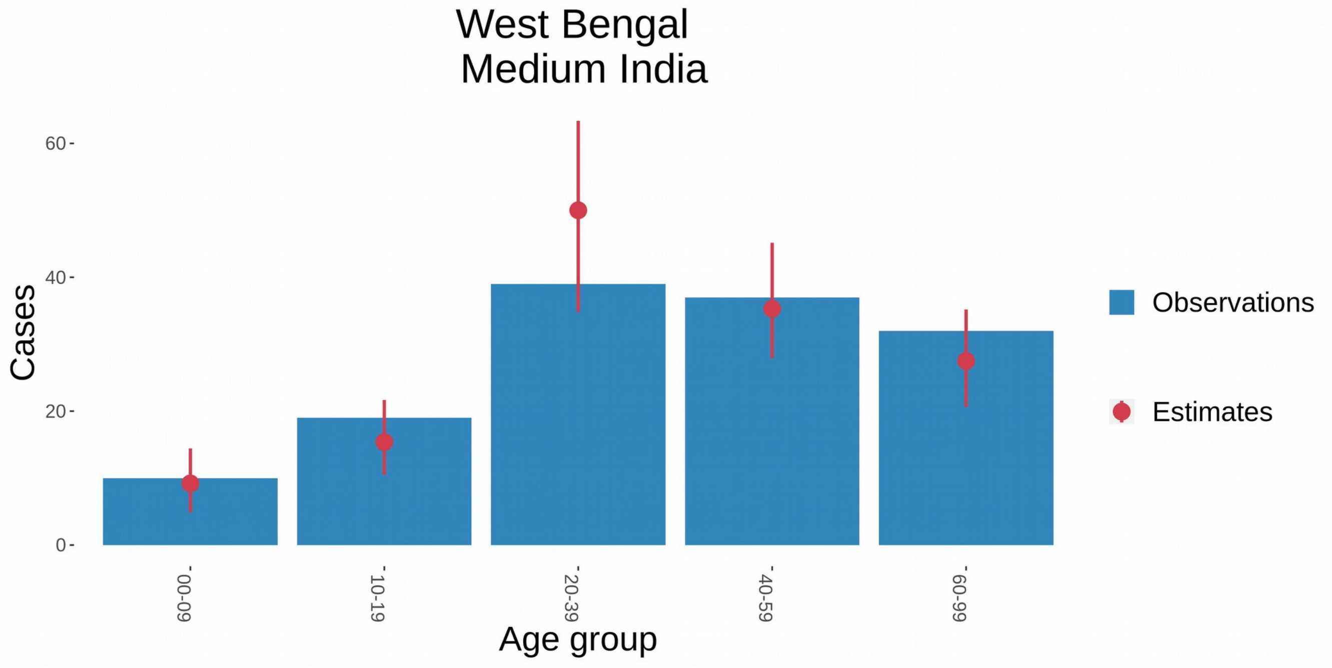

Fig4- Supp1 – 45. Model fit of all age-stratified case data in a study of Western Terai in high incidence region in Nepal. For each study, the red dots with red vertical lines are the mean cases by age group estimated from the model with 95% credible interval. The blue bars are the cases reported by each age group.

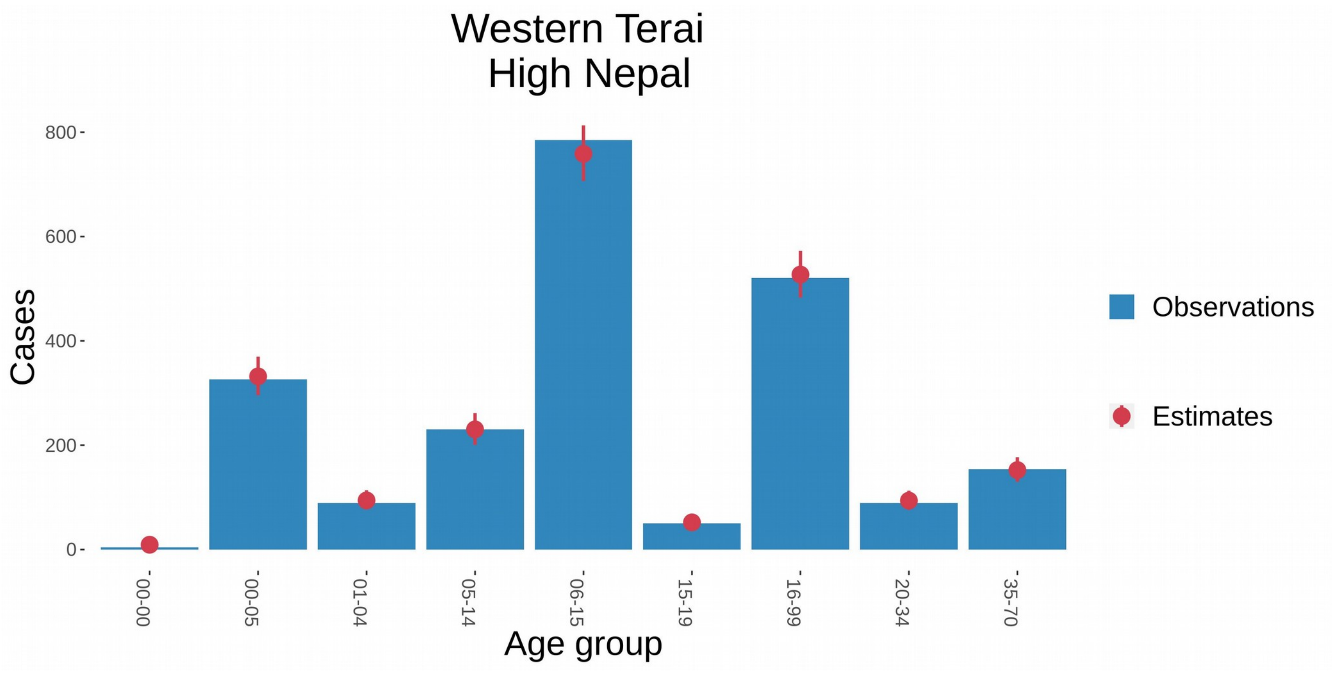

Fig4- Supp1 – 46. Model fit of all age-stratified case data in a study of Yichang in high incidence region in China. For each study, the red dots with red vertical lines are the mean cases by age group estimated from the model with 95% credible interval. The blue bars are the cases reported by each age group.

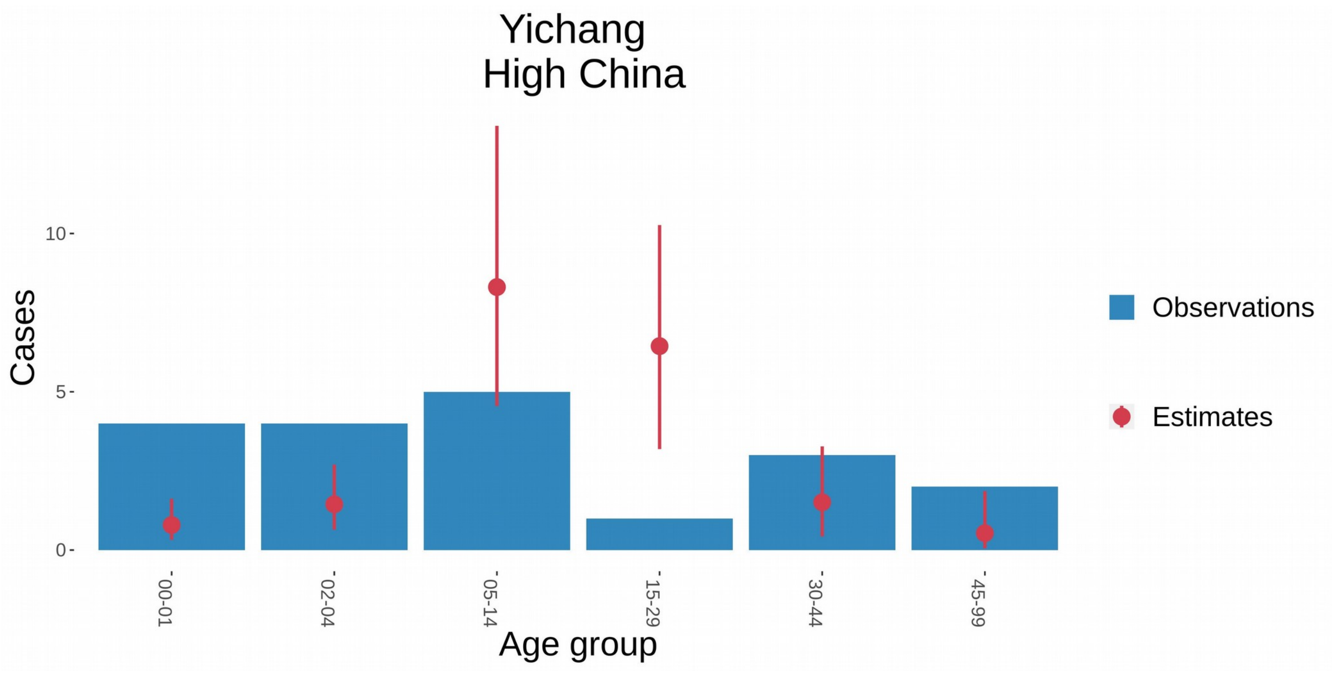

Supplement: Figure 4—source data 2. — For each study, the red dots with red vertical lines are the mean cases by age group estimated from the model with 95% credible interval. The blue dots are the cases by each age group. [file elife-51027-fig4-data2.pdf]
