# Supplementary material for: Estimates of the global burden of Japanese encephalitis and the impact of vaccination from 2000-2015
Source: eLife. 2020 May 26;9:e51027. doi: 10.7554/eLife.51027 (PMC7282807; doi:10.7554/eLife.51027)
Supplement: Figure 4—source data 4. [file elife-51027-fig4-data4.pdf]

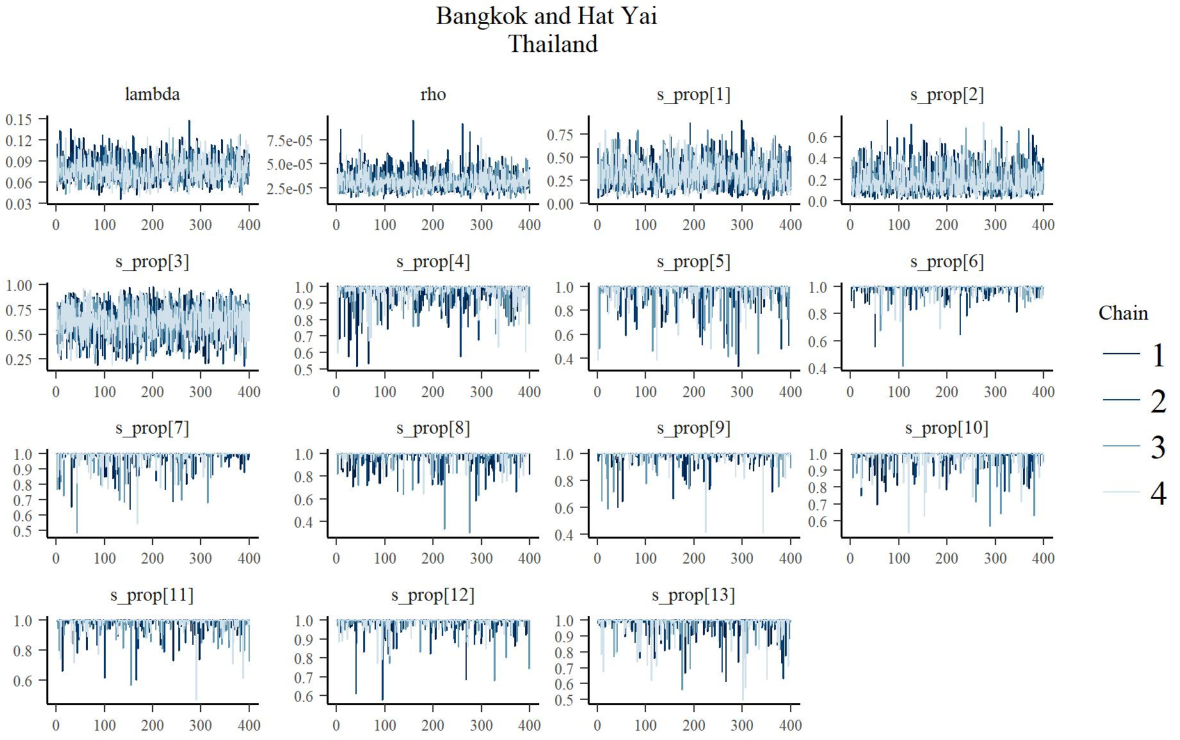
Bangkok and Hat Yai
Thailand
lambda
rho
s_prop[1]
s_prop[2]
s_prop[3]
s_prop[4]
s_prop[5]
s_prop[6]
s_prop[7]
s_prop[8]
s_prop[9]
s_prop[10]
s_prop[11]
s_prop[12]
s_prop[13]
Chain
1
2
3
4

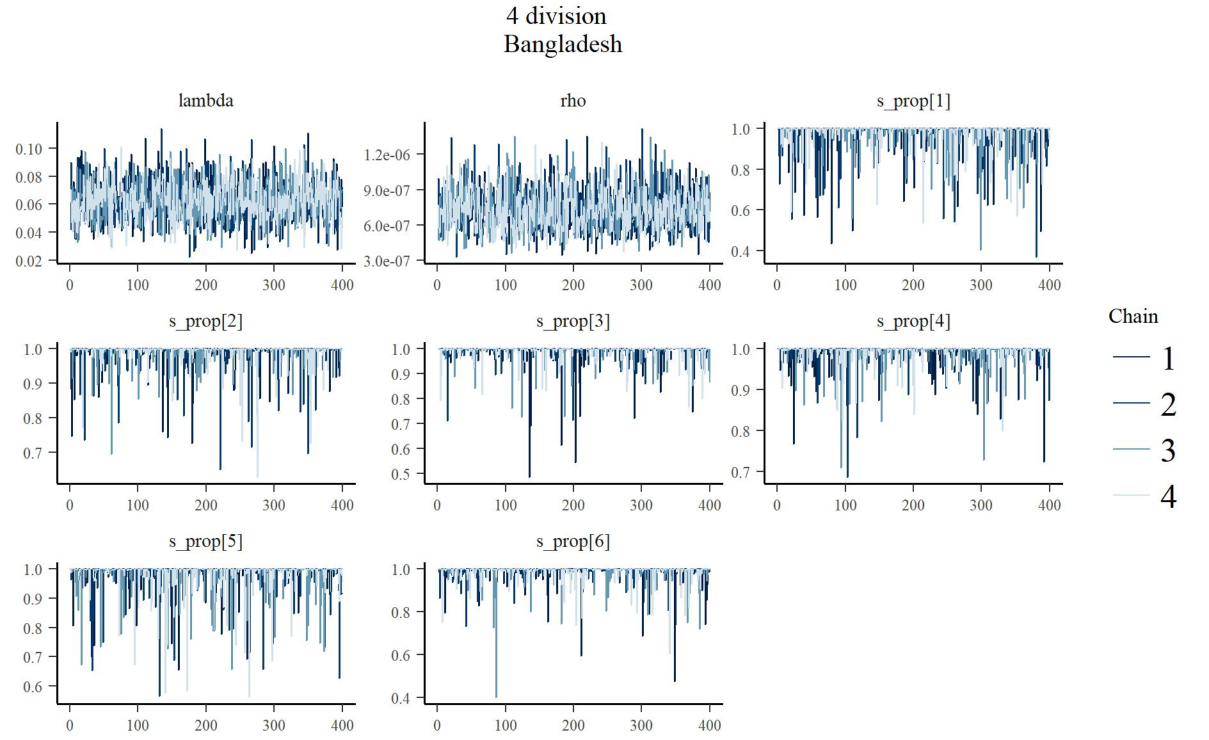

4 division
Bangladesh
lambda
rho
s_prop[1]
s_prop[2]
s_prop[3]
s_prop[4]
s_prop[5]
s_prop[6]
Chain
1
2
3
4

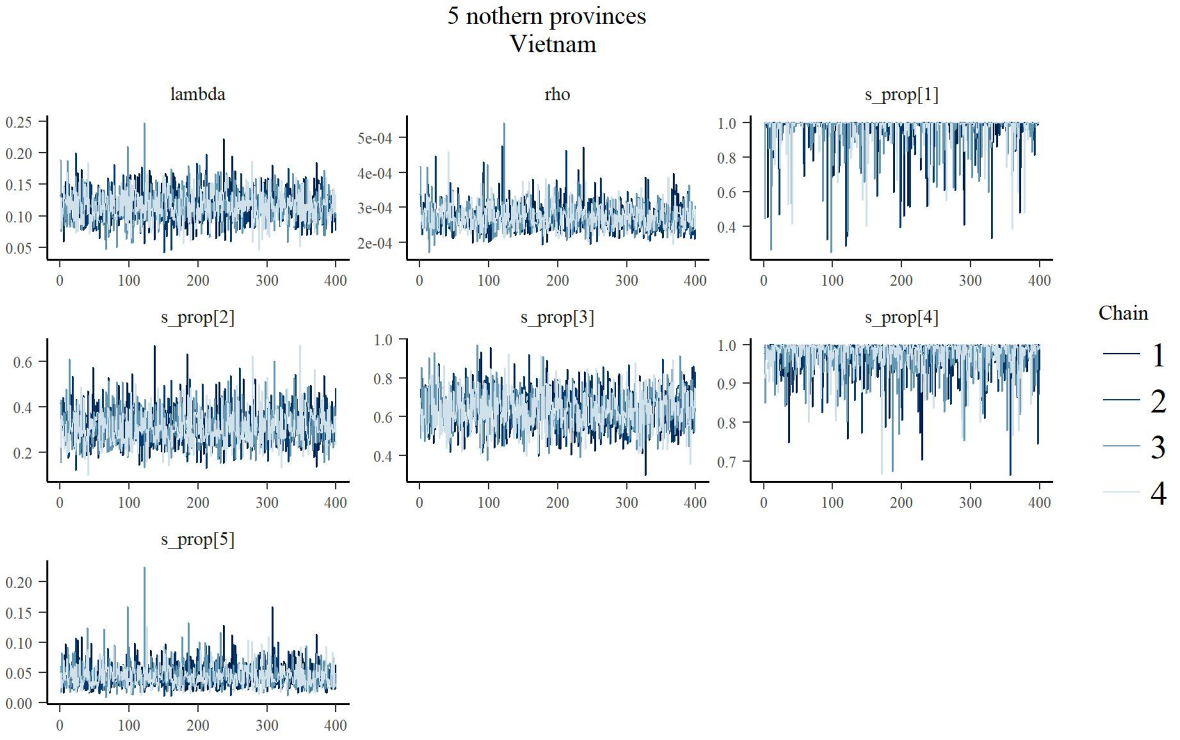
5 nothern provinces
Vietnam
lambda
rho
s_prop[1]
s_prop[2]
s_prop[3]
s_prop[4]
s_prop[5]
Chain
1
2
3
4

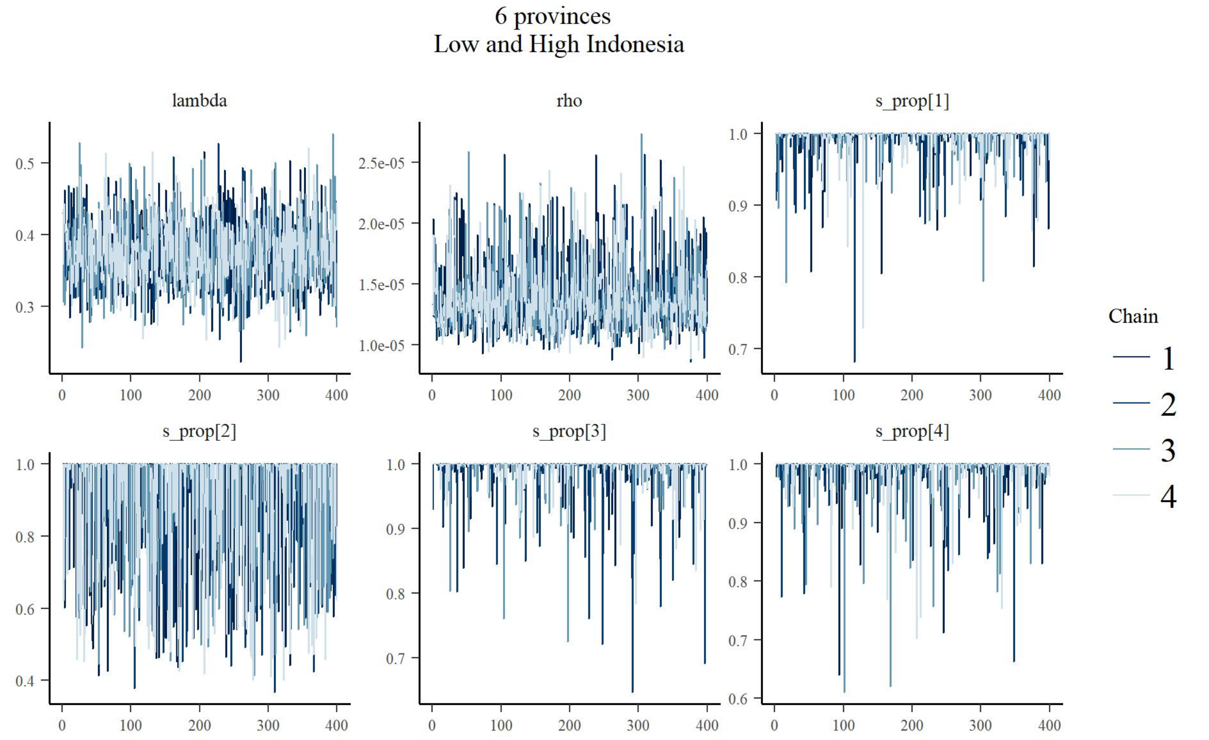
6 provinces
Low and High Indonesia
lambda
rho
s_prop[1]
s_prop[2]
s_prop[3]
s_prop[4]
Chain
1
2
3
4
0.3
0.4
0.5
1.0e-05
1.5e-05
2.0e-05
2.5e-05
0.6
0.7
0.8
0.9
1.0
0
100
200
300
400

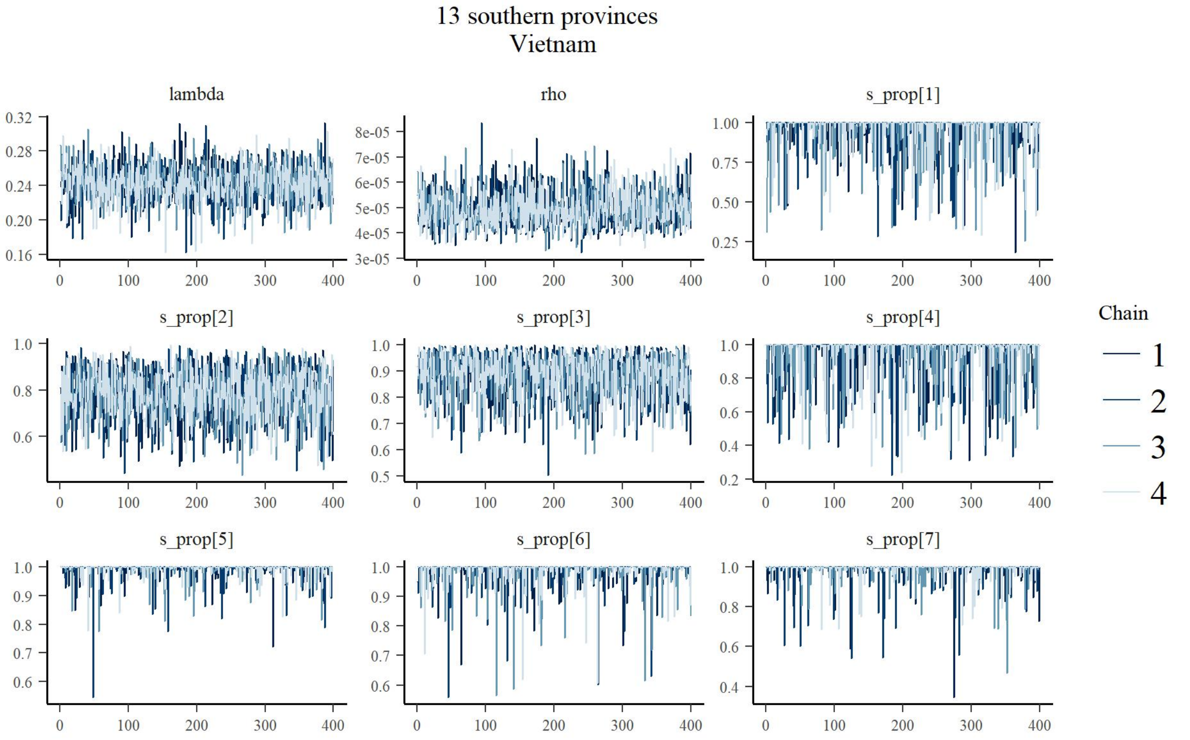
13 southern provinces
Vietnam
lambda
rho
s_prop[1]
s_prop[2]
s_prop[3]
s_prop[4]
s_prop[5]
s_prop[6]
s_prop[7]
Chain
1
2
3
4

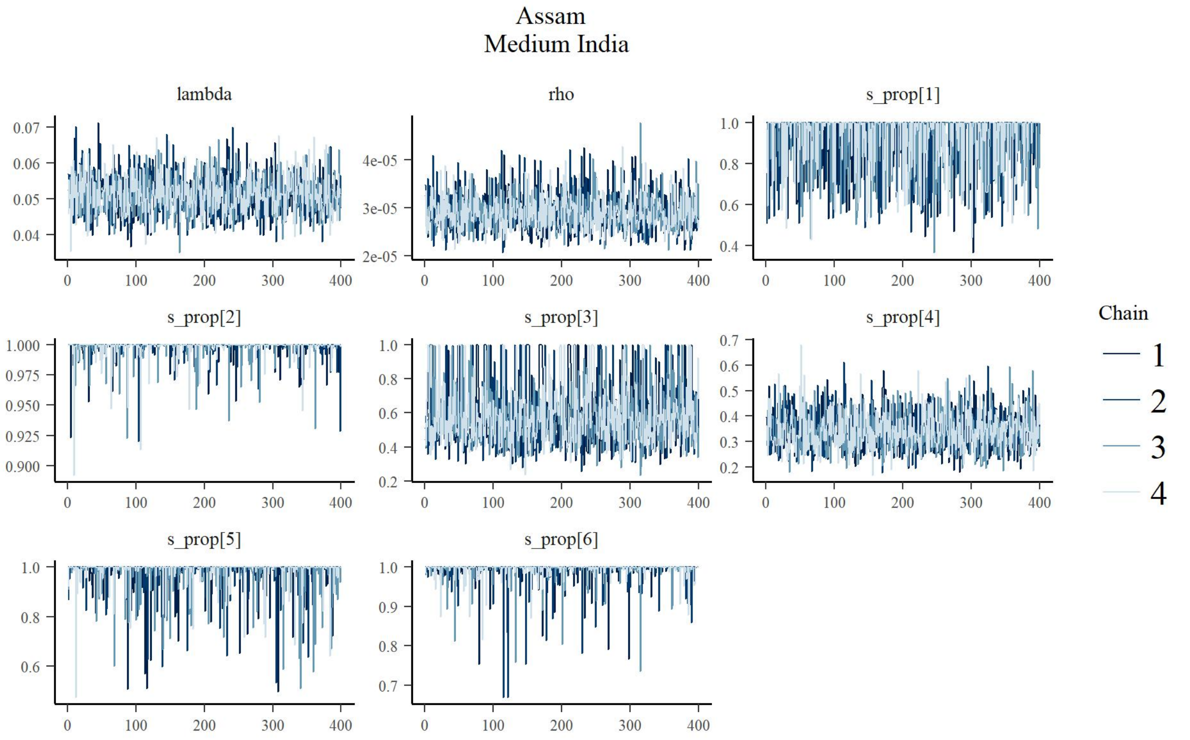

Assam
Medium India
lambda
rho
s_prop[1]
s_prop[2]
s_prop[3]
s_prop[4]
s_prop[5]
s_prop[6]
Chain
1
2
3
4
0.07
0.06
0.05
0.04
4e-05
3e-05
2e-05
1.0
0.8
0.6
0.4
1.000
0.975
0.950
0.925
0.900
0.2
0.7
0.5
0.3
0.9
0
100
200
300
400

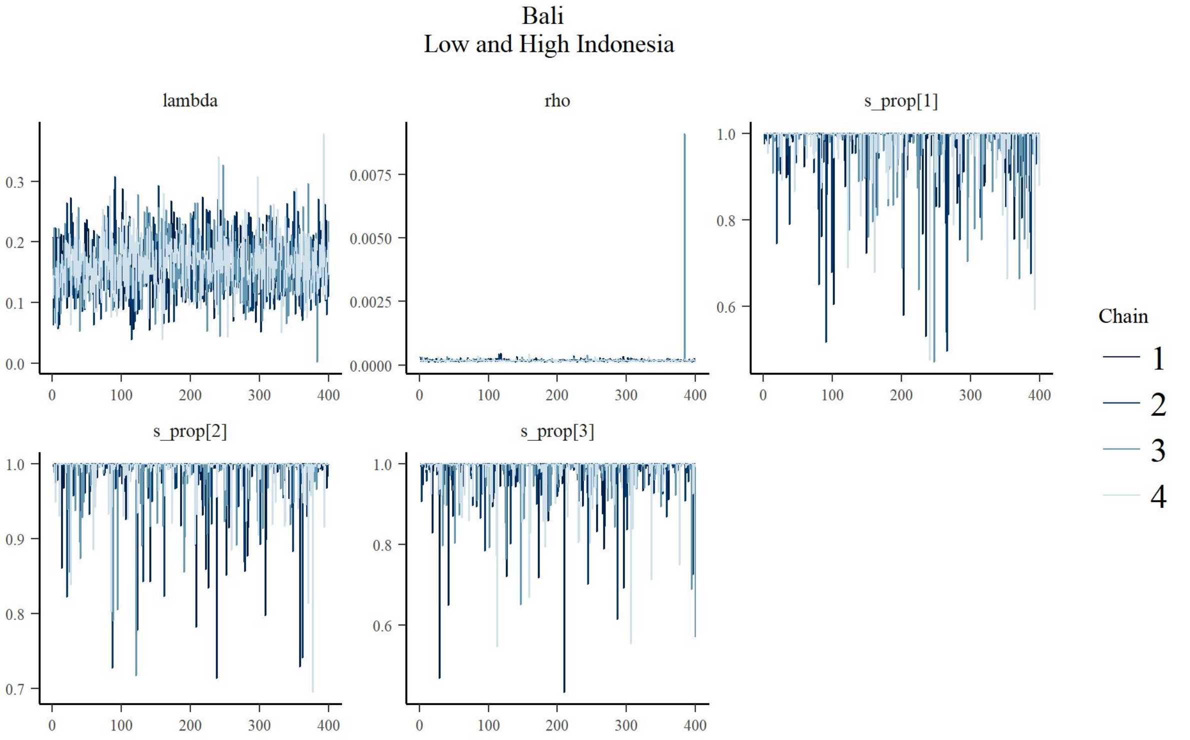

Bali
Low and High Indonesia
lambda
rho
s_prop[1]
s_prop[2]
s_prop[3]
Chain
1
2
3
4

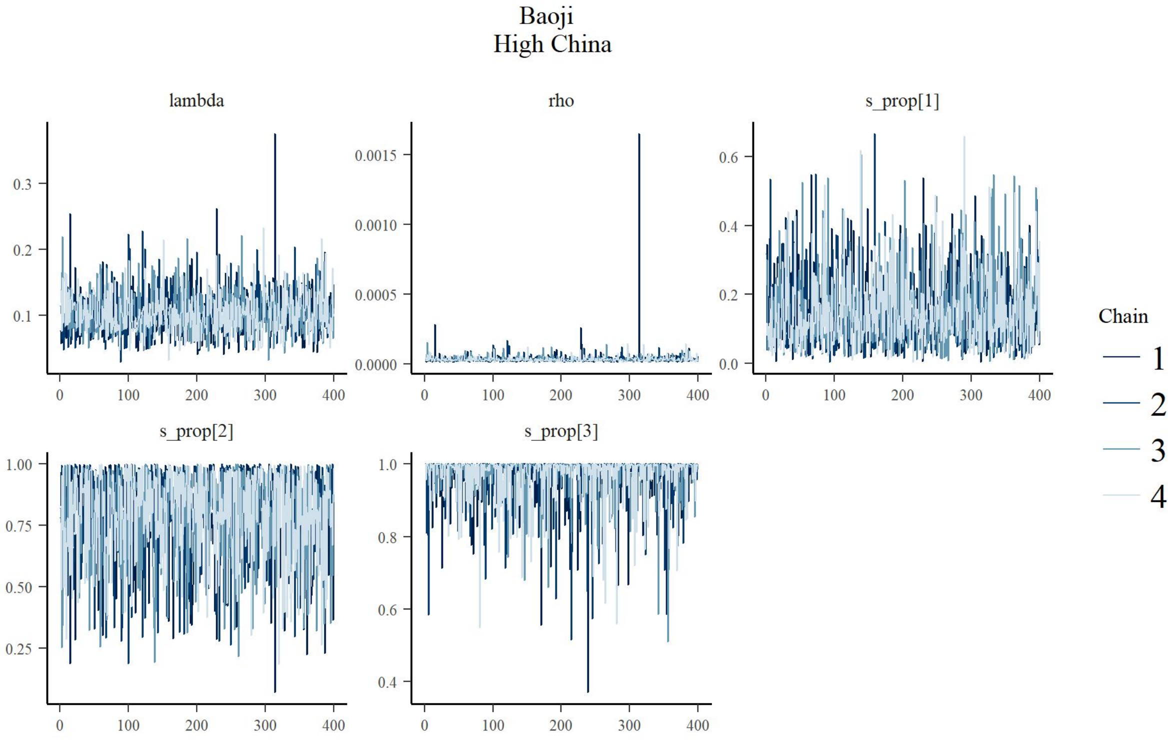

Baoji
High China
lambda
rho
s_prop[1]
s_prop[2]
s_prop[3]
Chain
1
2
3
4
0.1
0.2
0.3
0.0000
0.0005
0.0010
0.0015
0.0
0.2
0.4
0.6
0.25
0.50
0.75
1.00
0.4
0.6
0.8
1.0
0
100
200
300
400

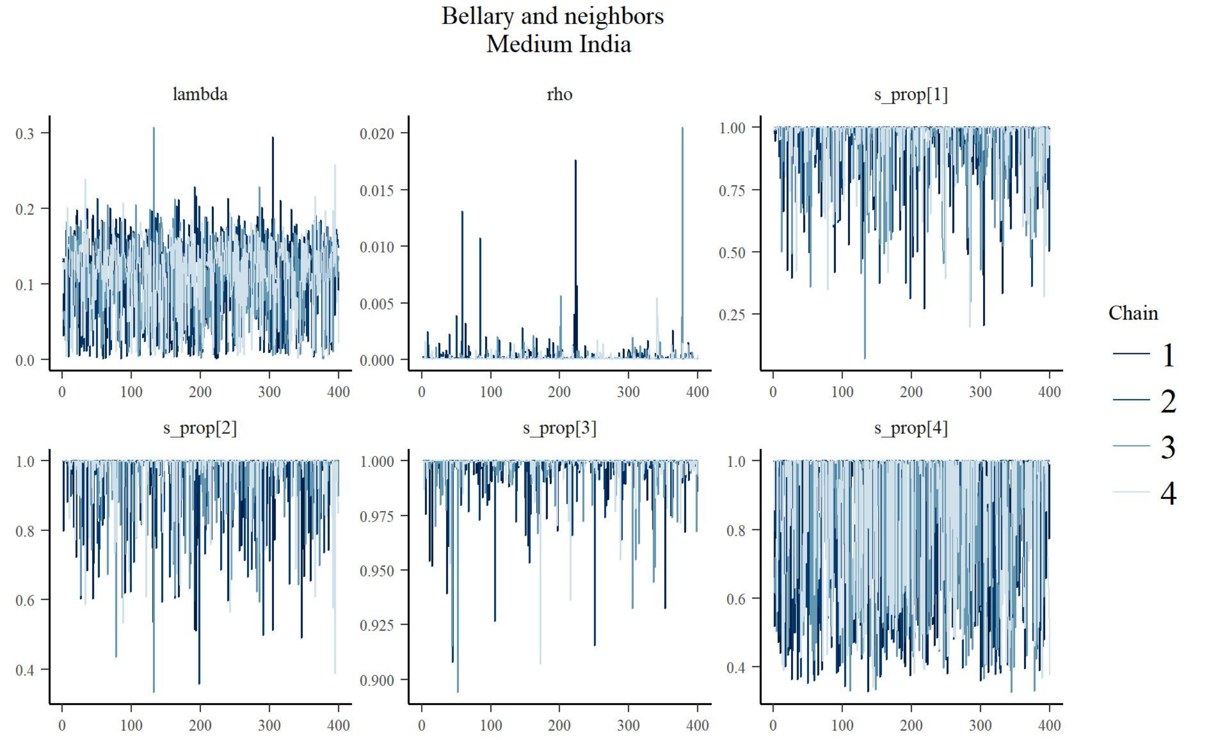

Bellary and neighbors
Medium India
lambda
rho
s_prop[1]
s_prop[2]
s_prop[3]
s_prop[4]
Chain
1
2
3
4
0.0
0.1
0.2
0.3
0.000
0.005
0.010
0.015
0.020
0.25
0.50
0.75
1.00
0.4
0.6
0.8
1.0
0.900
0.925
0.950
0.975
1.000
0
100
200
300
400

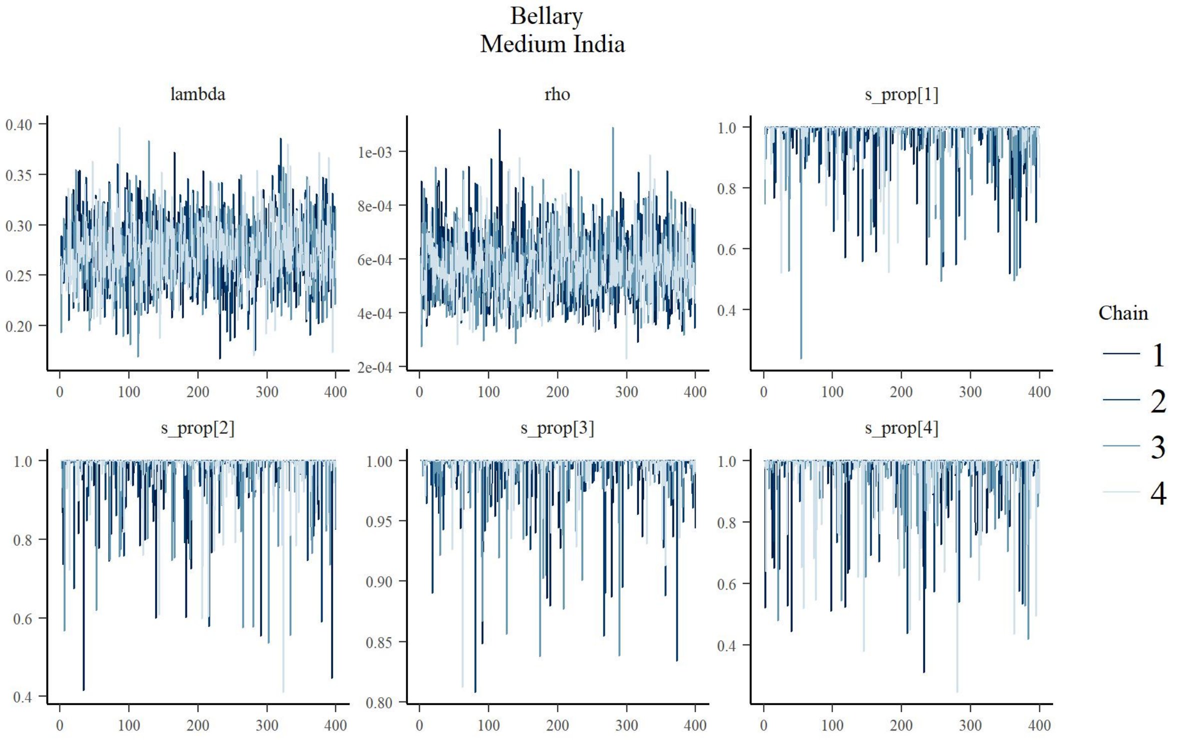

Bellary
Medium India
lambda
rho
s_prop[1]
s_prop[2]
s_prop[3]
s_prop[4]
Chain
1
2
3
4

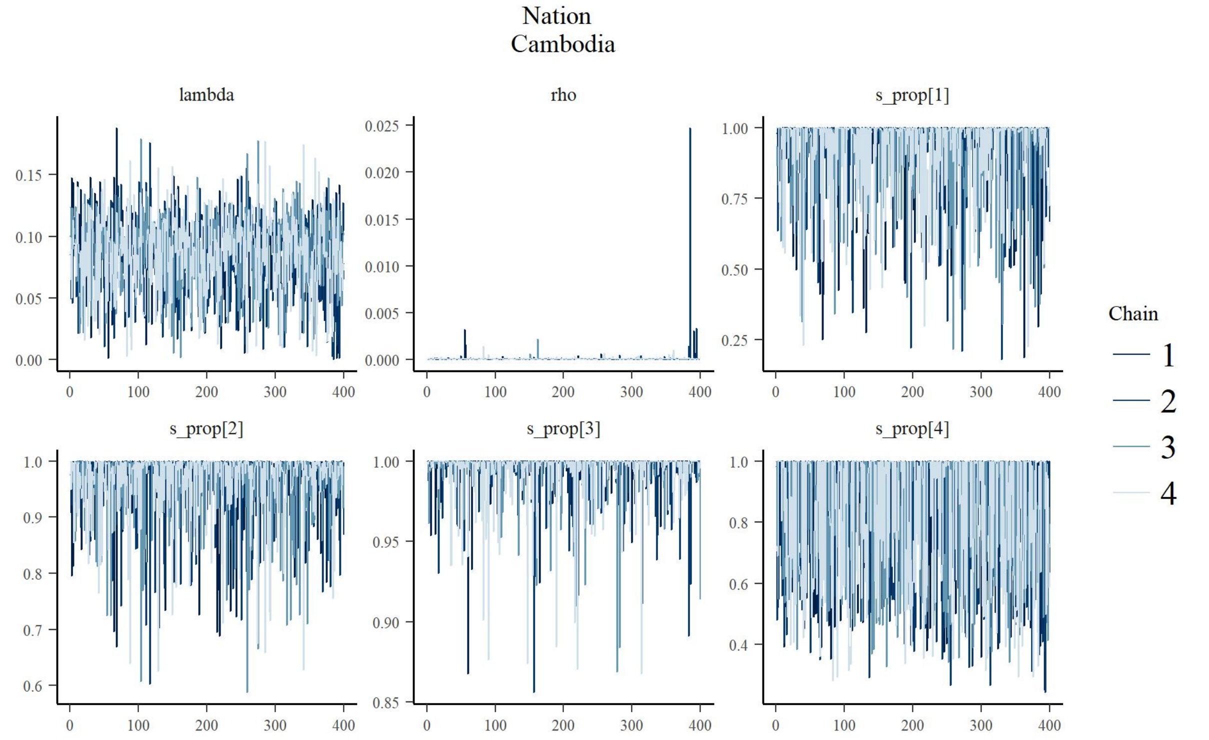
Nation
Cambodia
lambda
rho
s_prop[1]
s_prop[2]
s_prop[3]
s_prop[4]
Chain
1
2
3
4
0.15
0.10
0.05
0.00
0.025
0.020
0.015
0.010
0.005
0.000
1.00
0.75
0.50
0.25
1.0
0.9
0.8
0.7
0.6
1.00
0.95
0.90
0.85
1.0
0.8
0.6
0.4
0
100
200
300
400

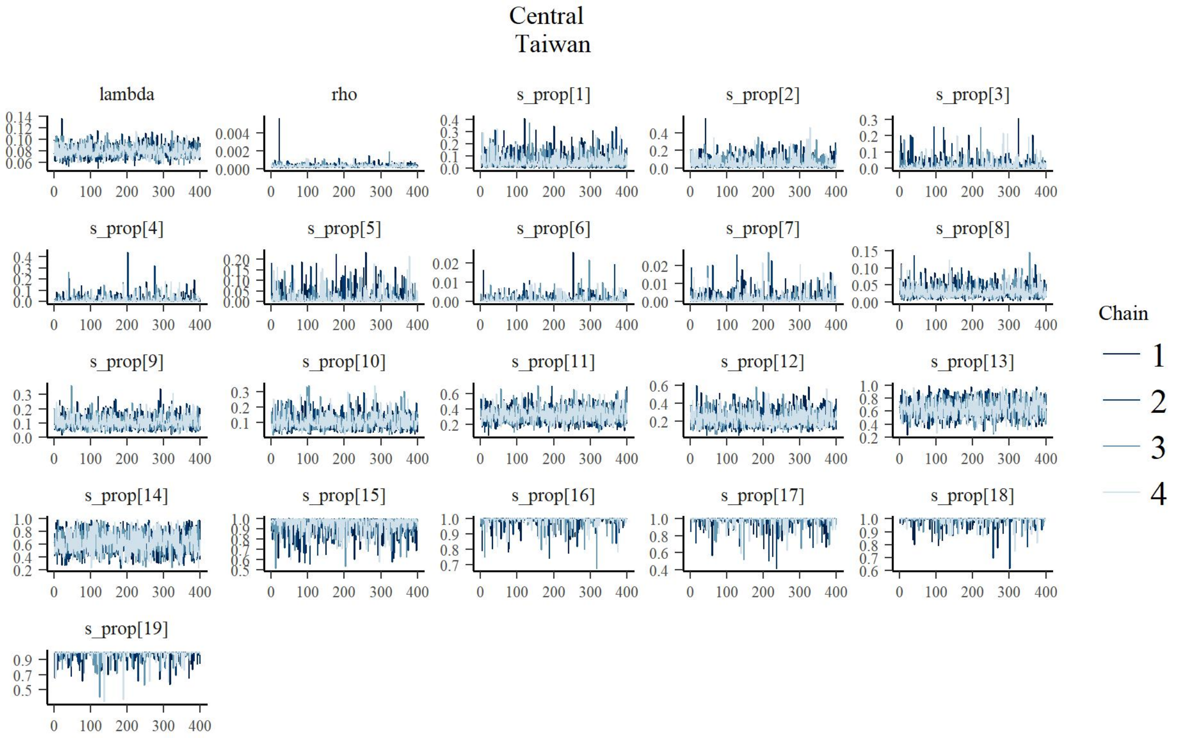
Central
Taiwan
lambda
rho
s_prop[1]
s_prop[2]
s_prop[3]
s_prop[4]
s_prop[5]
s_prop[6]
s_prop[7]
s_prop[8]
s_prop[9]
s_prop[10]
s_prop[11]
s_prop[12]
s_prop[13]
s_prop[14]
s_prop[15]
s_prop[16]
s_prop[17]
s_prop[18]
s_prop[19]
Chain
1
2
3
4

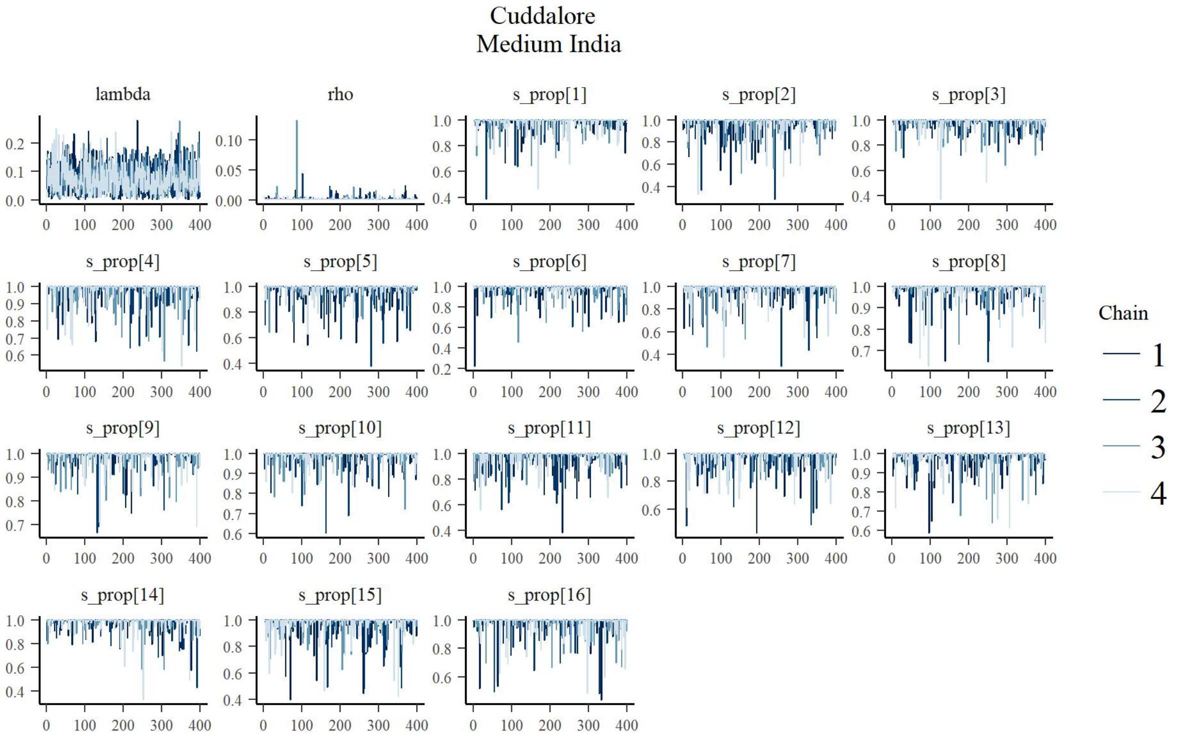
Cuddalore
Medium India
lambda
rho
s_prop[1]
s_prop[2]
s_prop[3]
s_prop[4]
s_prop[5]
s_prop[6]
s_prop[7]
s_prop[8]
s_prop[9]
s_prop[10]
s_prop[11]
s_prop[12]
s_prop[13]
s_prop[14]
s_prop[15]
s_prop[16]
Chain
1
2
3
4

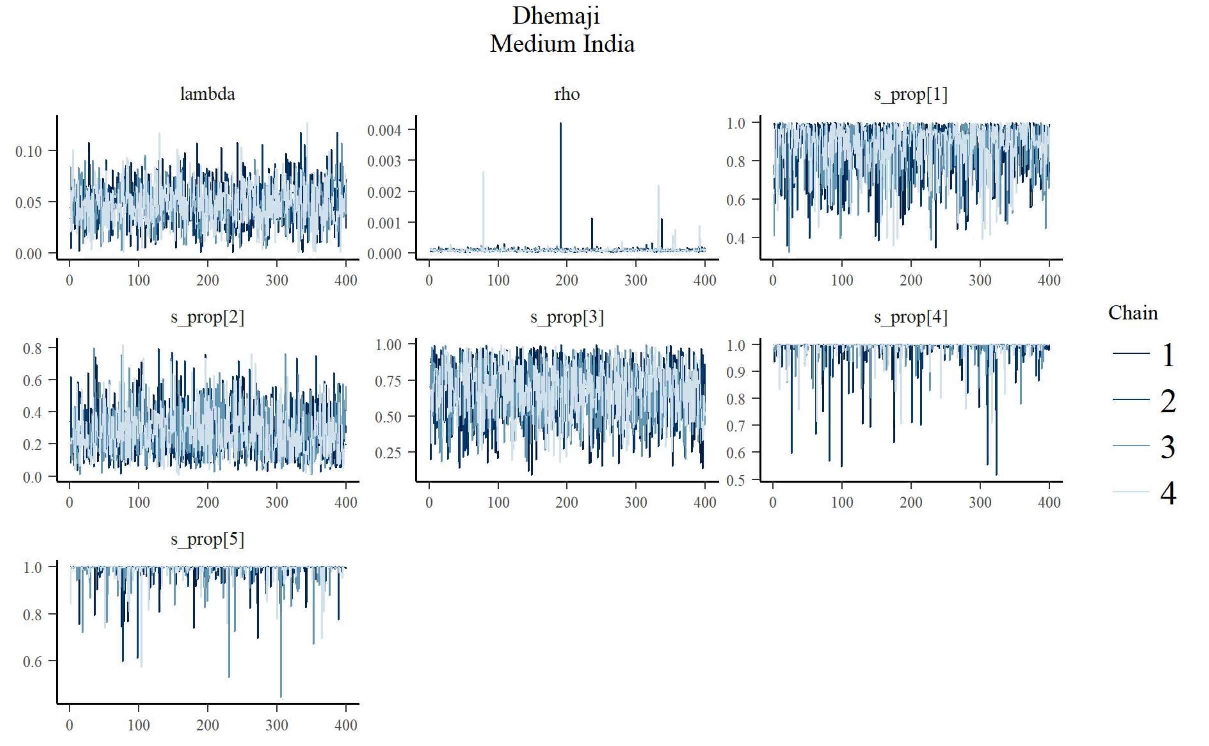

Dhemaji
Medium India
lambda
rho
s_prop[1]
s_prop[2]
s_prop[3]
s_prop[4]
s_prop[5]
Chain
1
2
3
4

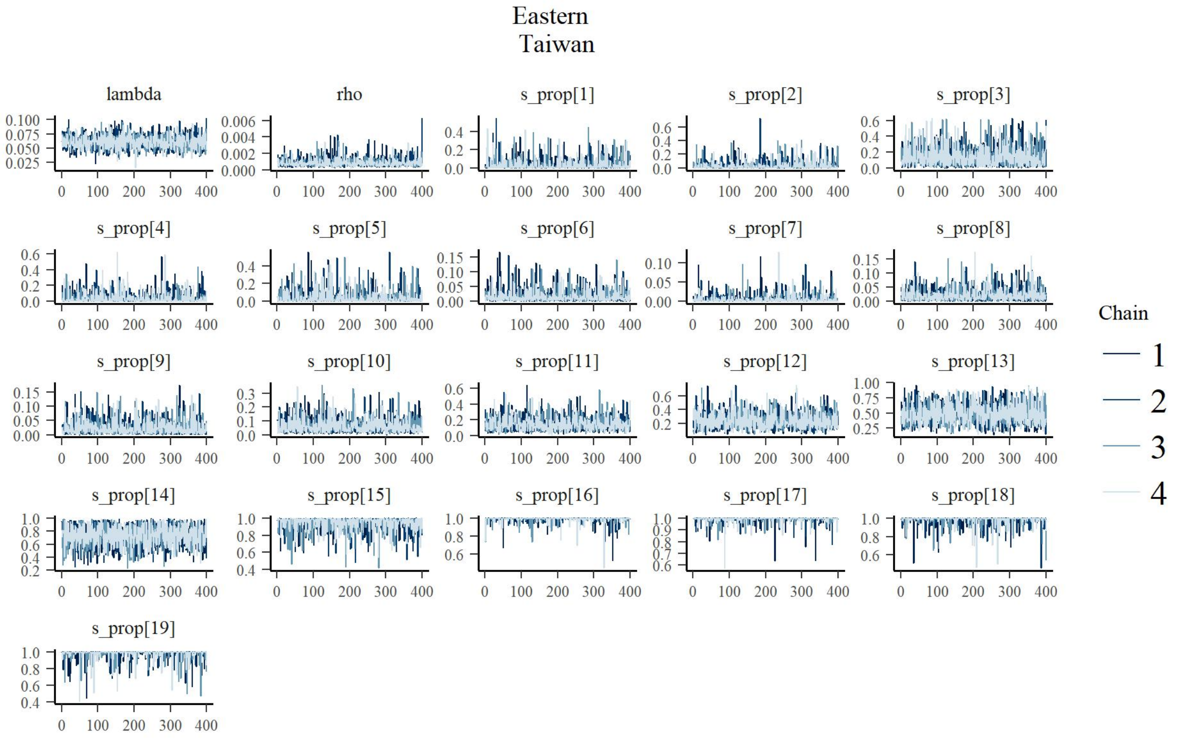

Eastern
Taiwan
lambda
rho
s_prop[1]
s_prop[2]
s_prop[3]
s_prop[4]
s_prop[5]
s_prop[6]
s_prop[7]
s_prop[8]
s_prop[9]
s_prop[10]
s_prop[11]
s_prop[12]
s_prop[13]
s_prop[14]
s_prop[15]
s_prop[16]
s_prop[17]
s_prop[18]
s_prop[19]
Chain
1
2
3
4

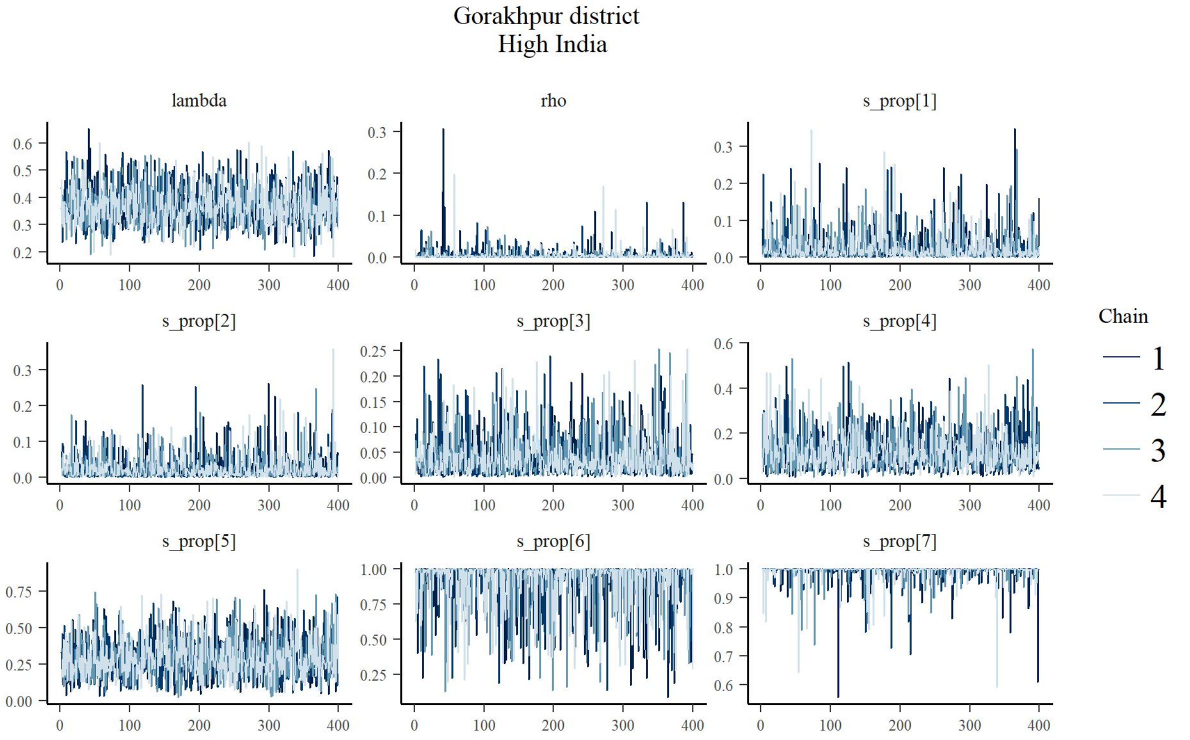
Gorakhpur district
High India
lambda
rho
s_prop[1]
s_prop[2]
s_prop[3]
s_prop[4]
s_prop[5]
s_prop[6]
s_prop[7]
Chain
1
2
3
4

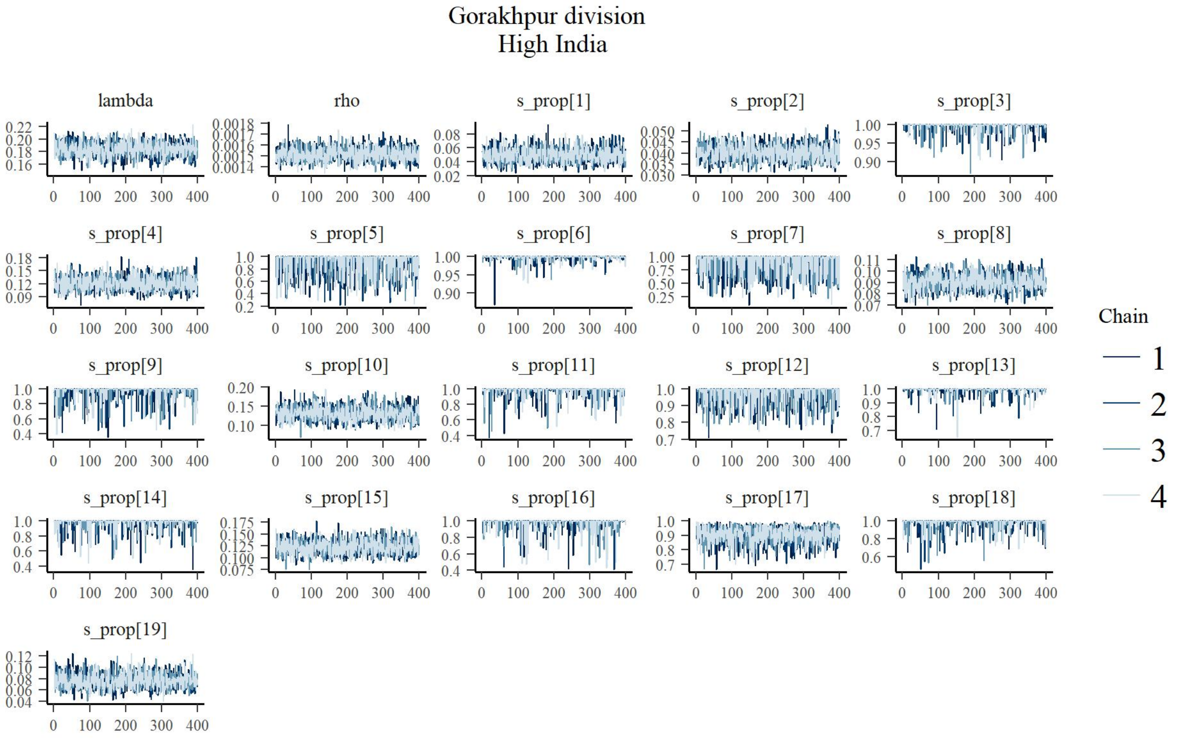

Gorakhpur division
High India
lambda
rho
s_prop[1]
s_prop[2]
s_prop[3]
s_prop[4]
s_prop[5]
s_prop[6]
s_prop[7]
s_prop[8]
s_prop[9]
s_prop[10]
s_prop[11]
s_prop[12]
s_prop[13]
s_prop[14]
s_prop[15]
s_prop[16]
s_prop[17]
s_prop[18]
s_prop[19]
Chain
1
2
3
4

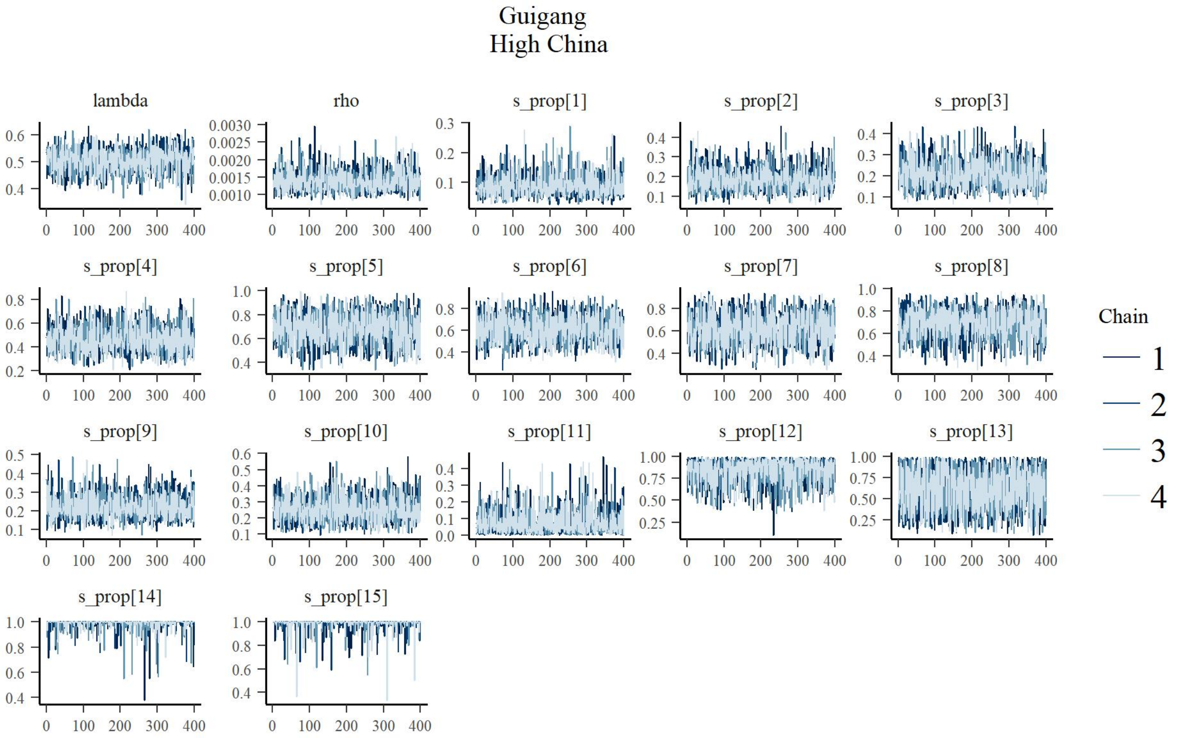
Guigang
High China
lambda
rho
s_prop[1]
s_prop[2]
s_prop[3]
s_prop[4]
s_prop[5]
s_prop[6]
s_prop[7]
s_prop[8]
s_prop[9]
s_prop[10]
s_prop[11]
s_prop[12]
s_prop[13]
s_prop[14]
s_prop[15]
Chain
1
2
3
4

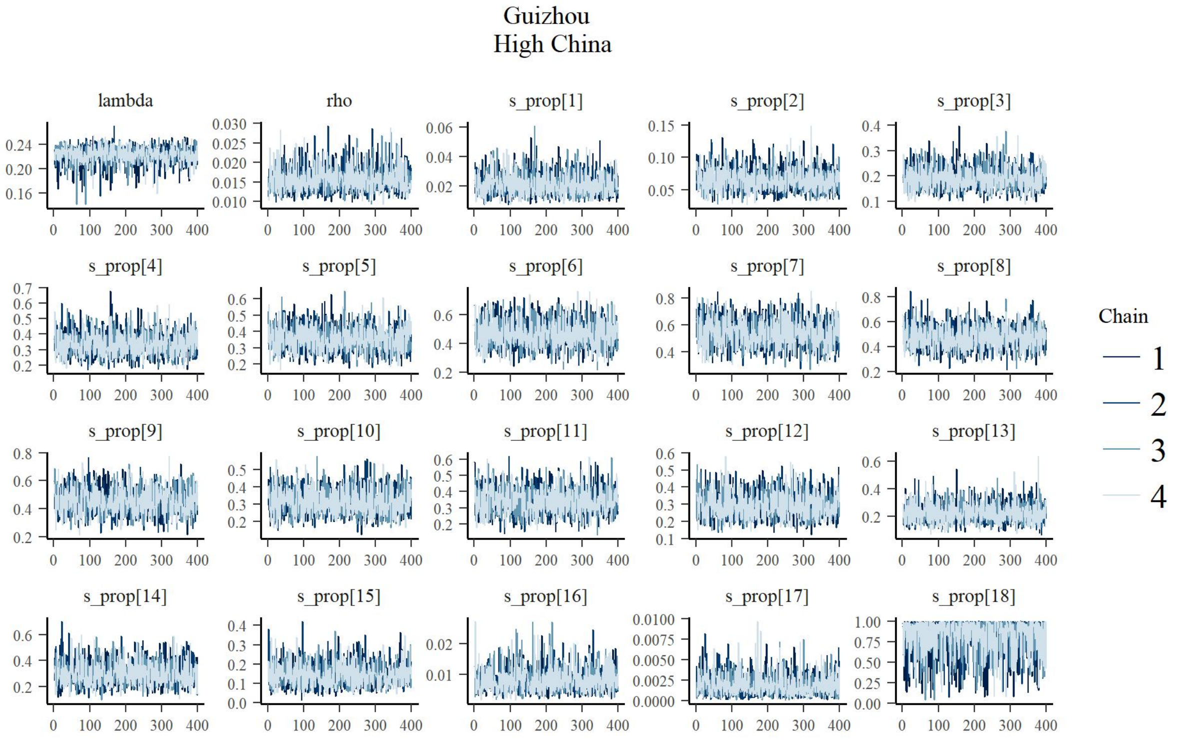
Guizhou
High China
lambda
rho
s_prop[1]
s_prop[2]
s_prop[3]
s_prop[4]
s_prop[5]
s_prop[6]
s_prop[7]
s_prop[8]
s_prop[9]
s_prop[10]
s_prop[11]
s_prop[12]
s_prop[13]
s_prop[14]
s_prop[15]
s_prop[16]
s_prop[17]
s_prop[18]
Chain
1
2
3
4

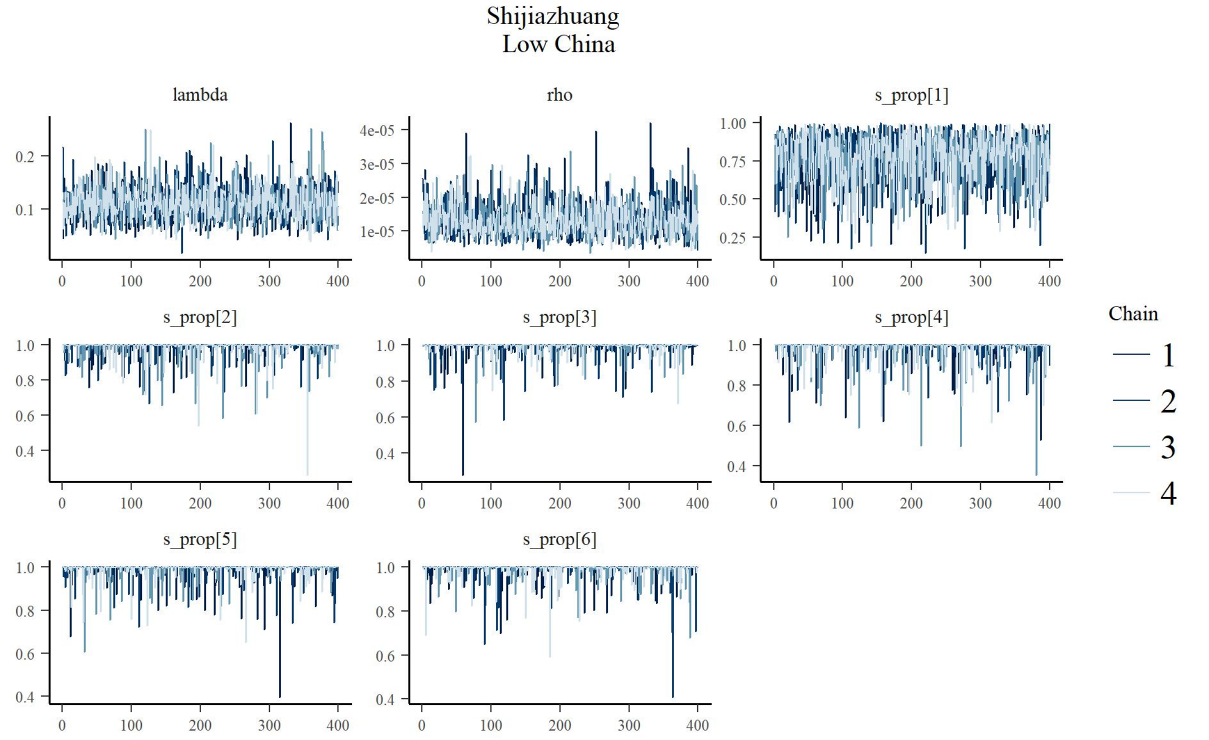
Shijiazhuang
Low China
lambda
rho
s_prop[1]
s_prop[2]
s_prop[3]
s_prop[4]
s_prop[5]
s_prop[6]
Chain
1
2
3
4
0.2
0.1
4e-05
3e-05
2e-05
1e-05
1.00
0.75
0.50
0.25
1.0
0.8
0.6
0.4
0
100
200
300
400

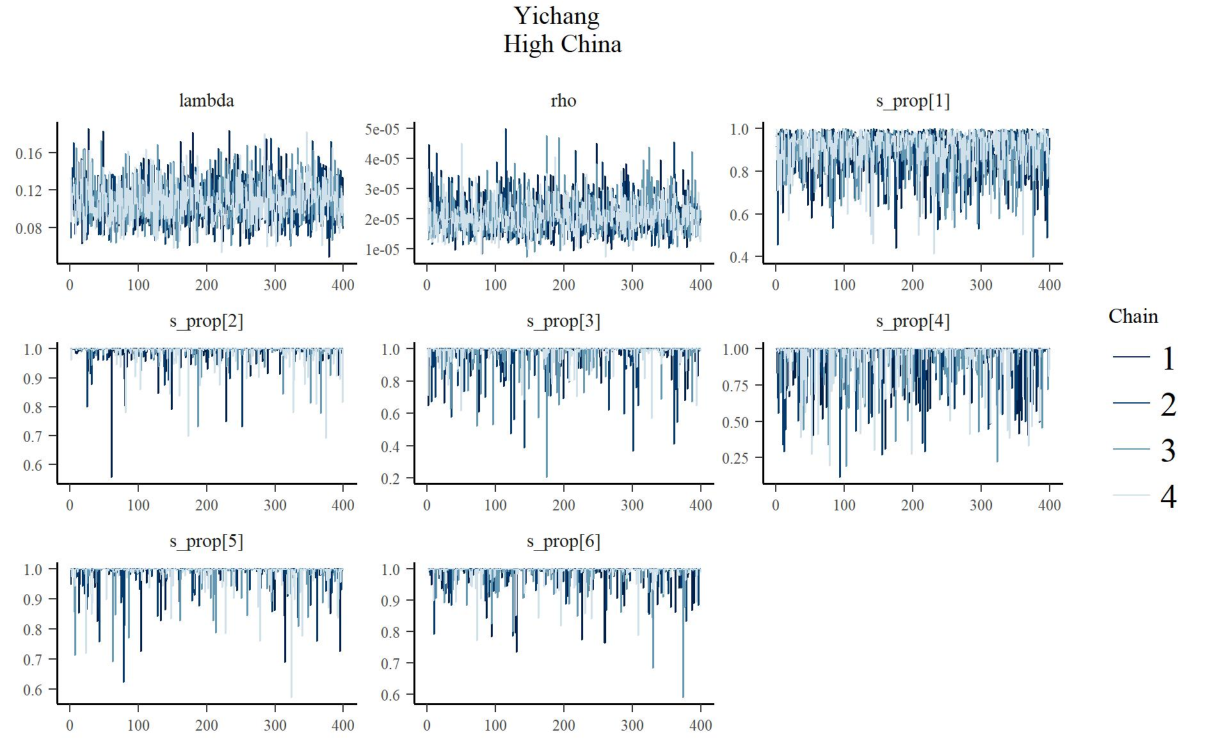
Yichang
High China
lambda
rho
s_prop[1]
s_prop[2]
s_prop[3]
s_prop[4]
s_prop[5]
s_prop[6]
Chain
1
2
3
4

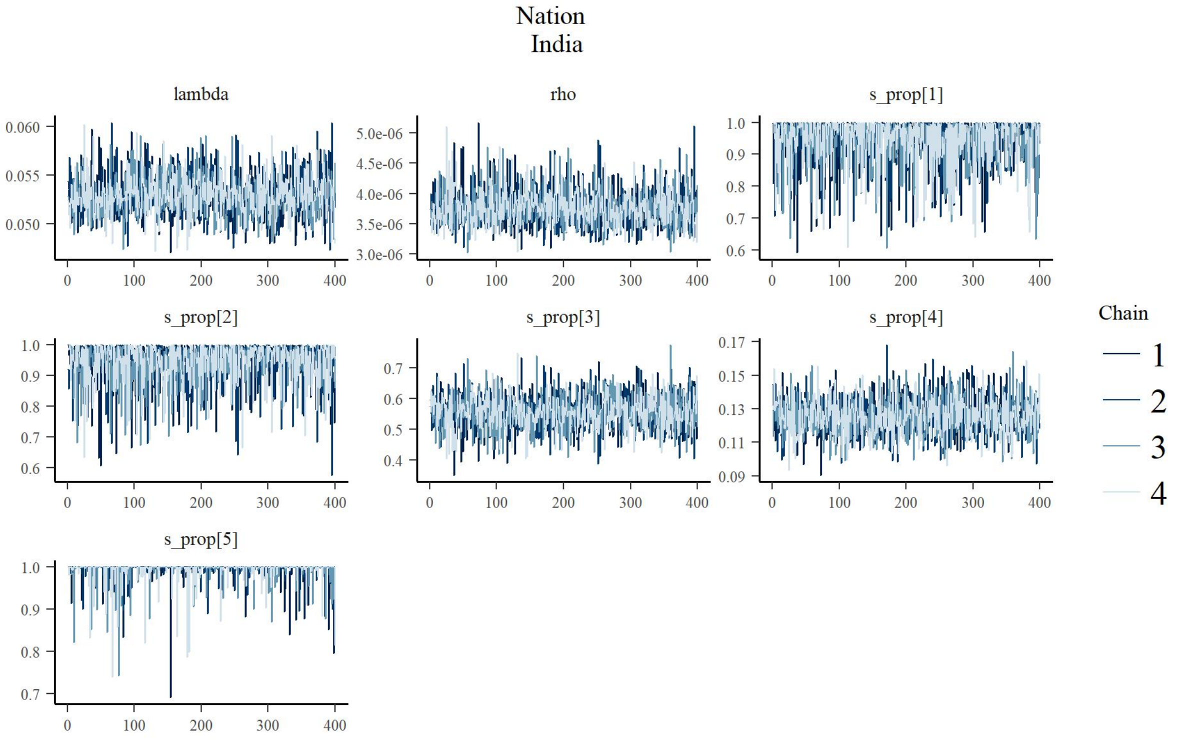

Nation
India
lambda
rho
s_prop[1]
s_prop[2]
s_prop[3]
s_prop[4]
s_prop[5]
Chain
1
2
3
4
0.060
0.055
0.050
5.0e-06
4.5e-06
4.0e-06
3.5e-06
3.0e-06
1.0
0.9
0.8
0.7
0.6
0.17
0.15
0.13
0.11
0.09
0
100
200
300
400

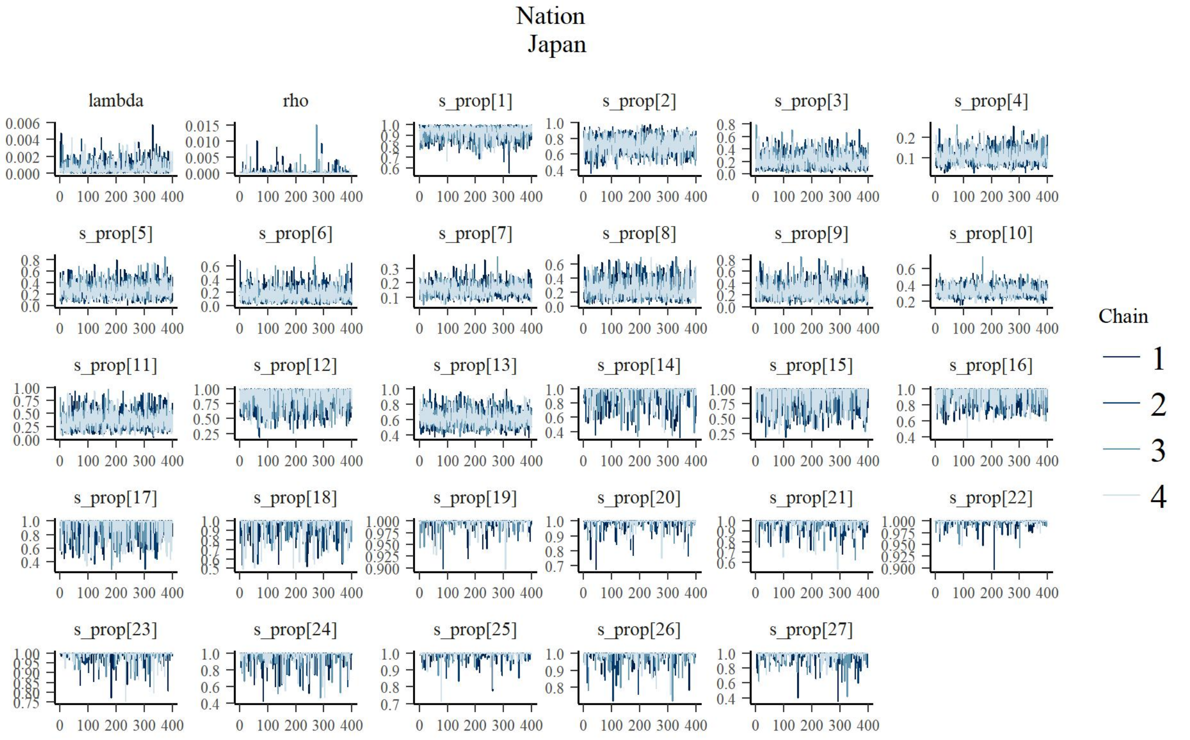
Nation
Japan
lambda
rho
s_prop[1]
s_prop[2]
s_prop[3]
s_prop[4]
s_prop[5]
s_prop[6]
s_prop[7]
s_prop[8]
s_prop[9]
s_prop[10]
s_prop[11]
s_prop[12]
s_prop[13]
s_prop[14]
s_prop[15]
s_prop[16]
s_prop[17]
s_prop[18]
s_prop[19]
s_prop[20]
s_prop[21]
s_prop[22]
s_prop[23]
s_prop[24]
s_prop[25]
s_prop[26]
s_prop[27]
Chain
1
2
3
4

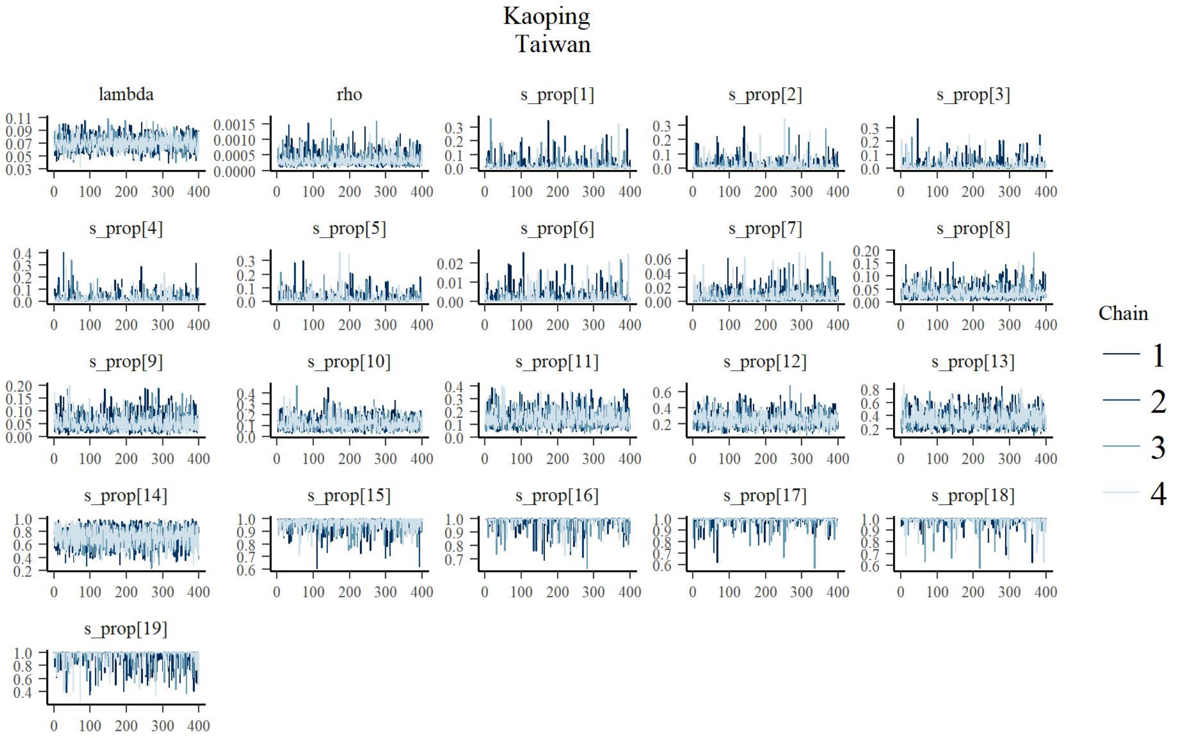

Kaoping
Taiwan
lambda
rho
s_prop[1]
s_prop[2]
s_prop[3]
s_prop[4]
s_prop[5]
s_prop[6]
s_prop[7]
s_prop[8]
s_prop[9]
s_prop[10]
s_prop[11]
s_prop[12]
s_prop[13]
s_prop[14]
s_prop[15]
s_prop[16]
s_prop[17]
s_prop[18]
s_prop[19]
Chain
1
2
3
4

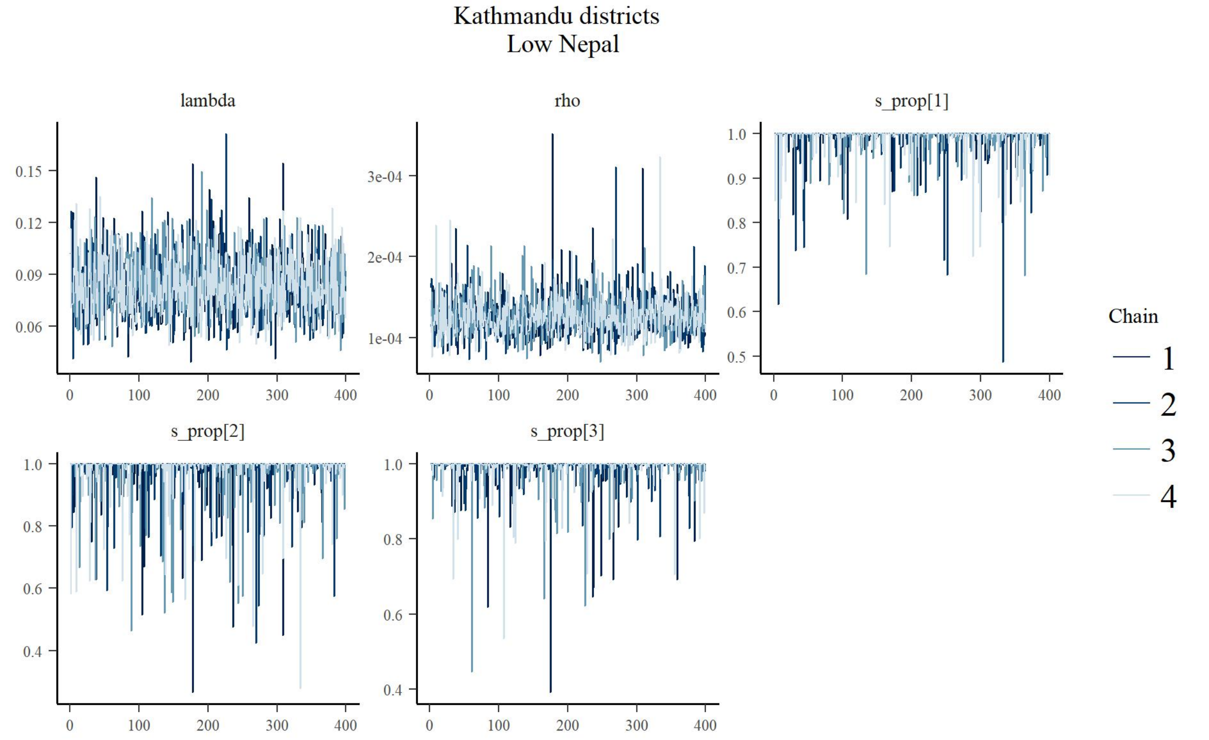
Kathmandu districts
Low Nepal
lambda
rho
s_prop[1]
s_prop[2]
s_prop[3]
Chain
1
2
3
4
0.15
0.12
0.09
0.06
3e-04
2e-04
1e-04
1.0
0.9
0.8
0.7
0.6
0.5
0.4
0
100
200
300
400

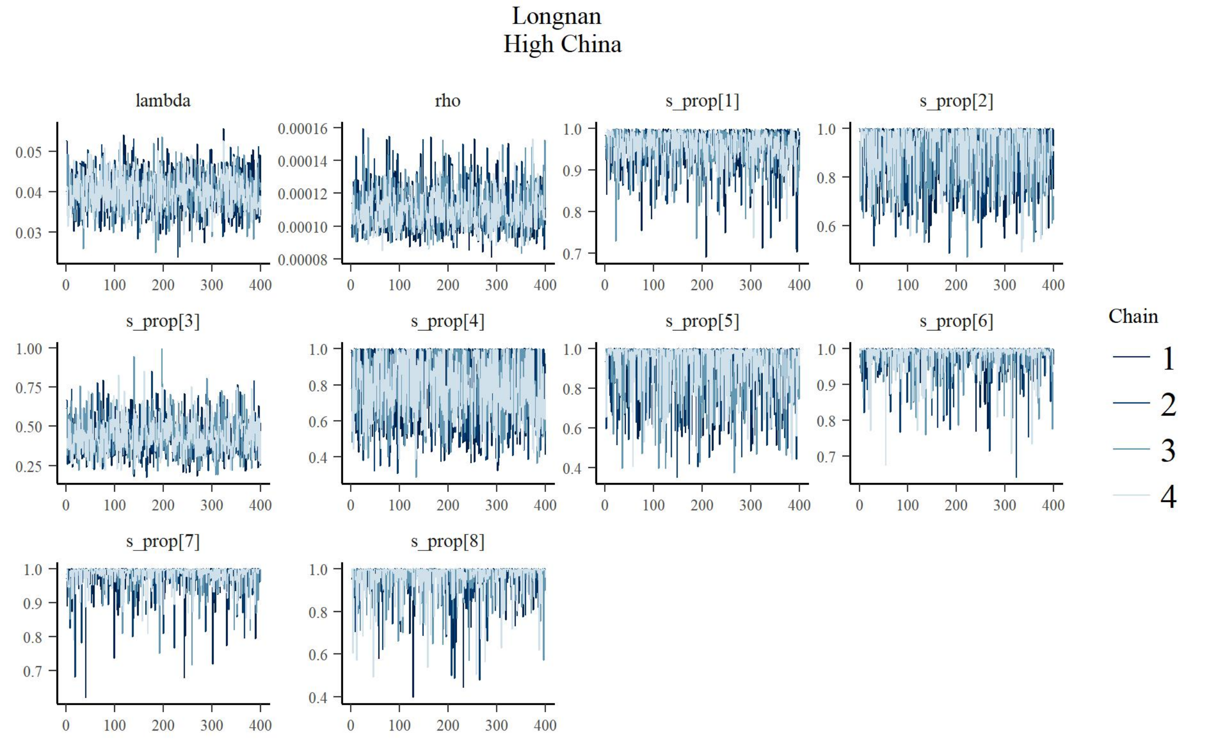
Longnan
High China
lambda
rho
s_prop[1]
s_prop[2]
s_prop[3]
s_prop[4]
s_prop[5]
s_prop[6]
s_prop[7]
s_prop[8]
Chain
1
2
3
4

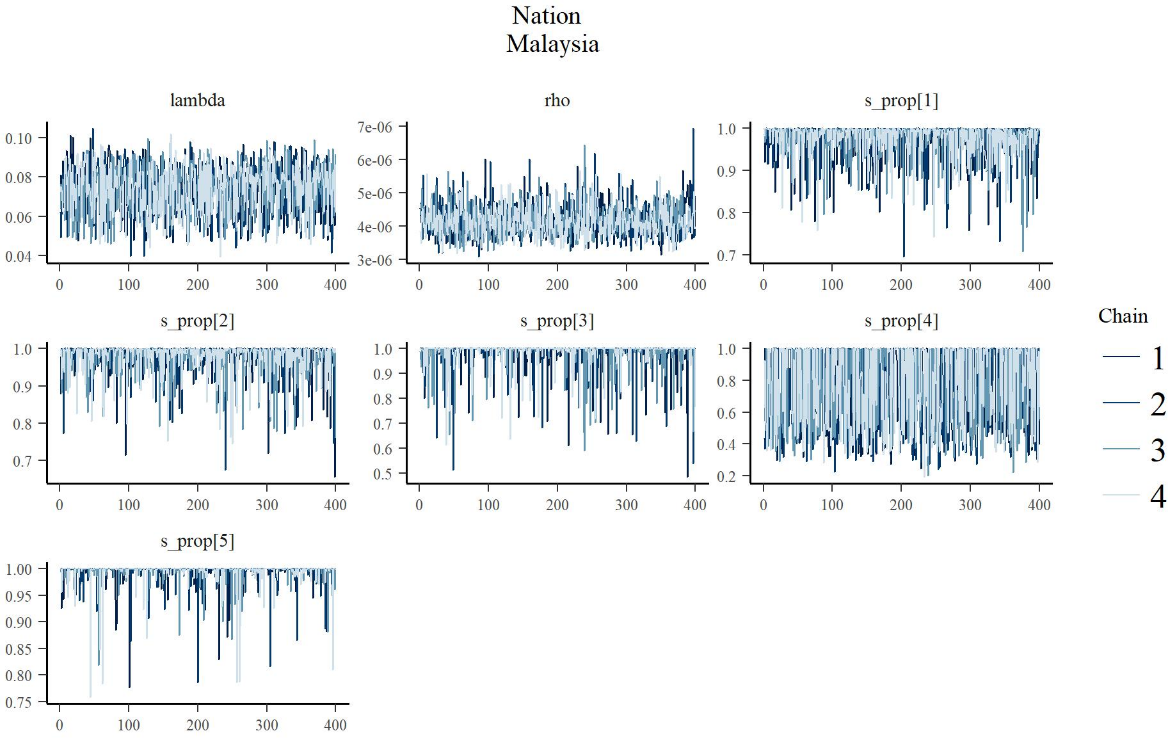

Nation
Malaysia
lambda
rho
s_prop[1]
s_prop[2]
s_prop[3]
s_prop[4]
s_prop[5]
Chain
1
2
3
4
0.10
0.08
0.06
0.04
7e-06
6e-06
5e-06
4e-06
3e-06
1.0
0.9
0.8
0.7
0.6
0.5
0.4
0.2
1.00
0.95
0.90
0.85
0.80
0.75
0
100
200
300
400

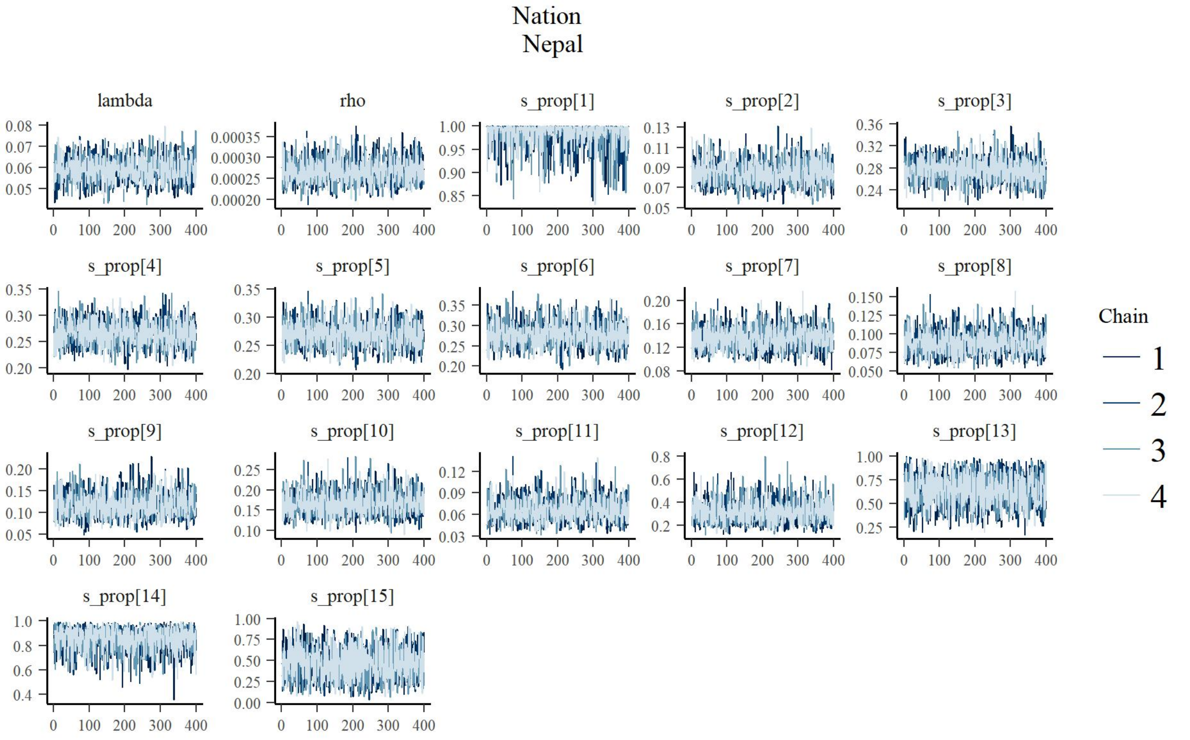
Nation
Nepal
lambda
rho
s_prop[1]
s_prop[2]
s_prop[3]
s_prop[4]
s_prop[5]
s_prop[6]
s_prop[7]
s_prop[8]
s_prop[9]
s_prop[10]
s_prop[11]
s_prop[12]
s_prop[13]
s_prop[14]
s_prop[15]
Chain
1
2
3
4

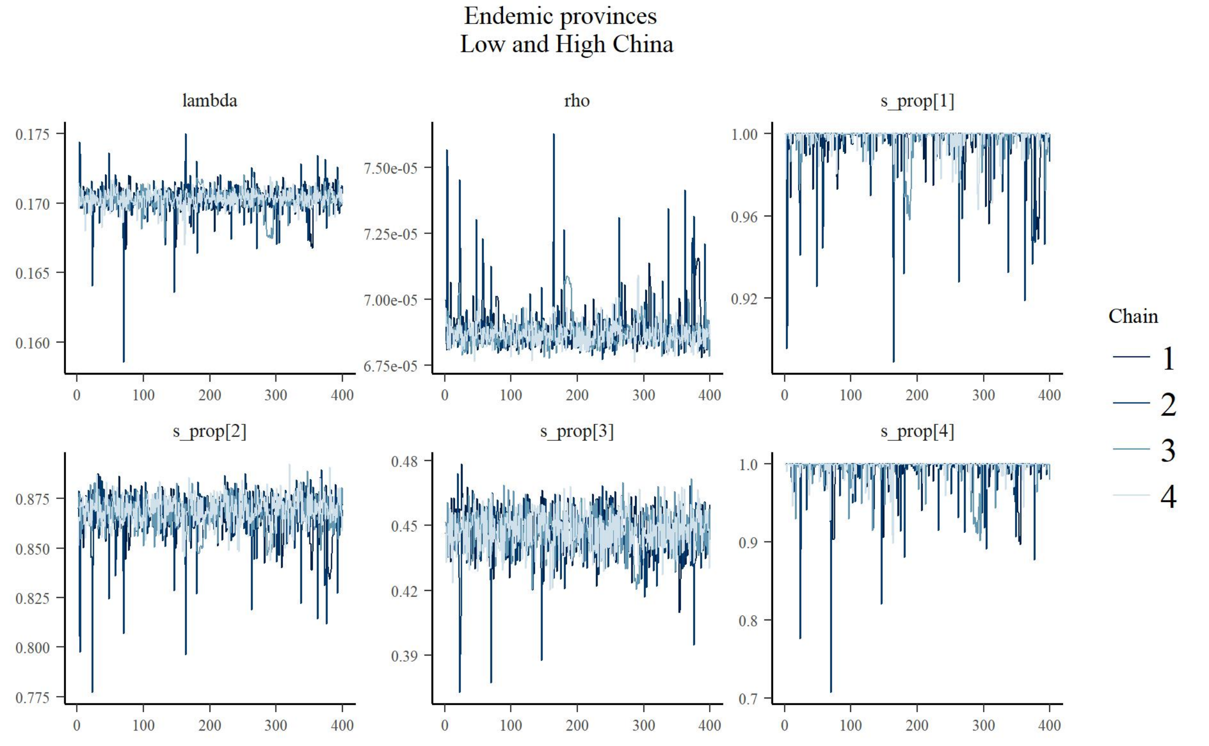

Endemic provinces
Low and High China
lambda
rho
s_prop[1]
s_prop[2]
s_prop[3]
s_prop[4]
Chain
1
2
3
4
0.175
0.170
0.165
0.160
7.50e-05
7.25e-05
7.00e-05
6.75e-05
1.00
0.96
0.92
0.875
0.850
0.825
0.800
0.775
0.48
0.45
0.42
0.39
1.0
0.9
0.8
0.7
0
100
200
300
400

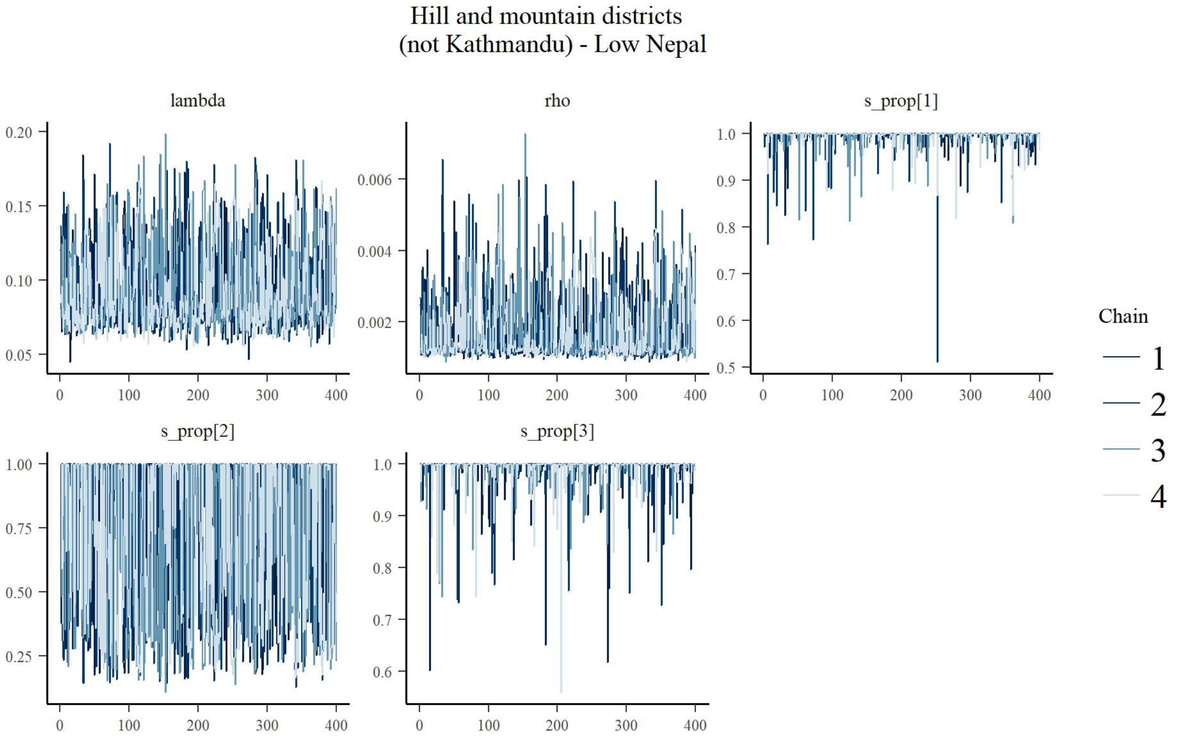

Hill and mountain districts
(not Kathmandu) - Low Nepal
lambda
rho
s_prop[1]
s_prop[2]
s_prop[3]
0.20
0.15
0.10
0.05
0.006
0.004
0.002
1.0
0.9
0.8
0.7
0.6
0.5
1.00
0.75
0.50
0.25
0
100
200
300
400
Chain
1
2
3
4

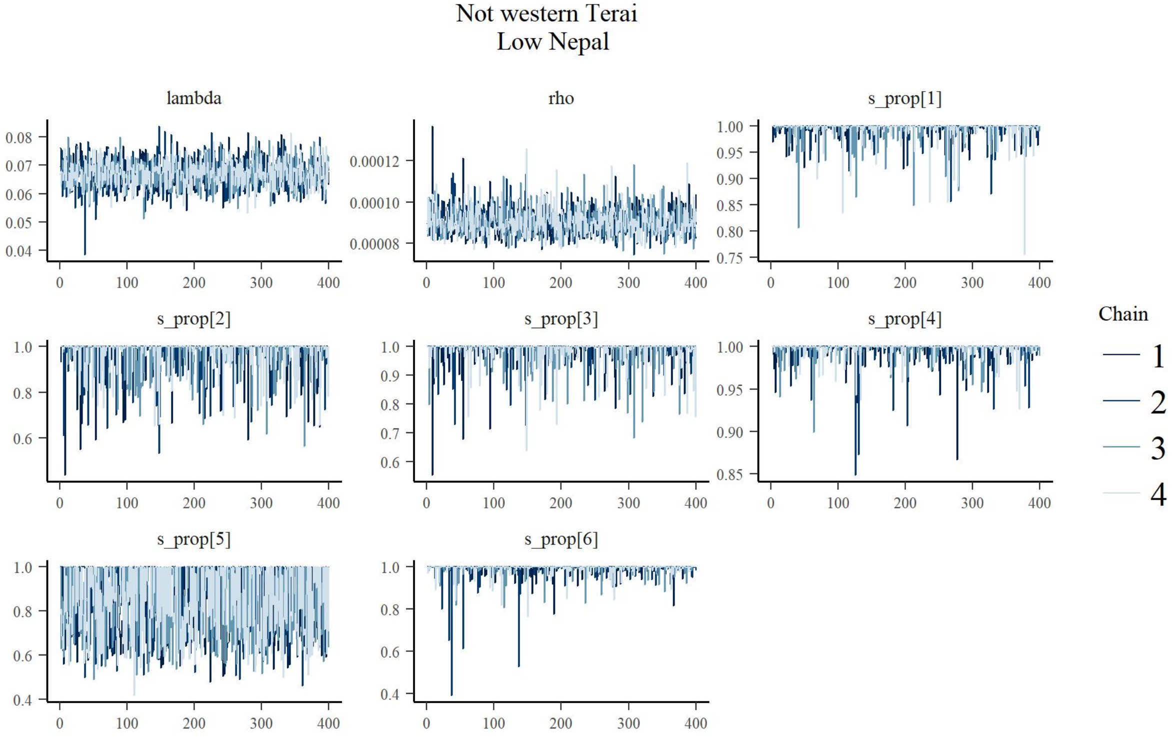
Not western Terai
Low Nepal
lambda
rho
s_prop[1]
s_prop[2]
s_prop[3]
s_prop[4]
s_prop[5]
s_prop[6]
Chain
1
2
3
4

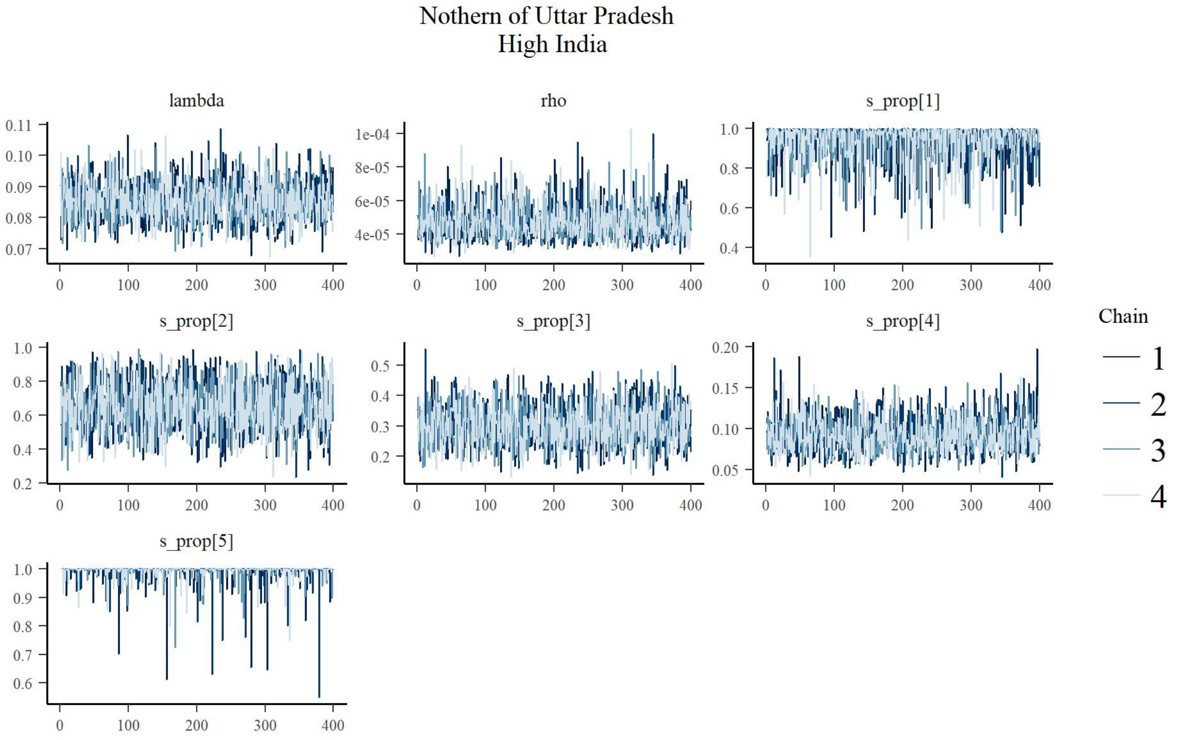

Nothern of Uttar Pradesh
High India
lambda
rho
s_prop[1]
s_prop[2]
s_prop[3]
s_prop[4]
s_prop[5]
Chain
1
2
3
4
0.11
0.10
0.09
0.08
0.07
1e-04
8e-05
6e-05
4e-05
1.0
0.8
0.6
0.4
0.2
0.5
0.3
0.20
0.15
0.10
0.05
0.9
0.7
0 100 200 300 400

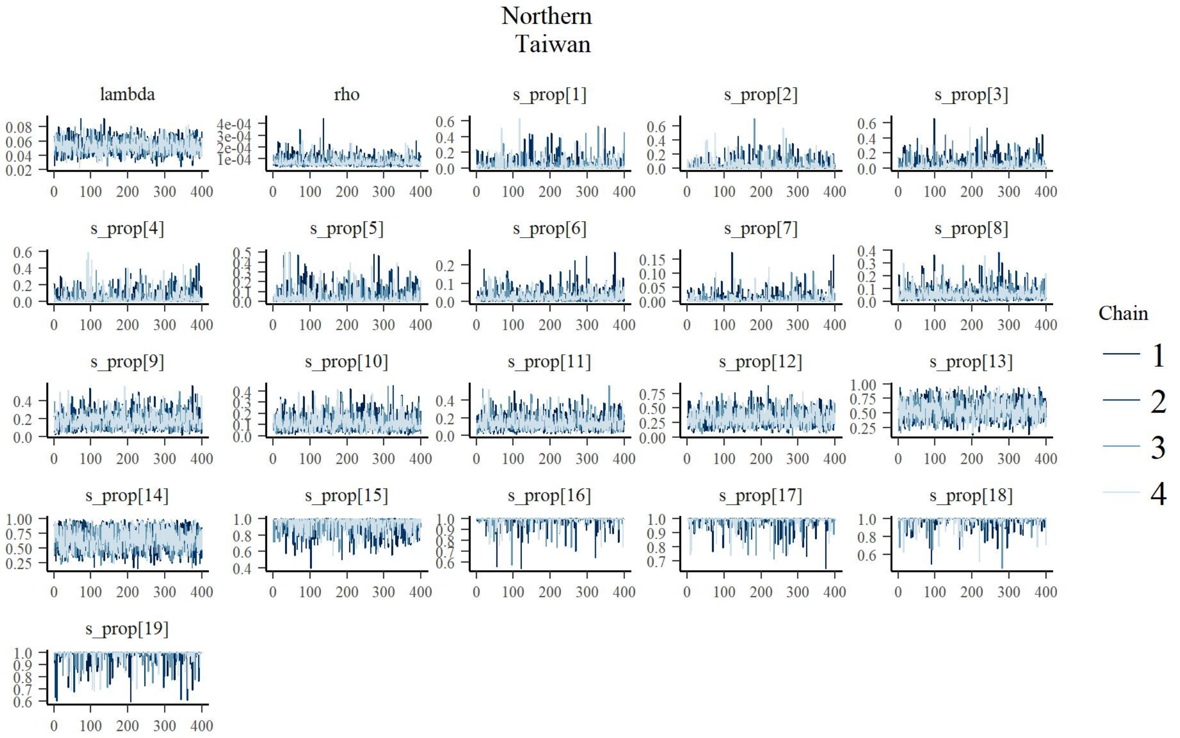

Northern
Taiwan
lambda
rho
s_prop[1]
s_prop[2]
s_prop[3]
s_prop[4]
s_prop[5]
s_prop[6]
s_prop[7]
s_prop[8]
s_prop[9]
s_prop[10]
s_prop[11]
s_prop[12]
s_prop[13]
s_prop[14]
s_prop[15]
s_prop[16]
s_prop[17]
s_prop[18]
s_prop[19]
Chain
1
2
3
4

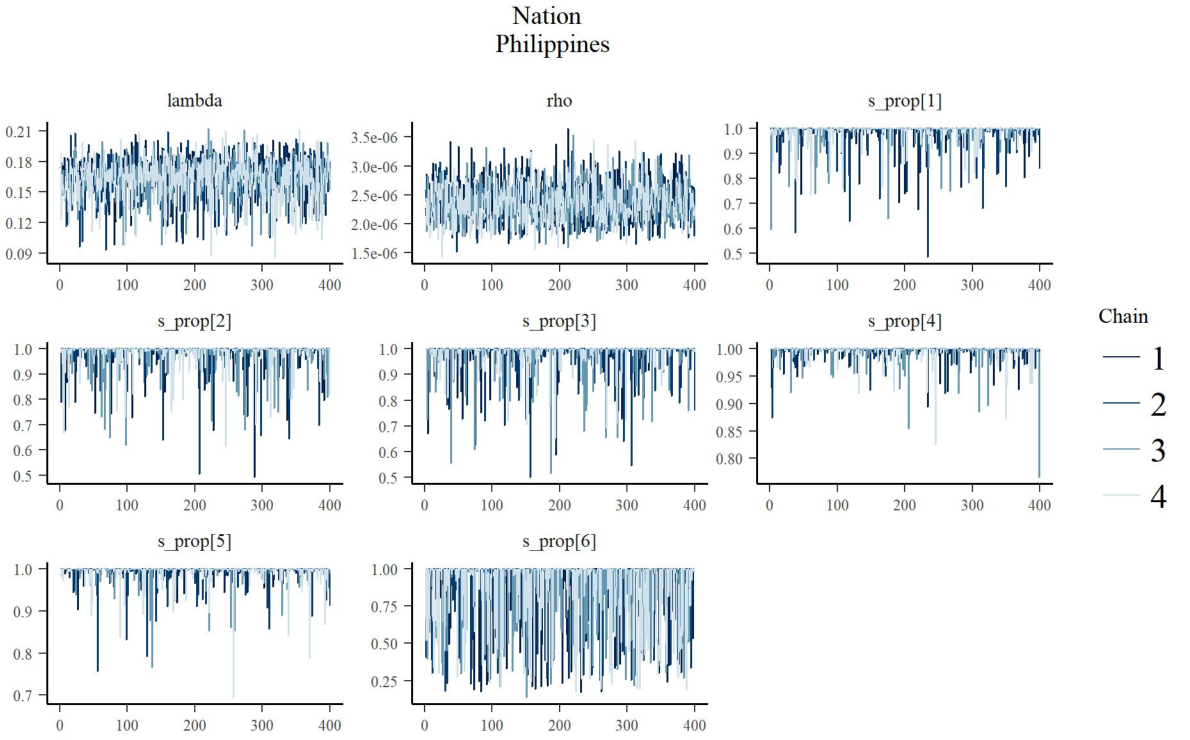

Nation
Philippines
lambda
rho
s_prop[1]
s_prop[2]
s_prop[3]
s_prop[4]
s_prop[5]
s_prop[6]
Chain
1
2
3
4

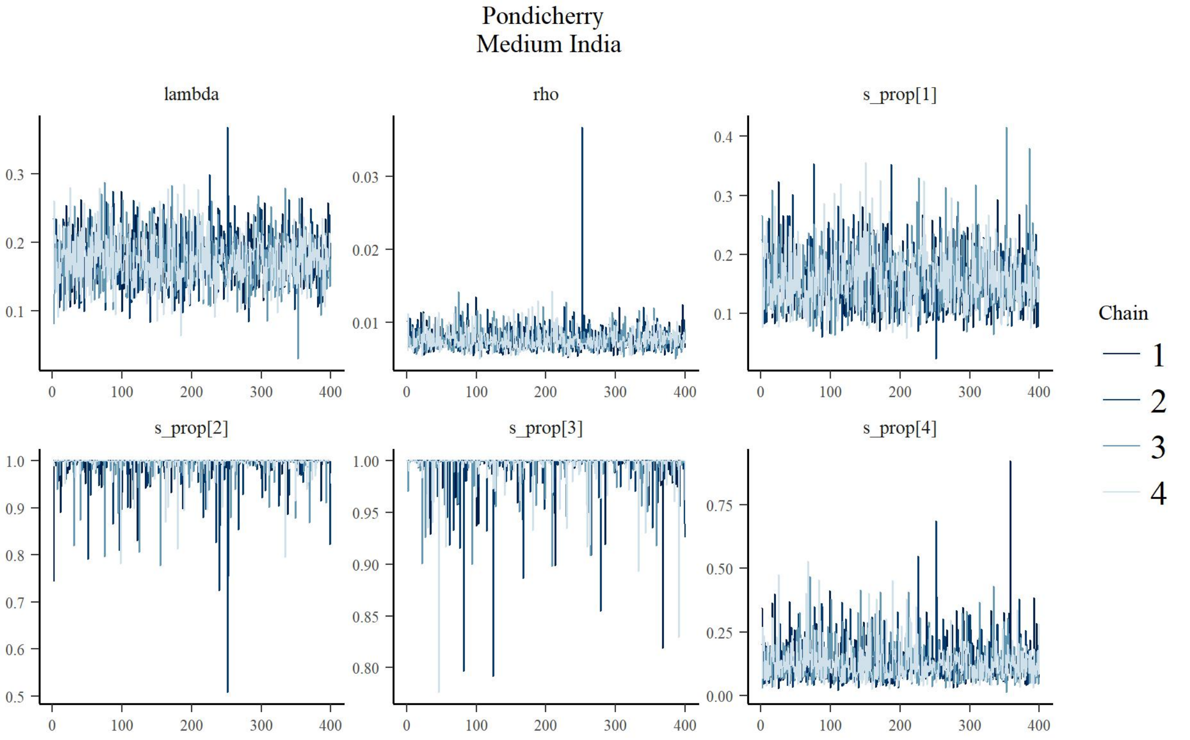

Pondicherry
Medium India
lambda
rho
s_prop[1]
s_prop[2]
s_prop[3]
s_prop[4]
Chain
1
2
3
4

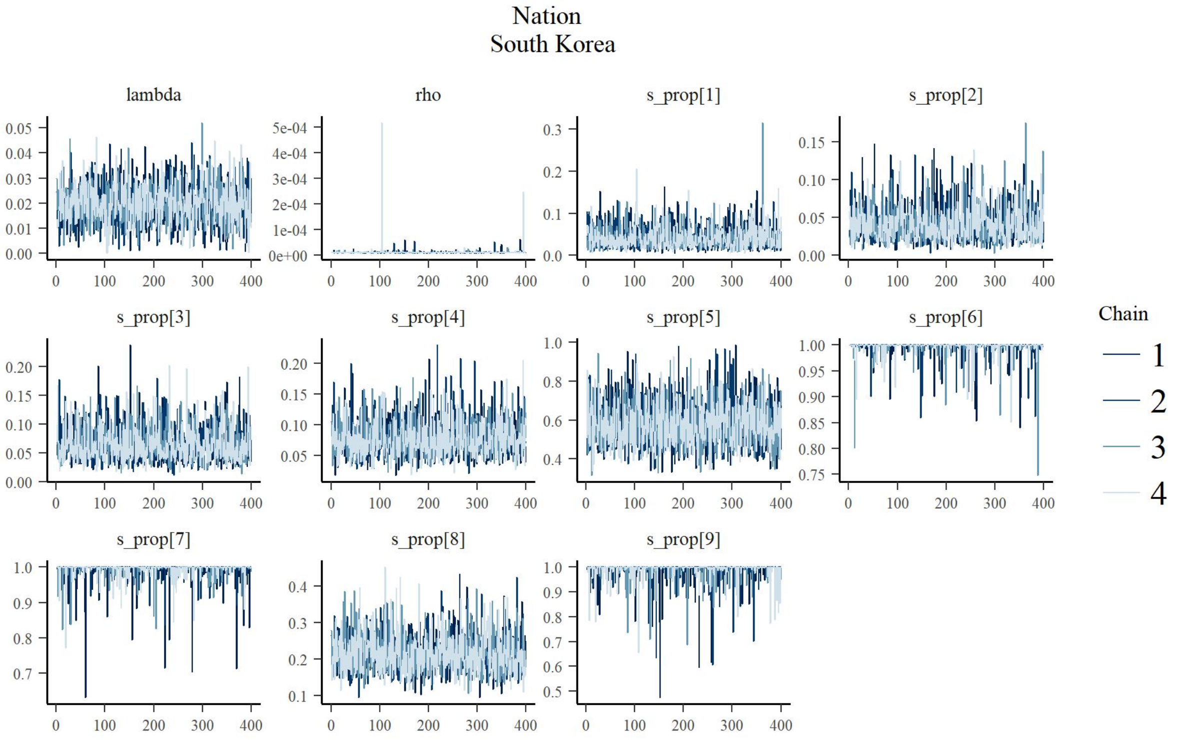

Nation
South Korea
lambda
rho
s_prop[1]
s_prop[2]
s_prop[3]
s_prop[4]
s_prop[5]
s_prop[6]
s_prop[7]
s_prop[8]
s_prop[9]
Chain
1
2
3
4

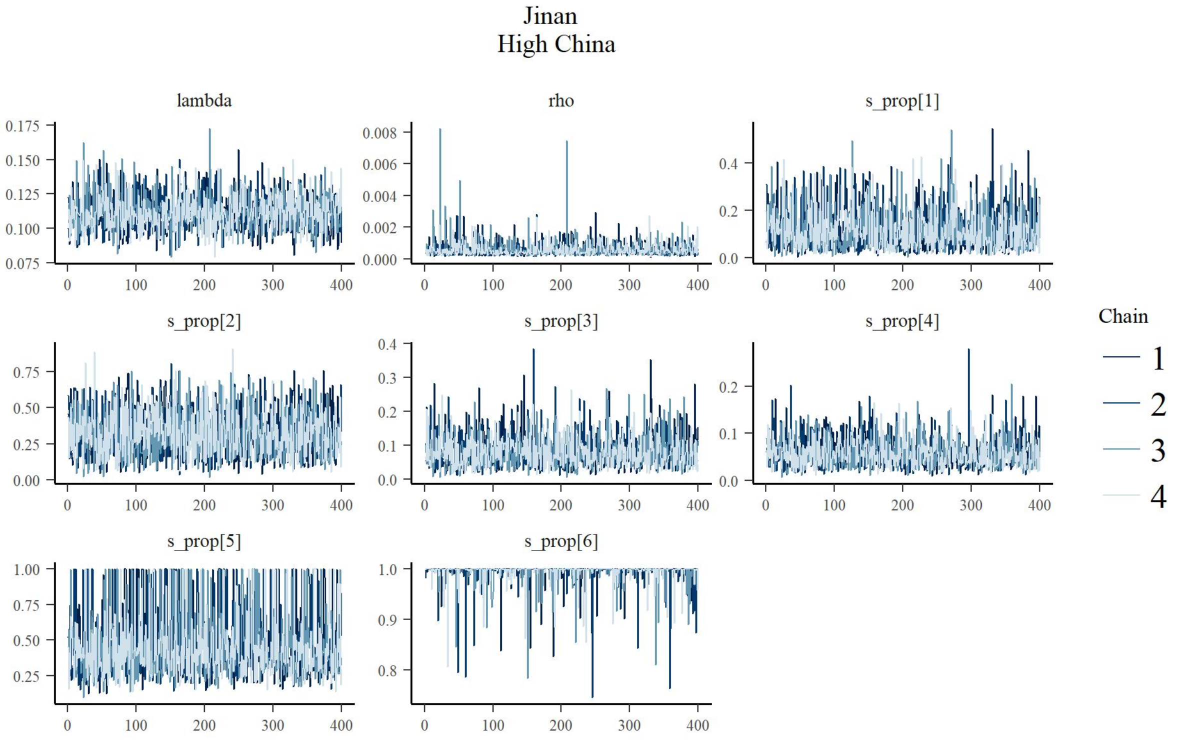
Jinan
High China
lambda
rho
s_prop[1]
s_prop[2]
s_prop[3]
s_prop[4]
s_prop[5]
s_prop[6]
0.175
0.150
0.125
0.100
0.075
0.008
0.006
0.004
0.002
0.000
0.4
0.2
0.0
0.75
0.50
0.25
0.00
0.3
0.1
1.00
1.0
0.9
0.8
0
100
200
300
400
Chain
1
2
3
4

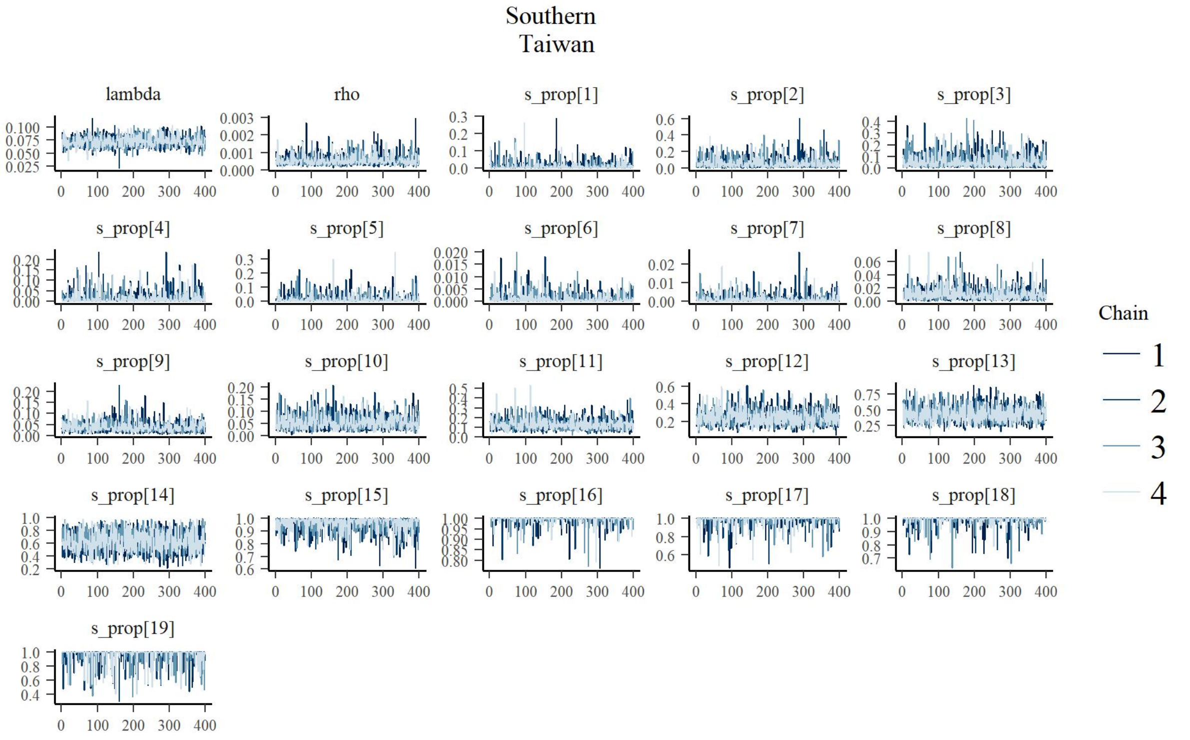
Southern
Taiwan
lambda
rho
s_prop[1]
s_prop[2]
s_prop[3]
s_prop[4]
s_prop[5]
s_prop[6]
s_prop[7]
s_prop[8]
s_prop[9]
s_prop[10]
s_prop[11]
s_prop[12]
s_prop[13]
s_prop[14]
s_prop[15]
s_prop[16]
s_prop[17]
s_prop[18]
s_prop[19]
Chain
1
2
3
4

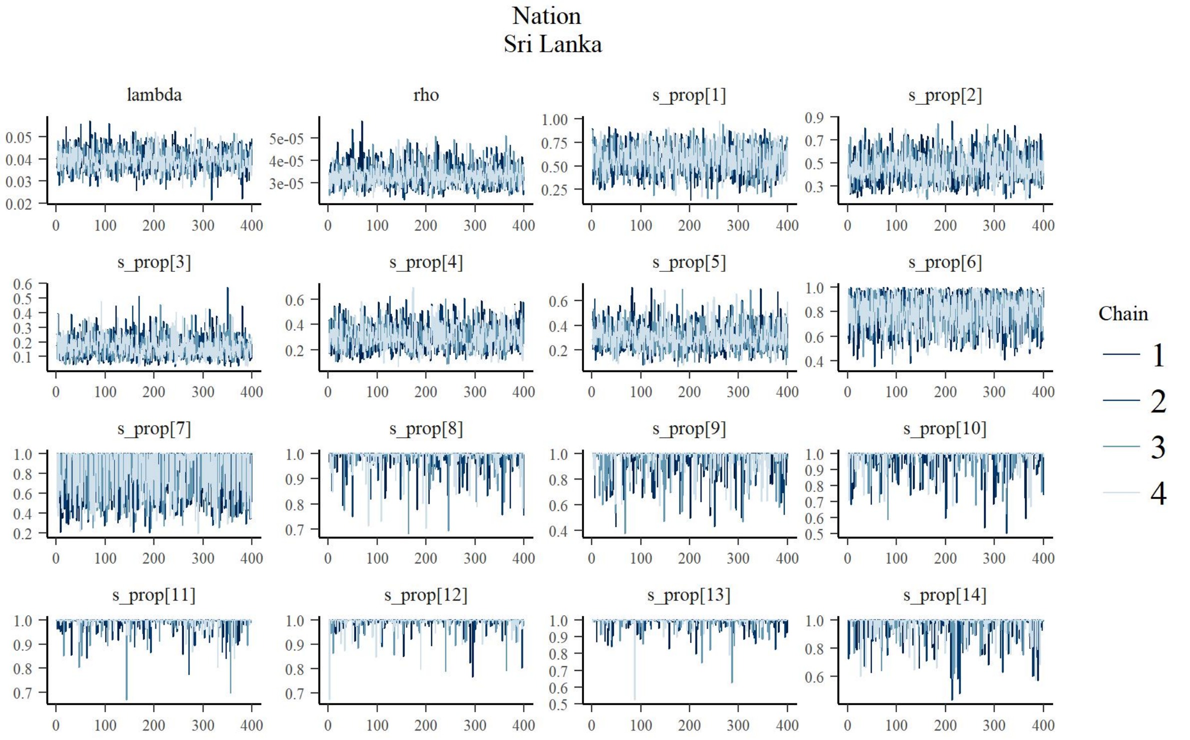
Nation
Sri Lanka
lambda
rho
s_prop[1]
s_prop[2]
s_prop[3]
s_prop[4]
s_prop[5]
s_prop[6]
s_prop[7]
s_prop[8]
s_prop[9]
s_prop[10]
s_prop[11]
s_prop[12]
s_prop[13]
s_prop[14]
Chain
1
2
3
4

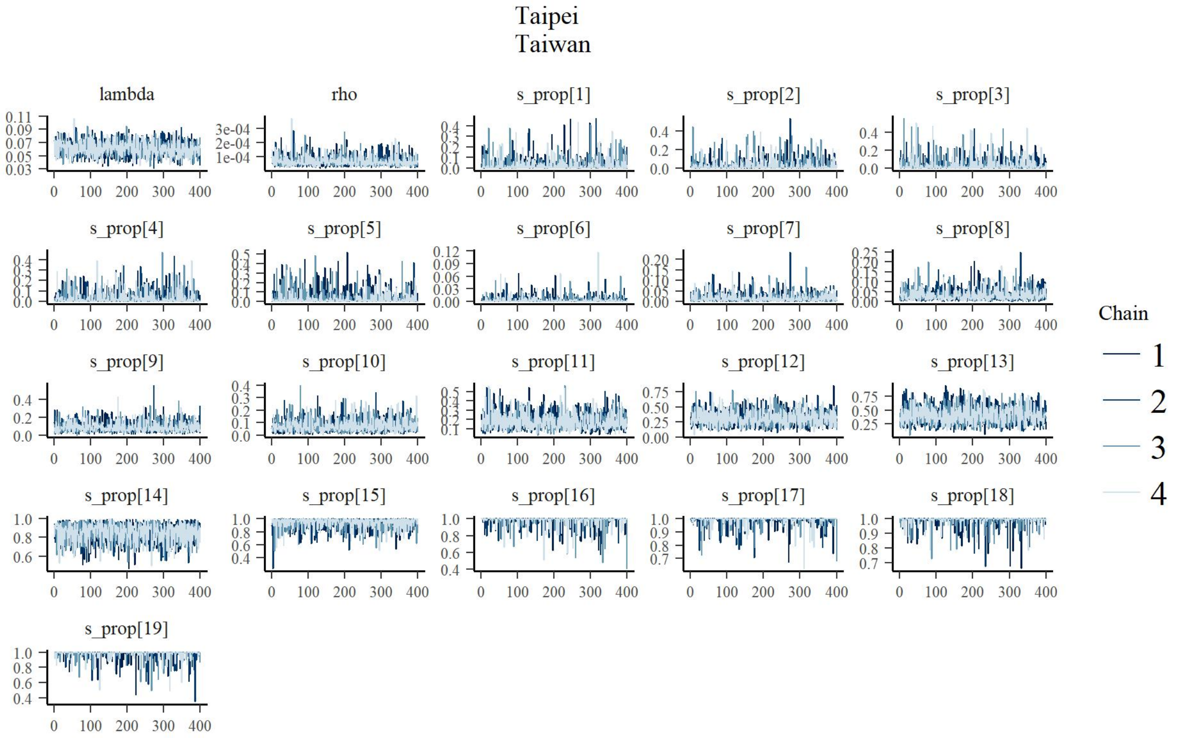

Taipei
Taiwan
lambda
rho
s_prop[1]
s_prop[2]
s_prop[3]
s_prop[4]
s_prop[5]
s_prop[6]
s_prop[7]
s_prop[8]
s_prop[9]
s_prop[10]
s_prop[11]
s_prop[12]
s_prop[13]
s_prop[14]
s_prop[15]
s_prop[16]
s_prop[17]
s_prop[18]
s_prop[19]
Chain
1
2
3
4

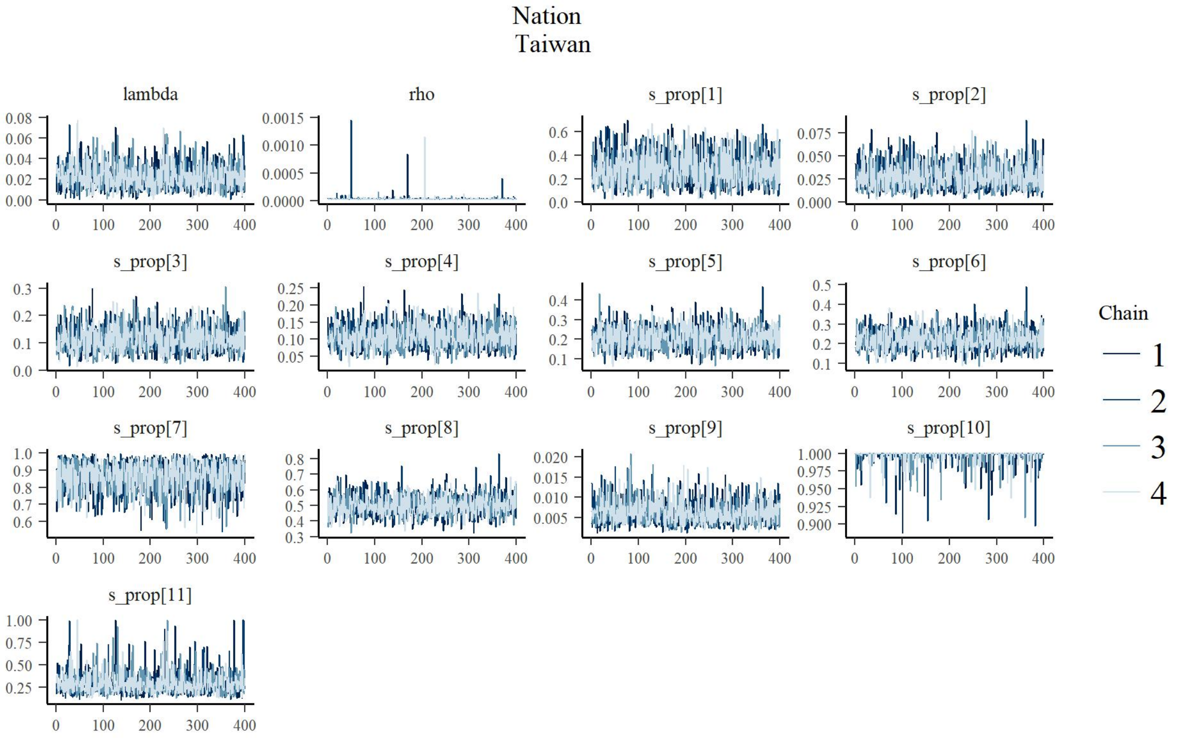
Nation
Taiwan
lambda
rho
s_prop[1]
s_prop[2]
s_prop[3]
s_prop[4]
s_prop[5]
s_prop[6]
s_prop[7]
s_prop[8]
s_prop[9]
s_prop[10]
s_prop[11]
Chain
1
2
3
4

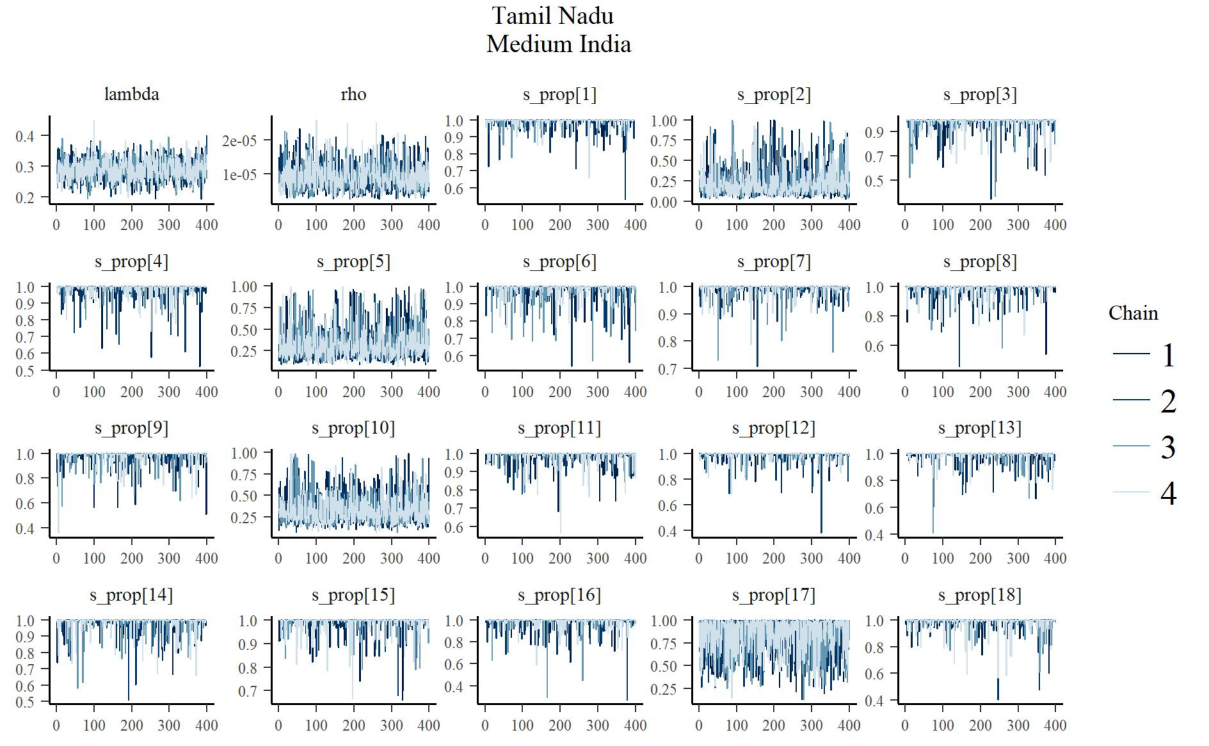

Tamil Nadu
Medium India
lambda
rho
s_prop[1]
s_prop[2]
s_prop[3]
s_prop[4]
s_prop[5]
s_prop[6]
s_prop[7]
s_prop[8]
s_prop[9]
s_prop[10]
s_prop[11]
s_prop[12]
s_prop[13]
s_prop[14]
s_prop[15]
s_prop[16]
s_prop[17]
s_prop[18]
0 100 200 300 400
Chain
1
2
3
4

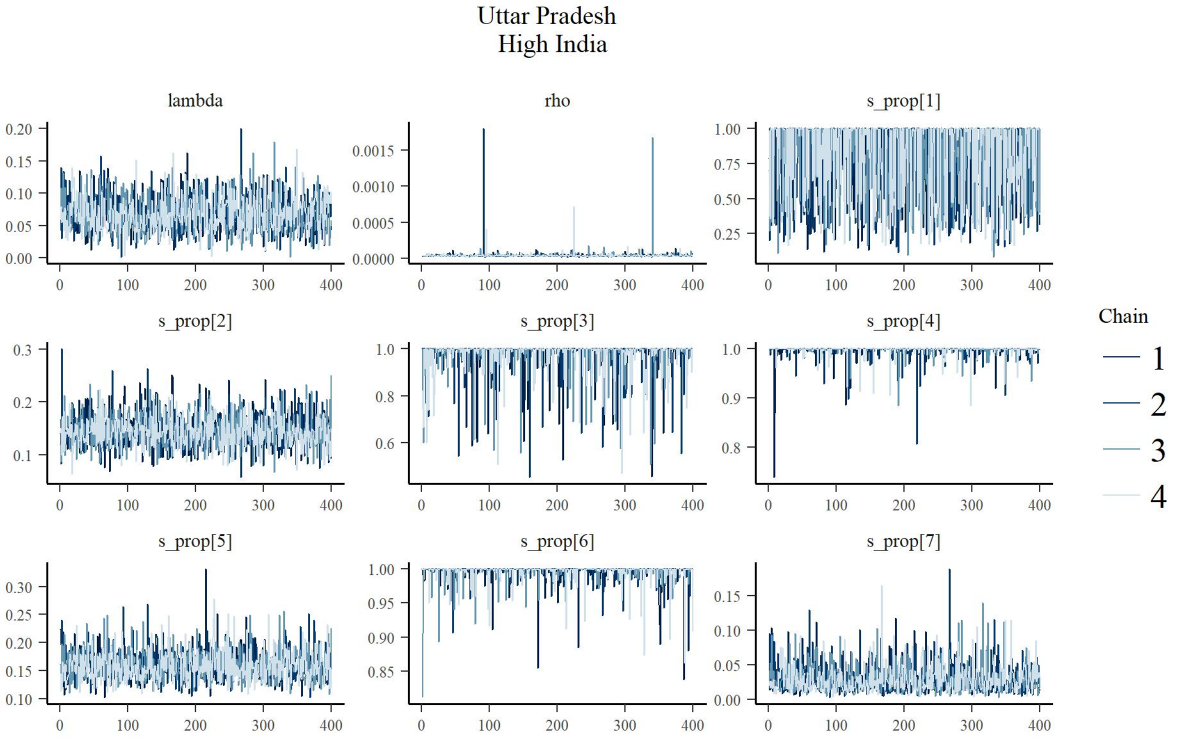

Uttar Pradesh
High India
lambda
rho
s_prop[1]
s_prop[2]
s_prop[3]
s_prop[4]
s_prop[5]
s_prop[6]
s_prop[7]
Chain
1
2
3
4

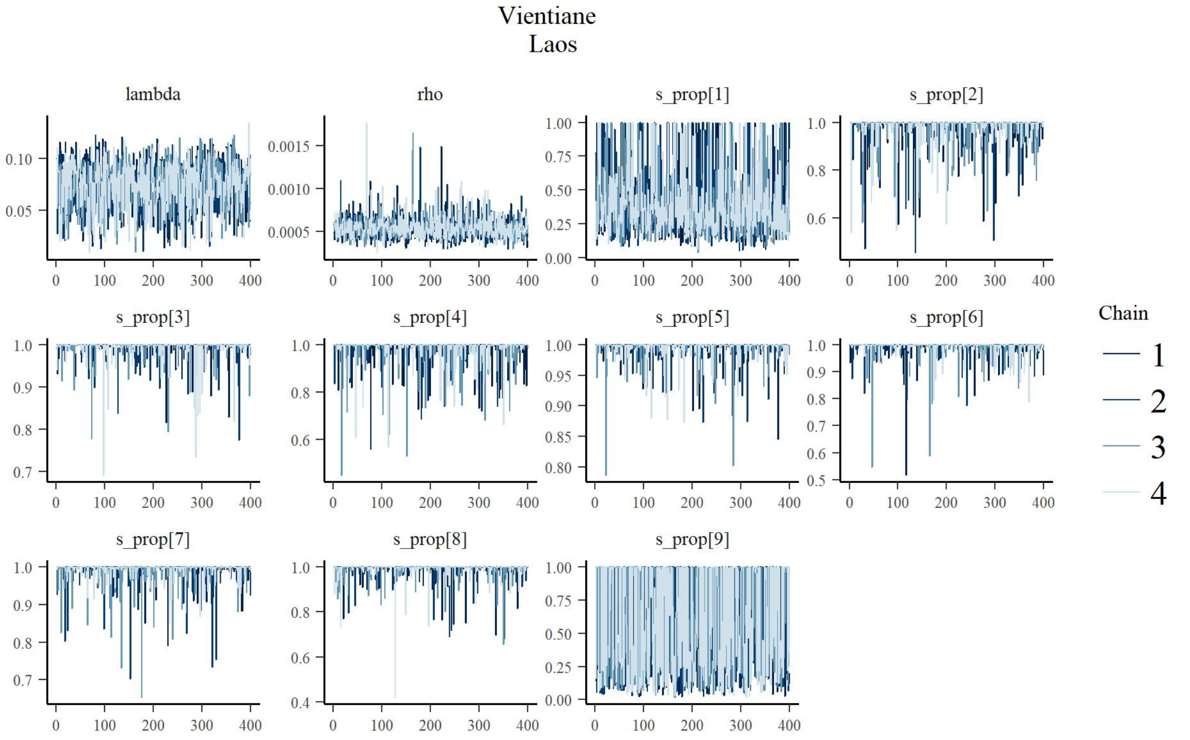

Vientiane
Laos
lambda
rho
s_prop[1]
s_prop[2]
s_prop[3]
s_prop[4]
s_prop[5]
s_prop[6]
s_prop[7]
s_prop[8]
s_prop[9]
Chain
1
2
3
4

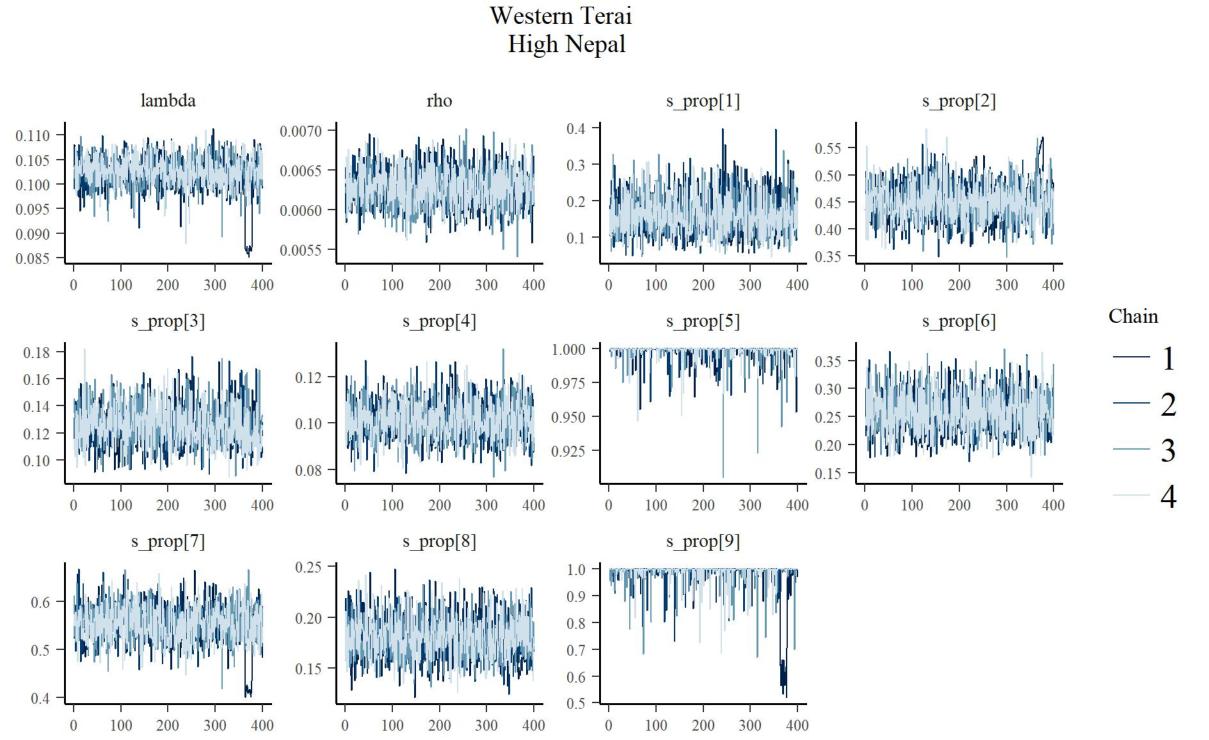
Western Terai
High Nepal
lambda
rho
s_prop[1]
s_prop[2]
s_prop[3]
s_prop[4]
s_prop[5]
s_prop[6]
s_prop[7]
s_prop[8]
s_prop[9]
Chain
1
2
3
4

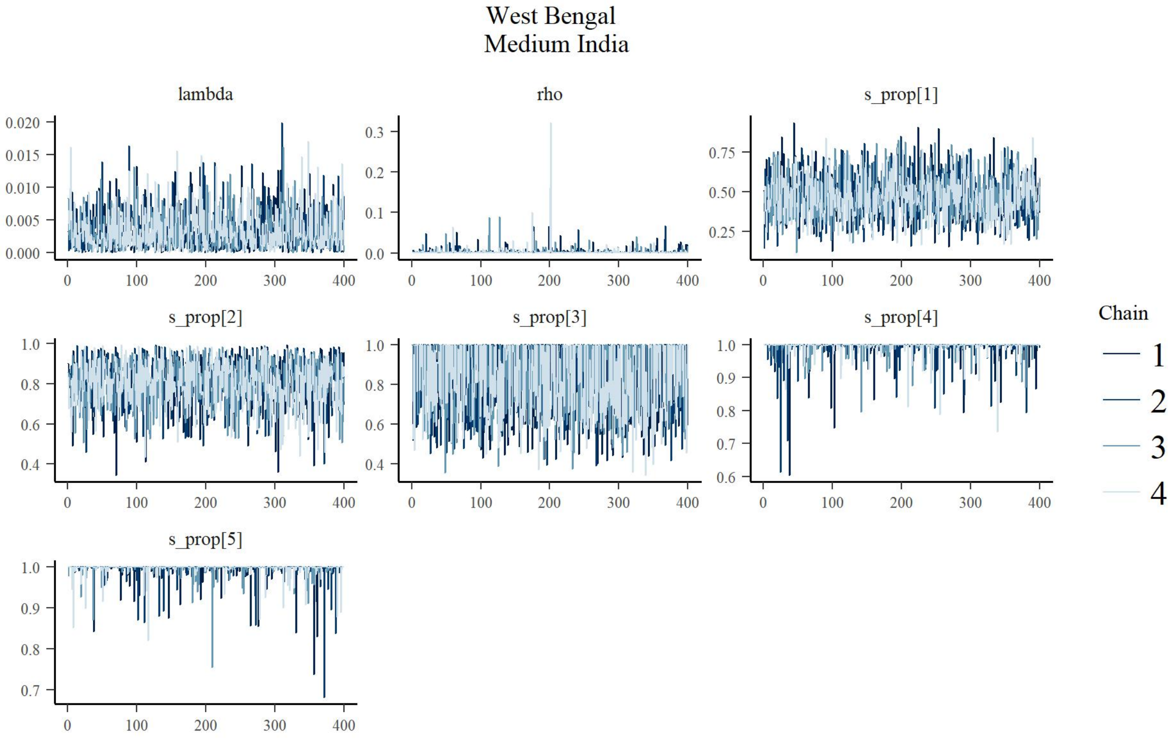
West Bengal
Medium India
lambda
rho
s_prop[1]
s_prop[2]
s_prop[3]
s_prop[4]
s_prop[5]
Chain
1
2
3
4
