# Supplementary material for: Estimates of the global burden of Japanese encephalitis and the impact of vaccination from 2000-2015
Source: eLife. 2020 May 26;9:e51027. doi: 10.7554/eLife.51027 (PMC7282807; doi:10.7554/eLife.51027)
Supplement: Figure 4—source data 5. [file elife-51027-fig4-data5.pdf]

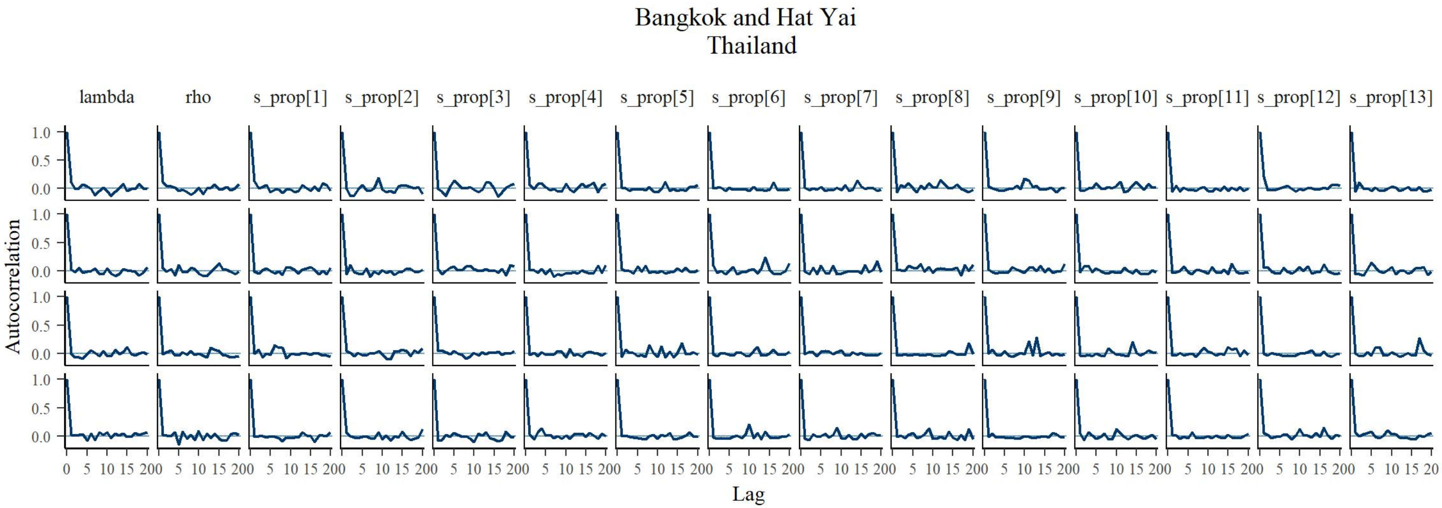

Bangkok and Hat Yai
Thailand
lambda
rho
s_prop[1]
s_prop[2]
s_prop[3]
s_prop[4]
s_prop[5]
s_prop[6]
s_prop[7]
s_prop[8]
s_prop[9]
s_prop[10]
s_prop[11]
s_prop[12]
s_prop[13]
Autocorrelation
Lag

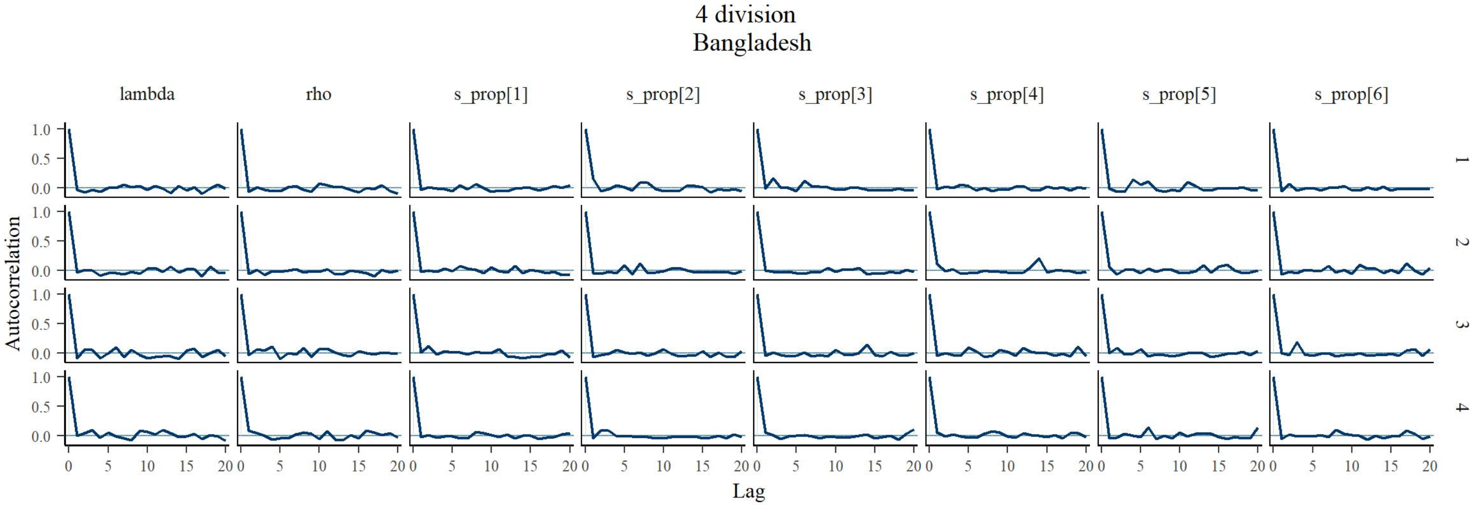

4 division
Bangladesh
lambda
rho
s_prop[1]
s_prop[2]
s_prop[3]
s_prop[4]
s_prop[5]
s_prop[6]
Autocorrelation
Lag
1
2
3
4

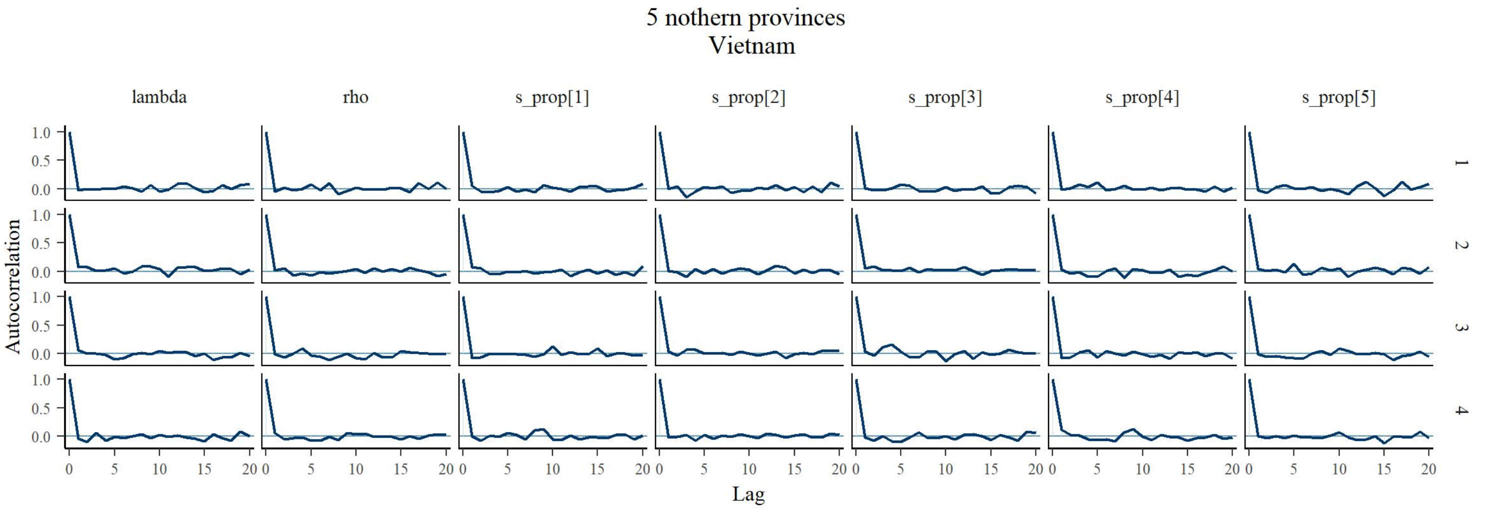

5 nothern provinces
Vietnam
lambda
rho
s_prop[1]
s_prop[2]
s_prop[3]
s_prop[4]
s_prop[5]
Autocorrelation
Lag
1
2
3
4

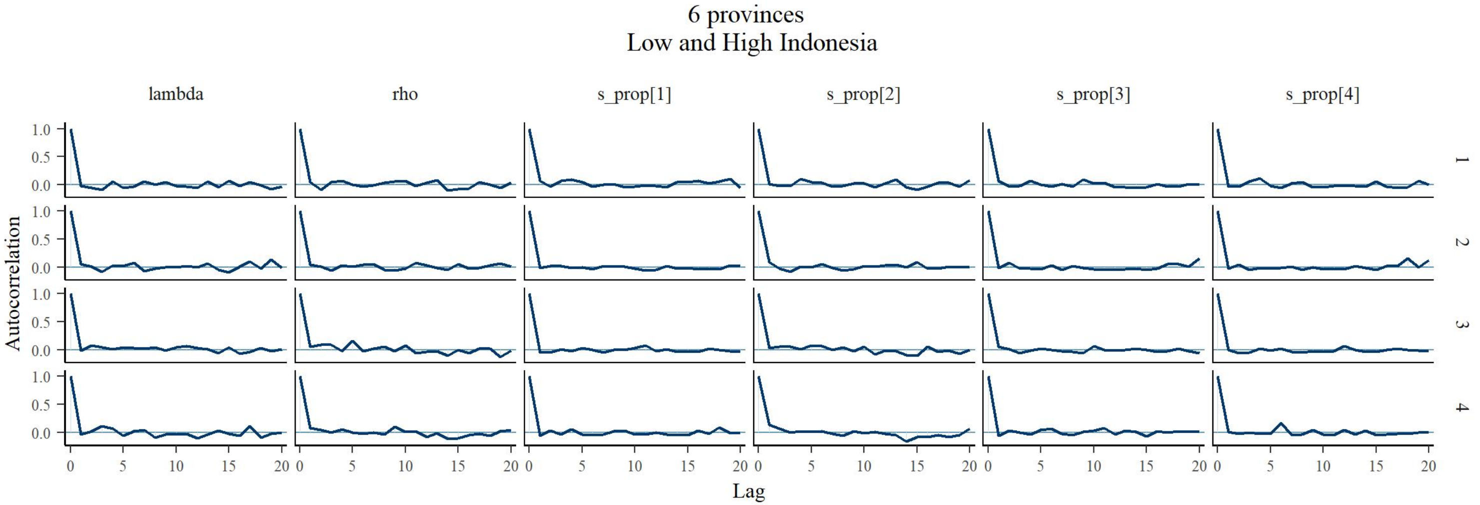

6 provinces
Low and High Indonesia
lambda
rho
s_prop[1]
s_prop[2]
s_prop[3]
s_prop[4]
1
2
3
4
Autocorrelation
Lag

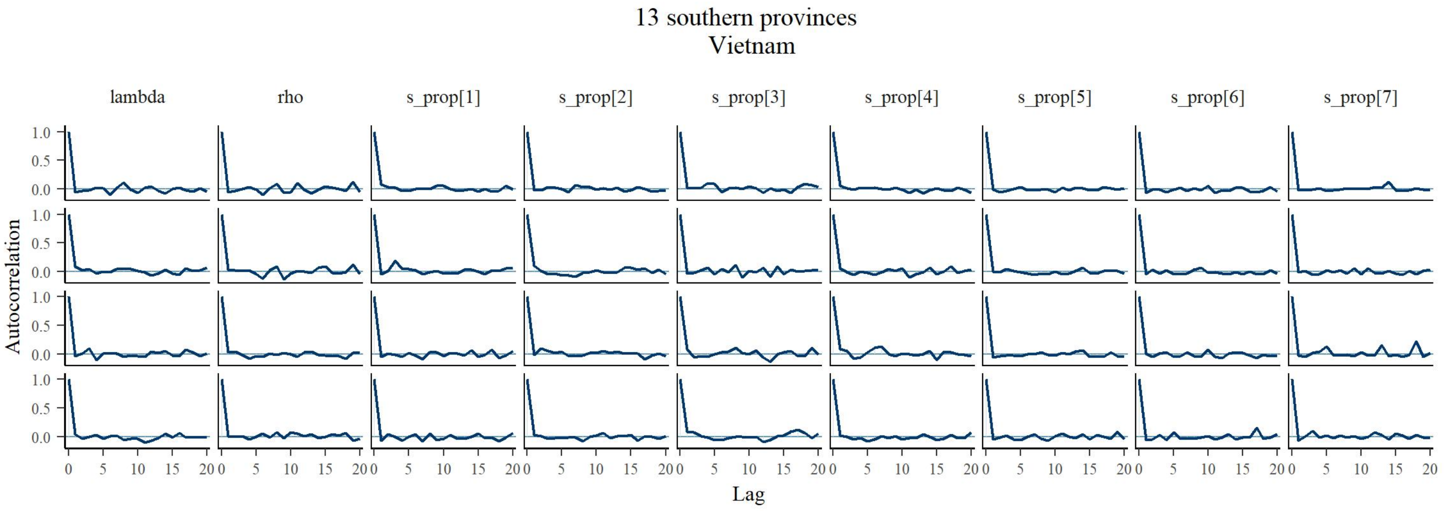
13 southern provinces
Vietnam
lambda
rho
s_prop[1]
s_prop[2]
s_prop[3]
s_prop[4]
s_prop[5]
s_prop[6]
s_prop[7]
1
2
3
4
Autocorrelation
1.0
0.5
0.0
0
5
10
15
20
Lag

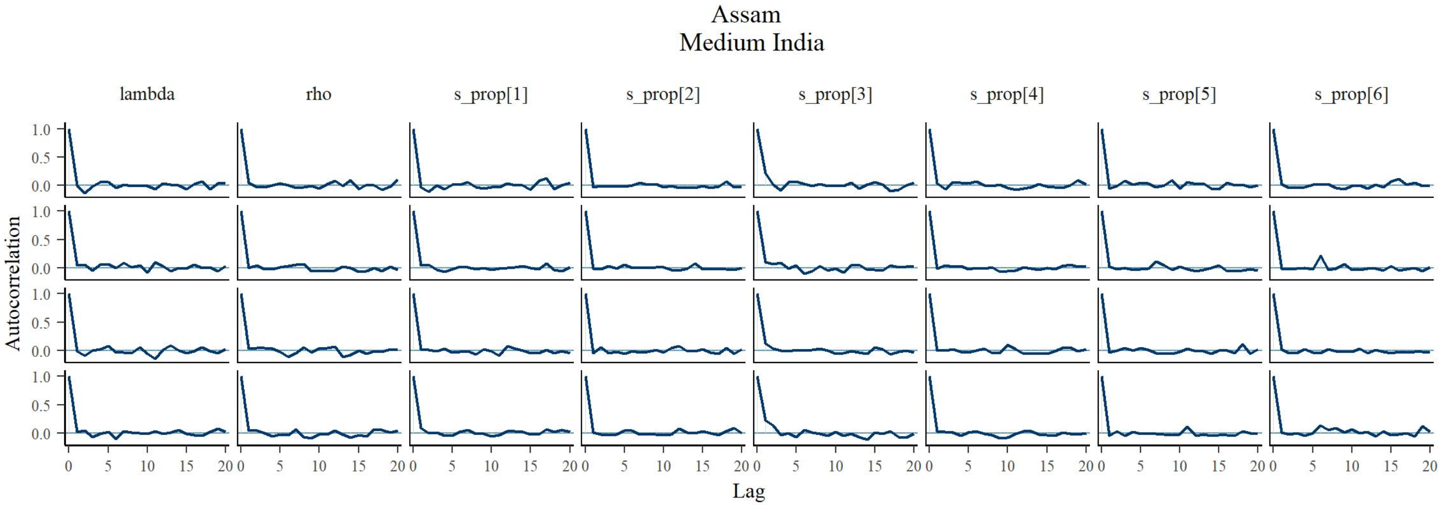
Assam
Medium India
lambda
rho
s_prop[1]
s_prop[2]
s_prop[3]
s_prop[4]
s_prop[5]
s_prop[6]
Autocorrelation
1.0
0.5
0.0
1
2
3
4
0
5
10
15
20
Lag

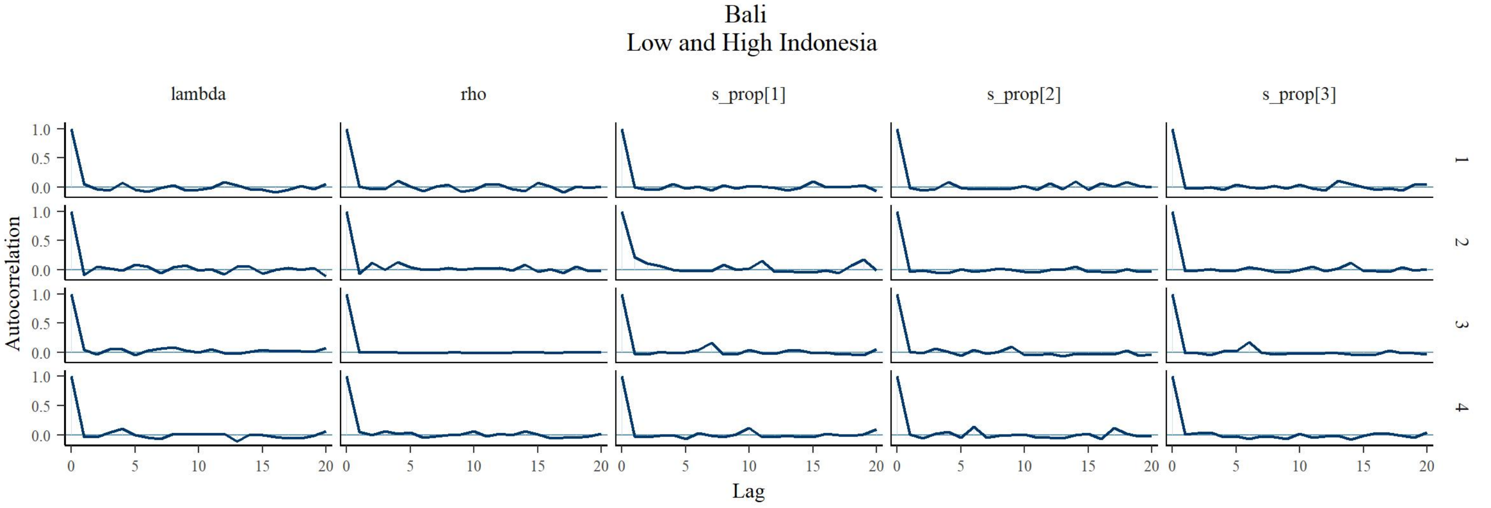
Bali
Low and High Indonesia
lambda
rho
s_prop[1]
s_prop[2]
s_prop[3]
Autocorrelation
Lag
1
2
3
4
1.0
0.5
0.0
0
5
10
15
20

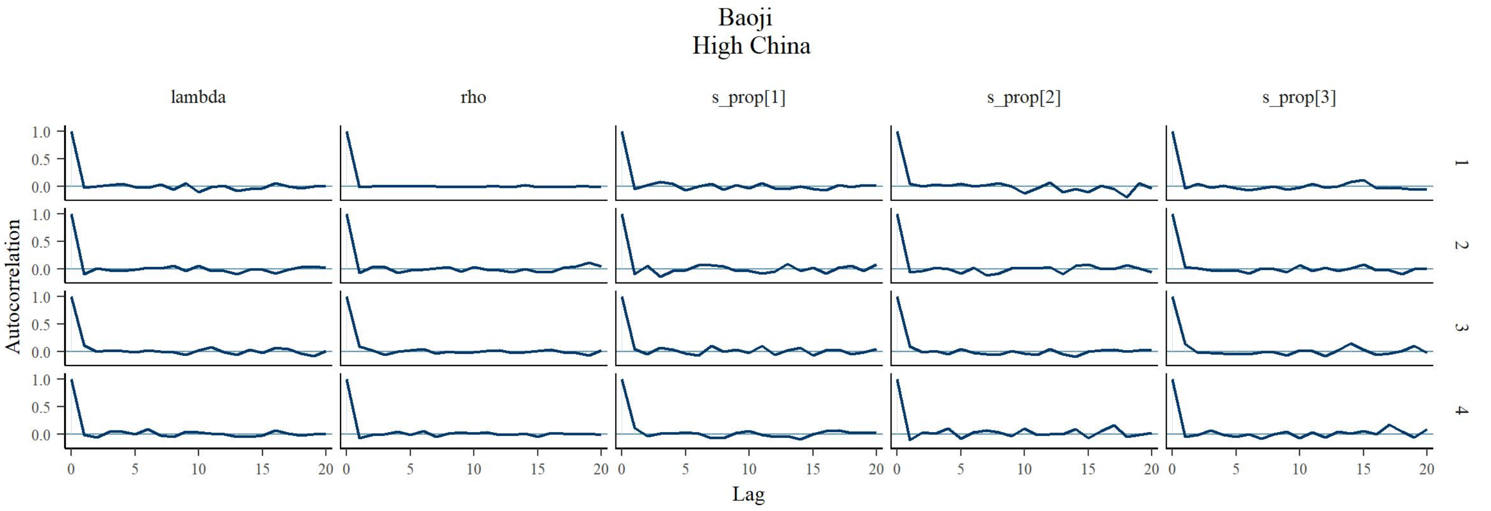

Baoji
High China
lambda
rho
s_prop[1]
s_prop[2]
s_prop[3]
Autocorrelation
Lag
1
2
3
4

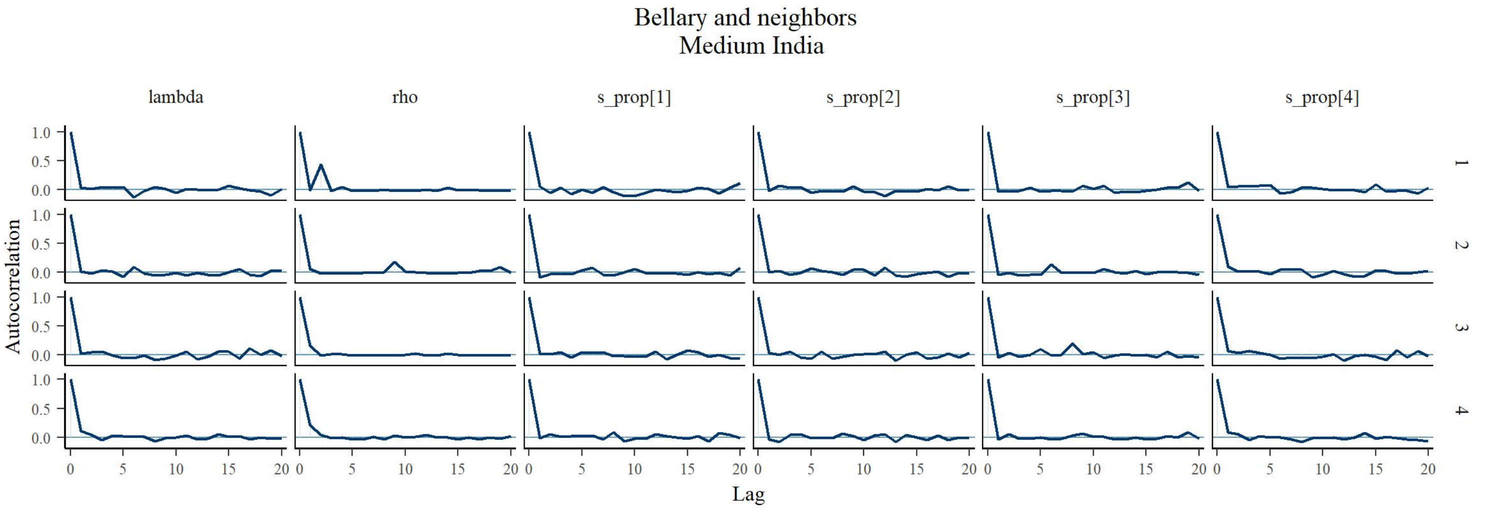
Bellary and neighbors
Medium India
lambda
rho
s_prop[1]
s_prop[2]
s_prop[3]
s_prop[4]
Autocorrelation
Lag
1
2
3
4
1.0
0.5
0.0
0
5
10
15
20

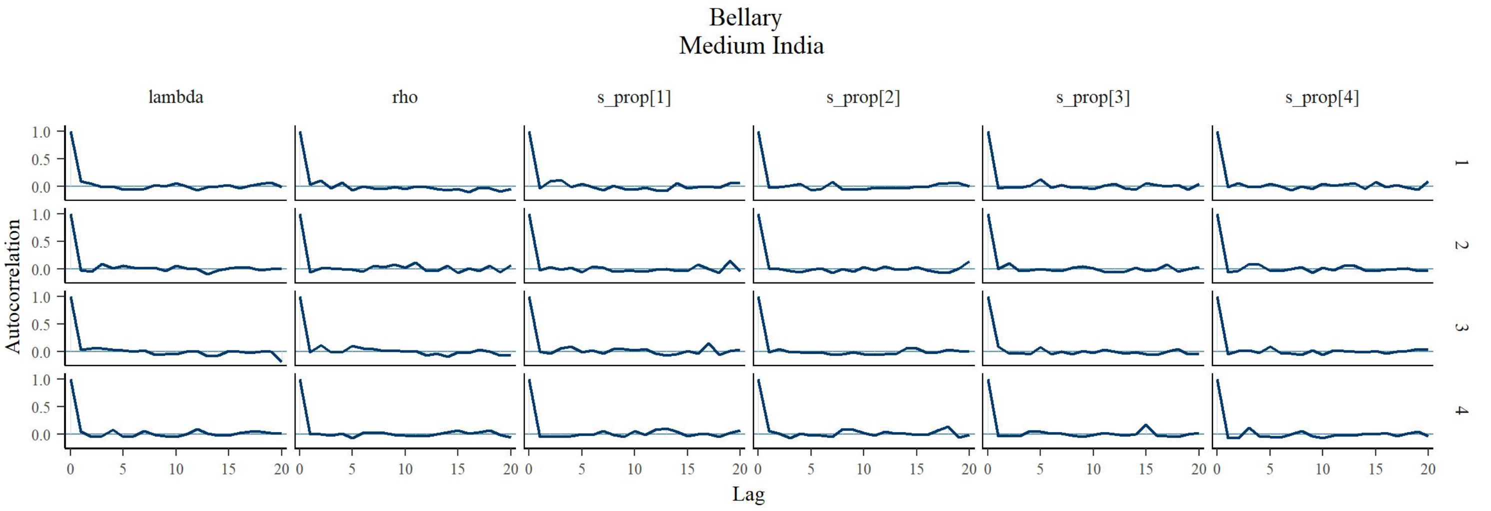

Bellary
Medium India
lambda
rho
s_prop[1]
s_prop[2]
s_prop[3]
s_prop[4]
1
2
3
4
Autocorrelation
1.0
0.5
0.0
0
5
10
15
20
Lag

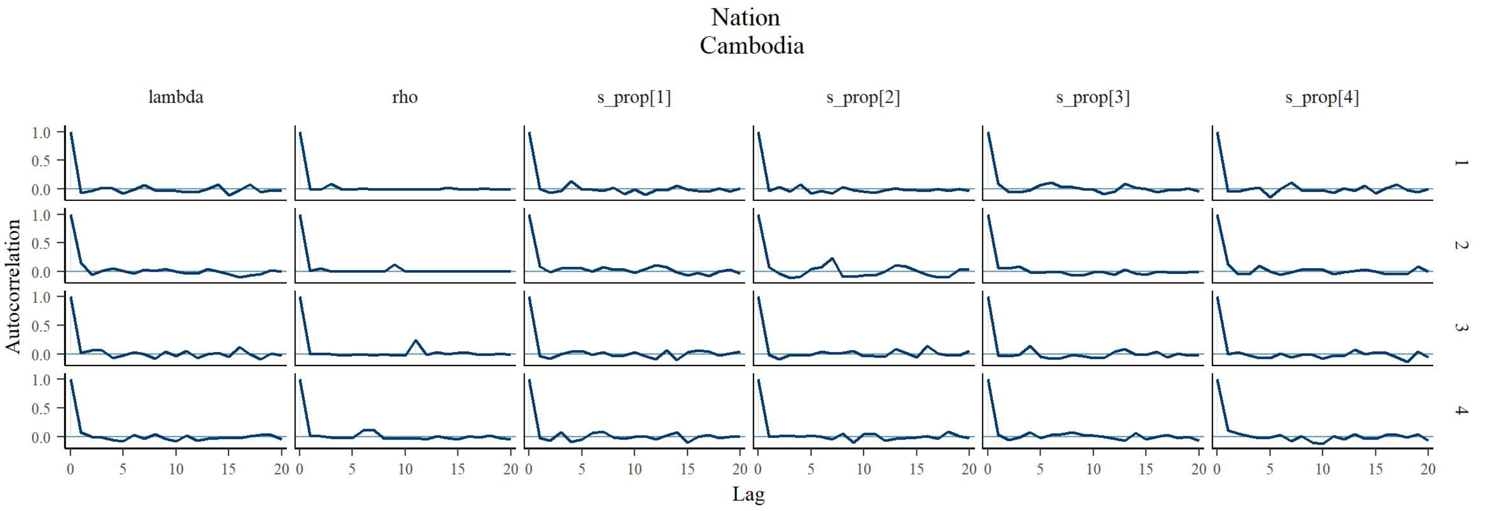

Nation
Cambodia
lambda
rho
s_prop[1]
s_prop[2]
s_prop[3]
s_prop[4]
Autocorrelation
Lag
1
2
3
4

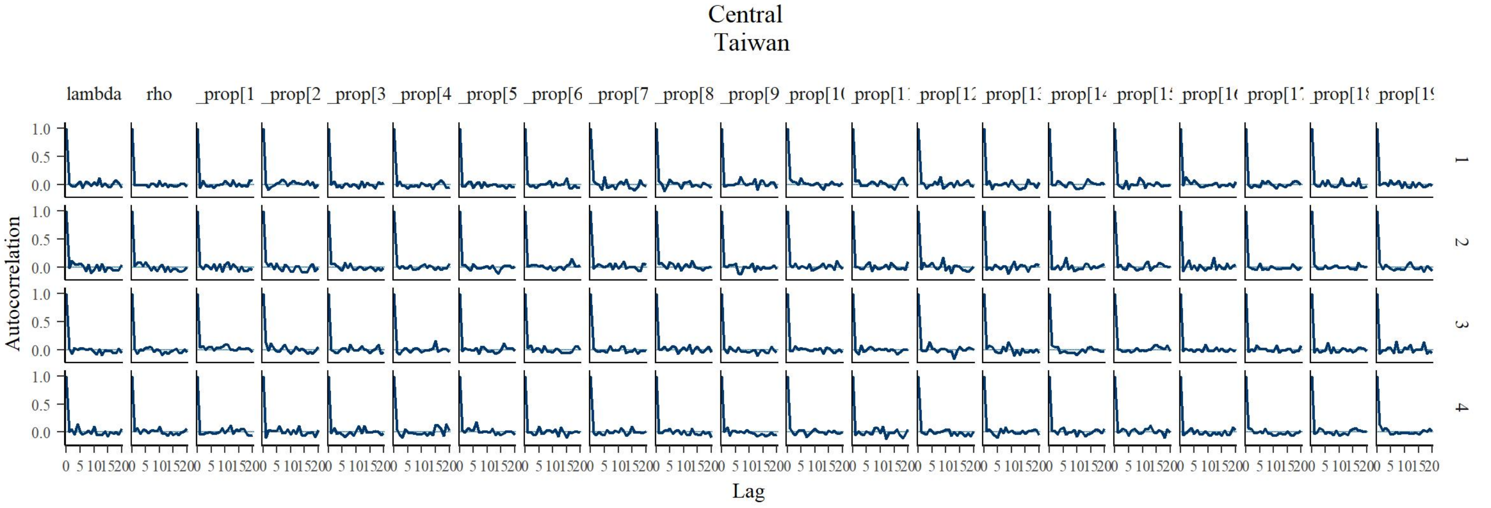

Central
Taiwan
lambda
rho
_prop[1
_prop[2
_prop[3
_prop[4
_prop[5
_prop[6
_prop[7
_prop[8
_prop[9
Autocorrelation
Lag

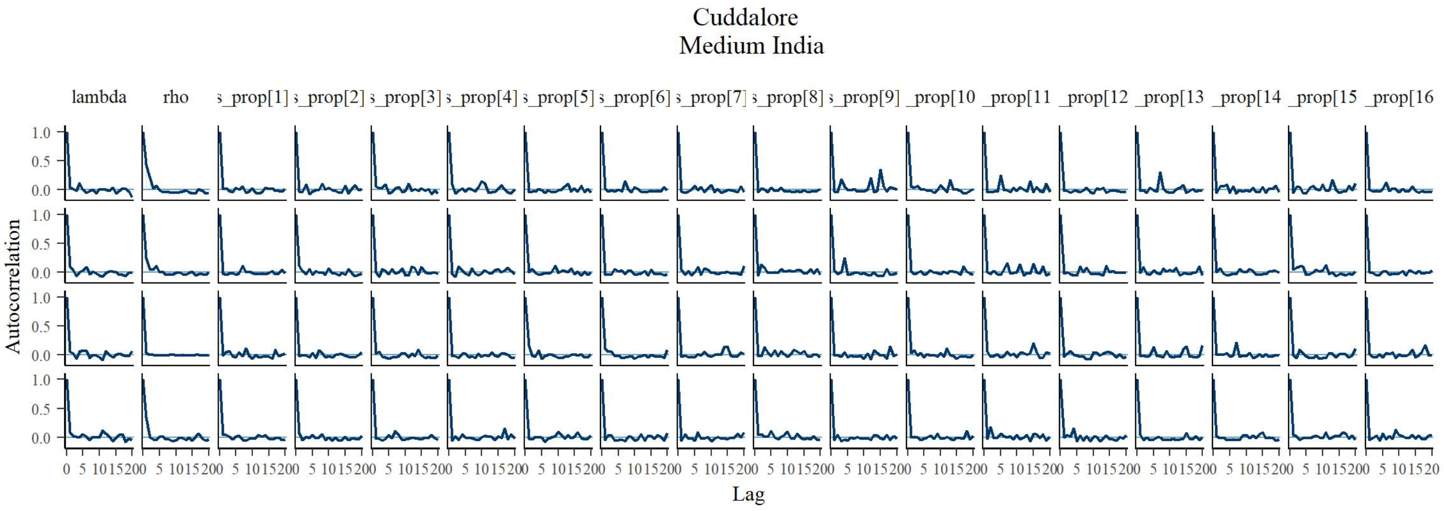

Cuddalore
Medium India
lambda
rho
s_prop[1]
s_prop[2]
s_prop[3]
s_prop[4]
s_prop[5]
s_prop[6]
s_prop[7]
s_prop[8]
s_prop[9]
_prop[10
_prop[11
_prop[12
_prop[13
_prop[14
_prop[15
_prop[16
Autocorrelation
Lag
1
2
3
4

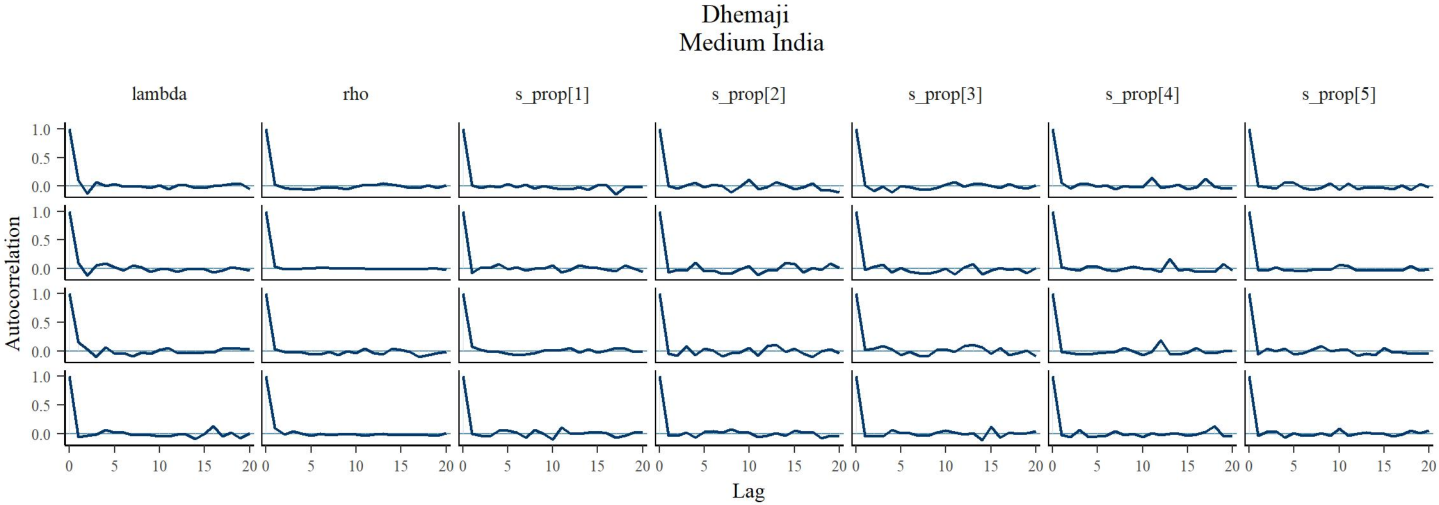

Dhemaji
Medium India
lambda
rho
s_prop[1]
s_prop[2]
s_prop[3]
s_prop[4]
s_prop[5]
1
2
3
4
Autocorrelation
1.0
0.5
0.0
0
5
10
15
20
Lag

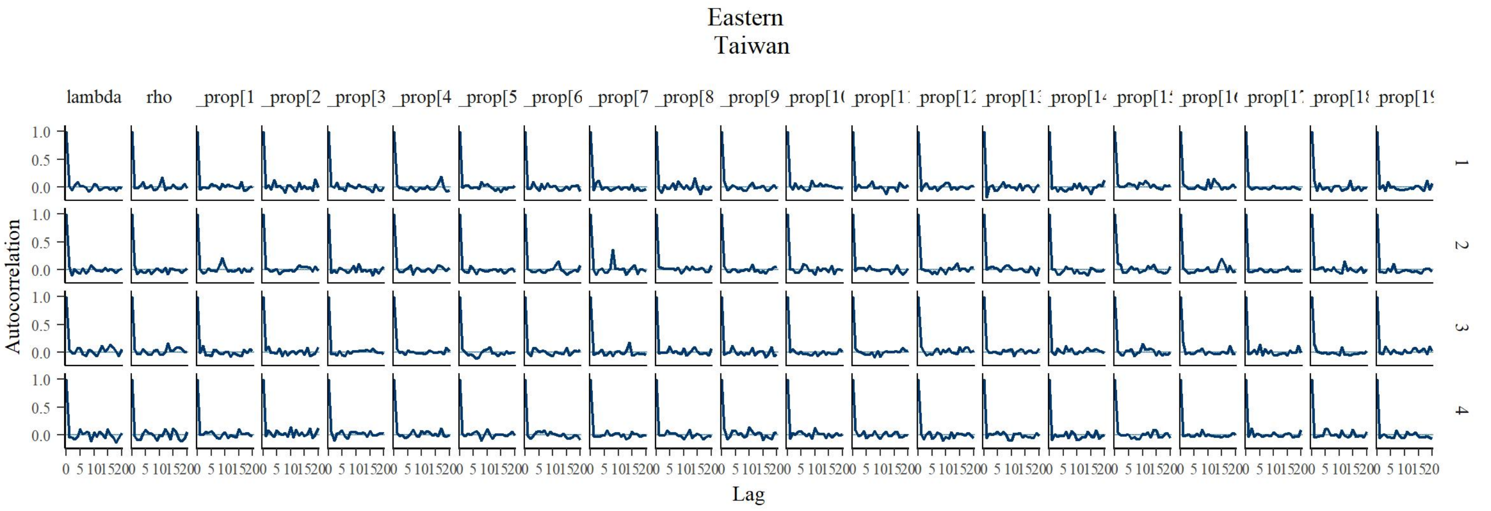

Eastern
Taiwan
lambda
rho
_prop[1
_prop[2
_prop[3
_prop[4
_prop[5
_prop[6
_prop[7
_prop[8
_prop[9
Autocorrelation
Lag
1
2
3
4

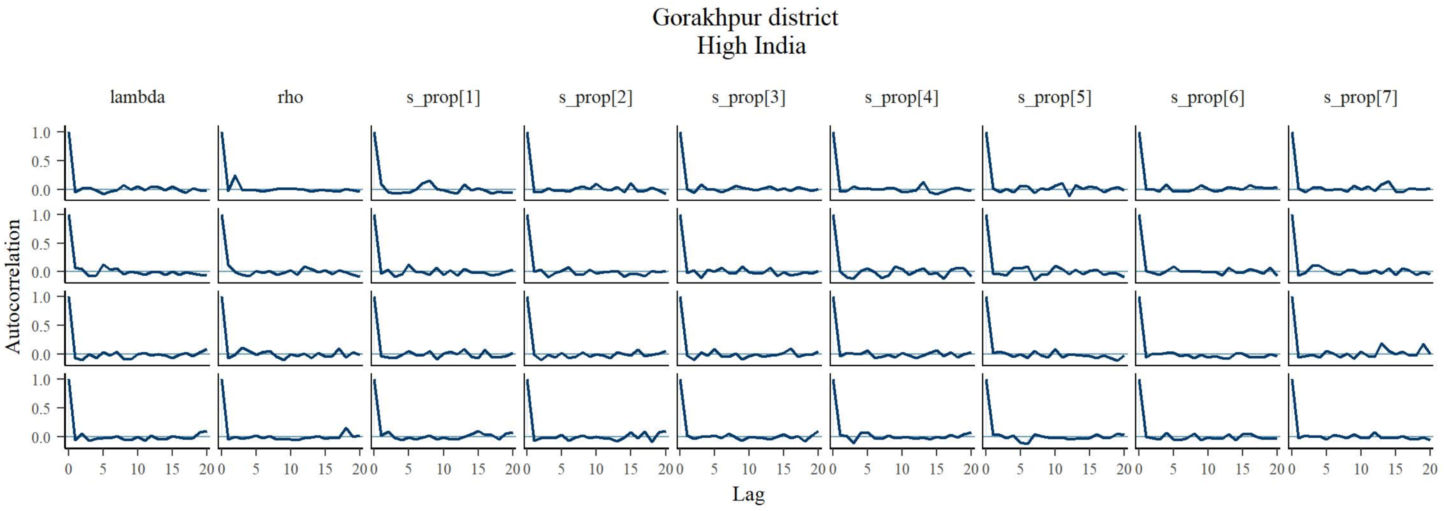

Gorakhpur district
High India
lambda
rho
s_prop[1]
s_prop[2]
s_prop[3]
s_prop[4]
s_prop[5]
s_prop[6]
s_prop[7]
Autocorrelation
1.0
0.5
0.0
1
2
3
4
0
5
10
15
20
Lag

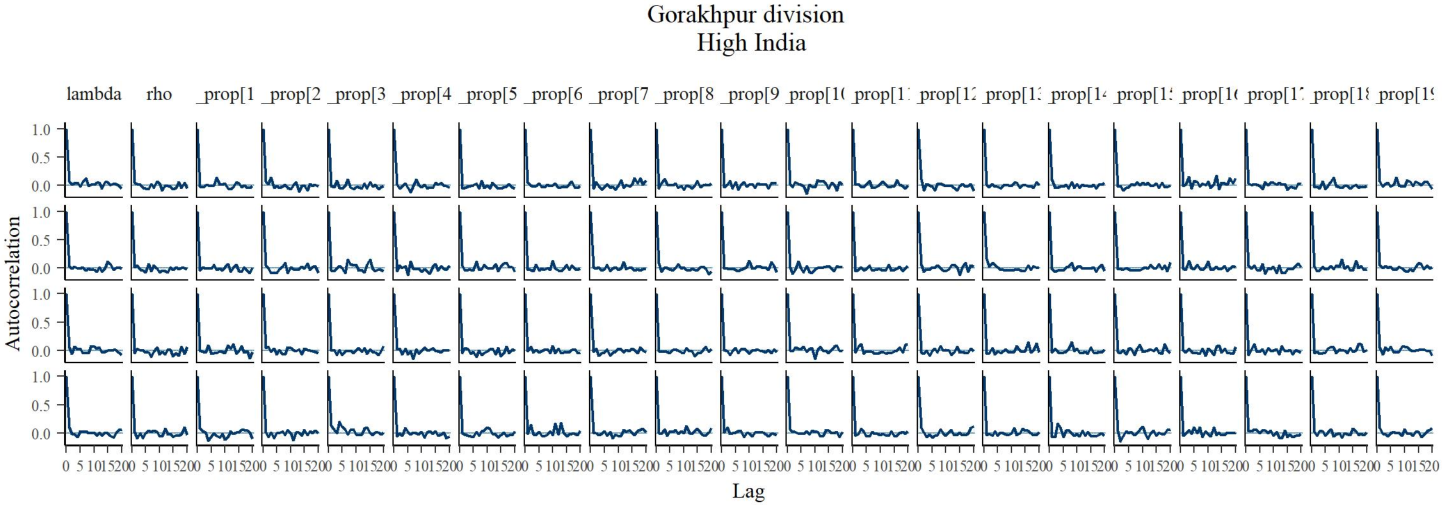
Gorakhpur division
High India
lambda
rho
_prop[1
_prop[2
_prop[3
_prop[4
_prop[5
_prop[6
_prop[7
_prop[8
_prop[9
Autocorrelation
Lag
1
2
3
4

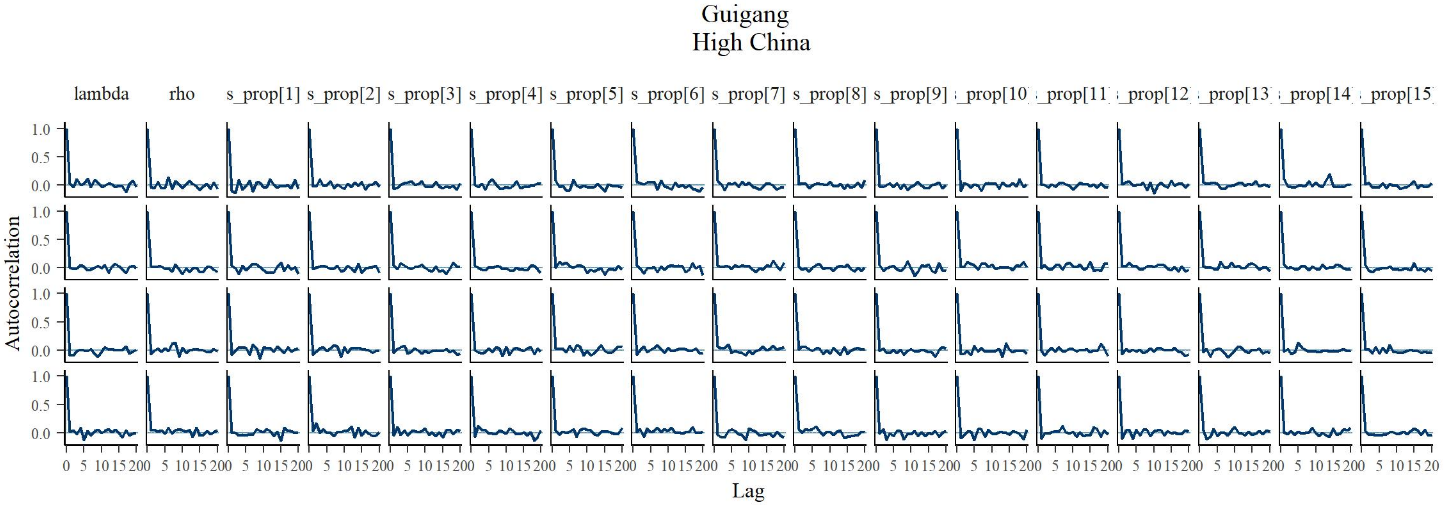

Guigang
High China
lambda
rho
s_prop[1]
s_prop[2]
s_prop[3]
s_prop[4]
s_prop[5]
s_prop[6]
s_prop[7]
s_prop[8]
s_prop[9]
Autocorrelation
Lag
1
2
3
4

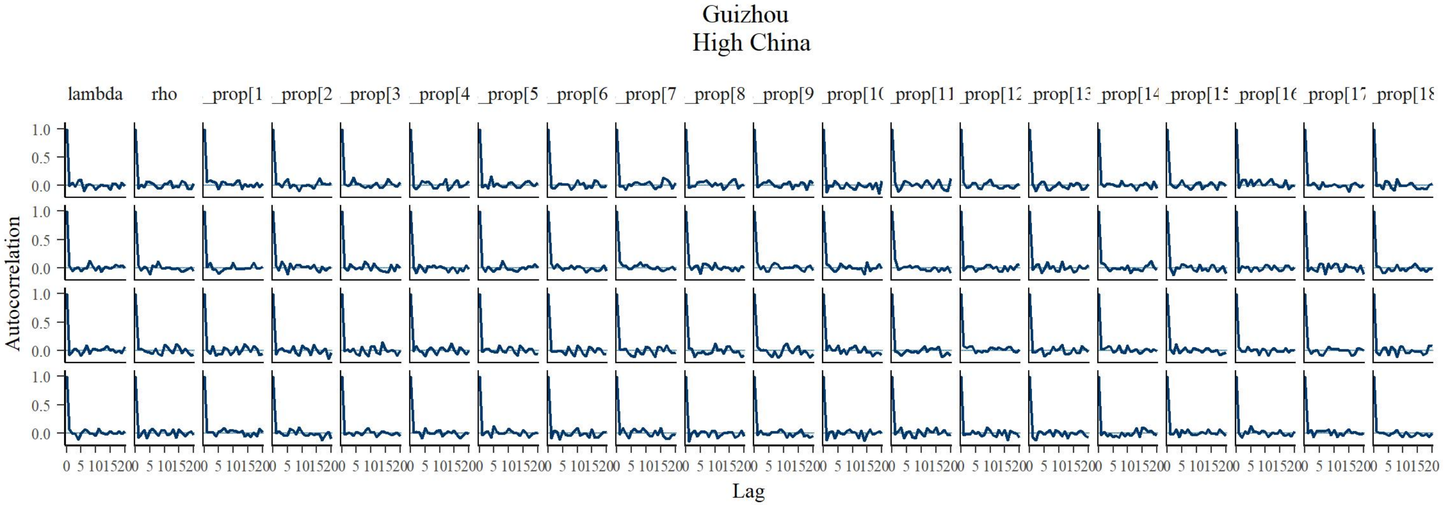

Guizhou
High China
lambda
rho
_prop[1
_prop[2
_prop[3
_prop[4
_prop[5
_prop[6
_prop[7
_prop[8
_prop[9
_prop[1(
_prop[11
_prop[12
_prop[13
_prop[14
_prop[15
_prop[16
_prop[17
_prop[18
Autocorrelation
1.0
0.5
0.0
1
2
3
4
0 5 10 15 20
Lag

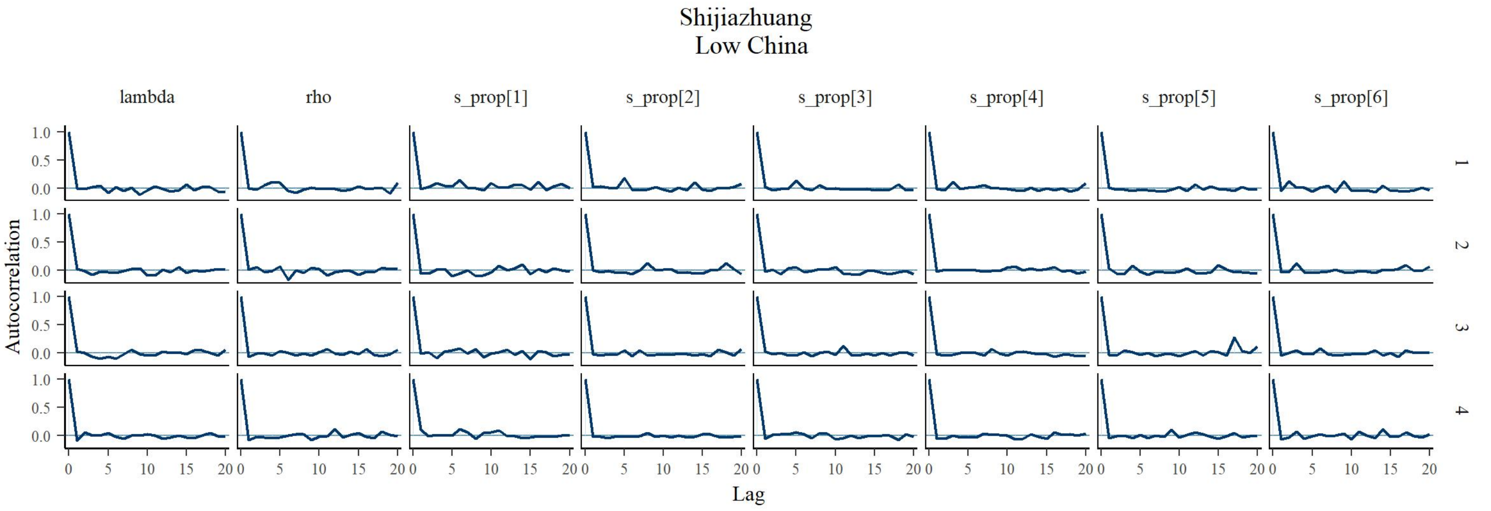

Shijiazhuang
Low China
lambda
rho
s_prop[1]
s_prop[2]
s_prop[3]
s_prop[4]
s_prop[5]
s_prop[6]
Autocorrelation
Lag
1
2
3
4

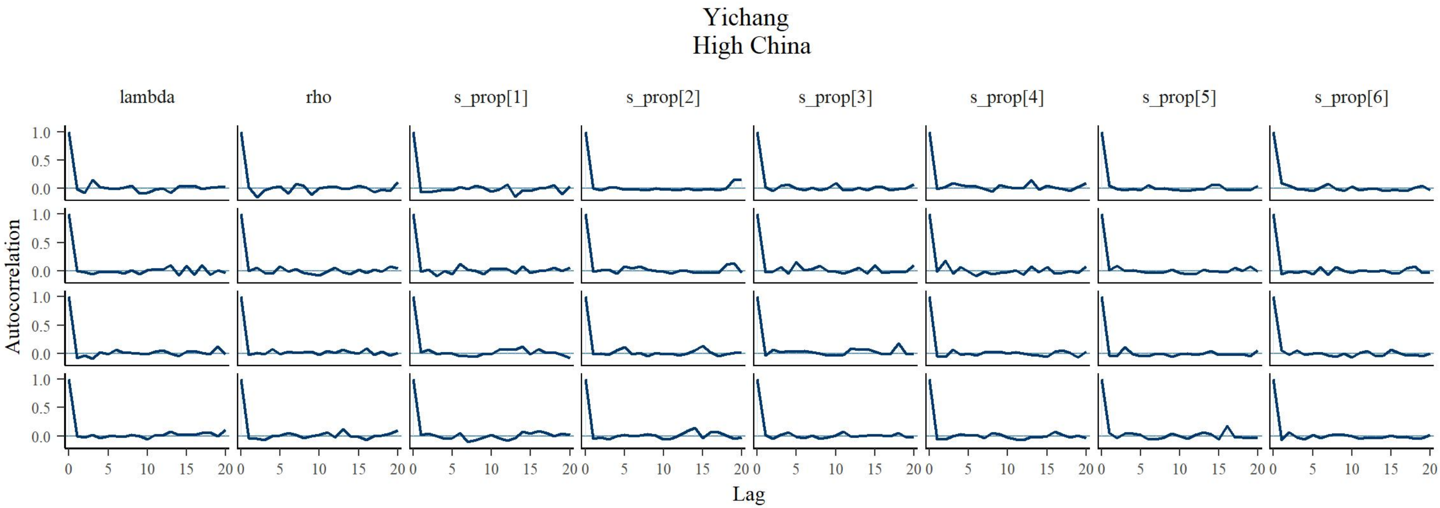

Yichang
High China
lambda
rho
s_prop[1]
s_prop[2]
s_prop[3]
s_prop[4]
s_prop[5]
s_prop[6]
Autocorrelation
1.0
0.5
0.0
1
2
3
4
0
5
10
15
20
Lag

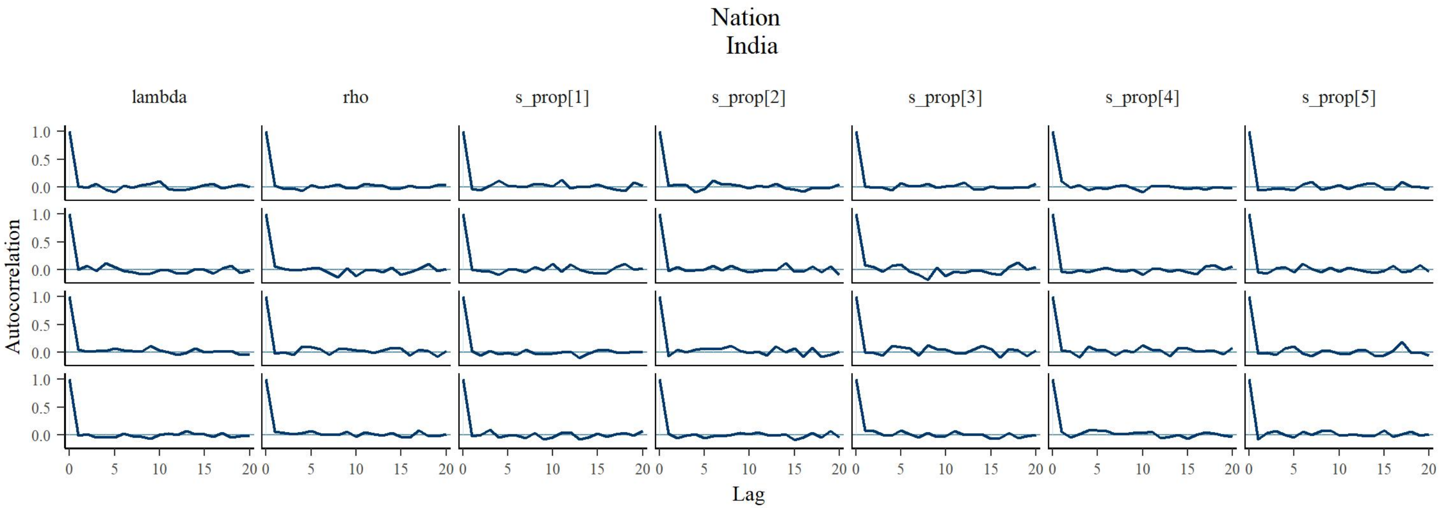

Nation
India
lambda
rho
s_prop[1]
s_prop[2]
s_prop[3]
s_prop[4]
s_prop[5]
Autocorrelation
Lag
1
2
3
4

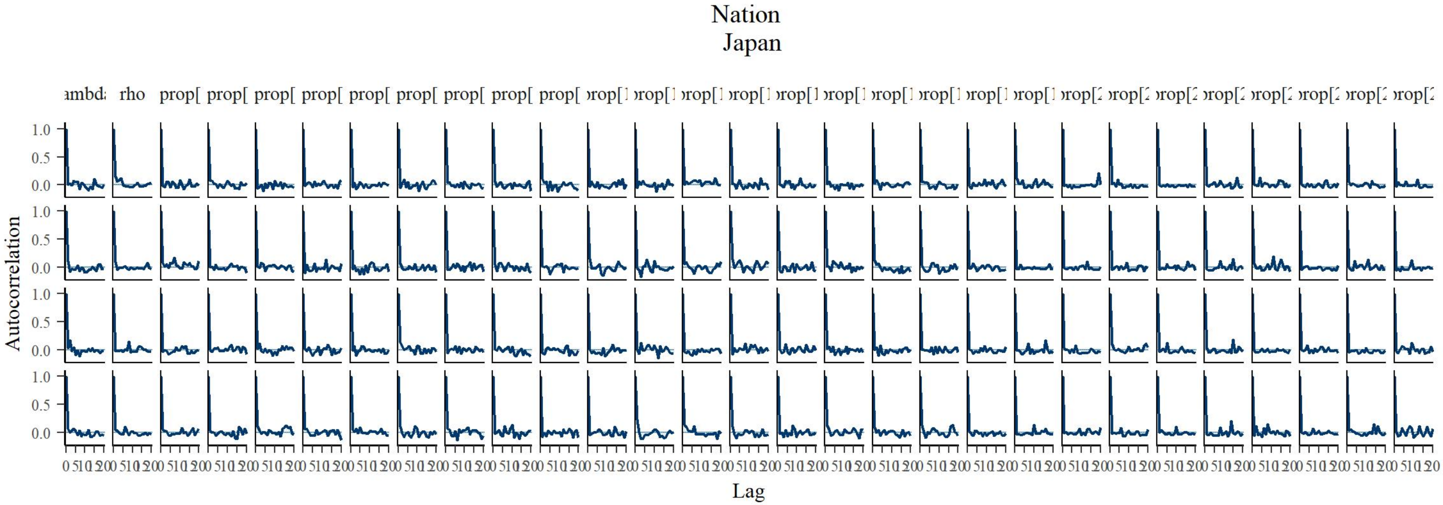

Nation
Japan
Autocorrelation
Lag

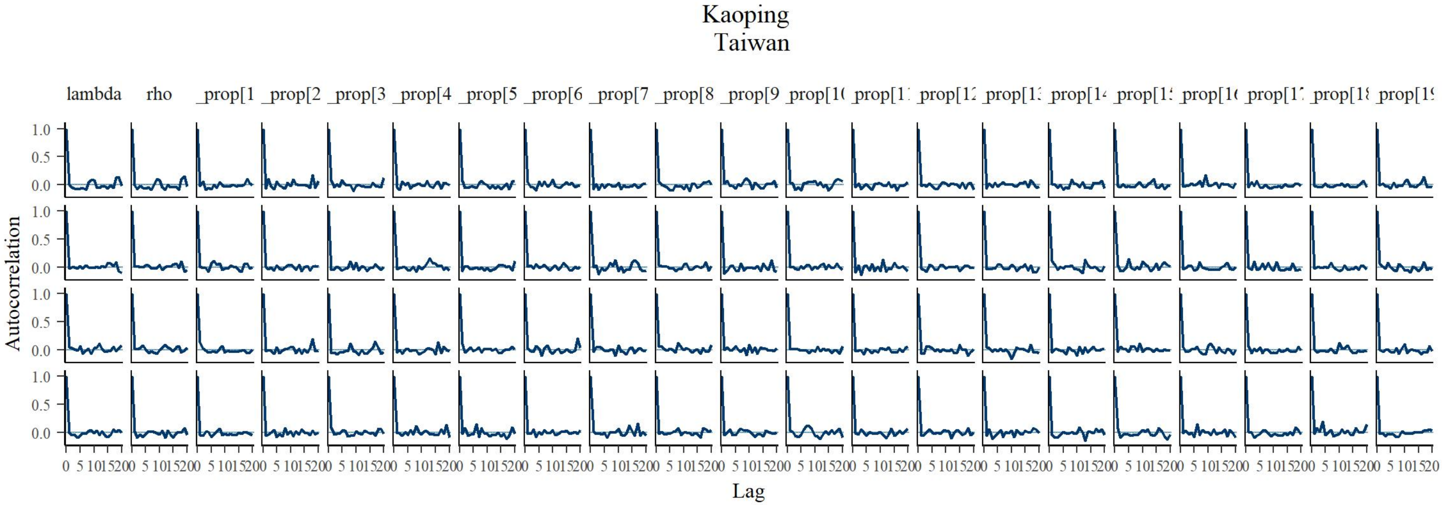
Kaoping
Taiwan
lambda
rho
_prop[1
_prop[2
_prop[3
_prop[4
_prop[5
_prop[6
_prop[7
_prop[8
_prop[9
_prop[1(
_prop[1:
_prop[12
_prop[1:
_prop[14
_prop[1:
_prop[1(
_prop[1:
_prop[18
_prop[19
1.0
0.5
0.0
Autocorrelation
1
2
3
4
0 5 101520
Lag

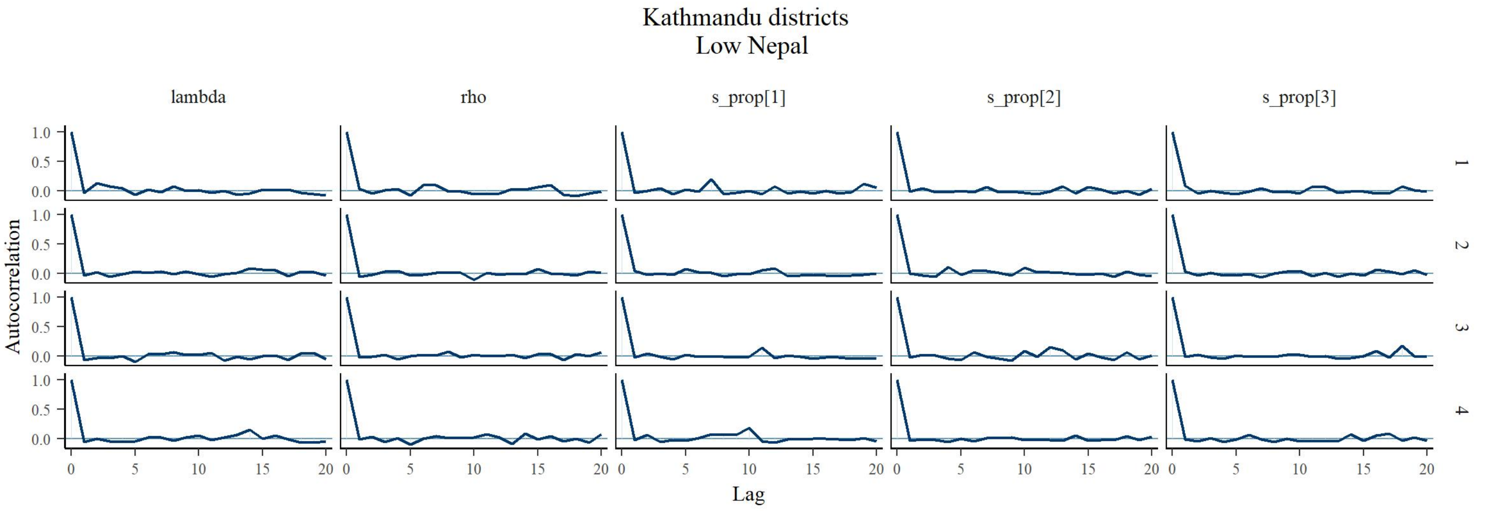

Kathmandu districts
Low Nepal
lambda
rho
s_prop[1]
s_prop[2]
s_prop[3]
Autocorrelation
Lag
1
2
3
4

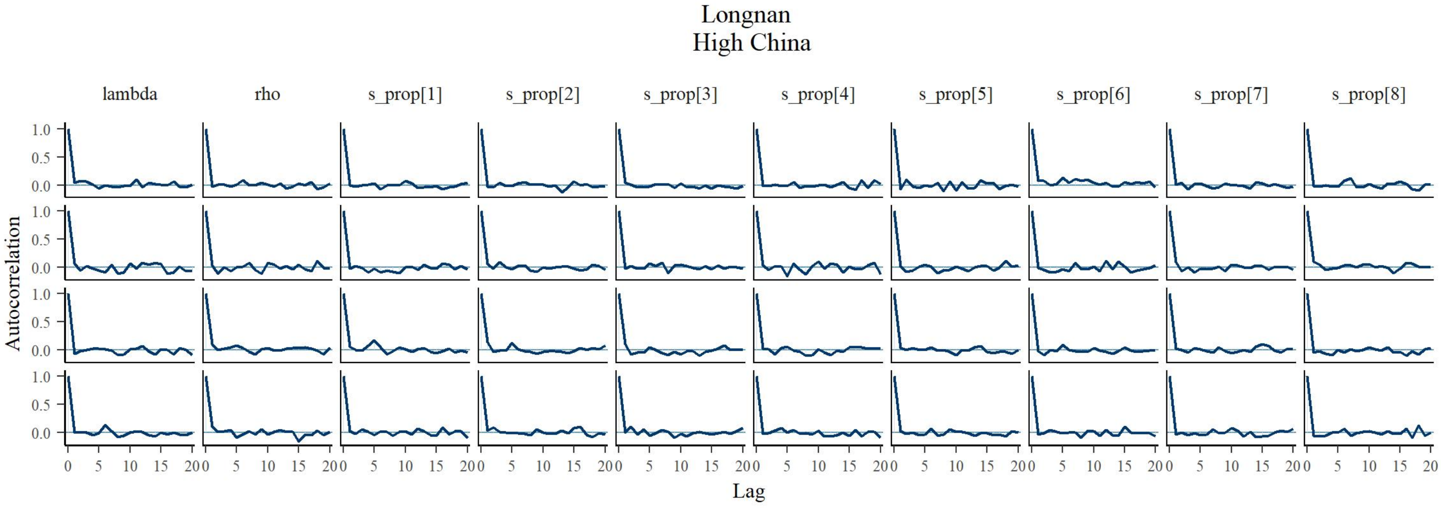
Longnan
High China
lambda
rho
s_prop[1]
s_prop[2]
s_prop[3]
s_prop[4]
s_prop[5]
s_prop[6]
s_prop[7]
s_prop[8]
1
2
3
4
Autocorrelation
1.0
0.5
0.0
0
5
10
15
20
Lag

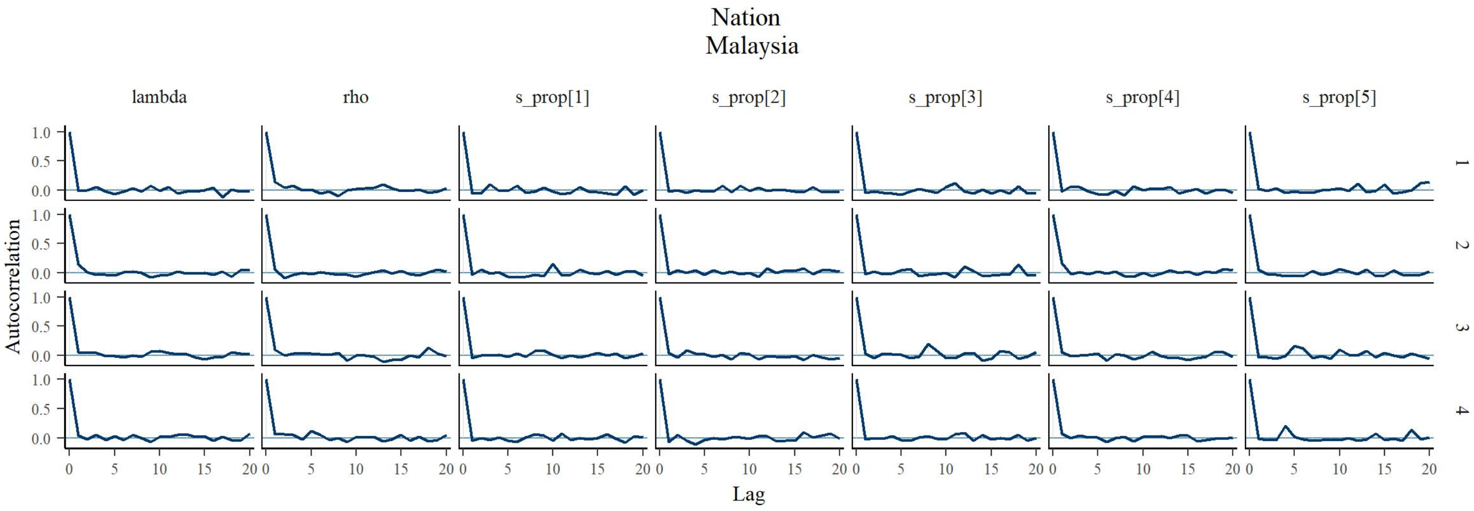

Nation
Malaysia
lambda
rho
s_prop[1]
s_prop[2]
s_prop[3]
s_prop[4]
s_prop[5]
1
2
3
4
Autocorrelation
Lag

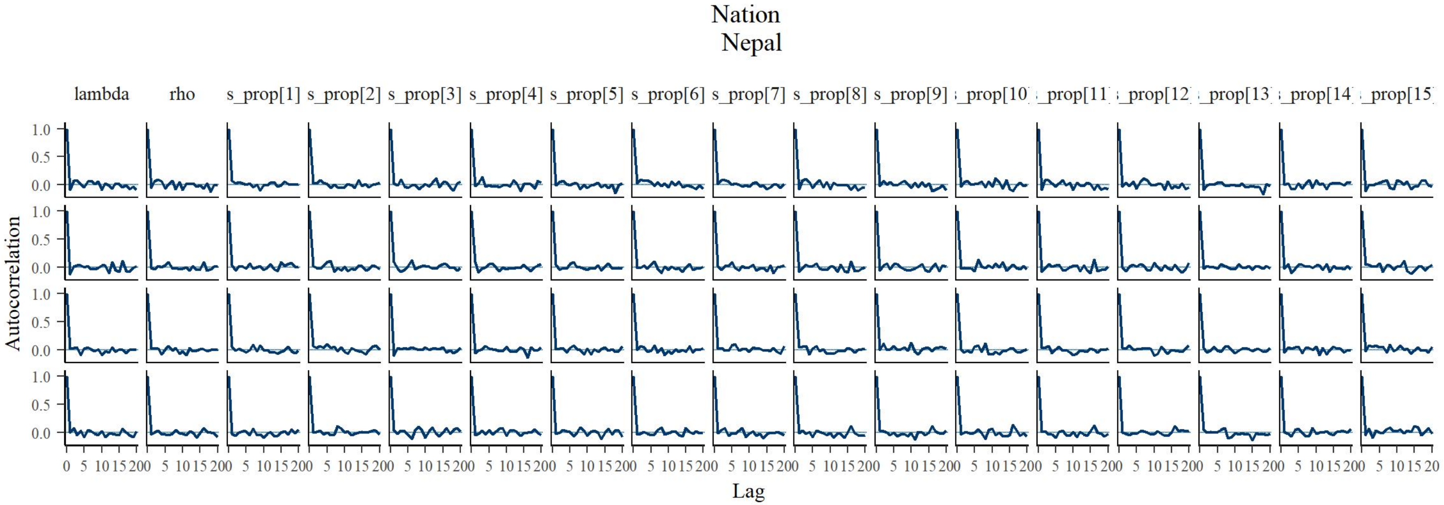

Nation
Nepal
lambda
rho
s_prop[1]
s_prop[2]
s_prop[3]
s_prop[4]
s_prop[5]
s_prop[6]
s_prop[7]
s_prop[8]
s_prop[9]
_prop[10
_prop[11
_prop[12
_prop[13
_prop[14
_prop[15
1.0
0.5
0.0
Autocorrelation
1
2
3
4
0 5 10 15 20
Lag

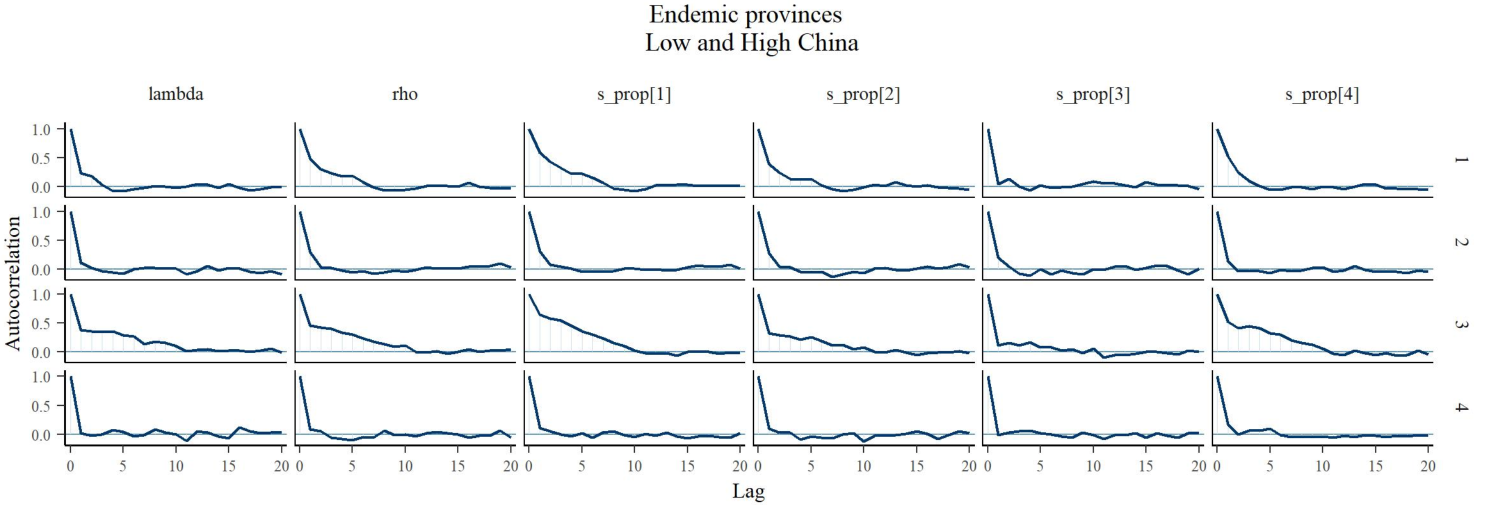

Endemic provinces
Low and High China
lambda
rho
s_prop[1]
s_prop[2]
s_prop[3]
s_prop[4]
Autocorrelation
Lag
1
2
3
4

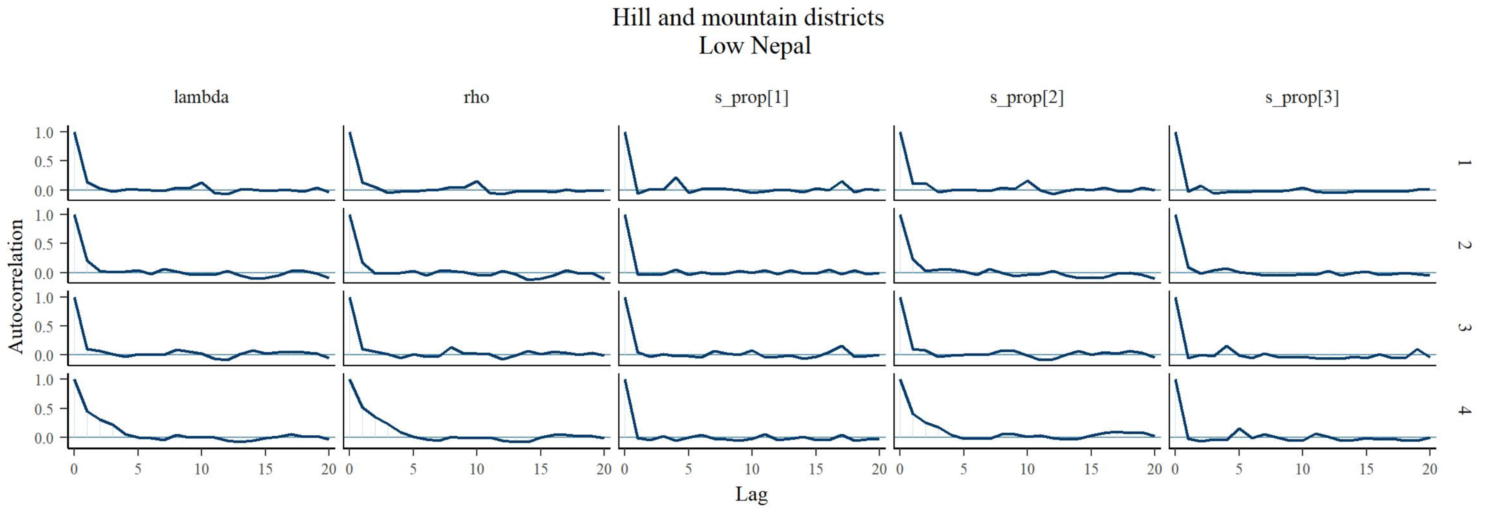
Hill and mountain districts
Low Nepal
lambda
rho
s_prop[1]
s_prop[2]
s_prop[3]
Autocorrelation
Lag
1
2
3
4
1.0
0.5
0.0
0
5
10
15
20

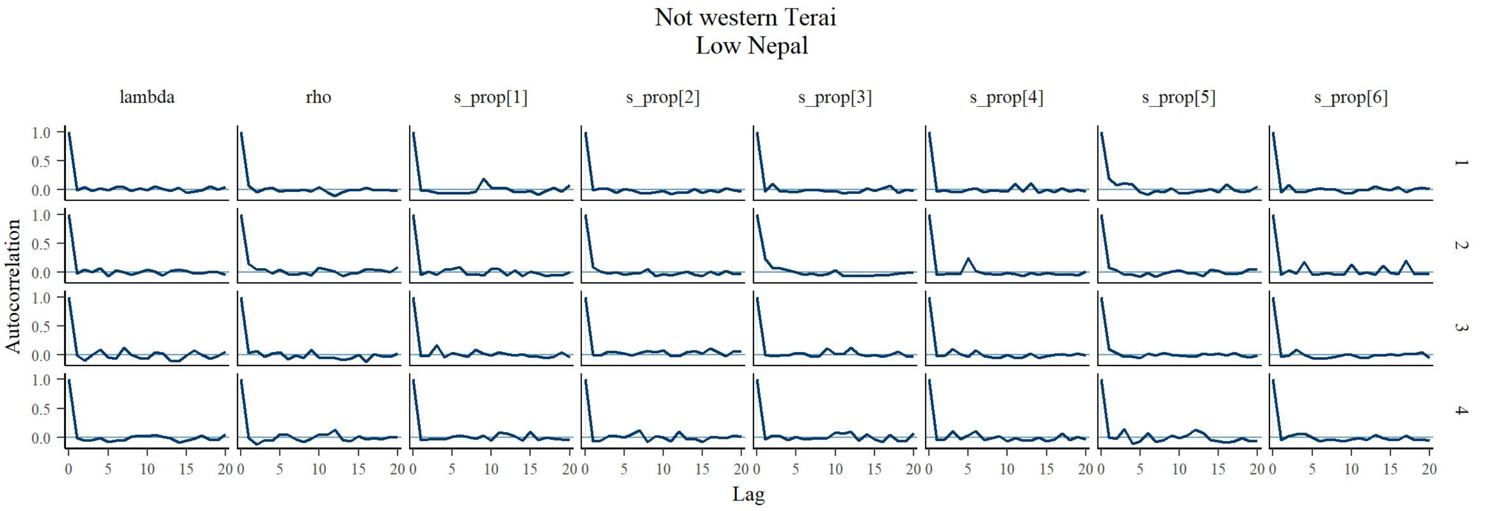
Not western Terai
Low Nepal
lambda
rho
s_prop[1]
s_prop[2]
s_prop[3]
s_prop[4]
s_prop[5]
s_prop[6]
Autocorrelation
Lag
1
2
3
4

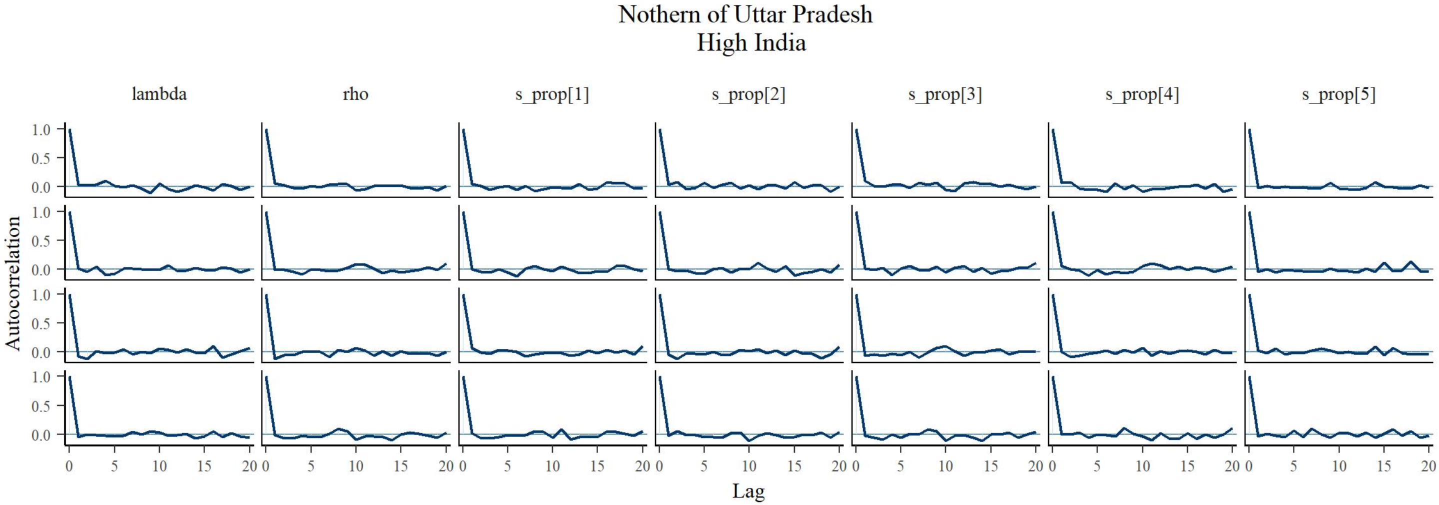

Nothern of Uttar Pradesh
High India
lambda
rho
s_prop[1]
s_prop[2]
s_prop[3]
s_prop[4]
s_prop[5]
Autocorrelation
1.0
0.5
0.0
1
2
3
4
0 5 10 15 20
Lag

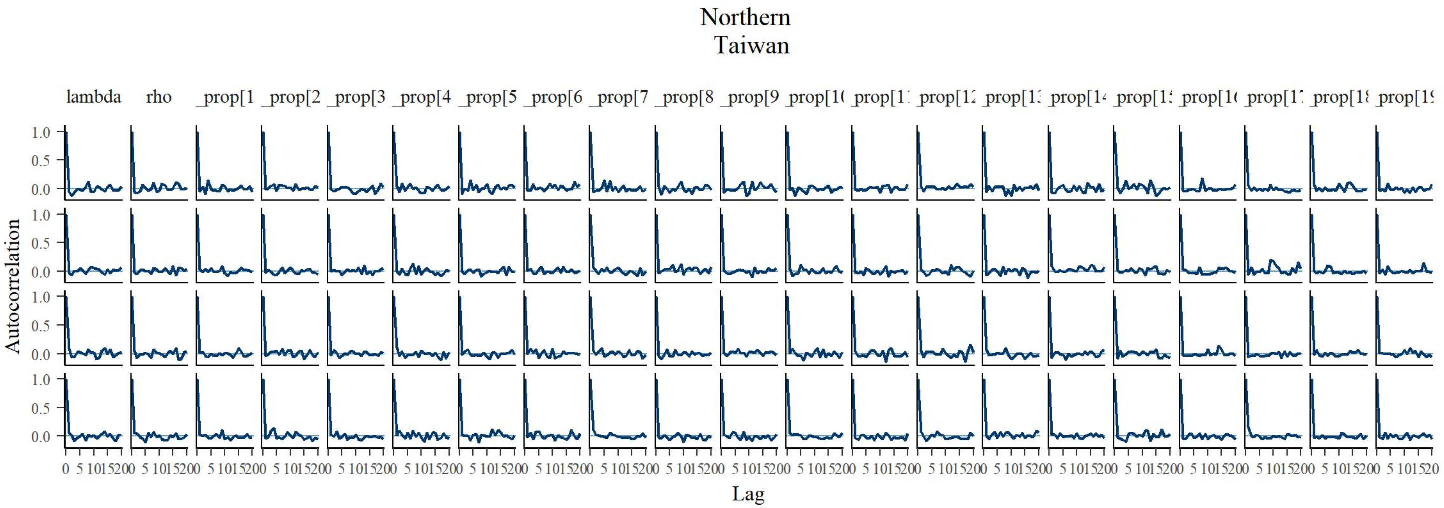

Northern
Taiwan
lambda
rho
_prop[1
_prop[2
_prop[3
_prop[4
_prop[5
_prop[6
_prop[7
_prop[8
_prop[9
_prop[1(
_prop[1
_prop[12
_prop[1
_prop[14
_prop[1
_prop[1(
_prop[1
_prop[18
_prop[19
Autocorrelation
1.0
0.5
0.0
1
2
3
4
Lag
0 5 101520

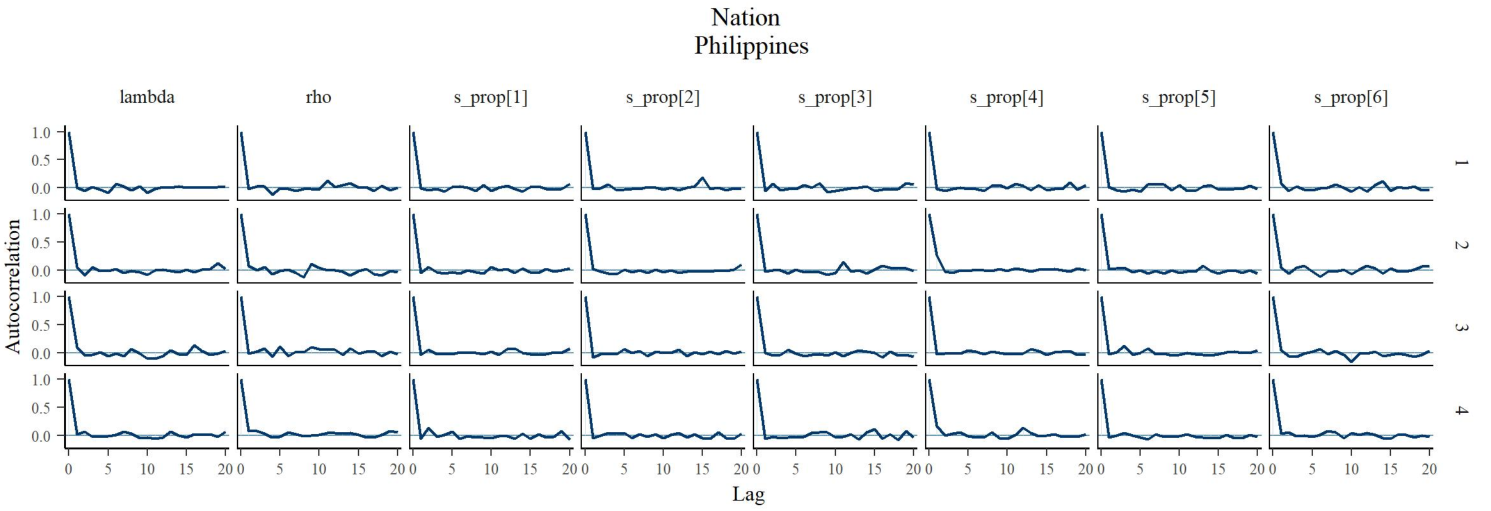

Nation
Philippines
lambda
rho
s_prop[1]
s_prop[2]
s_prop[3]
s_prop[4]
s_prop[5]
s_prop[6]
Autocorrelation
1.0
0.5
0.0
1
2
3
4
0
5
10
15
20
Lag

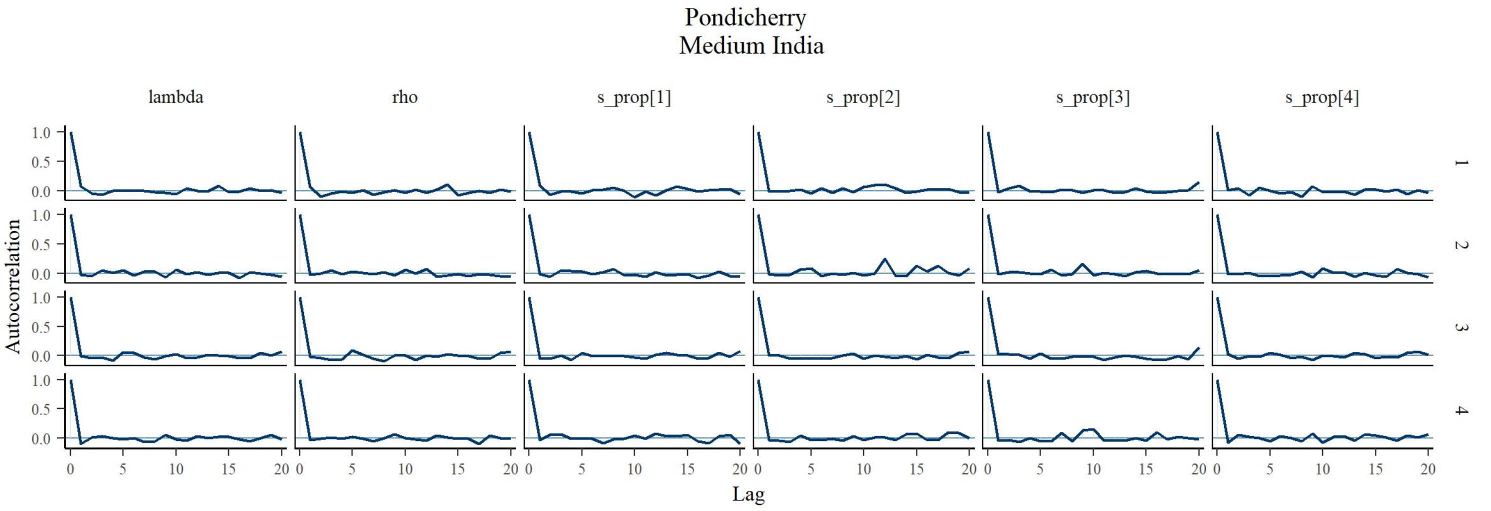

Pondicherry
Medium India
lambda
rho
s_prop[1]
s_prop[2]
s_prop[3]
s_prop[4]
1
2
3
4
1.0
0.5
0.0
Autocorrelation
0
5
10
15
20
Lag

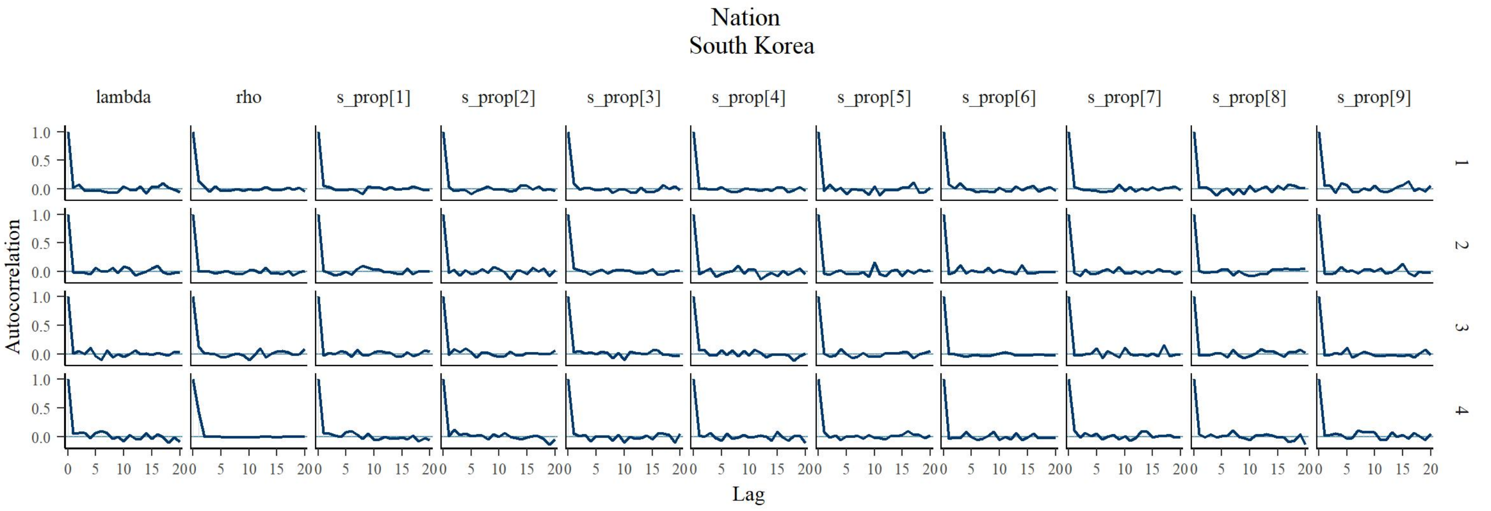

Nation
South Korea
lambda
rho
s_prop[1]
s_prop[2]
s_prop[3]
s_prop[4]
s_prop[5]
s_prop[6]
s_prop[7]
s_prop[8]
s_prop[9]
1
2
3
4
Autocorrelation
Lag

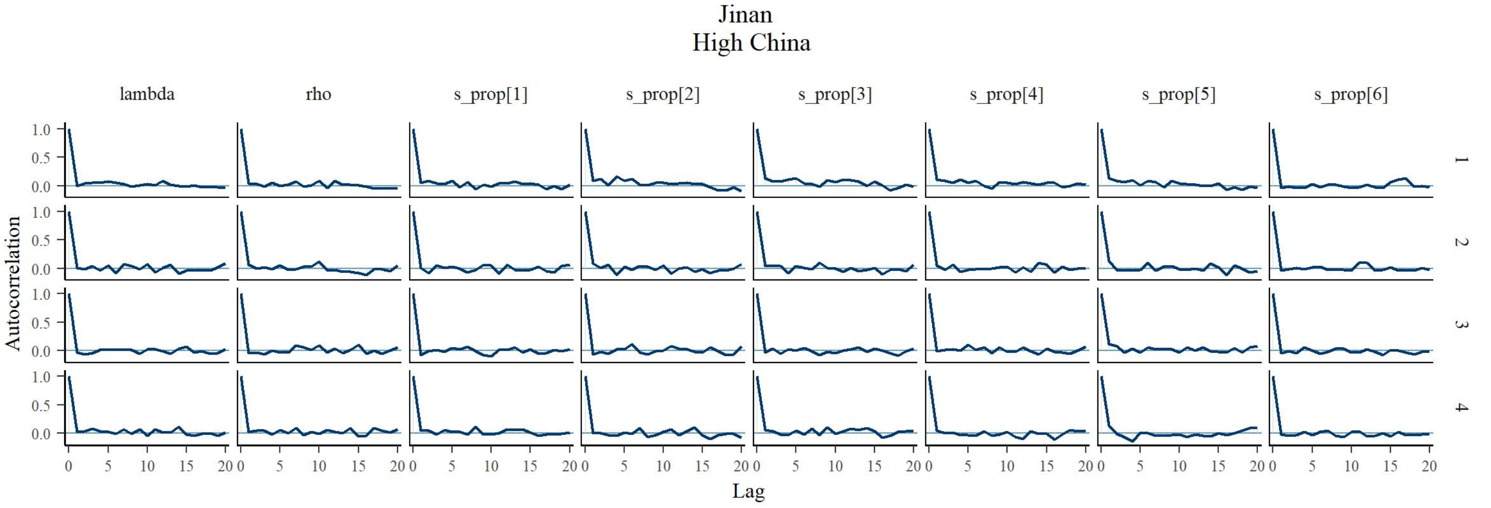

Jinan
High China
lambda
rho
s_prop[1]
s_prop[2]
s_prop[3]
s_prop[4]
s_prop[5]
s_prop[6]
Autocorrelation
Lag
1
2
3
4
0.0
0.5
1.0
0
5
10
15
20

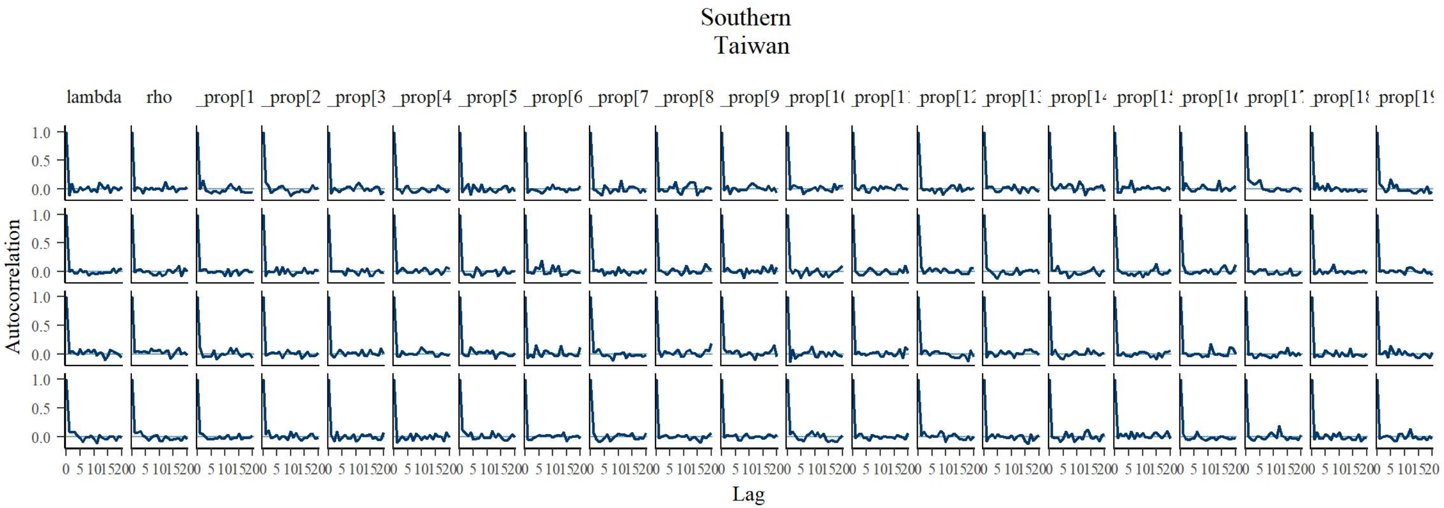

Southern
Taiwan
lambda
rho
_prop[1
_prop[2
_prop[3
_prop[4
_prop[5
_prop[6
_prop[7
_prop[8
_prop[9
_prop[1(
_prop[1:
_prop[12
_prop[1:
_prop[14
_prop[1!
_prop[1(
_prop[1'
_prop[18
_prop[19
Autocorrelation
1.0
0.5
0.0
1
2
3
4
0 5 101520
Lag

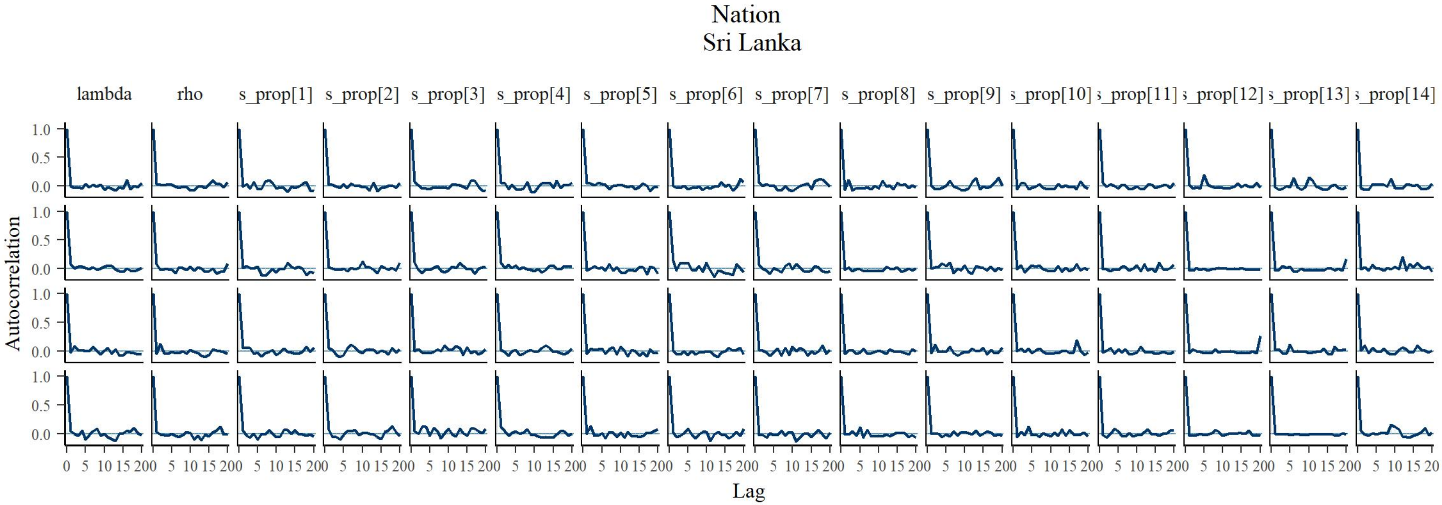

Nation
Sri Lanka
lambda
rho
s_prop[1]
s_prop[2]
s_prop[3]
s_prop[4]
s_prop[5]
s_prop[6]
s_prop[7]
s_prop[8]
s_prop[9]
s_prop[10]
s_prop[11]
s_prop[12]
s_prop[13]
s_prop[14]
1
2
3
4
Autocorrelation
Lag

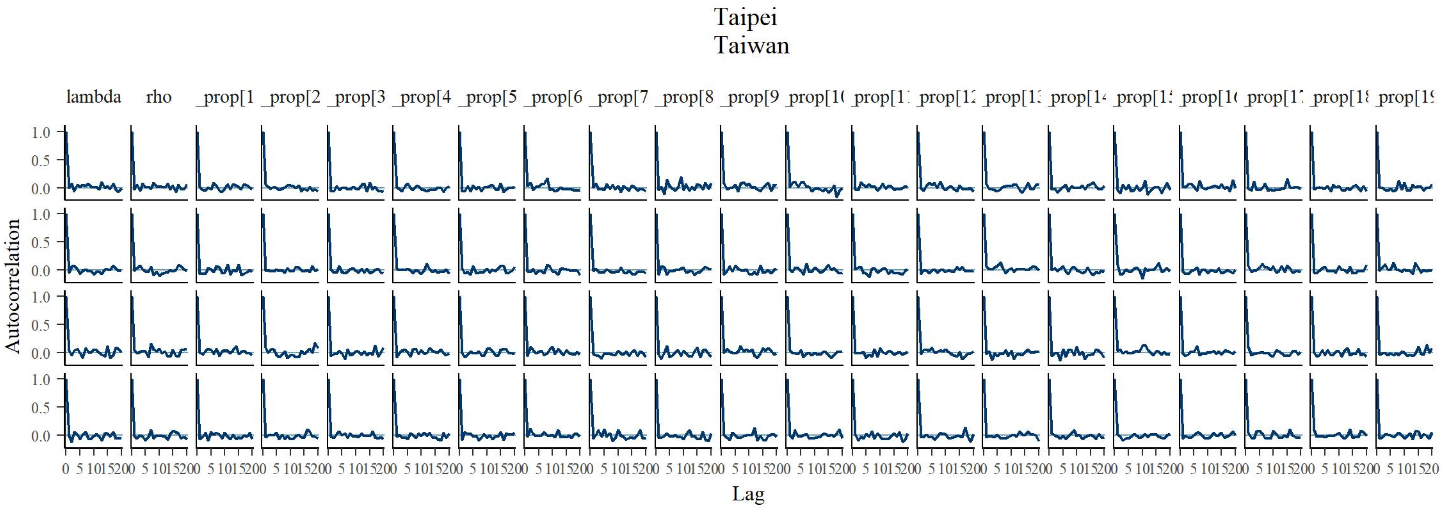

Taipei
Taiwan
lambda
rho
_prop[1
_prop[2
_prop[3
_prop[4
_prop[5
_prop[6
_prop[7
_prop[8
_prop[9
_prop[1(
_prop[1:
_prop[12
_prop[1:
_prop[14
_prop[1:
_prop[1(
_prop[1:
_prop[18
_prop[19
Autocorrelation
1.0
0.5
0.0
1
2
3
4
0 5 101520
Lag

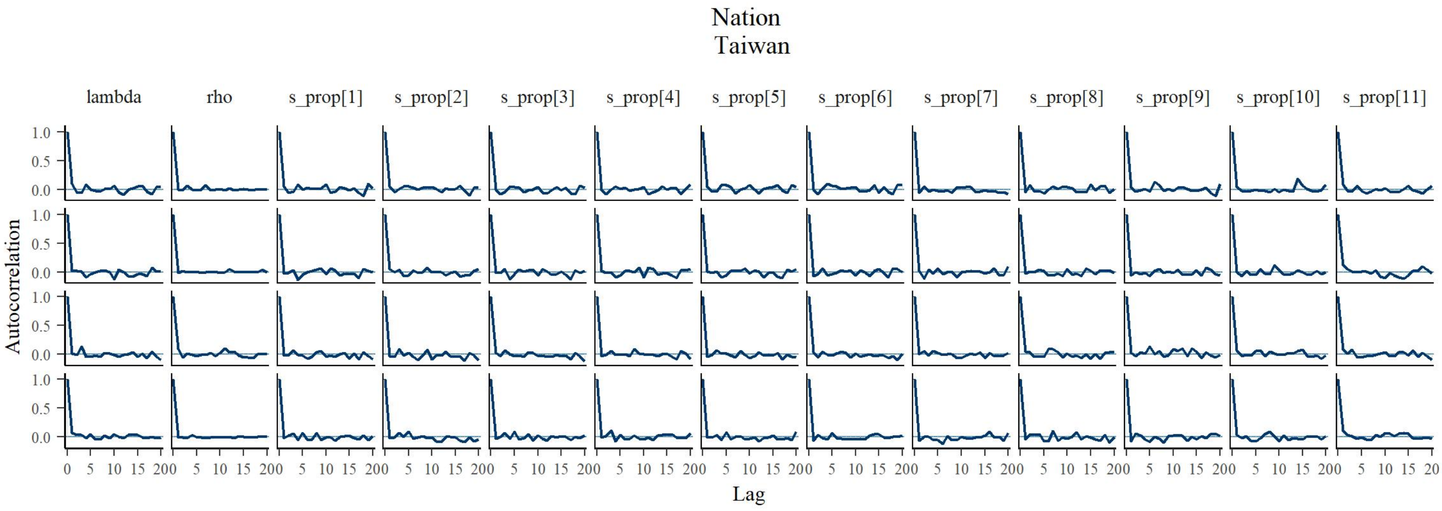

Nation
Taiwan
lambda
rho
s_prop[1]
s_prop[2]
s_prop[3]
s_prop[4]
s_prop[5]
s_prop[6]
s_prop[7]
s_prop[8]
s_prop[9]
s_prop[10]
s_prop[11]
Autocorrelation
1.0
0.5
0.0
1
2
3
4
0
5
10
15
20
Lag

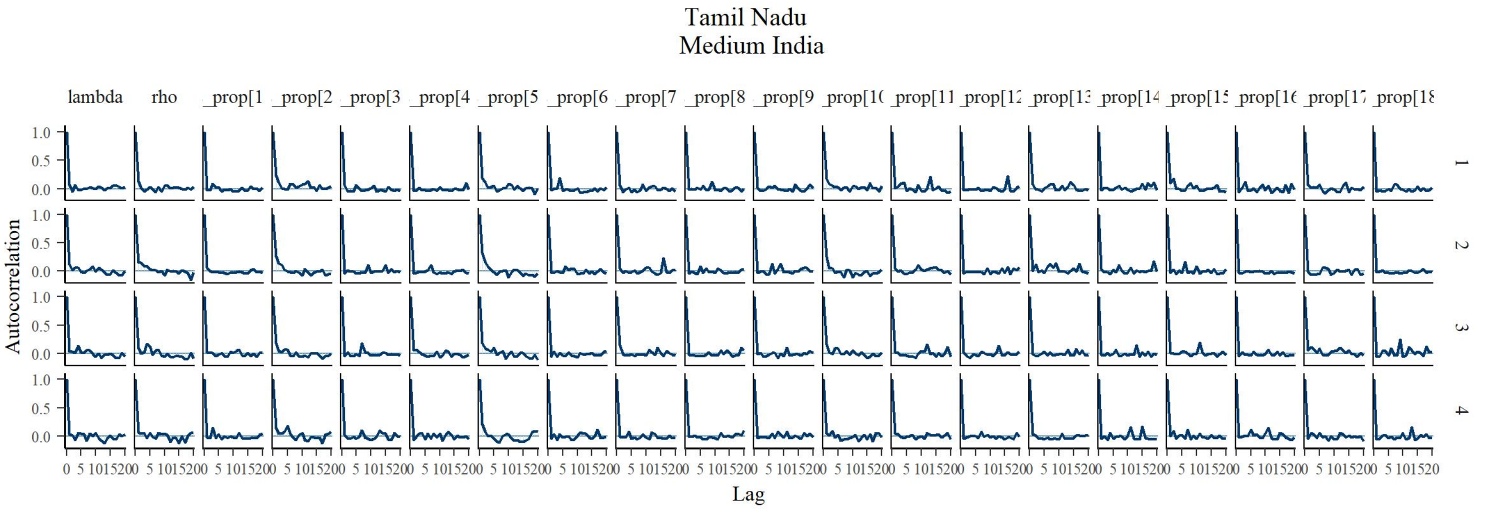

Tamil Nadu
Medium India
lambda
rho
_prop[1
_prop[2
_prop[3
_prop[4
_prop[5
_prop[6
_prop[7
_prop[8
_prop[9
_prop[1(
_prop[11
_prop[12
_prop[13
_prop[14
_prop[15
_prop[16
_prop[17
_prop[18
Autocorrelation
1.0
0.5
0.0
1
2
3
4
0 5 101520
Lag

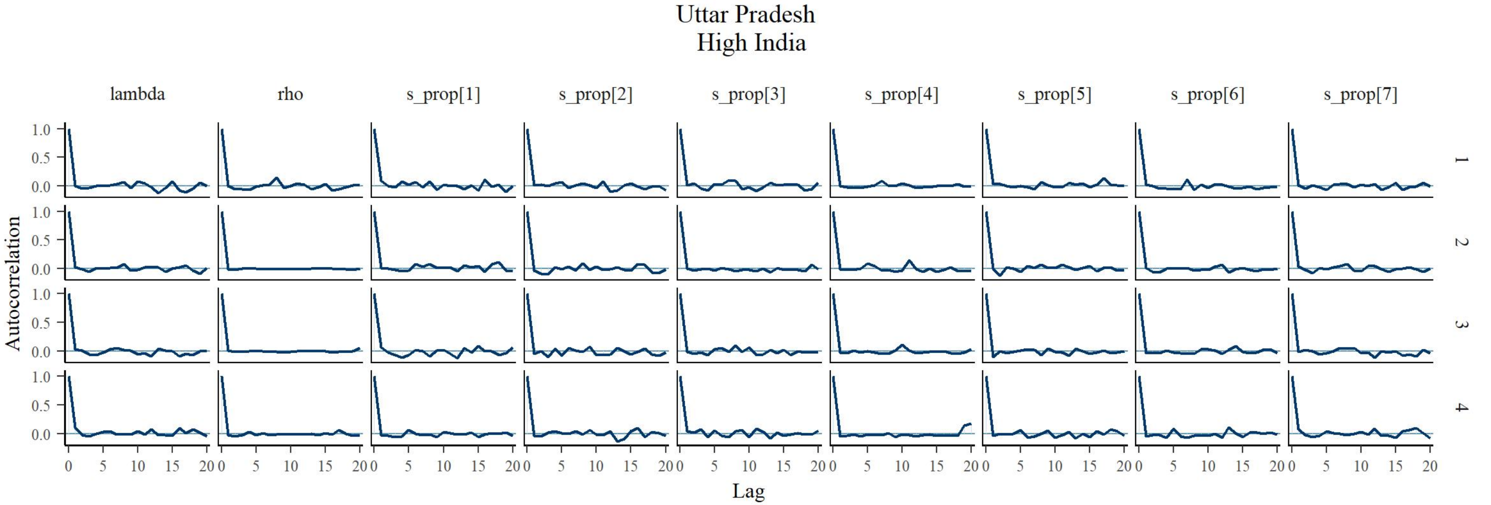

Uttar Pradesh
High India
lambda
rho
s_prop[1]
s_prop[2]
s_prop[3]
s_prop[4]
s_prop[5]
s_prop[6]
s_prop[7]
Autocorrelation
Lag
1
2
3
4

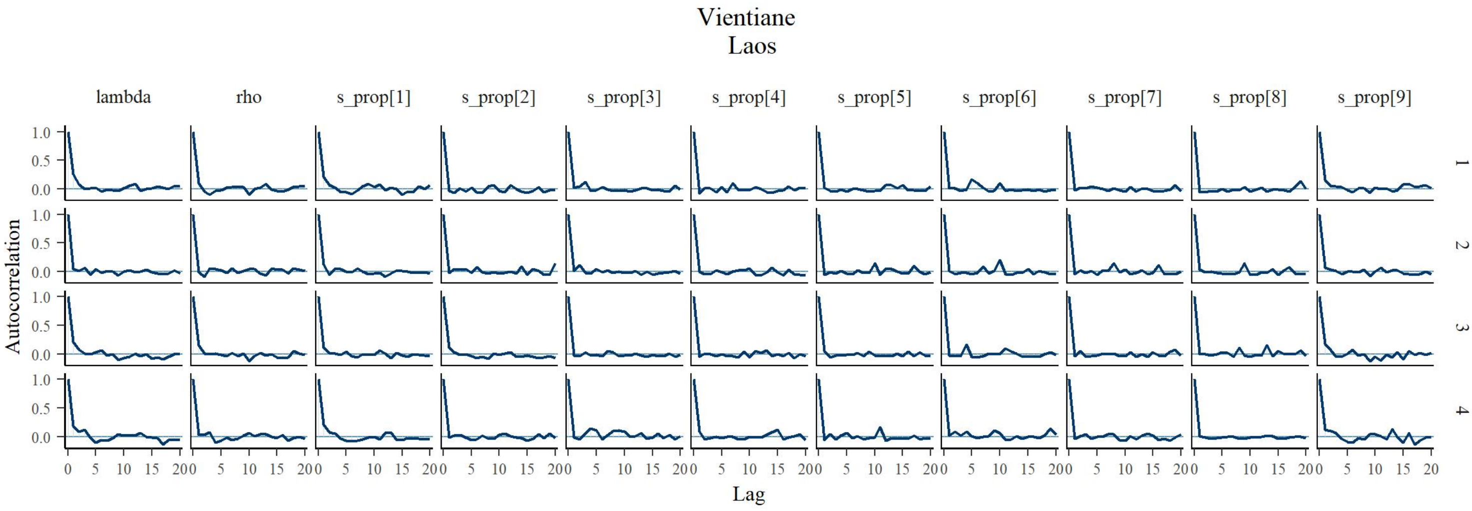

Vientiane
Laos
lambda
rho
s_prop[1]
s_prop[2]
s_prop[3]
s_prop[4]
s_prop[5]
s_prop[6]
s_prop[7]
s_prop[8]
s_prop[9]
1.0
0.5
0.0
1
2
3
4
Autocorrelation
0 5 10 15 20
Lag

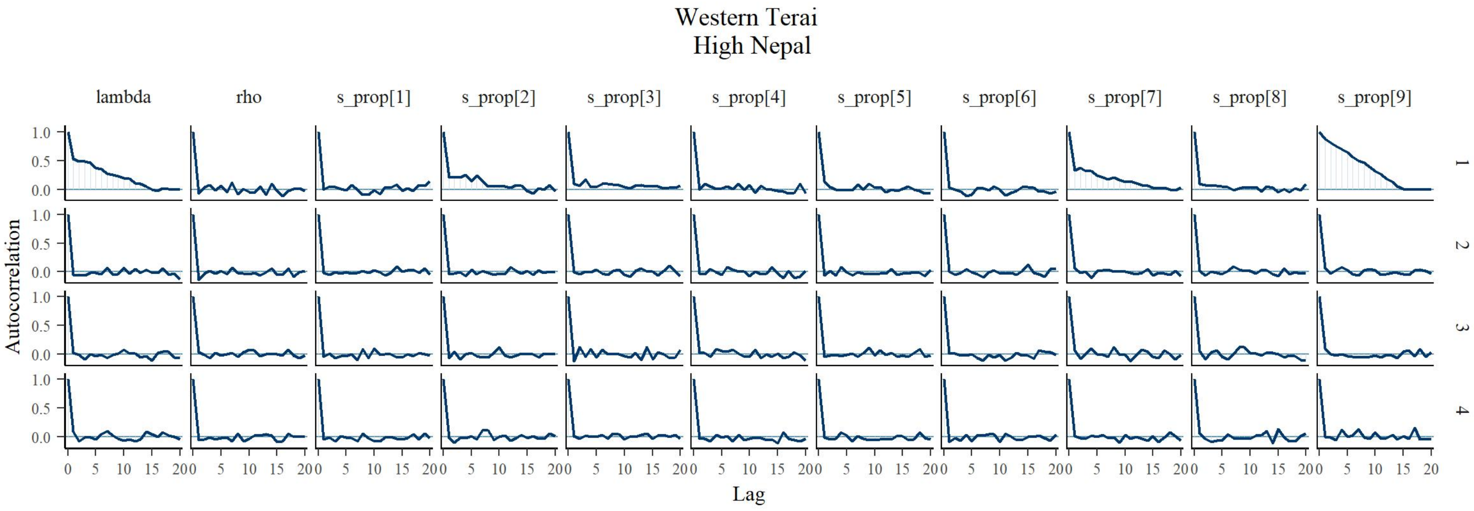
Western Terai
High Nepal
lambda
rho
s_prop[1]
s_prop[2]
s_prop[3]
s_prop[4]
s_prop[5]
s_prop[6]
s_prop[7]
s_prop[8]
s_prop[9]
Autocorrelation
Lag
1
2
3
4

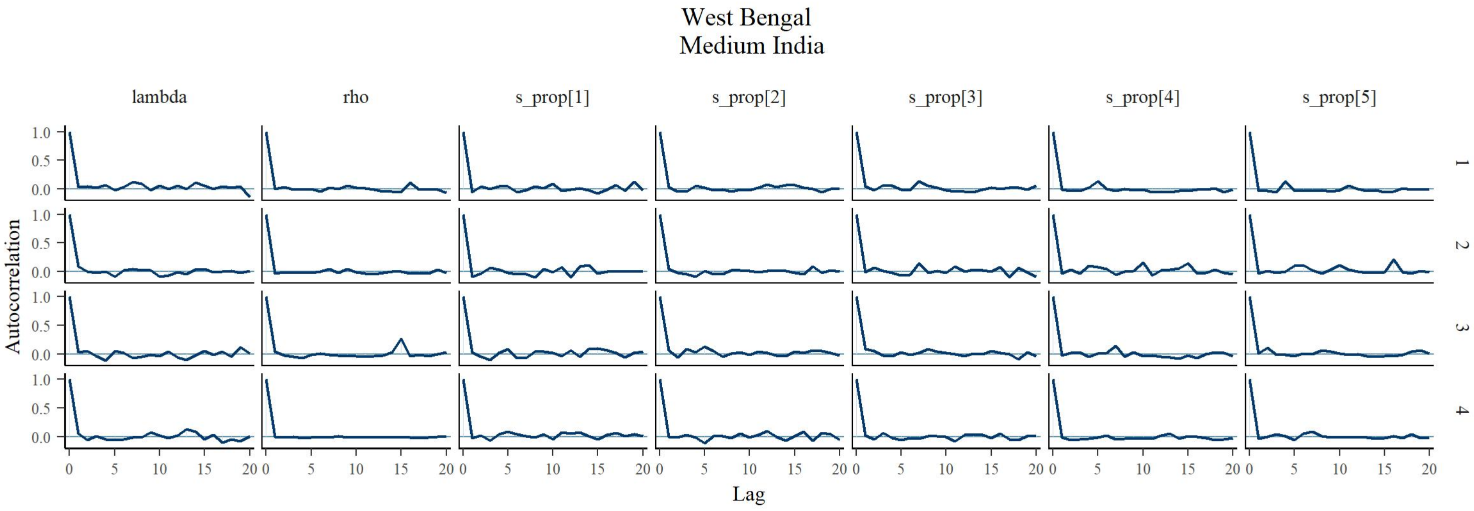

West Bengal
Medium India
lambda
rho
s_prop[1]
s_prop[2]
s_prop[3]
s_prop[4]
s_prop[5]
1
2
3
4
Autocorrelation
Lag
