# Supplementary material for: Estimates of the global burden of Japanese encephalitis and the impact of vaccination from 2000-2015
Source: eLife. 2020 May 26;9:e51027. doi: 10.7554/eLife.51027 (PMC7282807; doi:10.7554/eLife.51027)
Supplement: Figure 5—source data 1. [file elife-51027-fig5-data1.pdf]

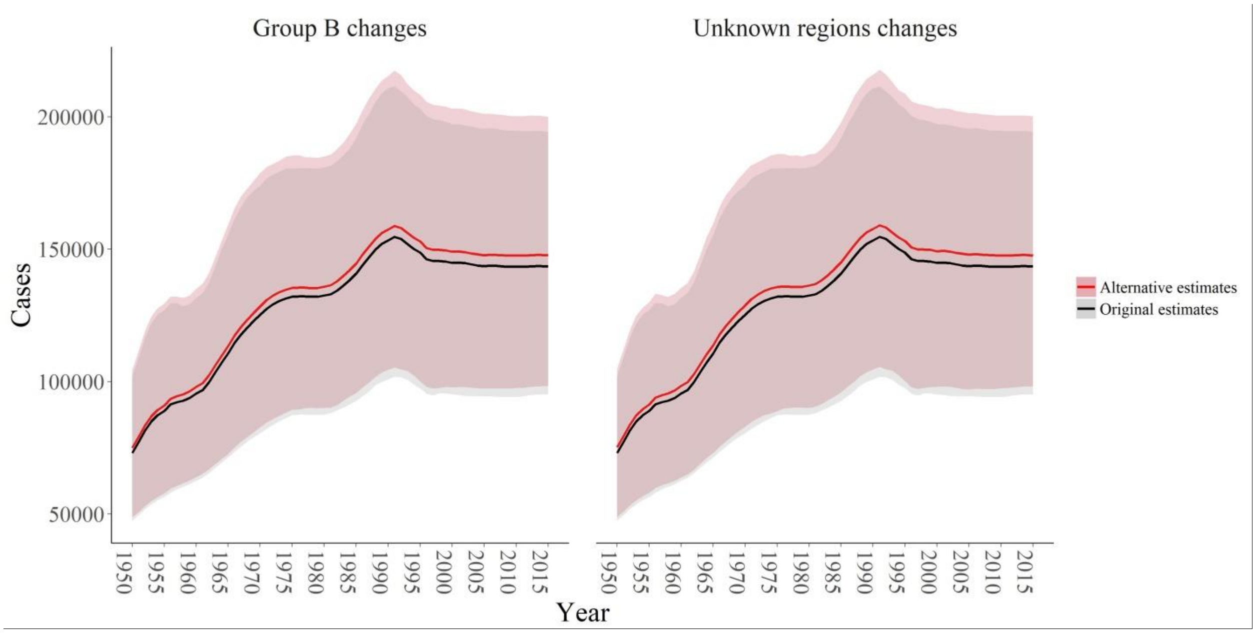

Figure 5- Supp1 A. Numbers of global cases from 1950 to 2015 when changing the FOI for countries with unavailable data. For endemic areas where there was no study conducted, we did a sensitivity analysis to assess how much impact these areas had on the global estimates. For the first assumption, FOI of the Group B endemic areas (Australia, low incidence area in India, Pakistan, North Korea, Russia and Singapore) were sampled from $uniform(0,0.5)$. The second assumption is similar to the first but we sampled from this FOI distribution for all Group B endemic areas and countries for which FOI was inferred from neighboring countries or from countries in the same incidence group (Bhutan, Brunei, Myanmar, Papua New Guinea, and Timor-Leste). Those assumed FOI will be use to generate global cases in the no vaccination scenario and compared with the original estimation. The red ribbon represents our original estimates, the grey ribbon represents the results of sensitivity analysis, each are presented with the 95% credible interval of cases estimated in each scenario, along with the solid lines represent the mean of the cases estimated.

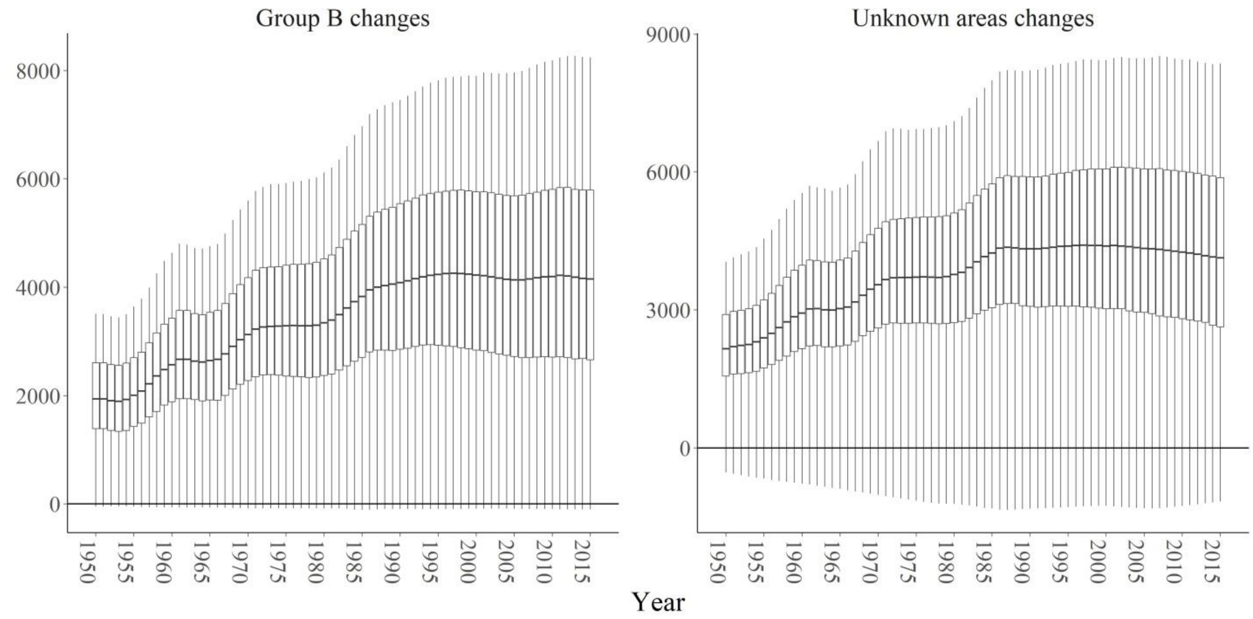

Figure 5- Supp1 B. Differences in numbers of global cases from 1950 to 2015 when changing the FOI for countries with unavailable data. For endemic areas where there was no study conducted, we did a sensitivity analysis to assess how much impact these areas had on the global estimates. For the first assumption, FOI of the Group B endemic areas (Australia, low incidence area in India, Pakistan, North Korea, Russia and Singapore) were sampled from $uniform(0,0.5)$. The second assumption is similar to the first but we sampled from this FOI distribution for all Group B endemic areas and countries for which FOI was inferred from neighboring countries or from countries in the same incidence group (Bhutan, Brunei, Myanmar, Papua New Guinea, and Timor-Leste). Those assumed FOI will be use to generate global cases in the no vaccination scenario and compared with the original estimation. The boxplots represent the differences in generated cases with its 95% credible intervals (also shown 1st quartile, 3rd quartile, and the means)

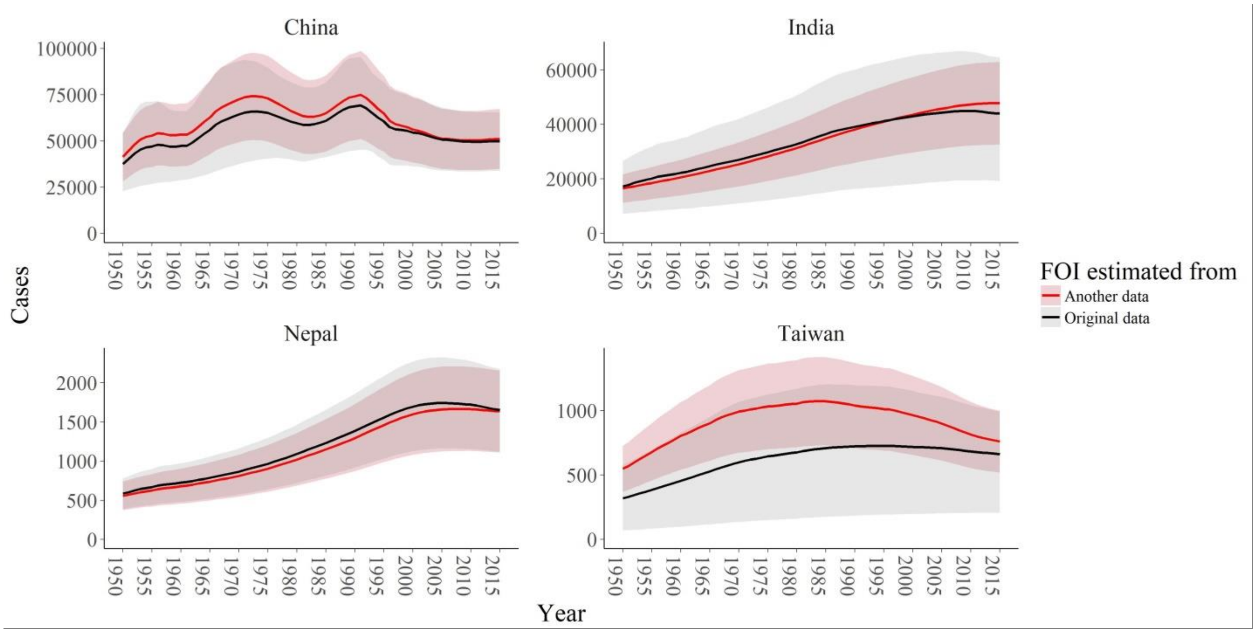

Figure 5- Supp1 C. Numbers of cases from 1950 to 2015 when FOI is estimated from subnational or national data. The red ribbon represents our original estimates, the grey ribbon represents the results of sensitivity analysis, each are presented with the 95% credible interval of cases estimated in each scenario, along with the solid lines represent the mean of the cases estimated.

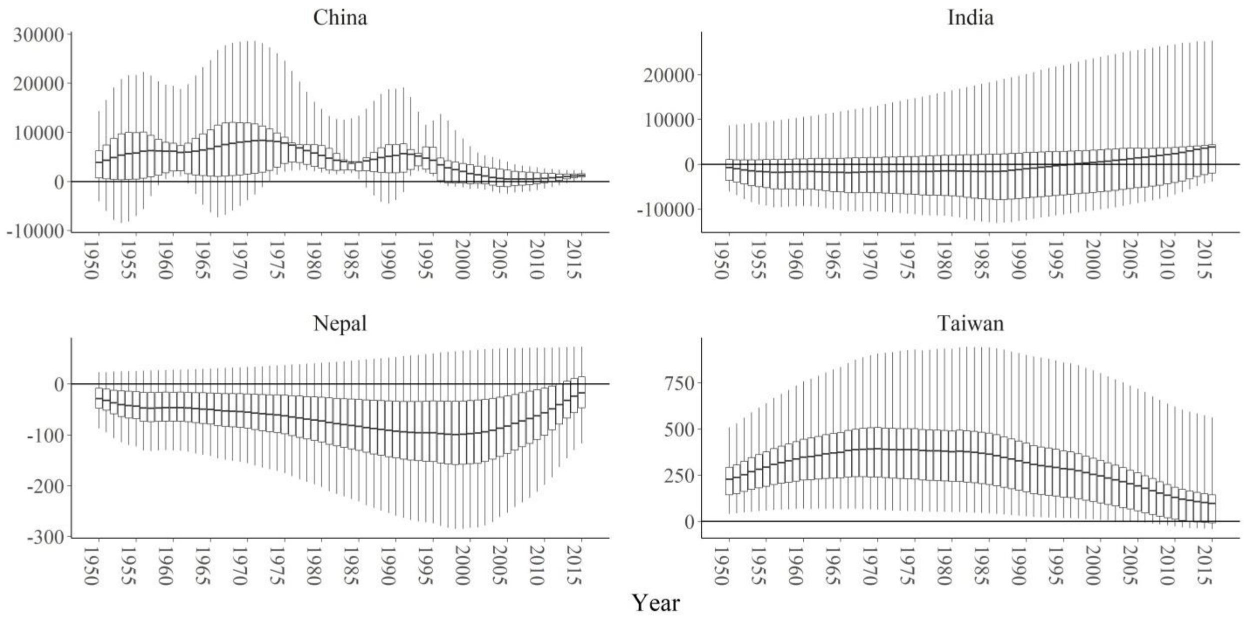

Figure 5- Supp1 D. Differences in numbers of cases from 1950 to 2015 when FOI is estimated from subnational or national data. The boxplots represent the differences in generated cases with its 95% credible intervals (also shown 1st quartile, 3rd quartile, and the means).

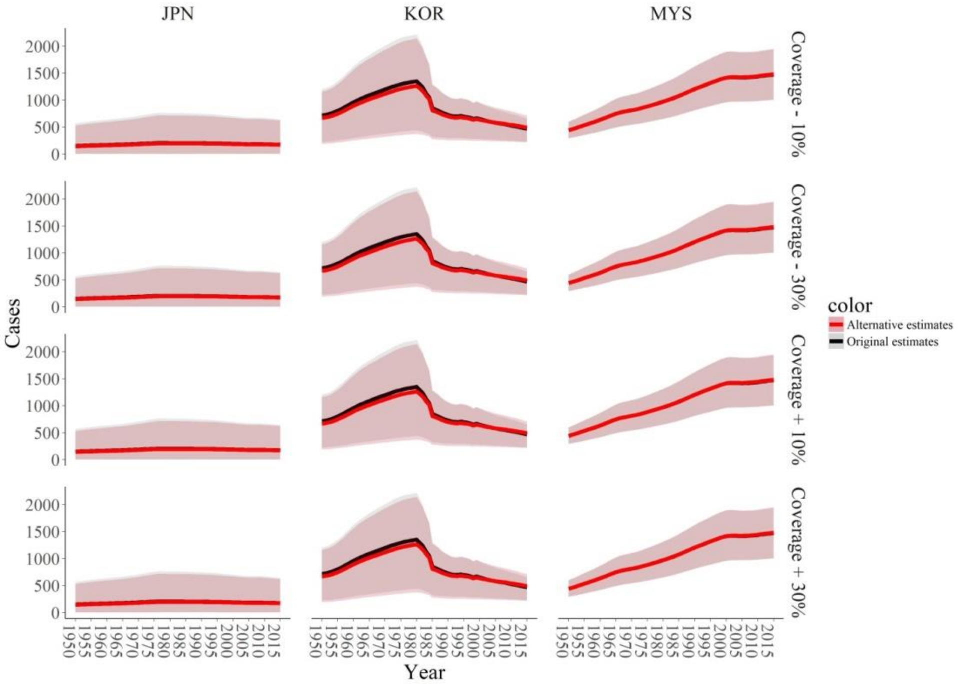

Figure 5- Supp1 E. Numbers of cases from 1950 to 2015 when changing the vaccine coverage in countries that used MBDV. South Korea, Malaysia and Japan are countries which used MBDV intensively in the past. This vaccine required 2 or 3 doses, hence it is unsure which doses coverage numbers were reported. Therefore, we conducted a sensitivity analysis to address this problem by increasing and decreasing the number of vaccinated people by 10% and 30%), The new coverage then used to generate new estimated cases in each country. The red ribbon represents our original estimates, the grey ribbon represents the results of sensitivity analysis, each are presented with the 95% credible interval of cases estimated in each scenario, along with the solid lines represent the mean of the cases estimated. Abbreviation: JPN: Japan, KOR: South Korea, MYS: Malaysia

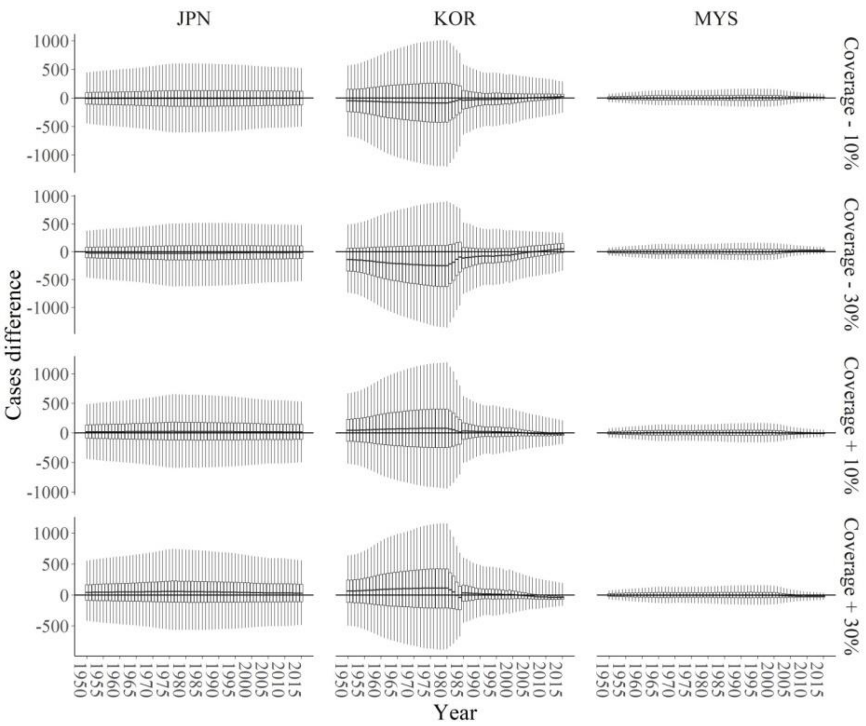

Figure 5- Supp1 F. Differences in numbers of cases from 1950 to 2015 when changing the vaccine coverage in countries that used MBDV. South Korea, Malaysia and Japan are countries which used MBDV intensively in the past. This vaccine required 2 or 3 doses, hence it is unsure which doses coverage numbers were reported. Therefore, we conducted a sensitivity analysis to address this problem by increasing and decreasing the number of vaccinated people by 10% and 30%). The new coverage then used to generate new estimated cases in each country. The boxplots represent the differences in generated cases with its 95% credible intervals (also shown 1st quartile, 3rd quartile, and the means). Abbreviation: JPN: Japan, KOR: South Korea, MYS: Malaysia.

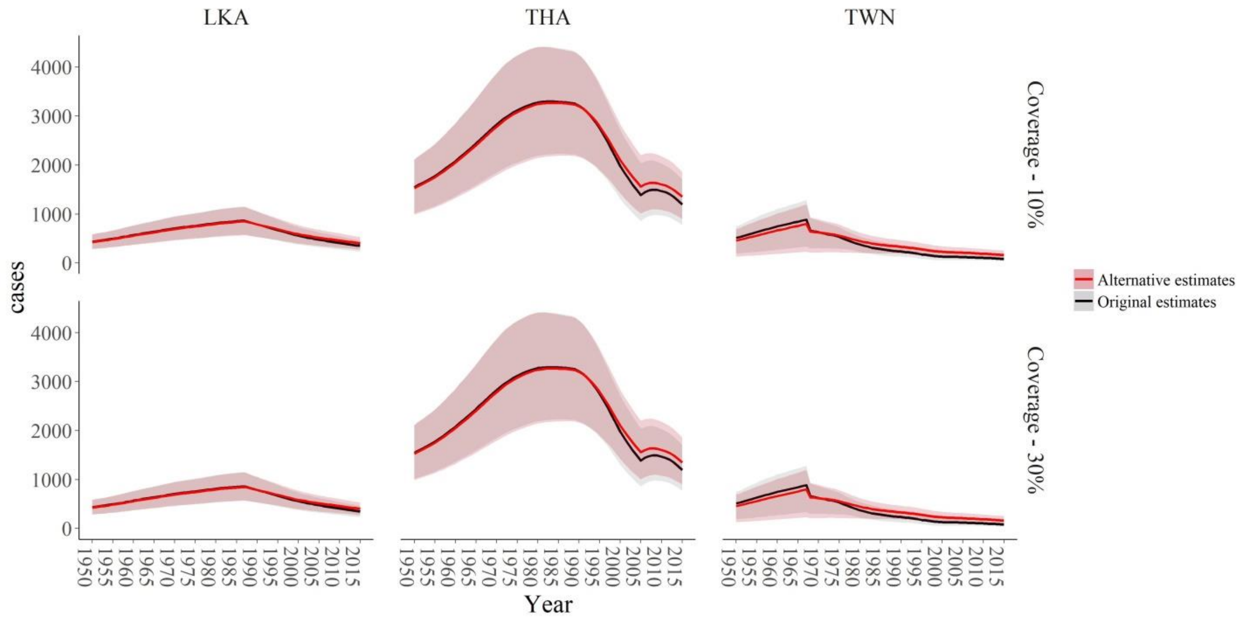

Figure 5- Supp1 G. Numbers of cases from 1950 to 2015 when changing the vaccine coverage in countries that have unclear coverage data. For Taiwan, Thailand, and Sri Lanka, information of vaccine coverage were unavailable and were assumed to be 99% coverage in our model. We therefore undertook sensitivity analysis by decreasing the number of vaccinated people by 10% and 30%. The red ribbon represents our original estimates, the grey ribbon represents the results of sensitivity analysis, each are presented with the 95% credible interval of cases estimated in each scenario, along with the solid lines represent the mean of the cases estimated.. Abbreviation: LKA: Sri Lanka, THA: Thailand, TWN: Taiwan.

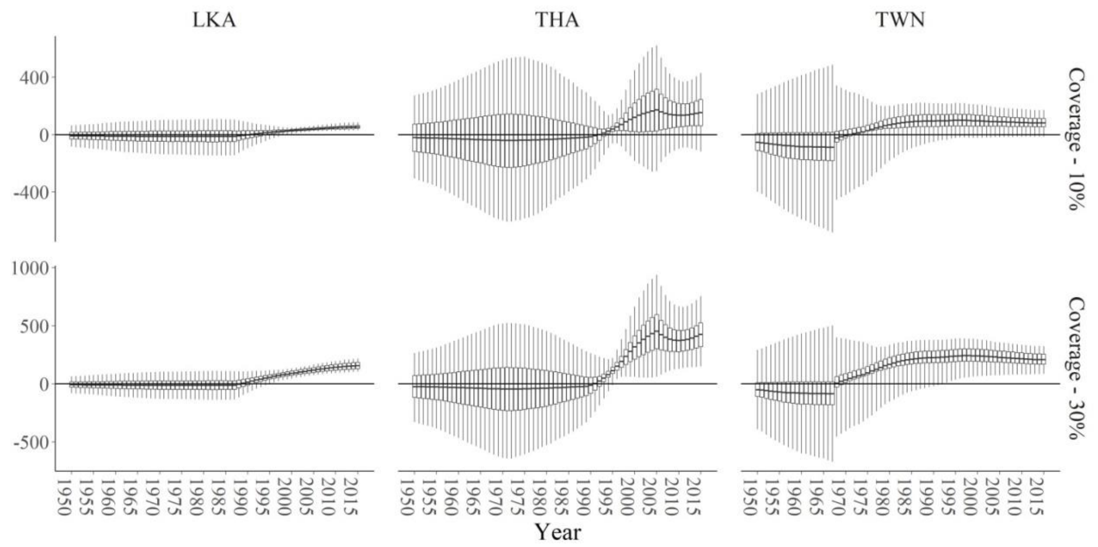

Figure 5- Supp1 H. Differences in numbers of cases from 1950 to 2015 when changing the vaccine coverage in countries that have unclear coverage data. For Taiwan, Thailand, and Sri Lanka, information of vaccine coverage were unavailable and were assumed to be 99% coverage in our model. We therefore undertook sensitivity analysis by decreasing the number of vaccinated people by 10% and 30%. The boxplots represent the differences in generated cases with its 95% credible intervals (also shown 1st quartile, 3rd quartile, and the means). Abbreviation: LKA: Sri Lanka, THA: Thailand, TWN: Taiwan**.**

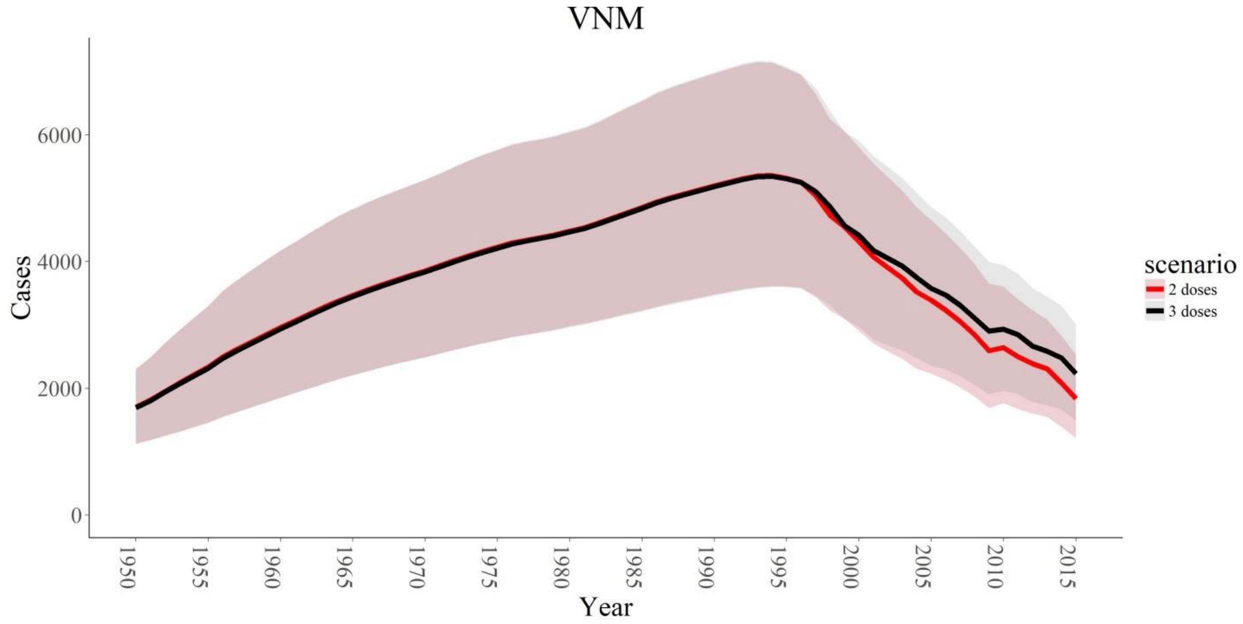

Figure 5- Supp1 I. Numbers of cases from 1950 to 2015 when using different coverage data in Vietnam. In Vietnam, the coverage data of 2 and 3 doses is available and then used to generate JE cases under the 2 schemes. The red ribbon represents our original estimates, the grey ribbon represents the results of sensitivity analysis, each are presented with the 95% credible interval of cases estimated in each scenario, along with the solid lines represent the mean of the cases estimated.Abbreviation: VNM: Vietnam

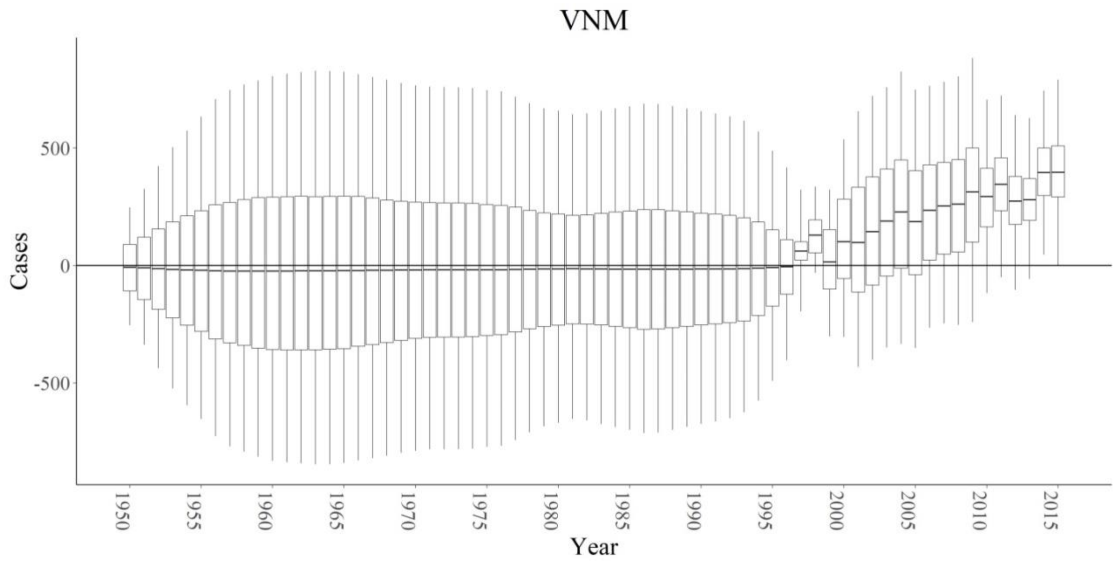

Figure 5- Supp1 J. Differences in numbers of cases from 1950 to 2015 when using different coverage data in Vietnam. In Vietnam, the coverage data of 2 and 3 doses is available and then used to generate JE cases under the 2 schemes. The boxplots represent the differences in generated cases with its 95% credible intervals (also shown 1st quartile, 3rd quartile, and the means).

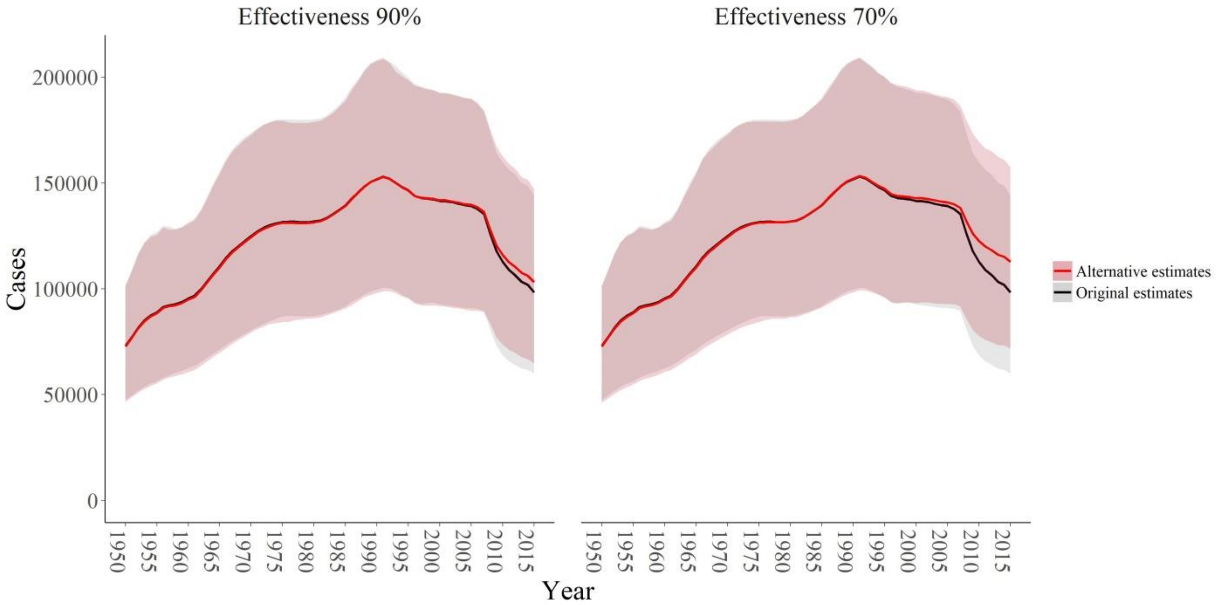

Figure 5- Supp1 K. Numbers of global cases from 1950 to 2015 when changing the vaccine effectiveness. We generated global cases with vaccine effectiveness changed to 90%, 70% The red ribbon represents our original estimates, the grey ribbon represents the results of sensitivity analysis, each are presented with the 95% credible interval of cases estimated in each scenario, along with the solid lines represent the mean of the cases estimated.

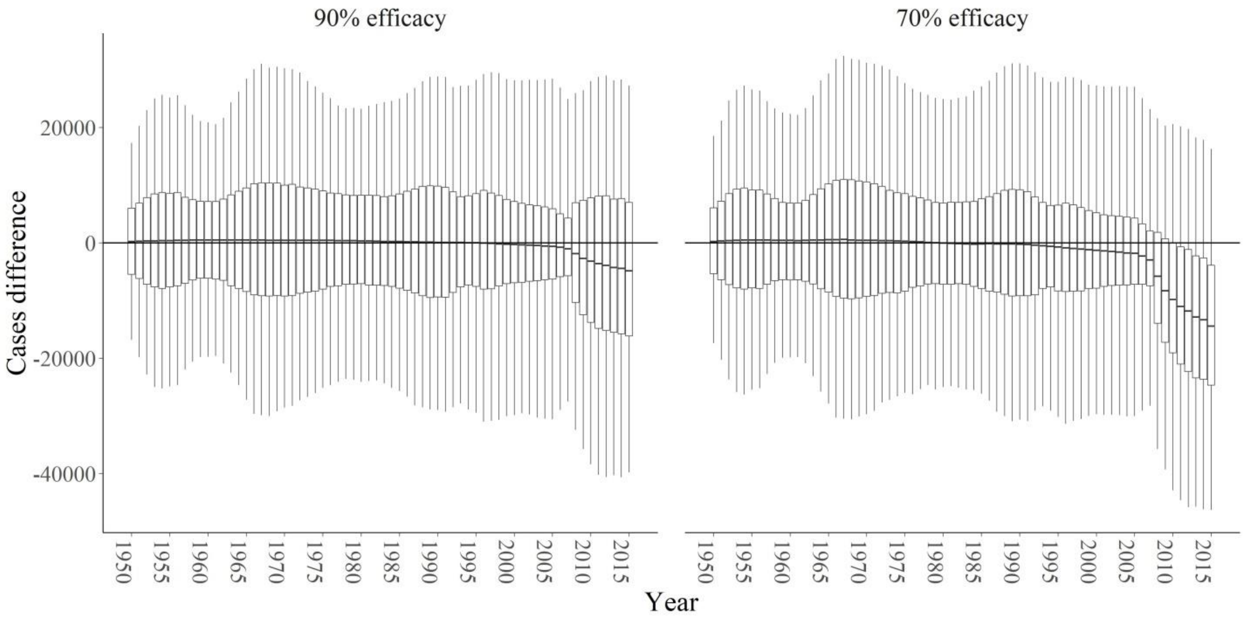

Figure 5- Supp1 L. Differences in numbers of global cases from 1950 to 2015 when changing the vaccine effectiveness. We generated global cases with vaccine effectiveness changed to 90%, 70%The boxplots represent the differences in generated cases with its 95% credible intervals (also shown 1st quartile, 3rd quartile, and the means).

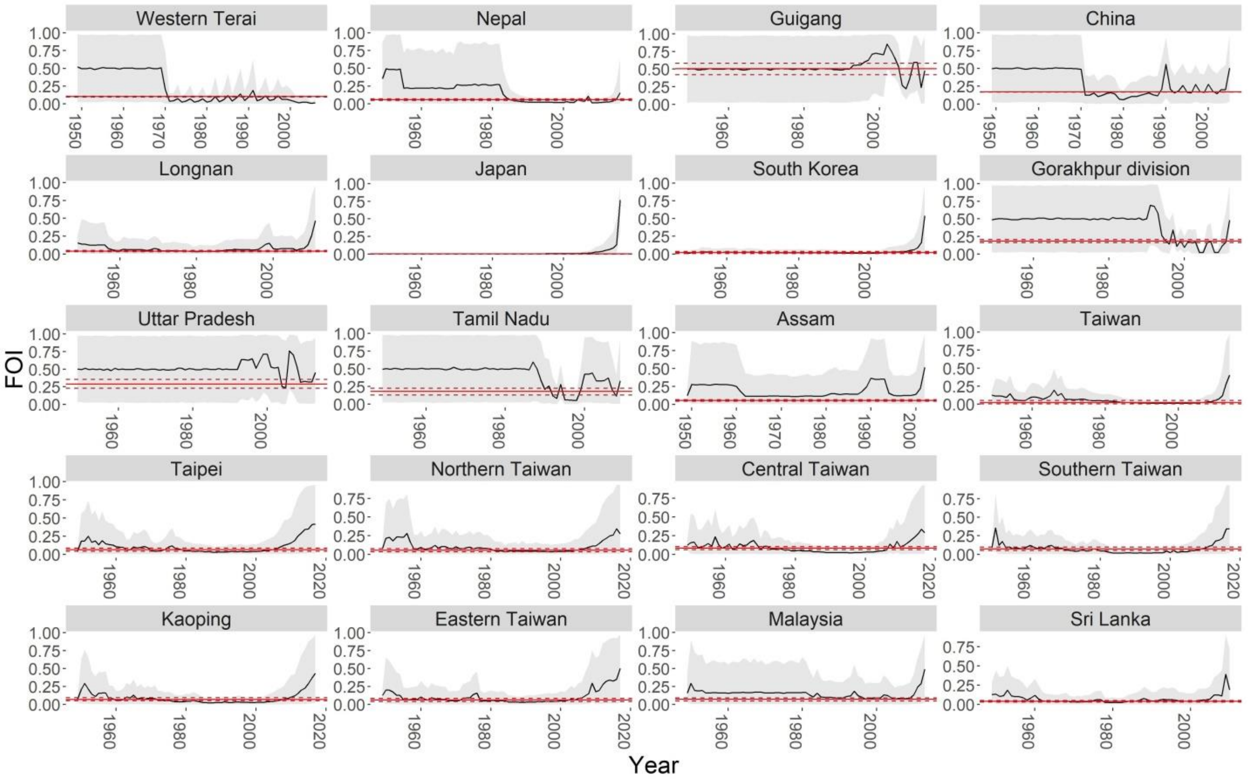

Figure 5- Supp1 M: Time dependent and constant FOI estimated from multiple years data. The grey ribbons and the solid black lines show the annual FOI estimated from time dependent model (lower bound and upper bound of the ribbons represent the 2.5% and 97.5% quantiles, and the black lines represent the mean estimates). The horizontal red lines show the constant FOI (lower and upper dash lines represent the 2.5% and 97.5% quantiles, and the solid red lines represent the mean estimates). In other to test our assumption that the estimated FOI is constant overtime, we fitted multiple years data set to a time-dependent catalytic model. For each catchment area c which has k study, each study start at year $y^l_{c,k}$ and stop at $y^u_{c,k}$, the proportion of infections in a specific age group i is now become:

$$P_{c,k,i} \; = \; \exp\left\{-\sum_{a^l = 0}^{age^l_{c,k,i}-1}\left(\sum_{y=\,y^l_{c,k}-\,a^l}^{y^u_{c,k}-\,a^l}\lambda_{c,y}\right)\right\} - \exp\left\{-\sum_{a^u = 0}^{age^u_{c,k,i}}\left(\sum_{y=\,y^l_{c,k}-\,a^u}^{y^u_{c,k}-\,a^u}\lambda_{c,y}\right)\right\} \quad (1)$$

With $\lambda_{c,y}$ is FOI of each year y and catchment area c. $age^l_{c,k,i}$ and $age^u_{c,k,i}$ are the lower bound and upper bound of the age group i. If $age^l_{c,k,i} = 0$, the minuend of (1) becomes 1.

Parameter $\lambda_{c,y}$ was estimated in logit scale with weak informative priors $normal(0{,}1000)$. Every annual FOI from under the year 1950 were grouped into one group.
